# Correction: Bosica, G.; Abdilla, R. Novel Biopolymer-Based Catalyst for the Multicomponent Synthesis of *N*-aryl-4-aryl-Substituted Dihydropyridines Derived from Simple and Complex Anilines. *Molecules* 2024, *29*, 1884

**DOI:** 10.3390/molecules30214231

**Published:** 2025-10-30

**Authors:** Giovanna Bosica, Roderick Abdilla

**Affiliations:** Green Synthetic Organic Chemistry Lab, Department of Chemistry, University of Malta, 2080 Msida, Malta

## Text Correction

There was an error in the original publication [1], a wrong data description for the compound labeled 12.

A correction has been made to 3. Experimental, 3.5. Product Characterization Data:

**[12]** *1-[4-[(4-bromophenyl)(1H-indol-3-yl)methyl]phenyl]-7,7-dimethyl-4-(3-nitrophenyl)-4,6,7,8-tetrahydroquinoline-2,5(1H,3H)-dione.* Novel white solid. M.P. 160–162 °C. IR (KBr) (ν, cm^−1^): 3358, 3084, 3055, 2957, 2928, 2868, 1699, 1624, 1526, 1506, 1485, 1456, 1377, 1348, 1296, 1265, 1223, 1200, 1140, 988, 961, 908, 806, 791, 741, 621. ^1^H NMR (500 MHz, Chloroform-*d*) δ 8.16 (t, *J* = 2.1 Hz, 1H), 8.11 (ddt, *J* = 8.3, 2.1, 0.9 Hz, 1H), 8.08 (s, 1H), 7.67 (d, *J* = 7.7 Hz, 1H), 7.51 (t, *J* = 7.9 Hz, 1H), 7.45 (d, *J* = 8.5 Hz, 2H), 7.40 (d, *J* = 8.3 Hz, 1H), 7.35 (d, *J* = 8.0 Hz, 2H), 7.24–7.21 (m, 1H), 7.21–7.14 (m, 3H), 7.14–7.05 (m, 2H), 7.03 (dddd, *J* = 8.0, 6.9, 3.5, 1.0 Hz, 1H), 6.62 (td, *J* = 2.6, 1.1 Hz, 1H), 5.71 (s, 1H), 4.59 (d, *J* = 7.5 Hz, 1H), 3.17 (dd, *J* = 16.1, 7.8 Hz, 1H), 2.99 (dd, *J* = 16.3, 1.7 Hz, 1H), 2.34 (s, 2H), 2.22 (d, *J* = 17.6 Hz, 1H), 2.12 (d, *J* = 17.8 Hz, 1H), 1.09 (d, *J* = 2.3 Hz, 3H), 1.05 (d, *J* = 2.9 Hz, 3H). ^13^C NMR (126 MHz, Chloroform-*d*) δ 195.67, 169.14, 154.48, 148.58, 144.49, 143.44, 142.19/142.14, 136.75, 134.83, 133.71, 131.60, 130.77, 130.58, 130.41, 130.22, 130.06, 129.18, 128.06, 126.60, 124.16/124.14, 122.40, 122.29, 121.26, 120.51, 119.60, 119.59, 119.55, 118.65/118.61, 116.21/116.19, 111.33, 50.02, 47.95/47.93, 41.80, 38.75, 33.25, 33.12, 28.45, 28.17. ES(+) *m*/*z* = 674.52 [M+H], 607.84, 579.83, 565.82, 551.75, 534.46, 523.73, 453.51, 391.40.

The authors state that the scientific conclusions are unaffected. This correction was approved by the Academic Editor. The original publication has also been updated.
