# Supplementary material for: Novel Biopolymer-Based Catalyst for the Multicomponent Synthesis of N-aryl-4-aryl-Substituted Dihydropyridines Derived from Simple and Complex Anilines
Source: Molecules. 2024 Apr 20;29(8):1884. doi: 10.3390/molecules29081884 (PMC11053630; doi:10.3390/molecules29081884)
Supplement: Supplementary file 1 [file molecules-29-01884-s001.zip › molecules-2940825-supplementary.pdf]

1.1. Product 5a: 2-amino-7,8-dimethyl-5-oxo-1,4-diphenyl-1,4,5,6,7,8 hexahydroquinoline-3-carbonitrile

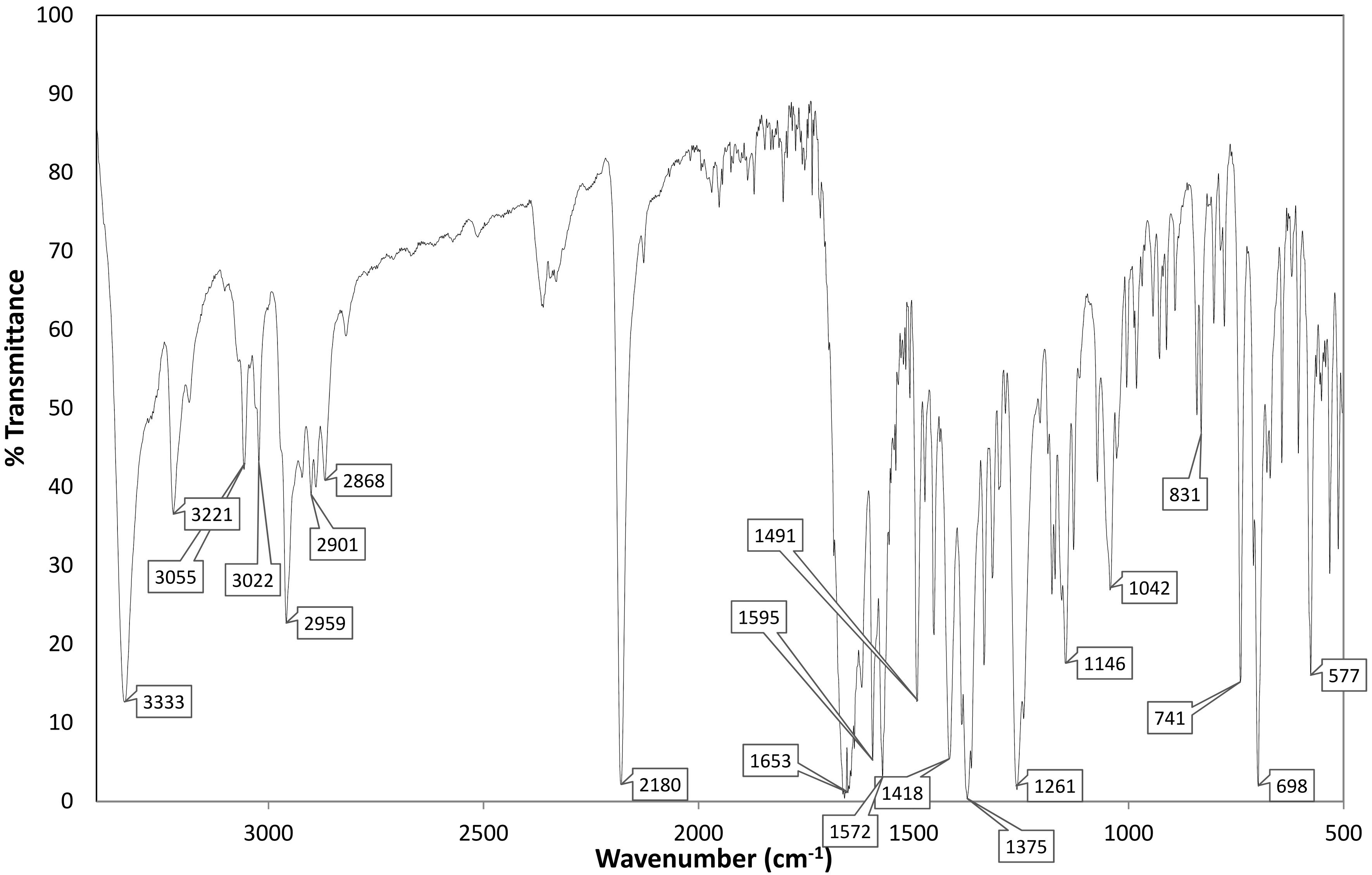

Figure S1 - IR spectrum of 5a

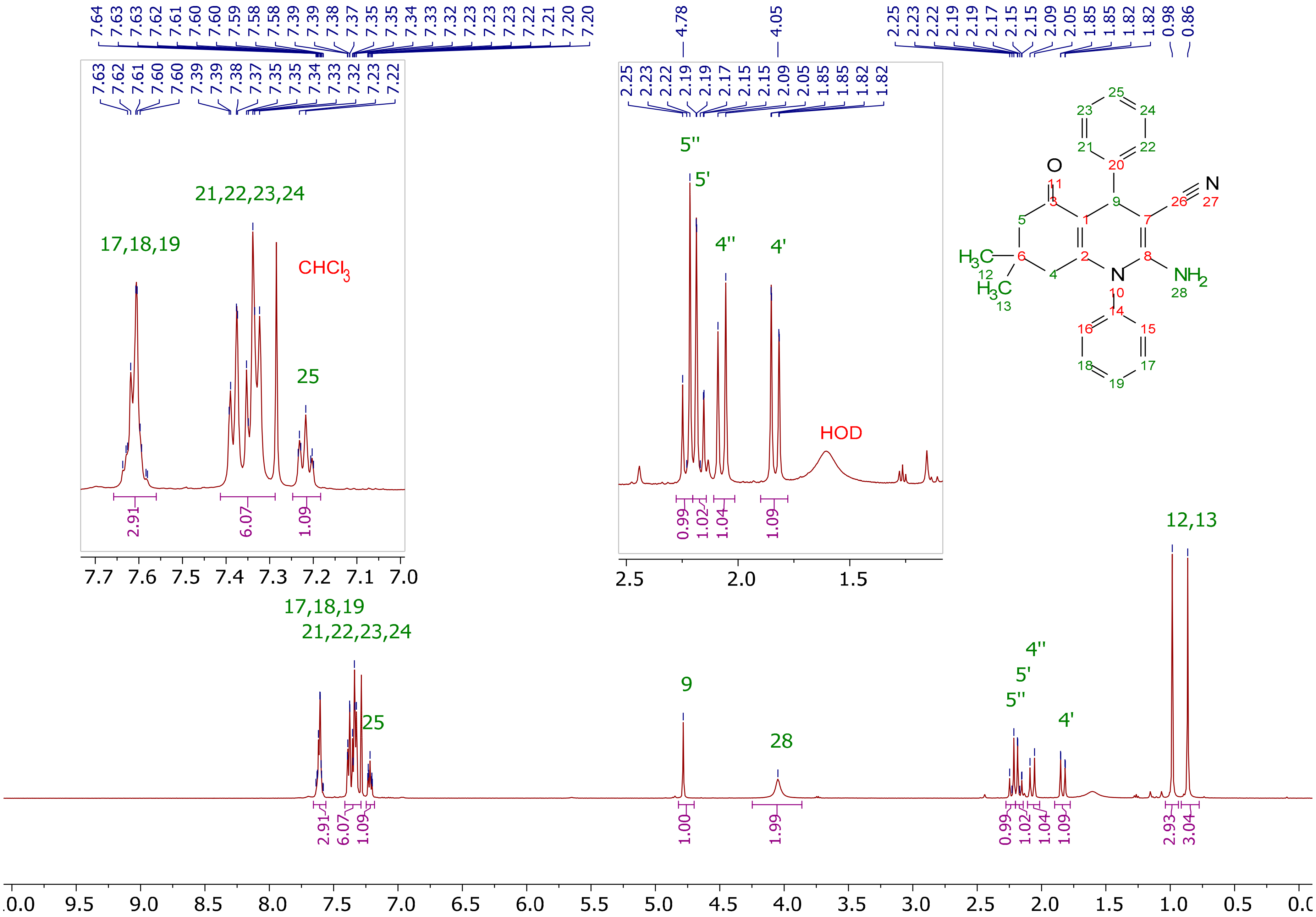

Figure S2 - ^{1}H NMR spectrum of 5a

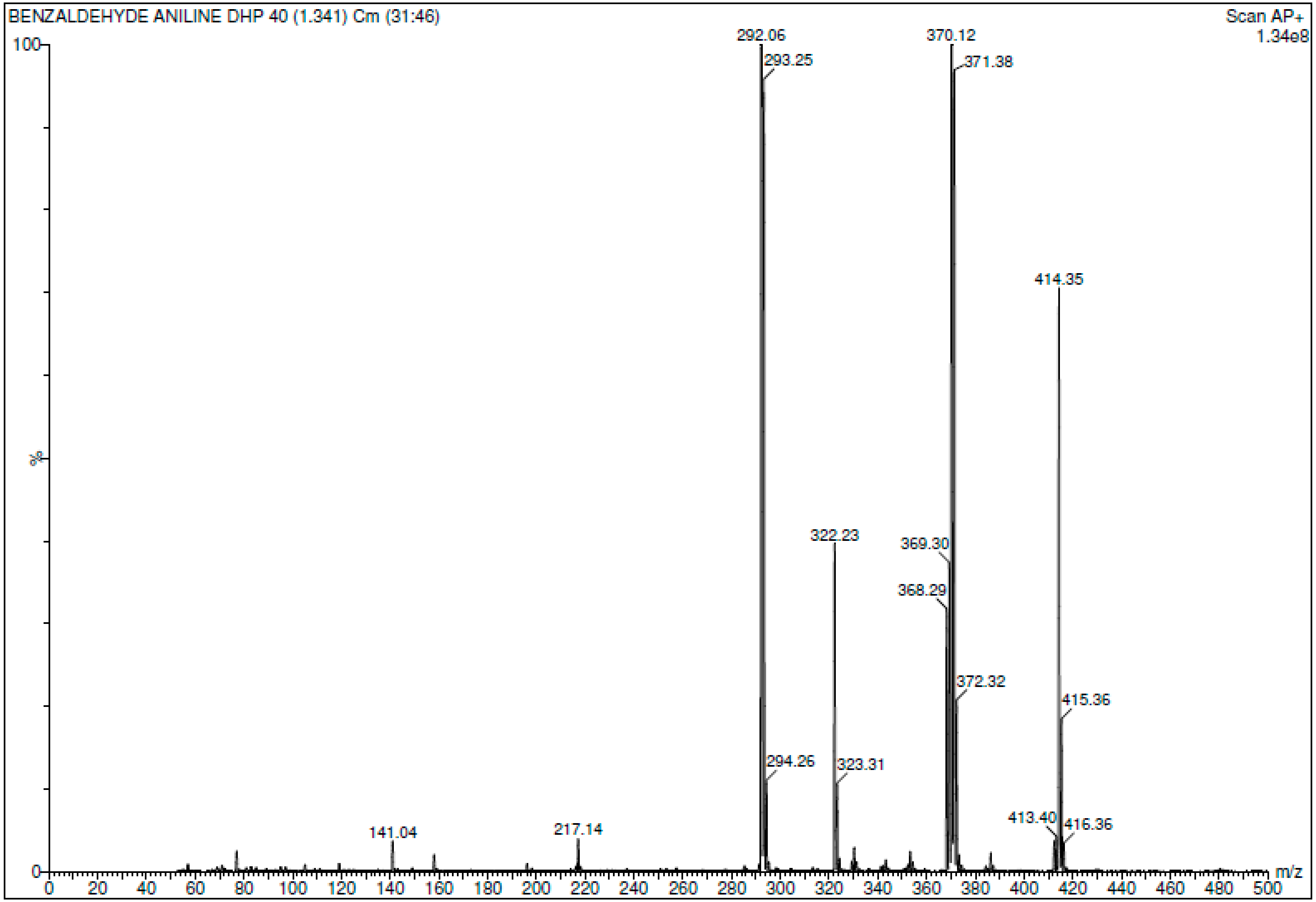

Figure S3 - MS spectrum of 5a

Table S1 - Fragmentation positions for peaks in MS spectrum of 5a

m/z	Fragmentation position and structure
**370.12**	$[M-H]^+$
**323.31**	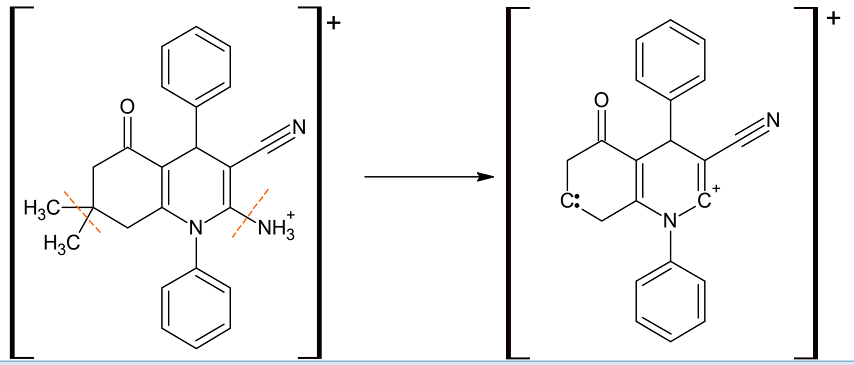
**292.06**	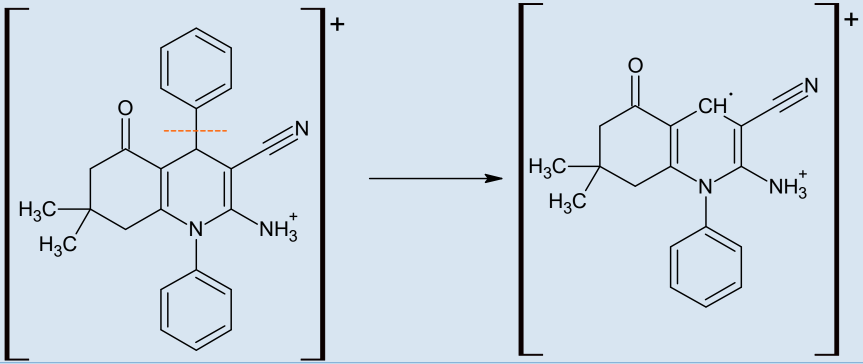
**217.14**	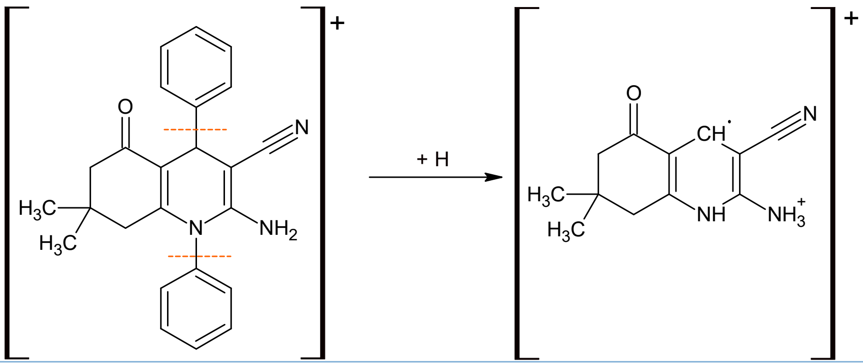

1.2. Product 5b: 2-amino-7,8-dimethyl-4-(4-methylphenyl)-5-oxo-1-phenyl-1,4,5,6,7,8-hexahydroquinoline-3-carbonitrile

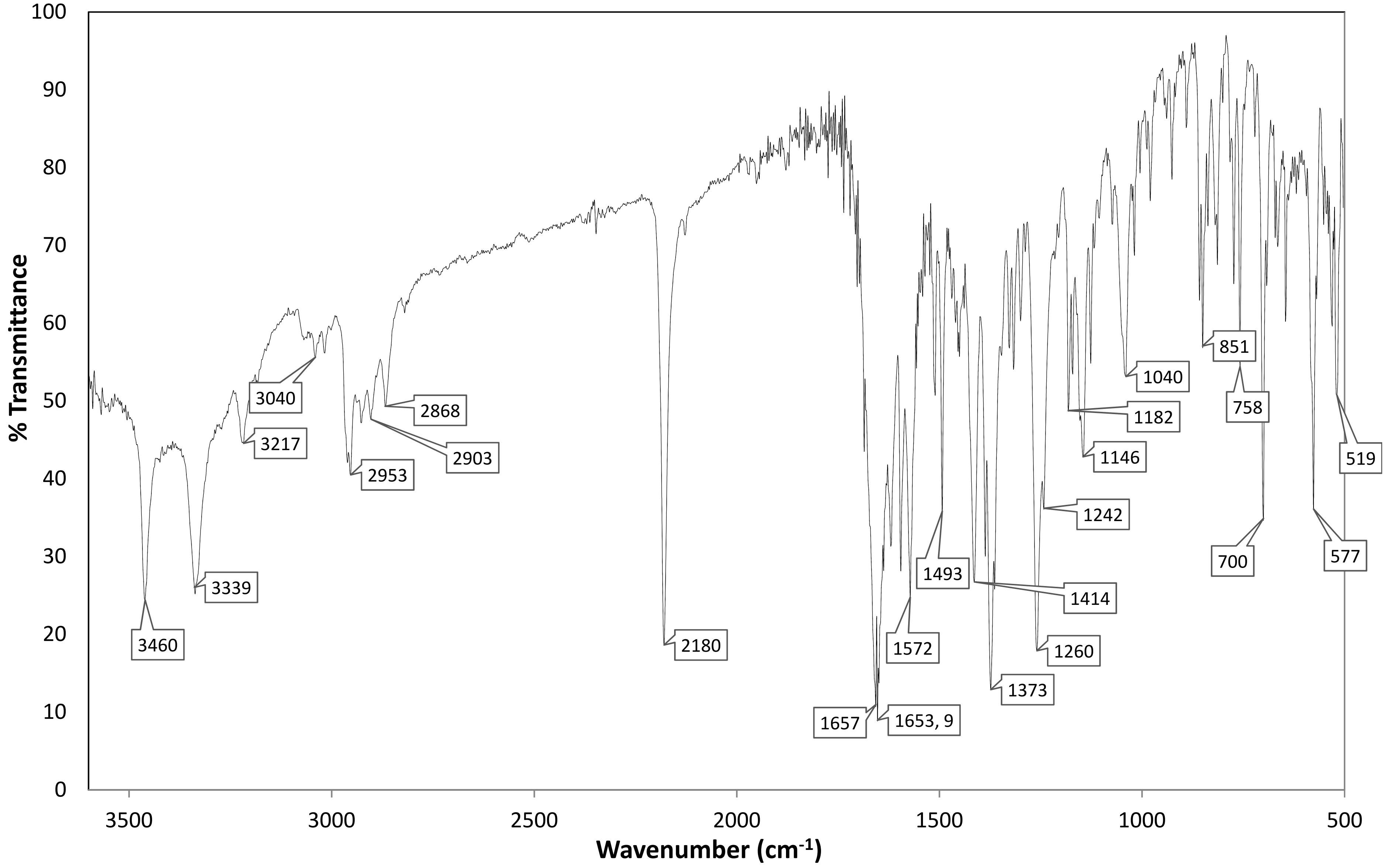

Figure S4 - IR spectrum of 5b

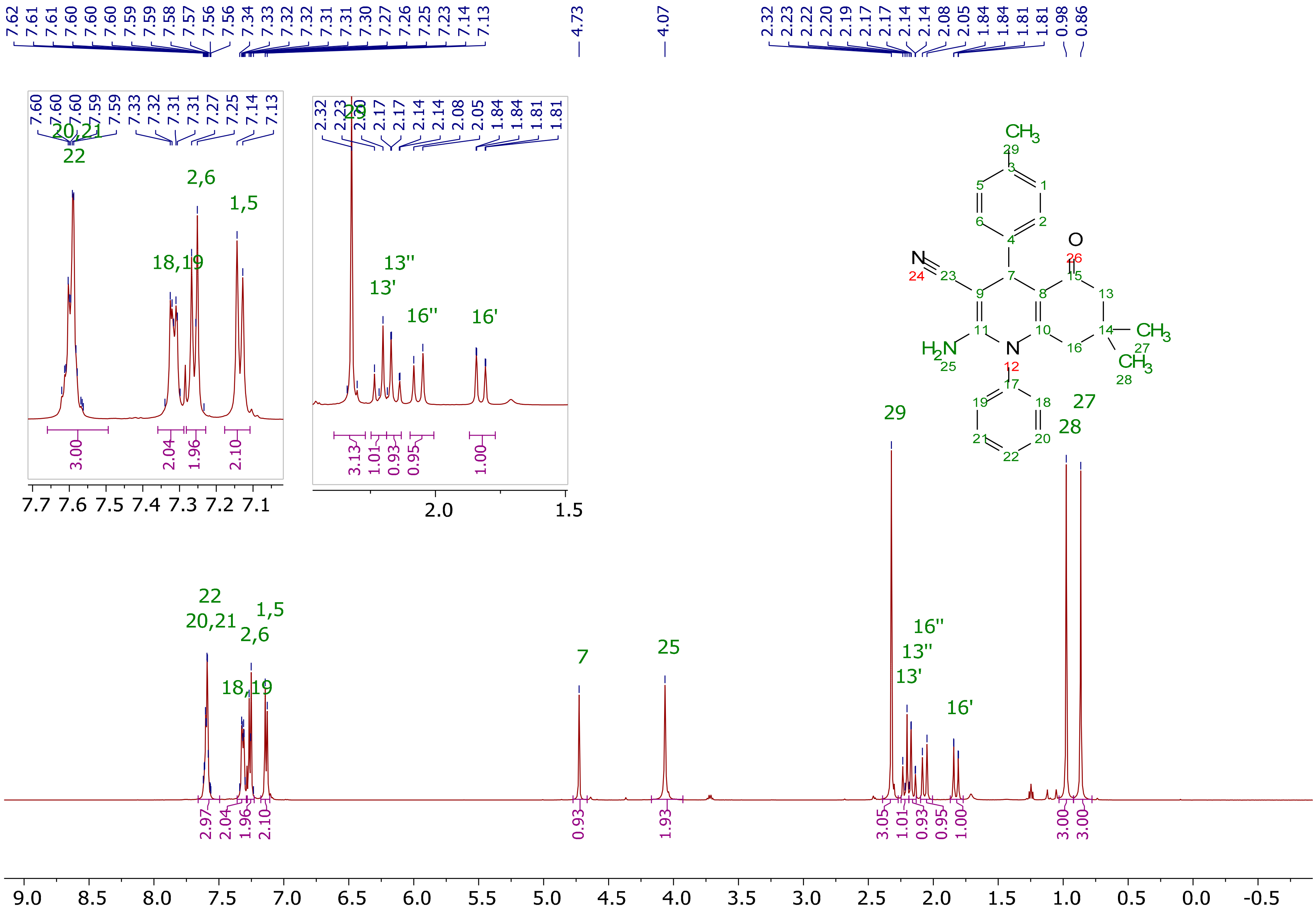

Figure S5 - ^{1}H NMR spectrum of 5b

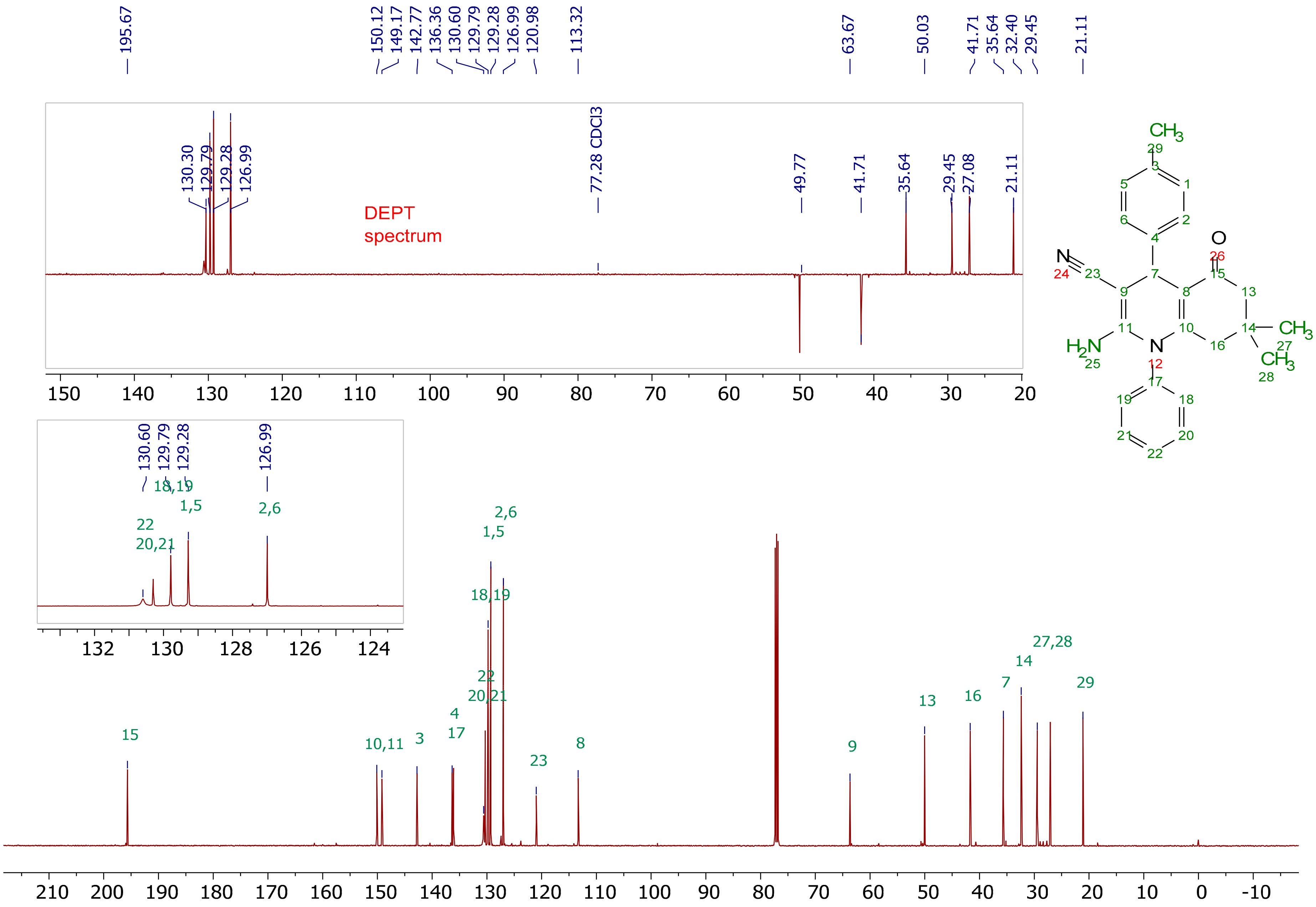

Figure S6 – ^{13}C NMR spectrum of 5b with inset expansion and DEPT spectrum

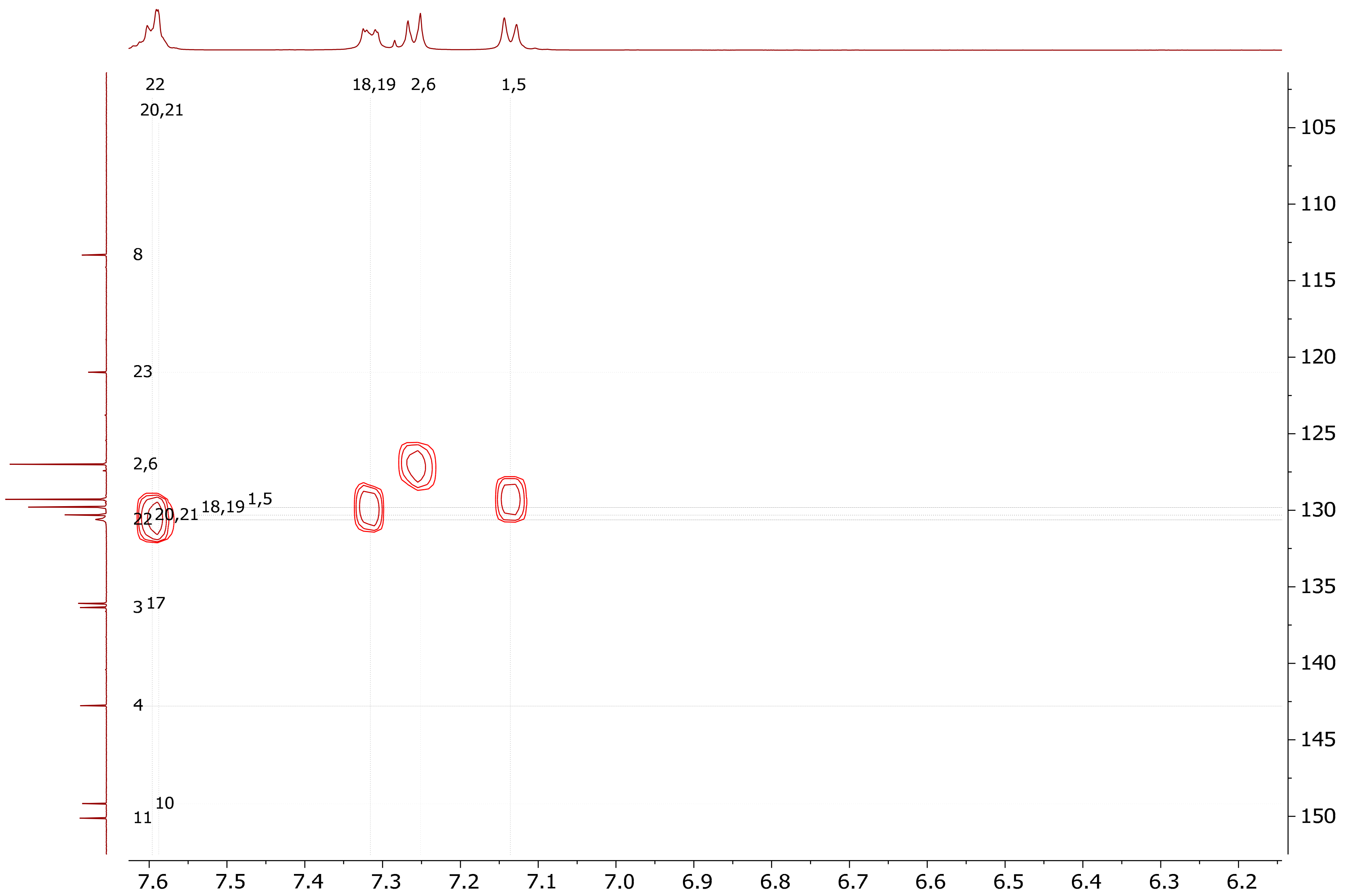

Figure S7 - HSQC spectrum of 5b

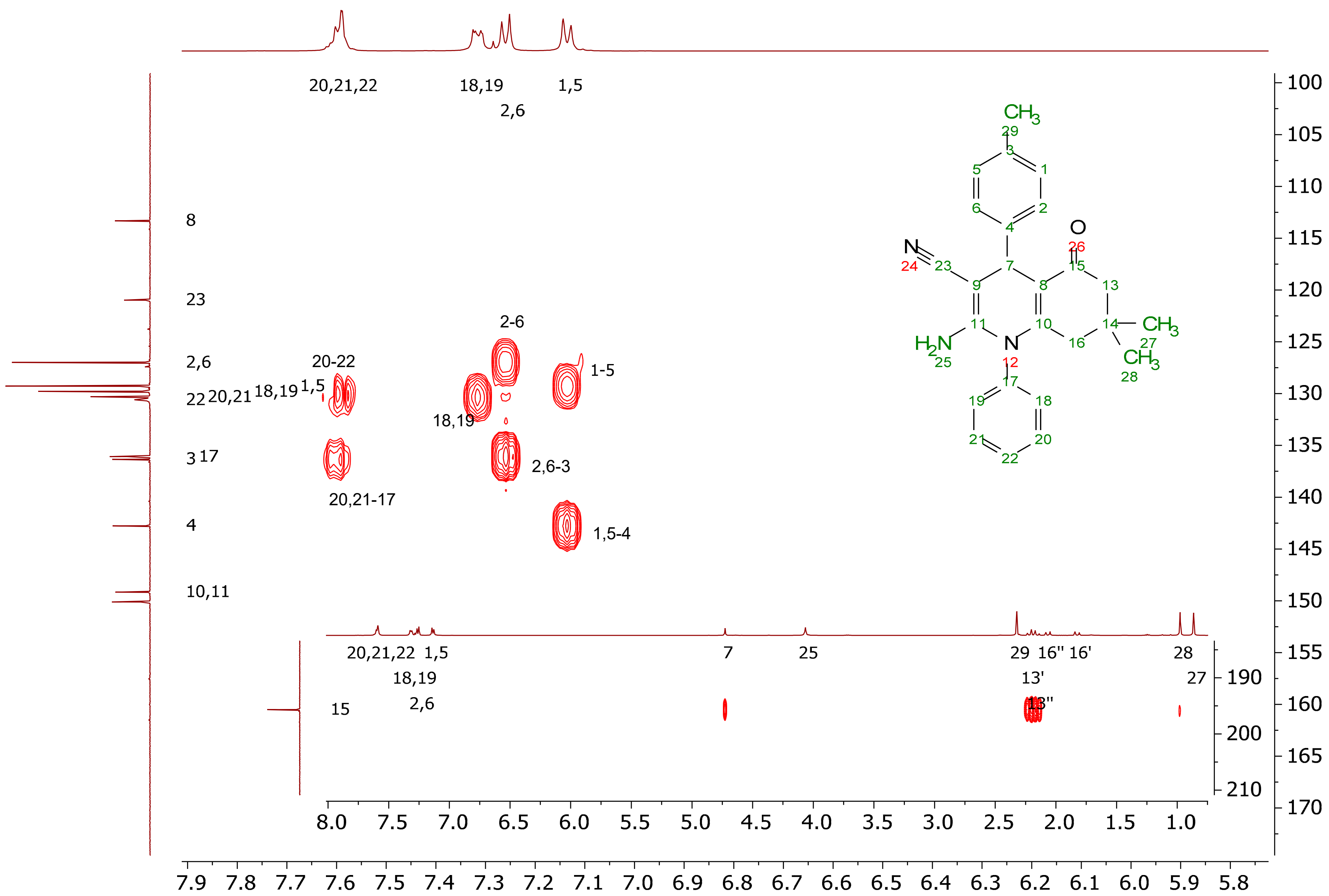

Figure S8 - Downfield region of HMBC spectrum of 5b

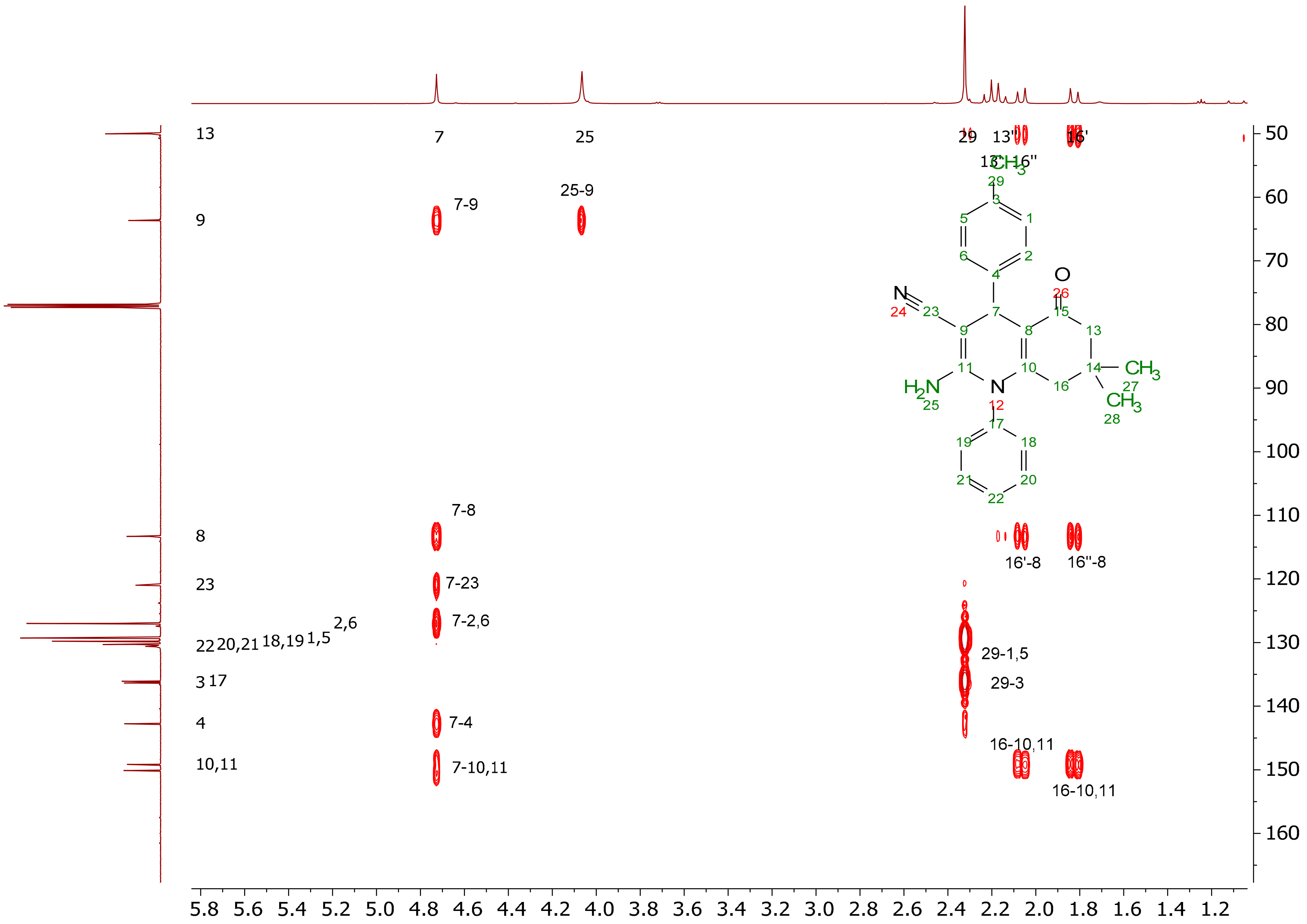

Figure S9 - Upfield region of HMBC spectrum of 5b

1.3. Product 5c: 2-amino-4-(4-chlorophenyl)-7,8-dimethyl-5-oxo-1-phenyl-1,4,5,6,7,8-hexahydroquinoline-3-carbonitrile

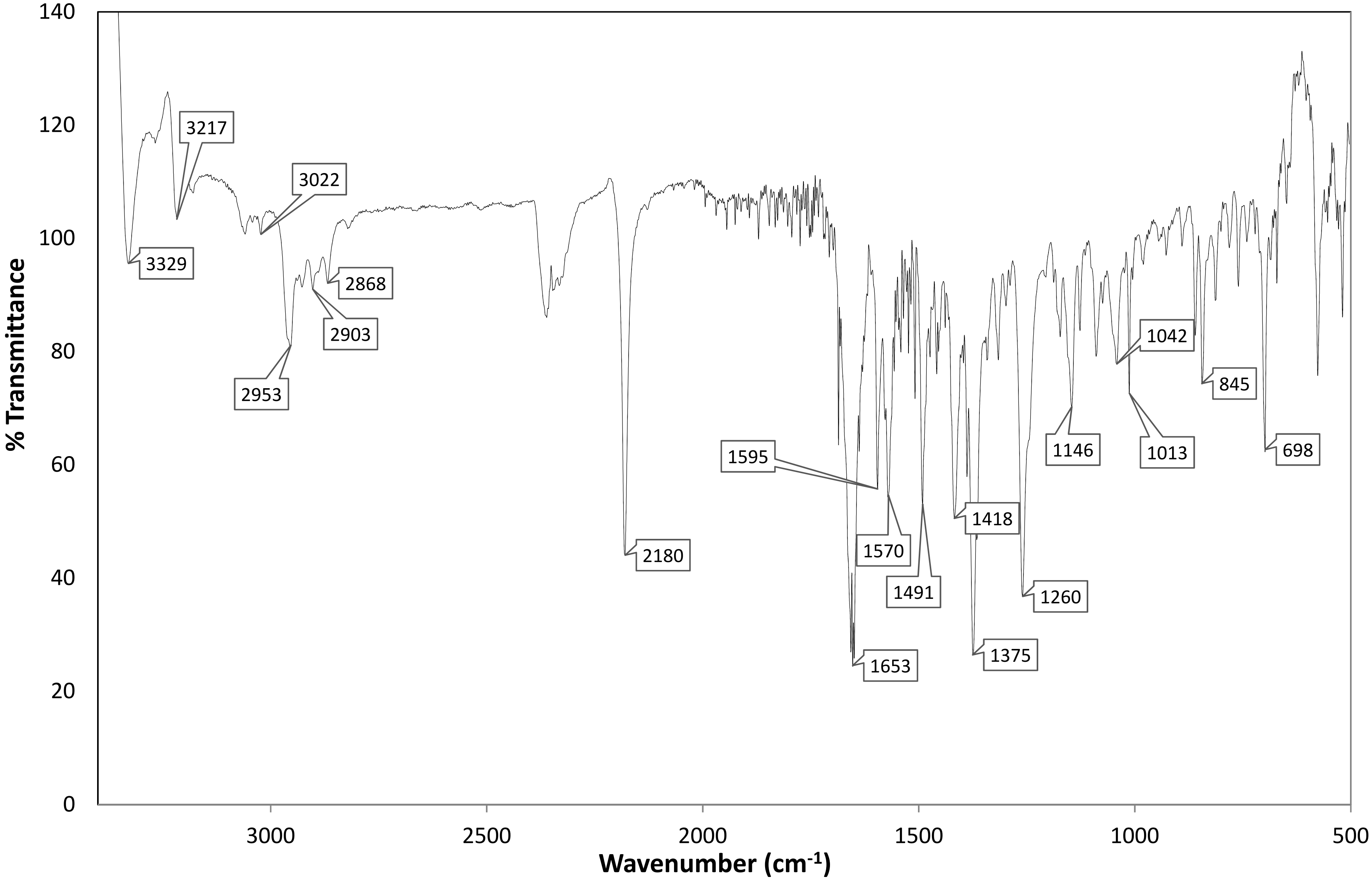

Figure S10 - IR spectrum of 5c

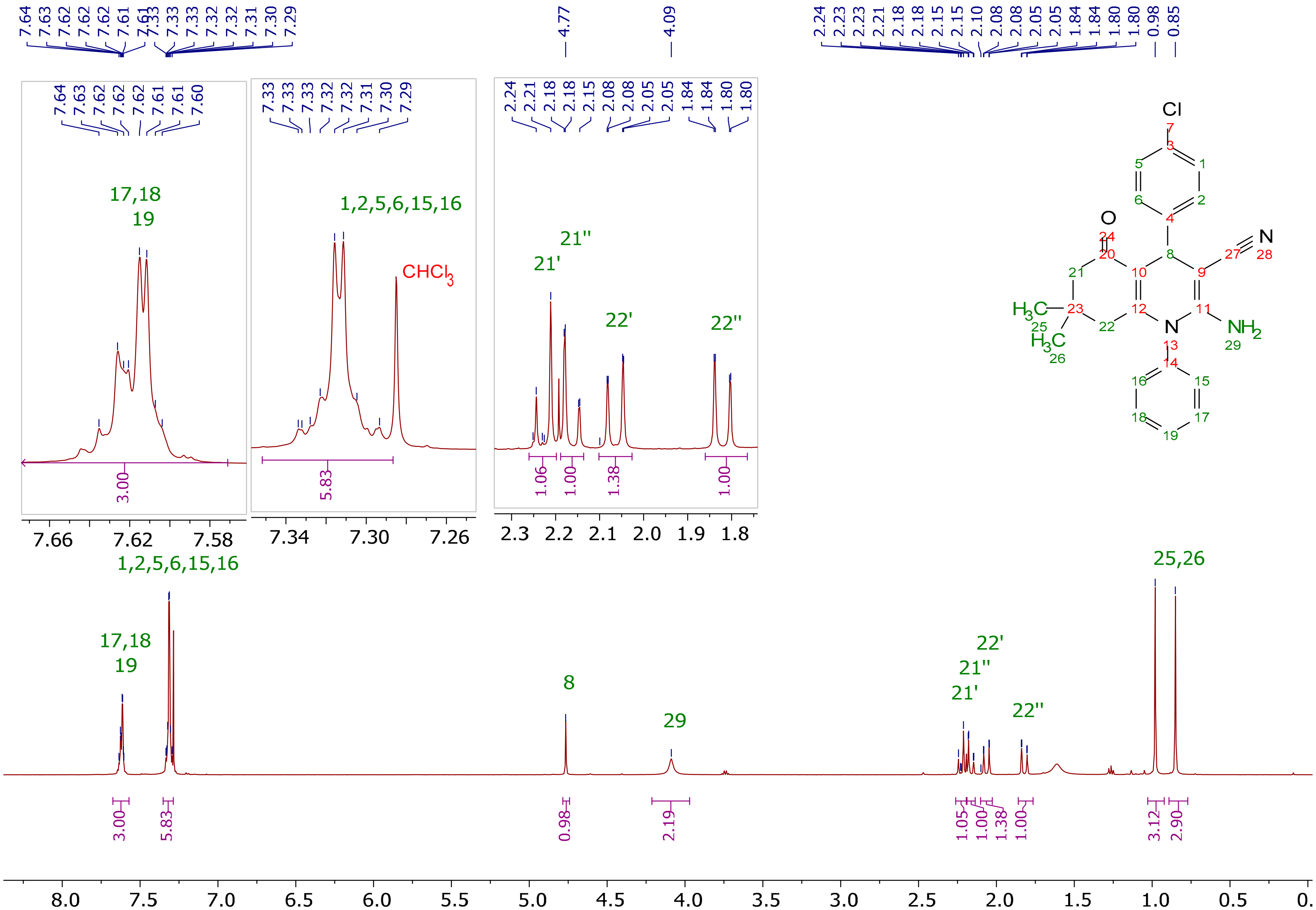

Figure S11 - ^{1}H NMR spectrum of 5c

1.4. Product 5d: 2-amino-4-(2,4-dichlorophenyl)-7,8-dimethyl-5-oxo-1-phenyl-1,4,5,6,7,8-hexahydroquinoline-3-carbonitrile

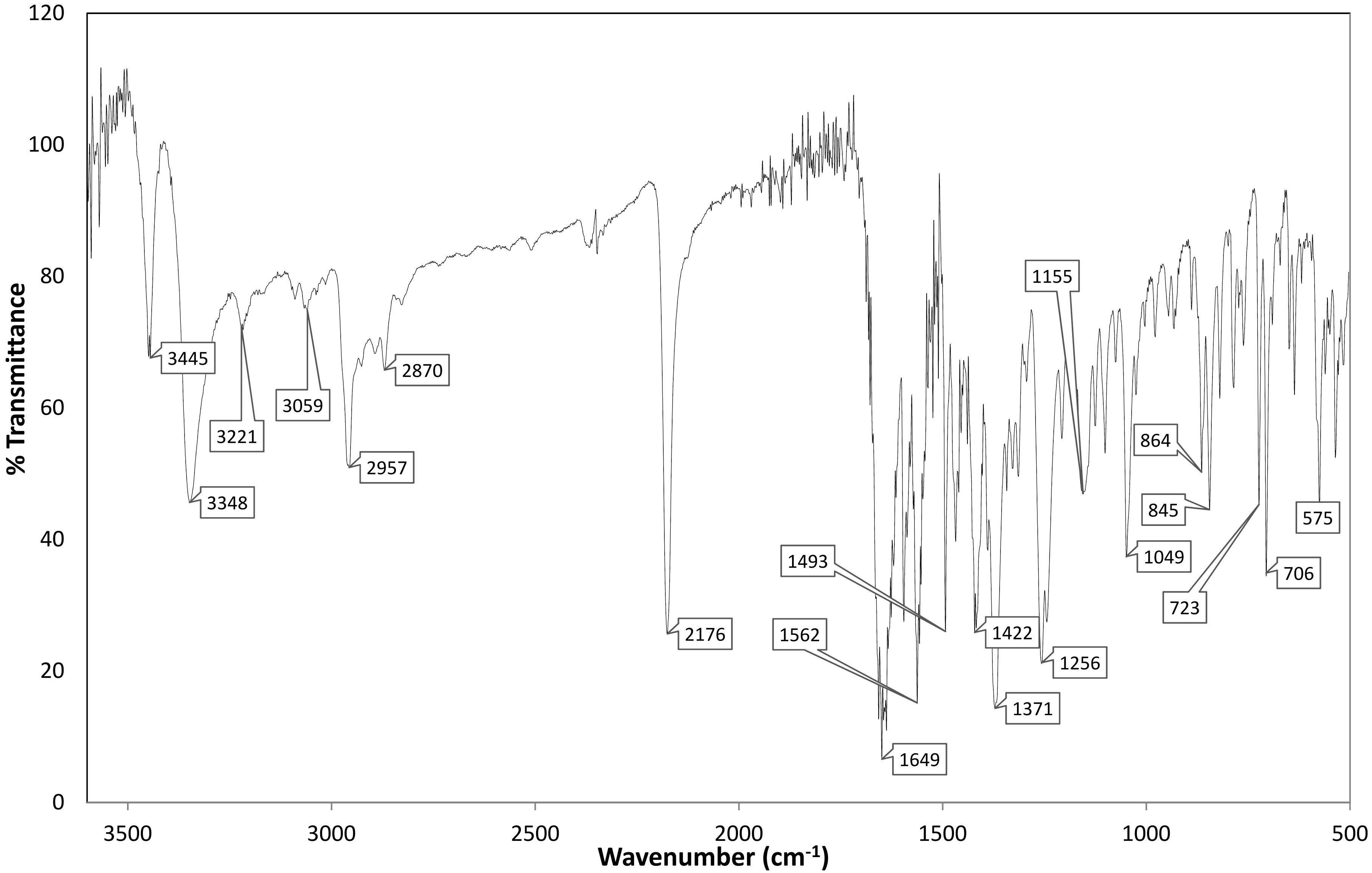

Figure S12 - IR spectrum of 5d

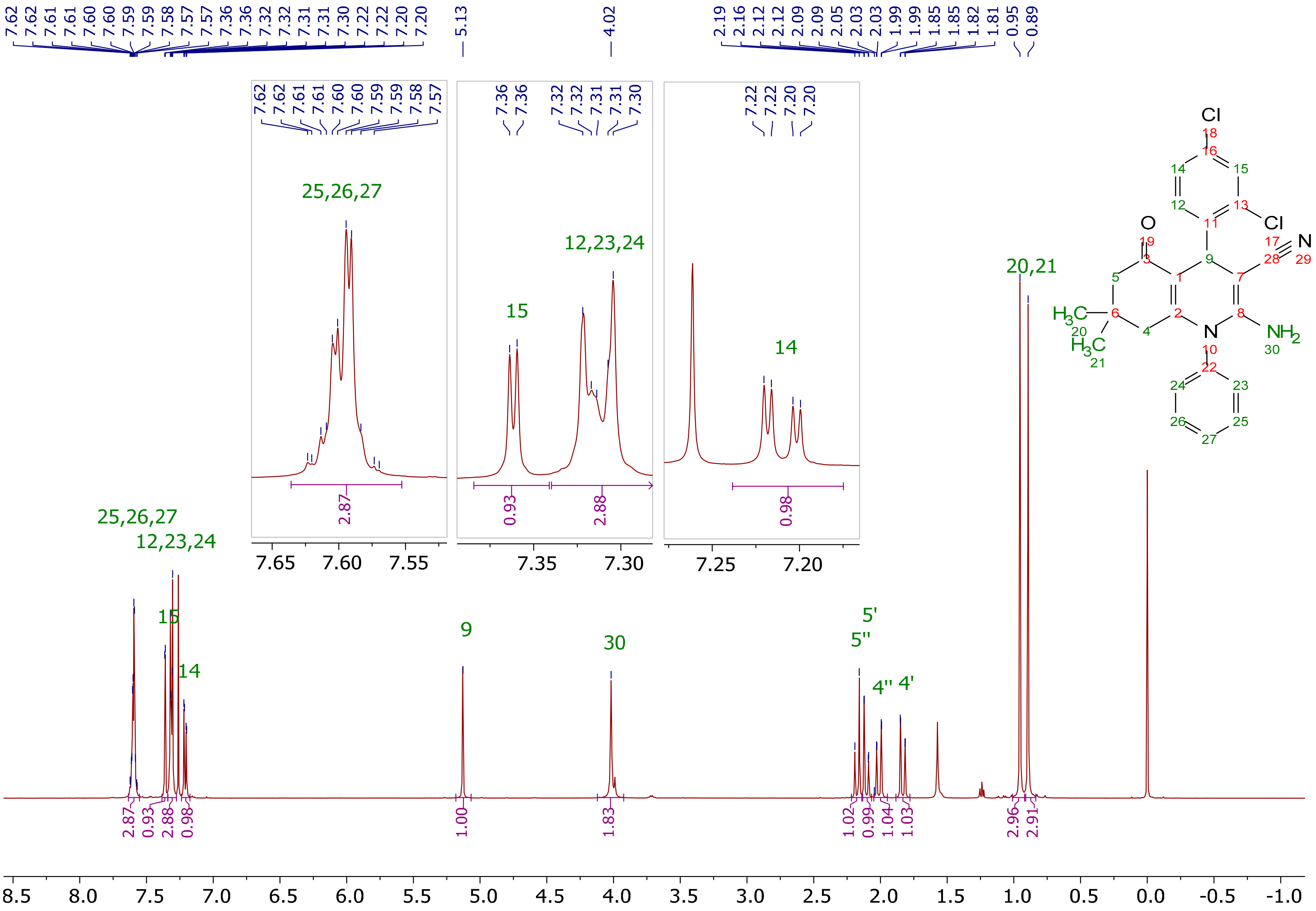

Figure S13 - ^{1}H NMR spectrum of 5d

1.5. Product 5e: 2-amino-4-(4-fluorophenyl)-7,8-dimethyl-5-oxo-1-phenyl-1,4,5,6,7,8-hexahydroquinoline-3-carbonitrile

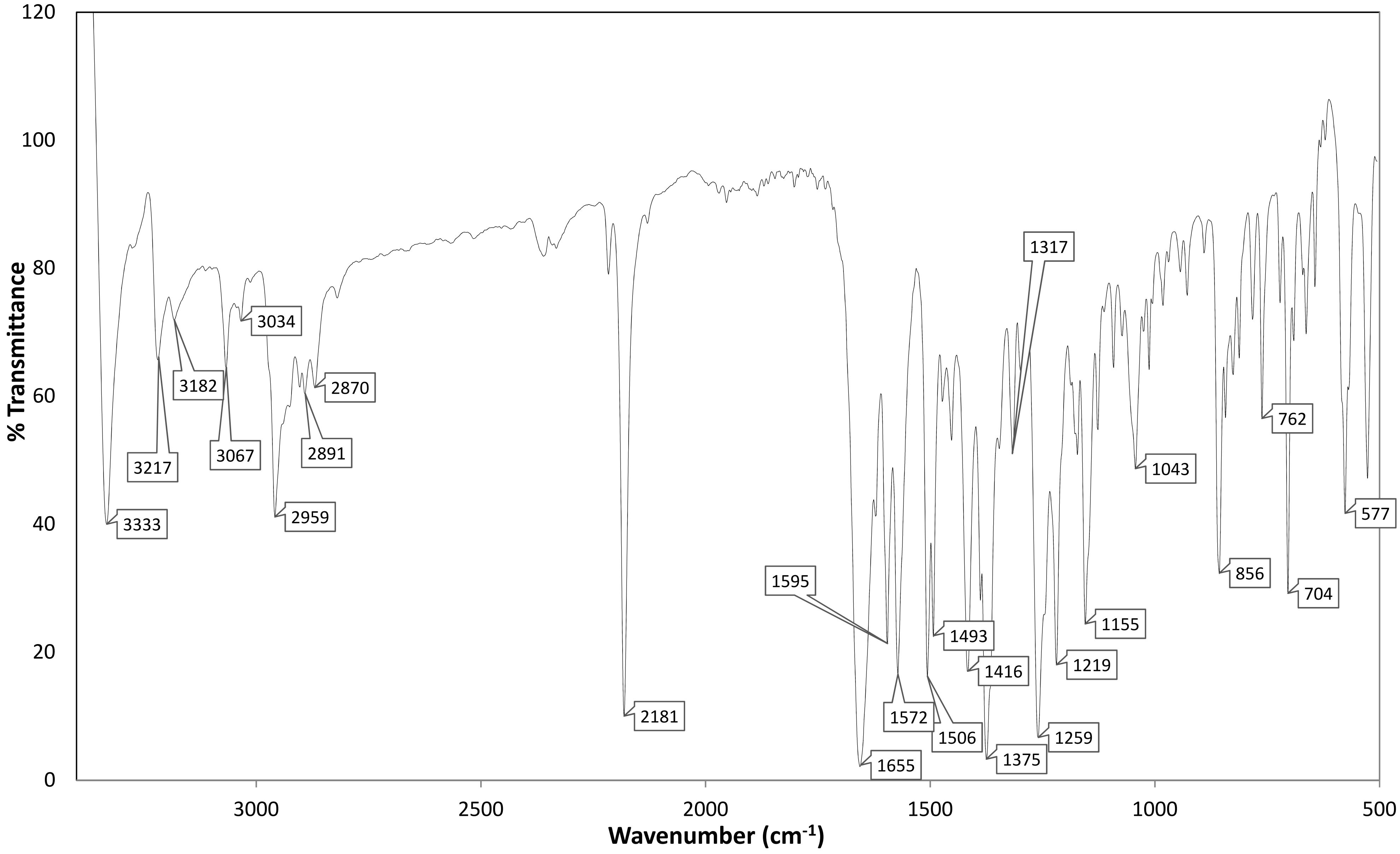

Figure S14 - IR spectrum of 5e

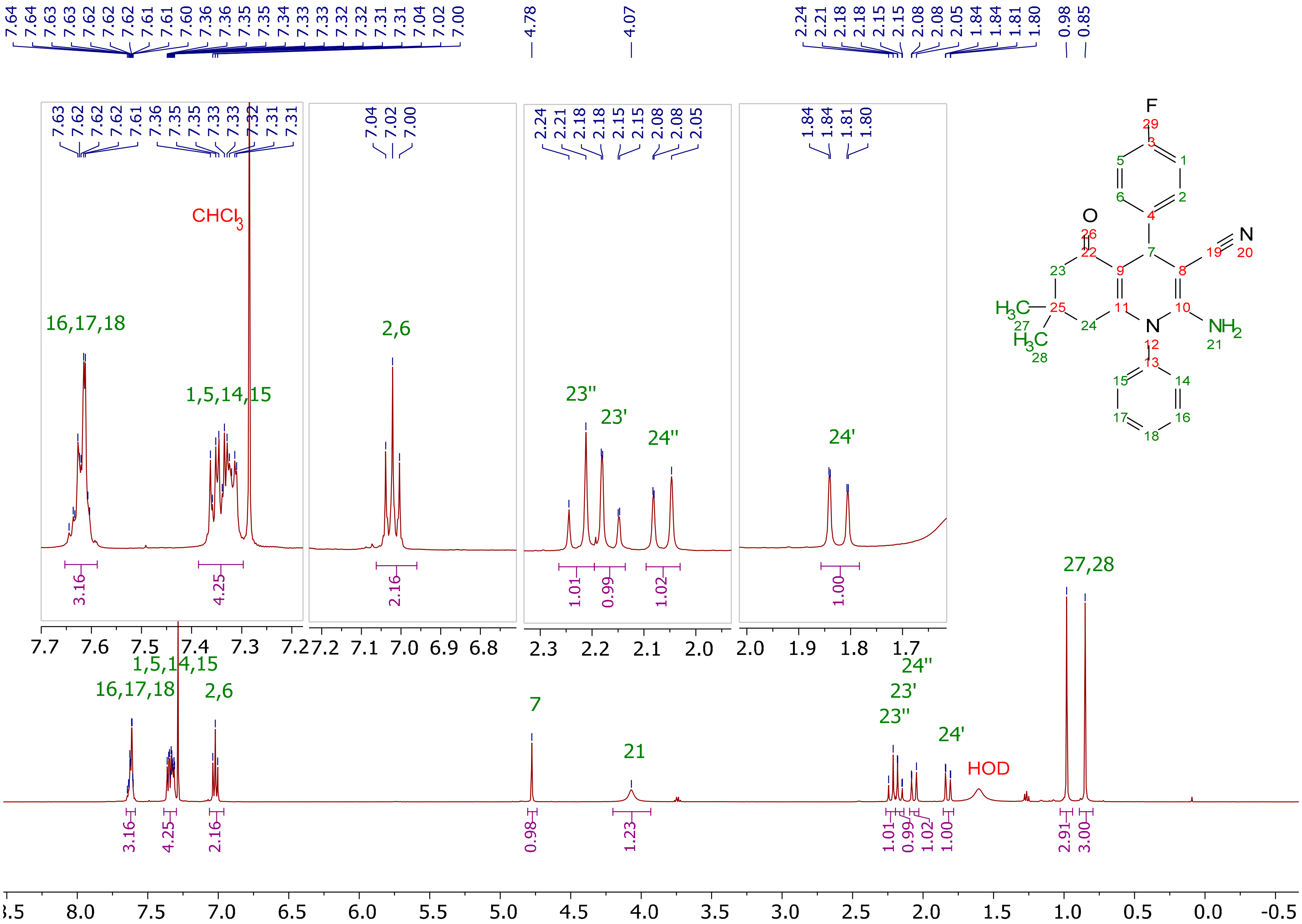

Figure S15 - ^{1}H NMR spectrum of 5e

1.6. Product 5f: 2-amino-7,8-dimethyl-5-oxo-1-phenyl-4-thiophen-2-yl-1,4,5,6,7,8-hexahydroquinoline-3-carbonitrile

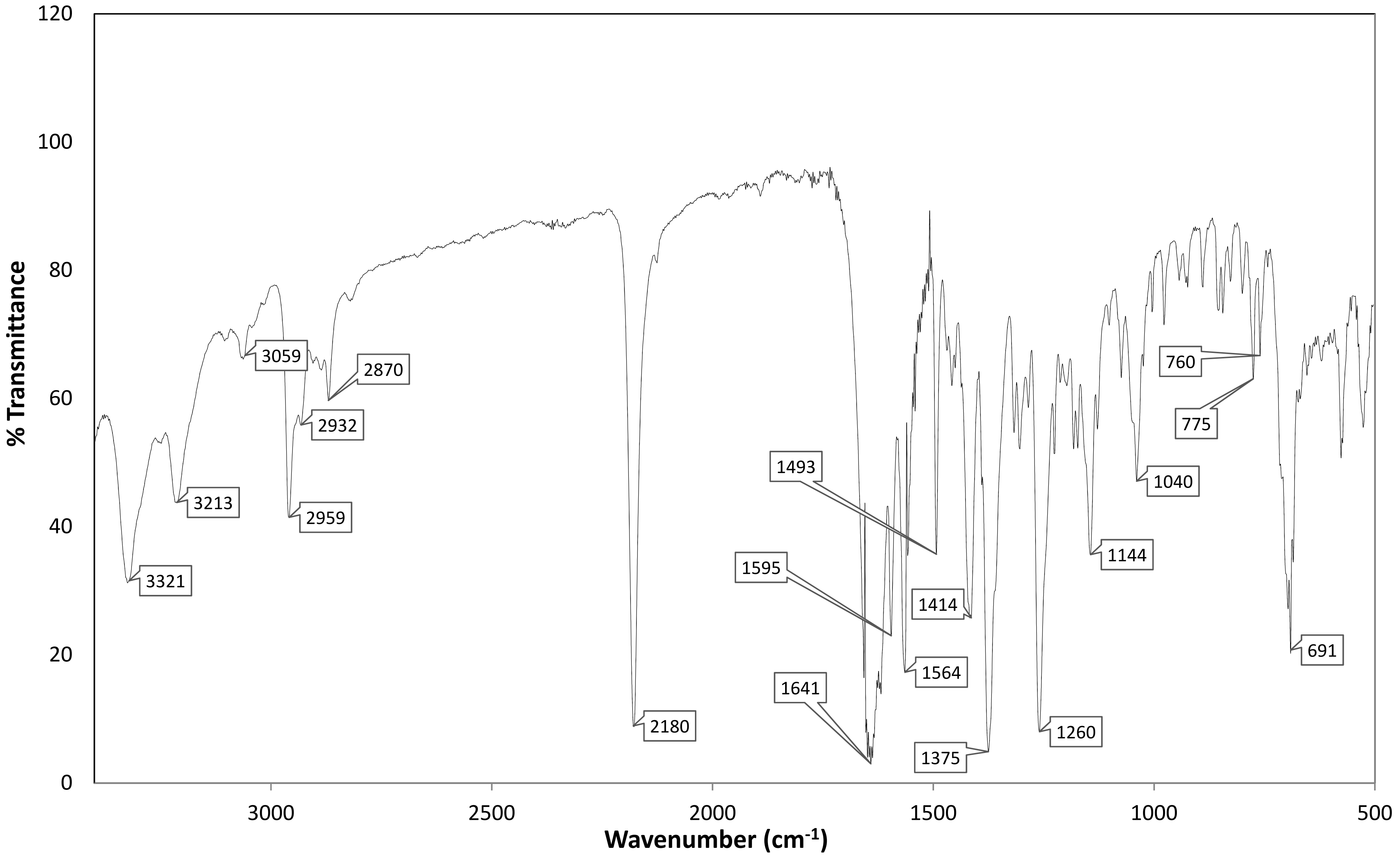

Figure S16 - IR spectrum of 5f

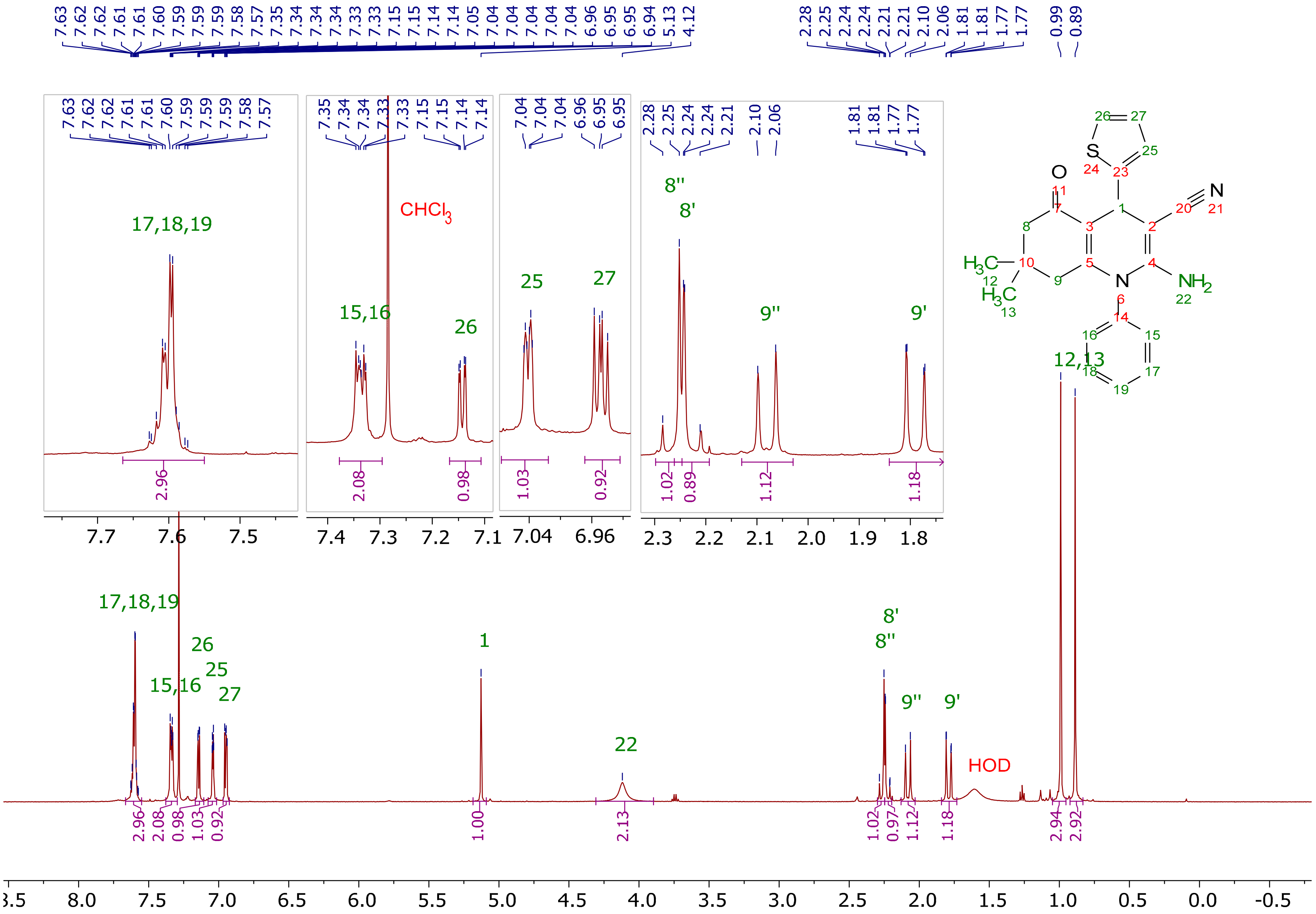

Figure S17 - ^{1}H NMR spectrum of 5f

1.7. Product 5g: 2-amino-7,8-dimethyl-5-oxo-1-phenyl-4-(3-nitrophenyl)-1,4,5,6,7,8-hexahydroquinoline-3-carbonitrile

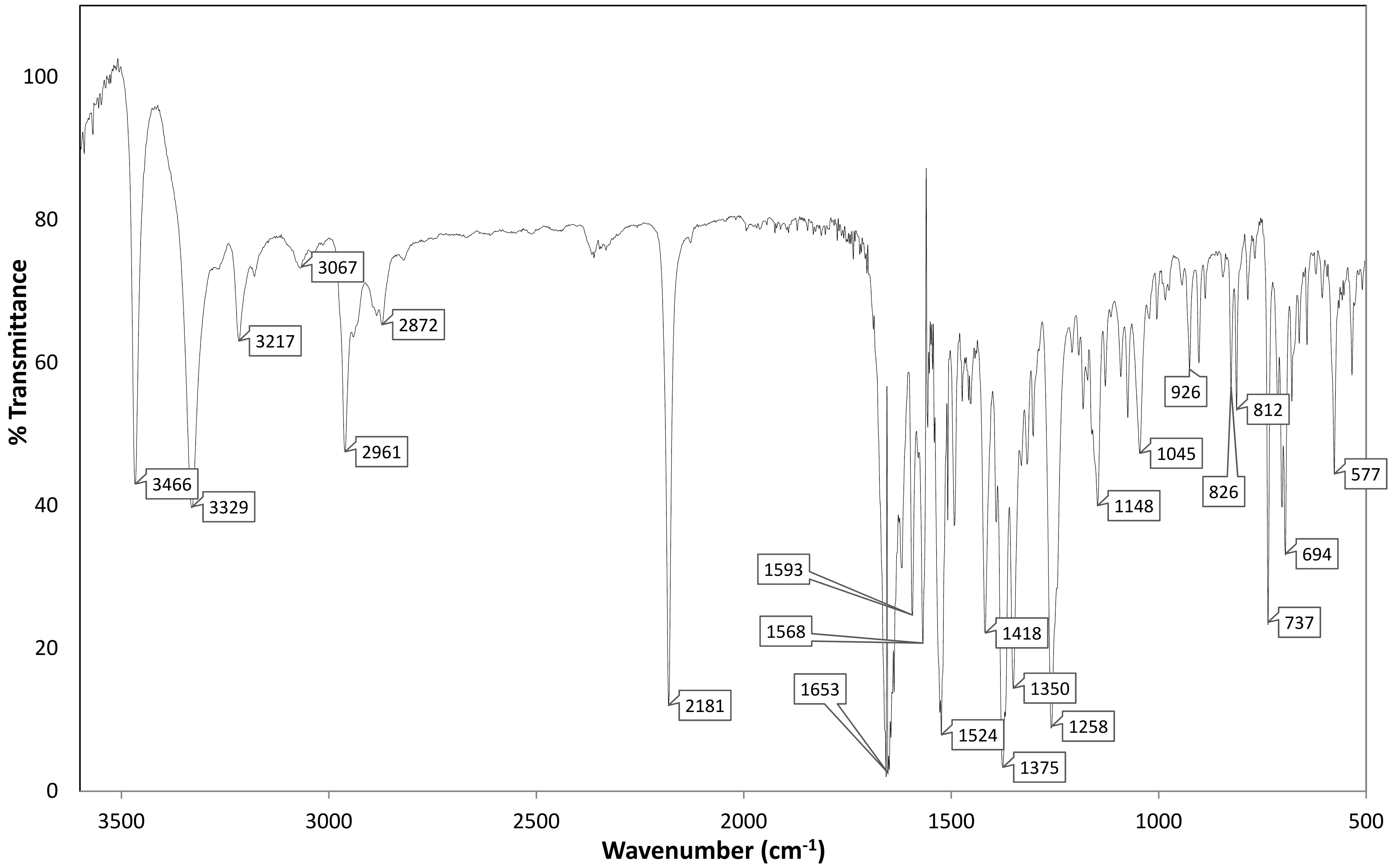

Figure S18 - IR spectrum of 5g

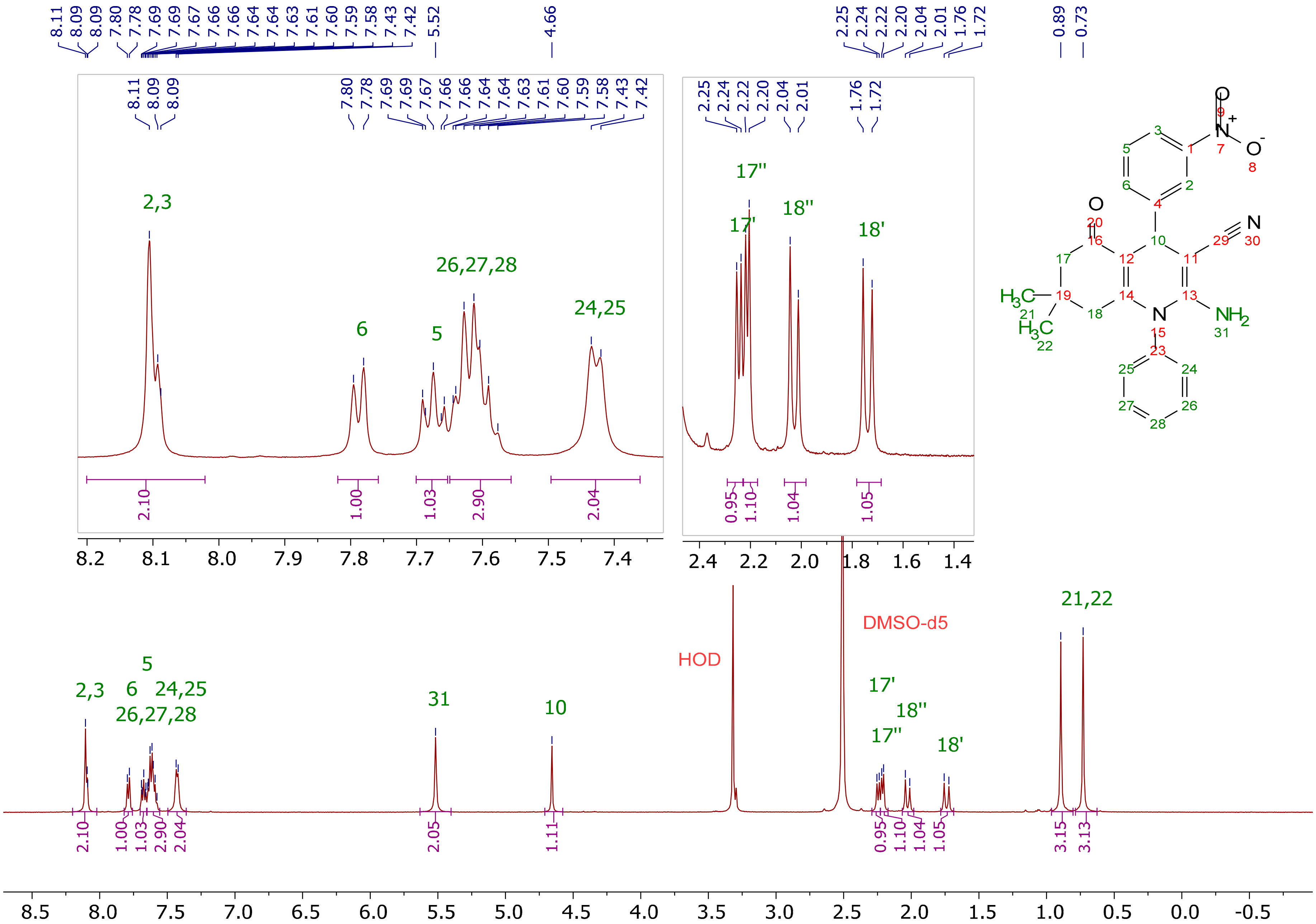

Figure S19 - ¹H NMR spectrum of 5g

1.8. Product 5h: 2-amino-4-(4-methoxyphenyl)-7,8-dimethyl-5-oxo-1-phenyl-1,4,5,6,7,8-hexahydroquinoline-3-carbonitrile

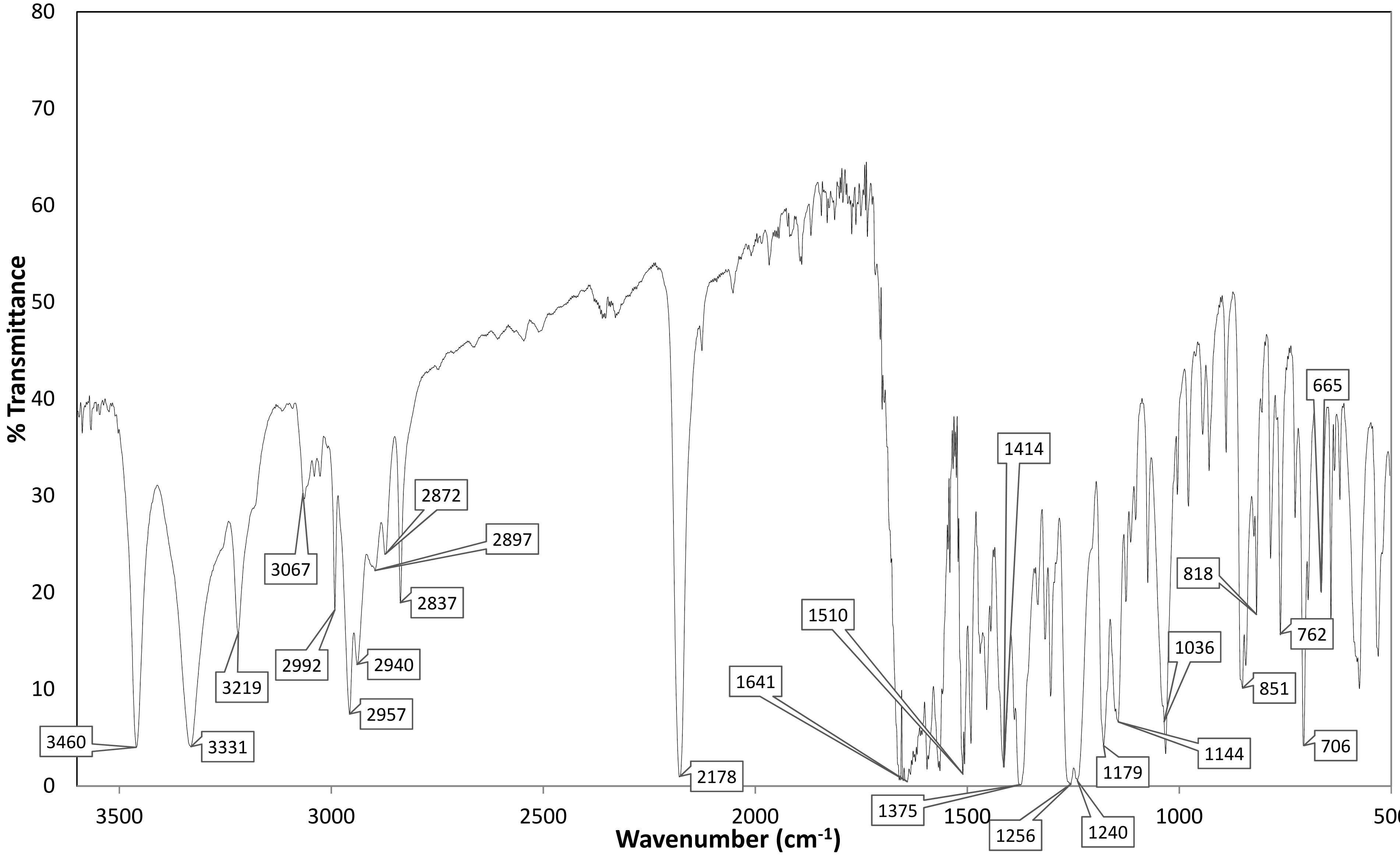

Figure S20 - IR spectrum of 5h

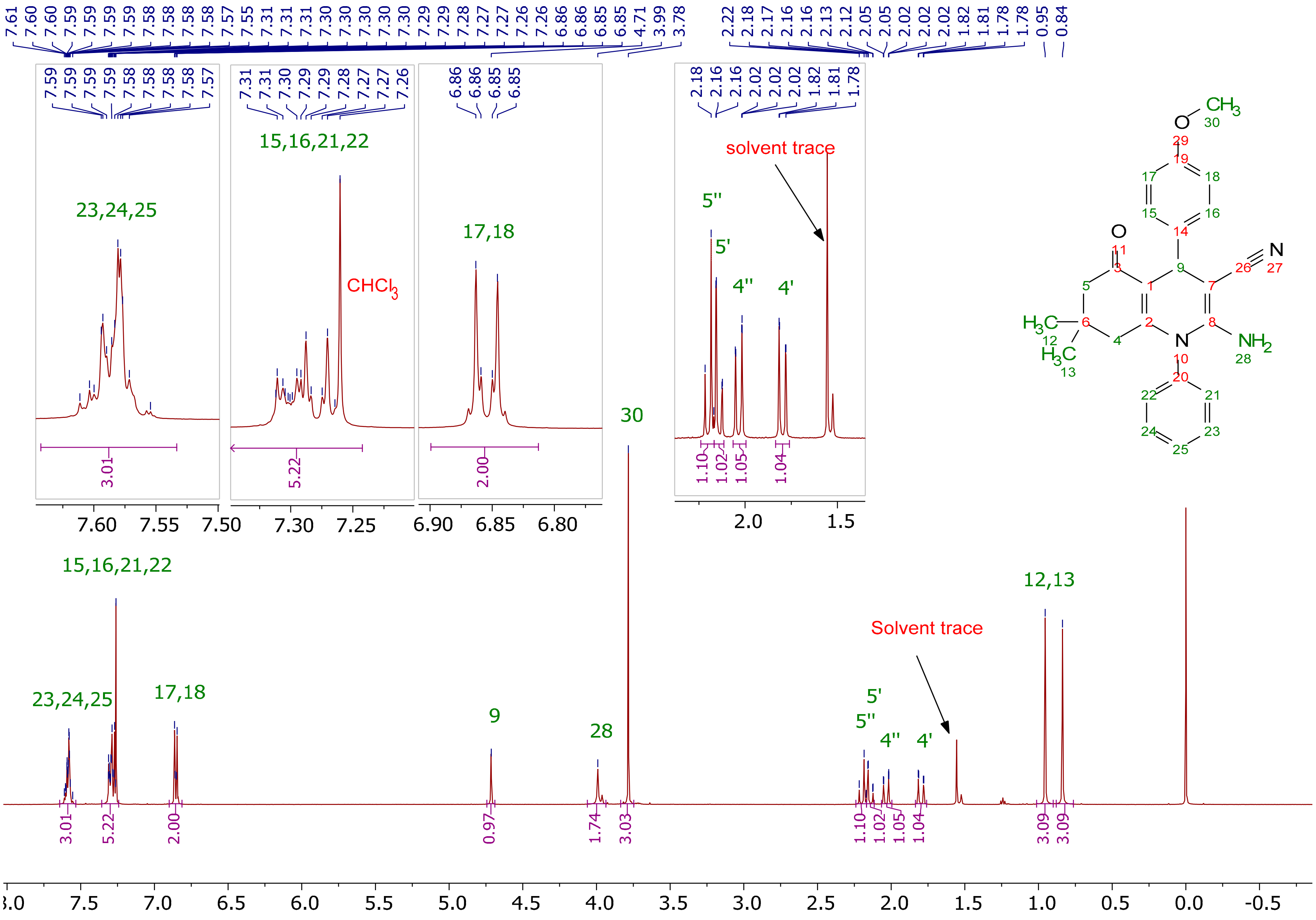

Figure S21 - ^{1}H NMR spectrum of 5h

1.9. Product 5i: Methyl 2-amino-4-(2,4-dichlorophenyl)-7,8-dimethyl-5-oxo-1-phenyl-1,4,5,6,7,8-hexahydroquinoline-3-carboxylate

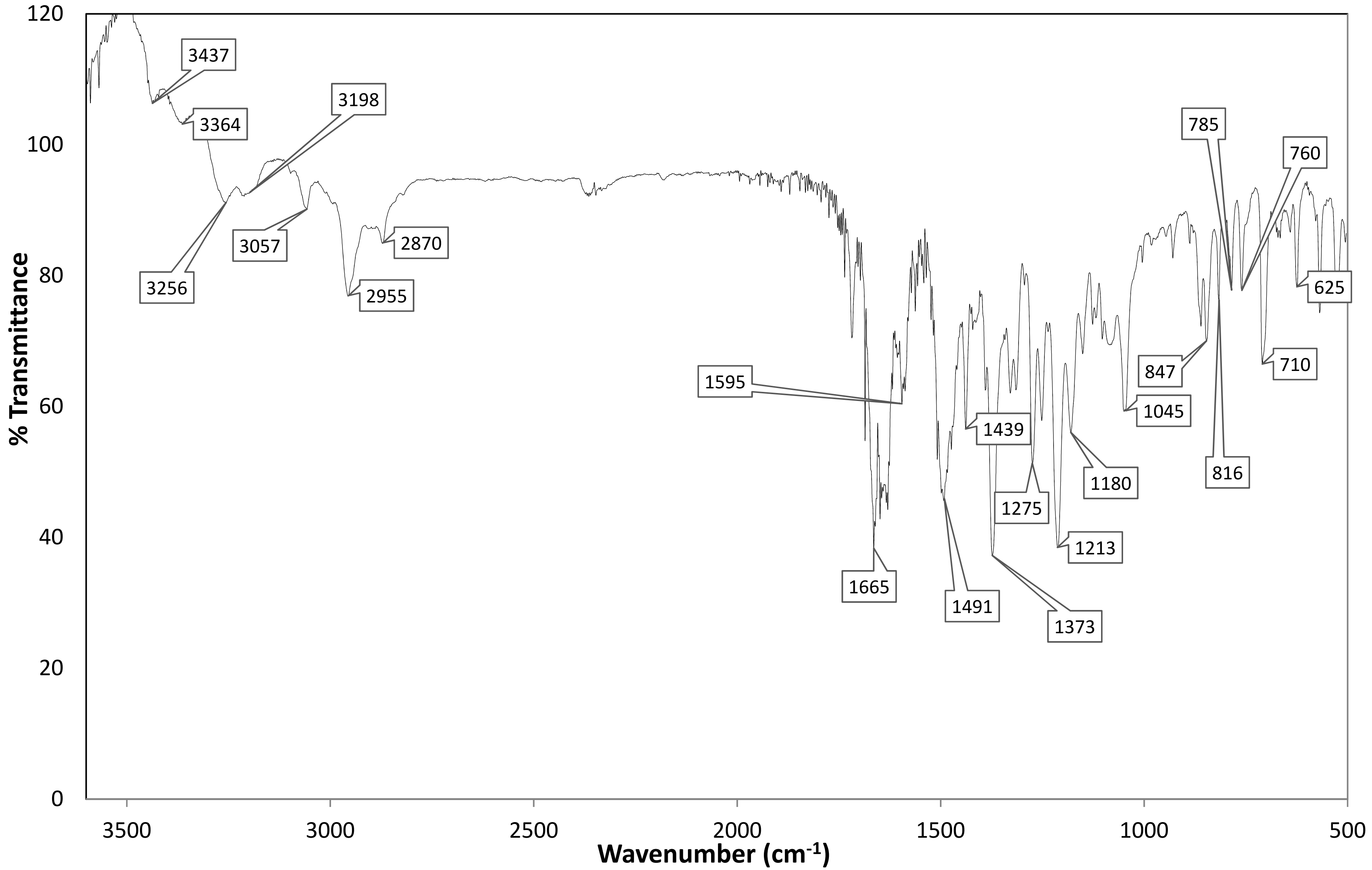

Figure S22 - IR spectrum of 5i

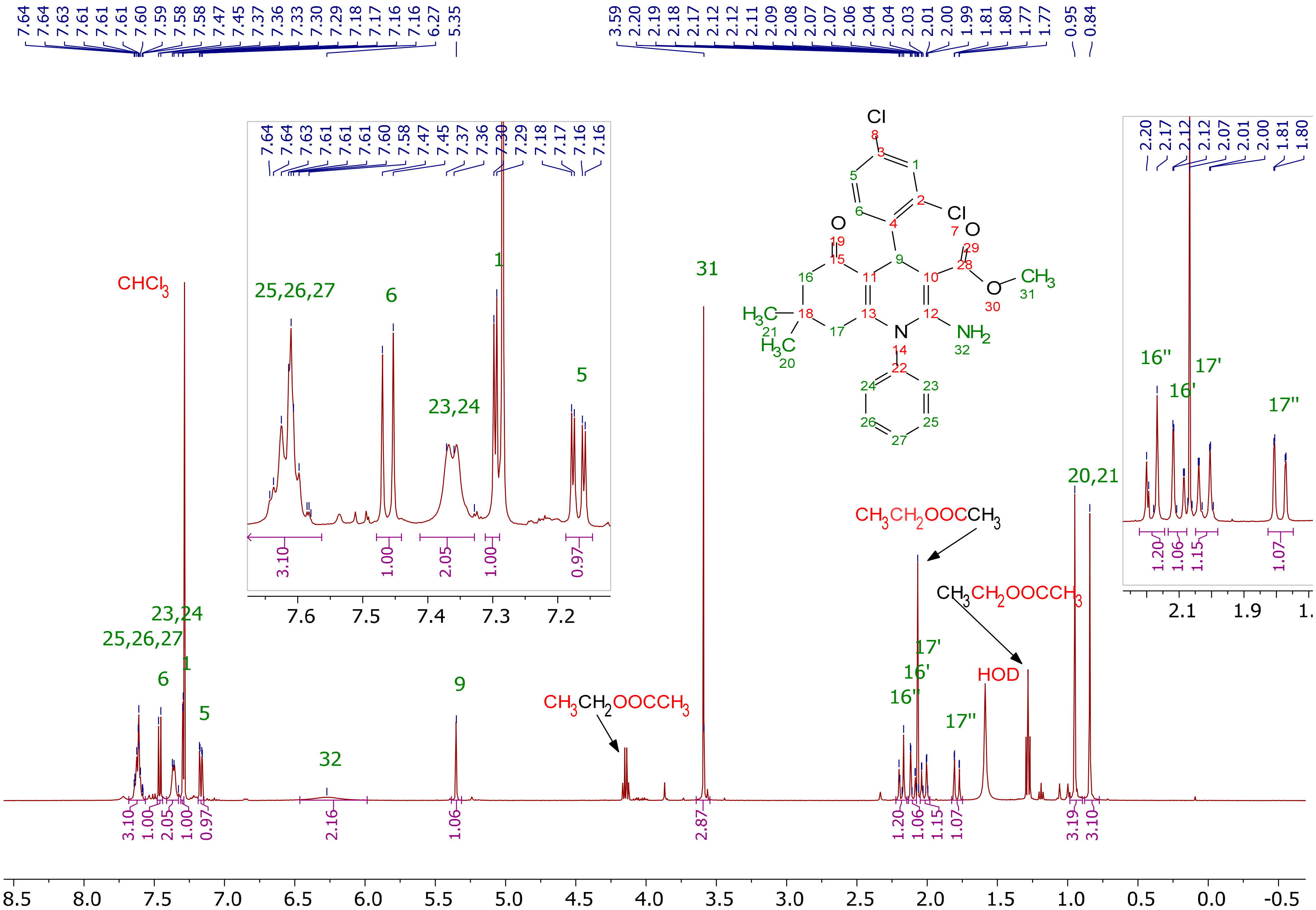

Figure S23 - ^{1}H NMR spectrum of 5i

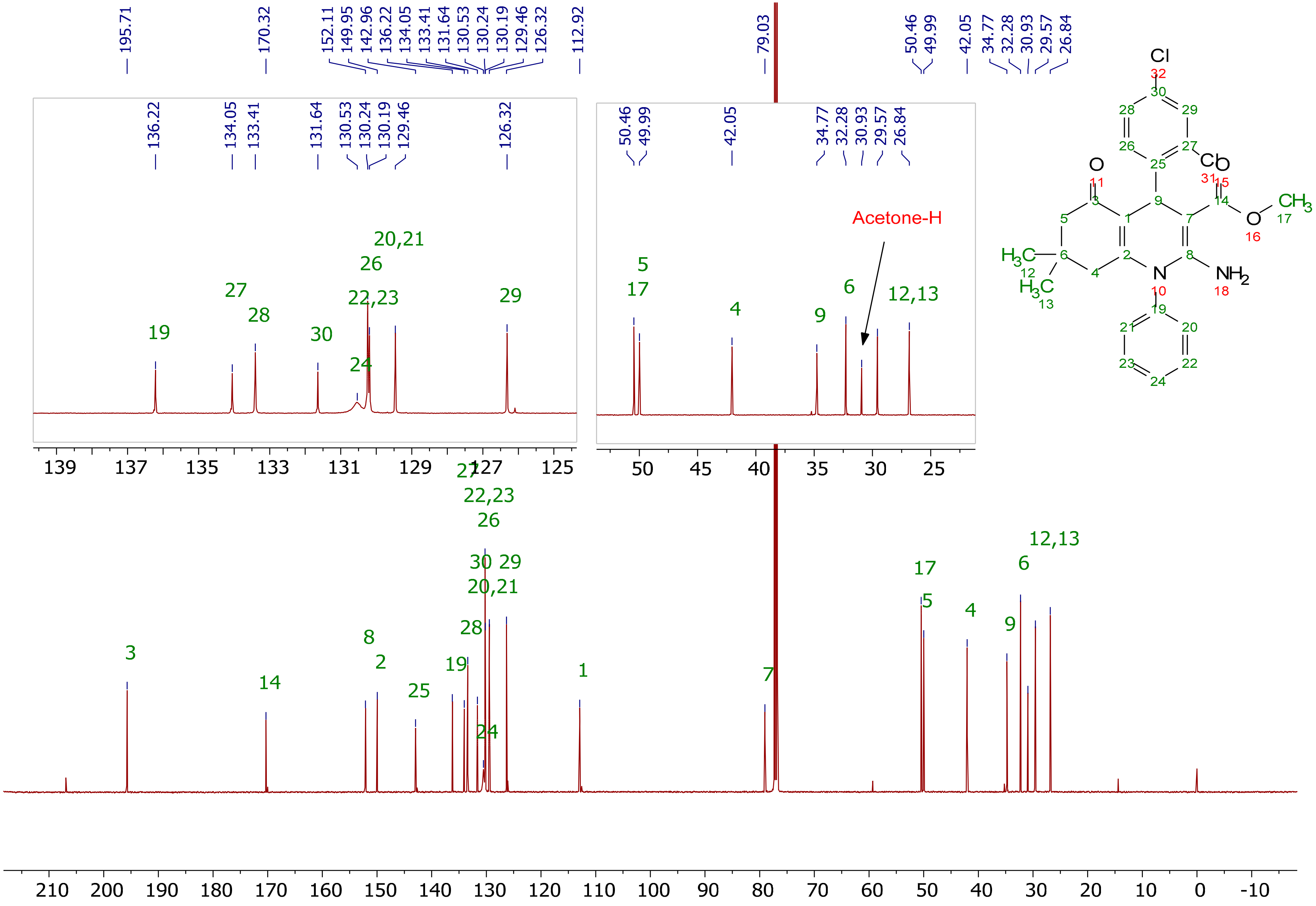

Figure S24 – ^{13}C NMR spectrum of 5i

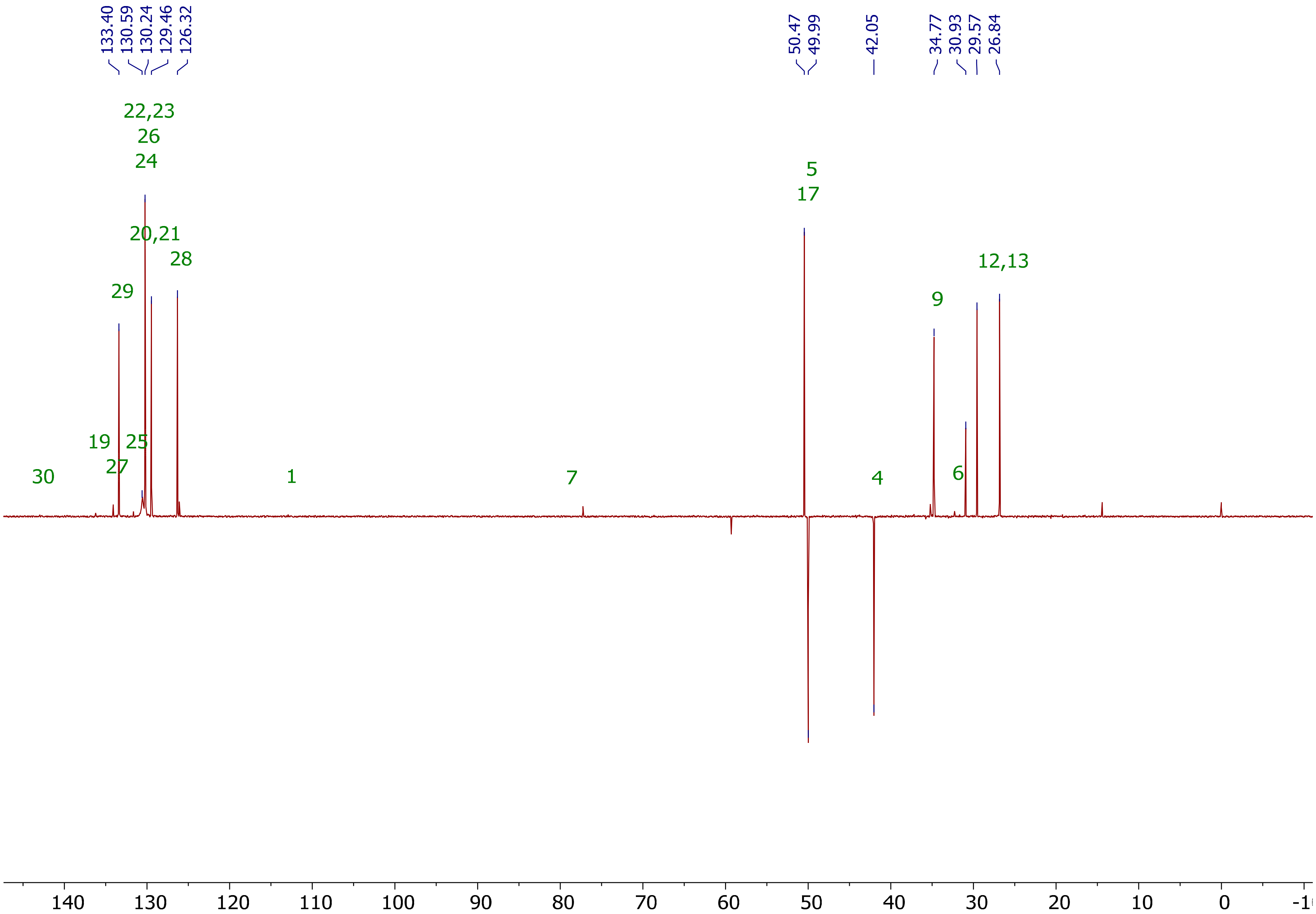

Figure S25 - DEPT spectrum of 5i

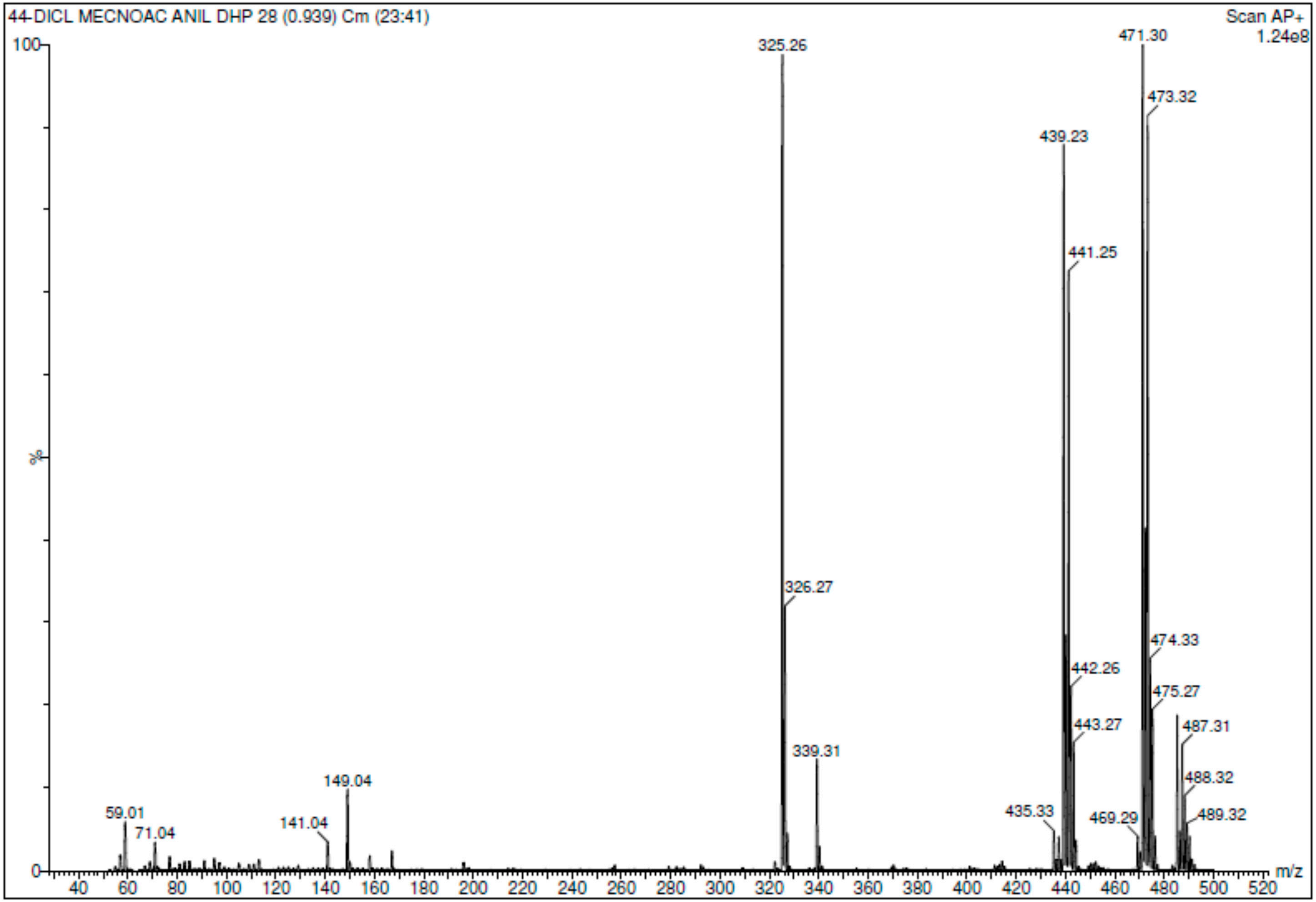

Figure S26 - MS spectrum of 29i

Table S2 - Fragmentation position for peaks in MS spectrum of 29i

m/z	Fragmentation position and structure
**471.30 – 475.27**	$[M+H]^+$ Multiple isotopes
**439.23 – 443.27**	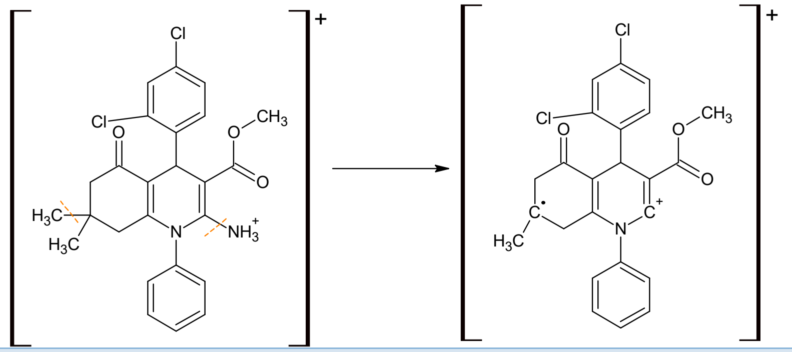
**325.26**	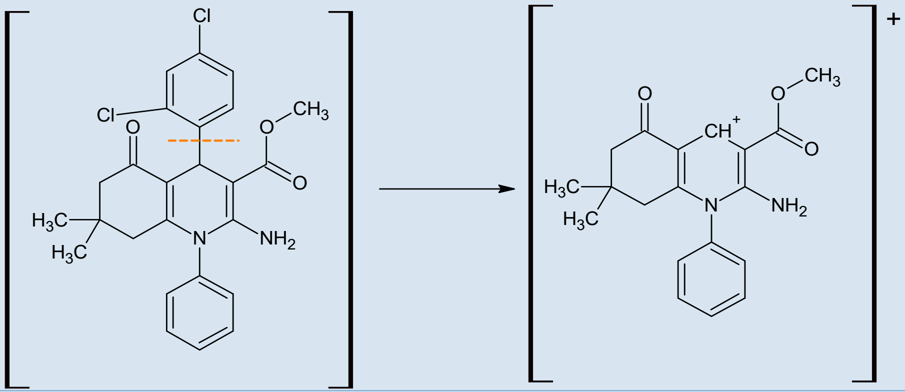
**326.27**	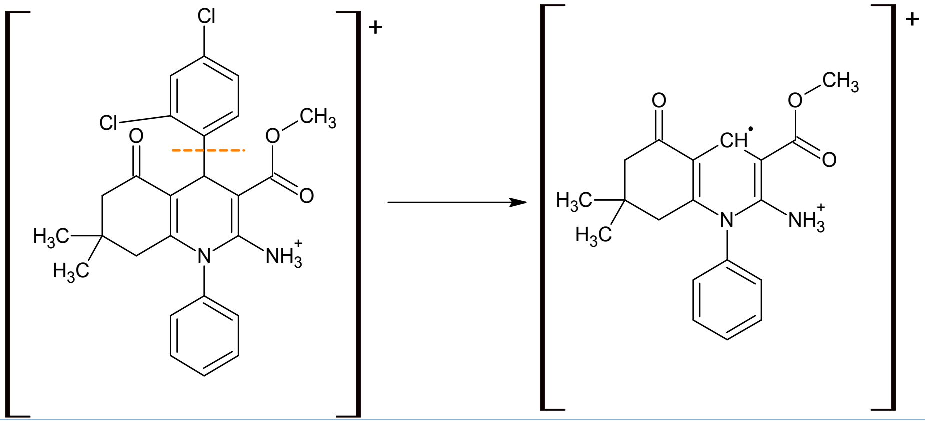

1.10. Product 5j: 2-amino-7,8-dimethyl-5-oxo-1-(3-methylphenyl)-4-(3-nitrophenyl)-1,4,5,6,7,8-hexahydroquinoline-3-carbonitrile

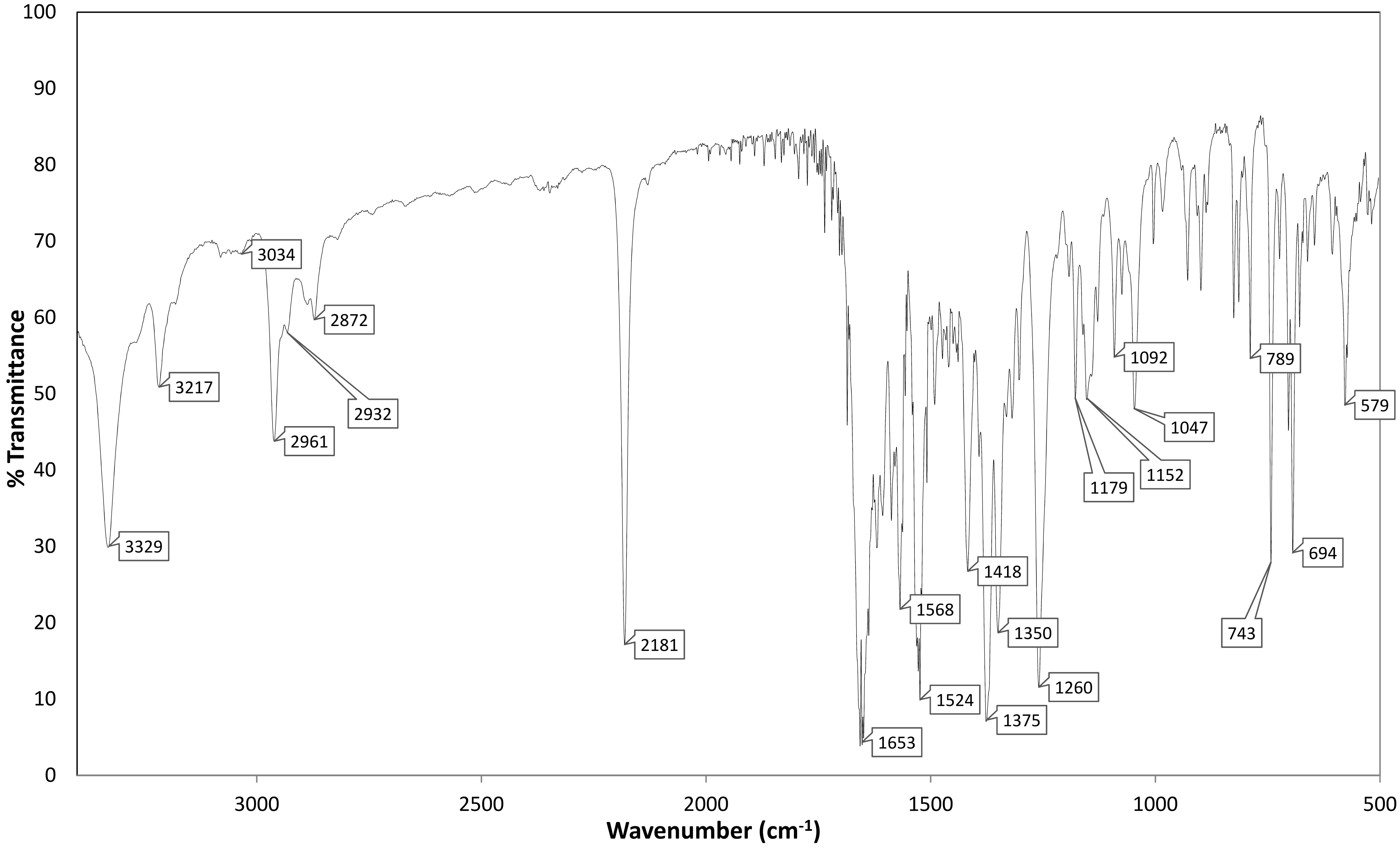

Figure S27 - IR spectrum of 5j

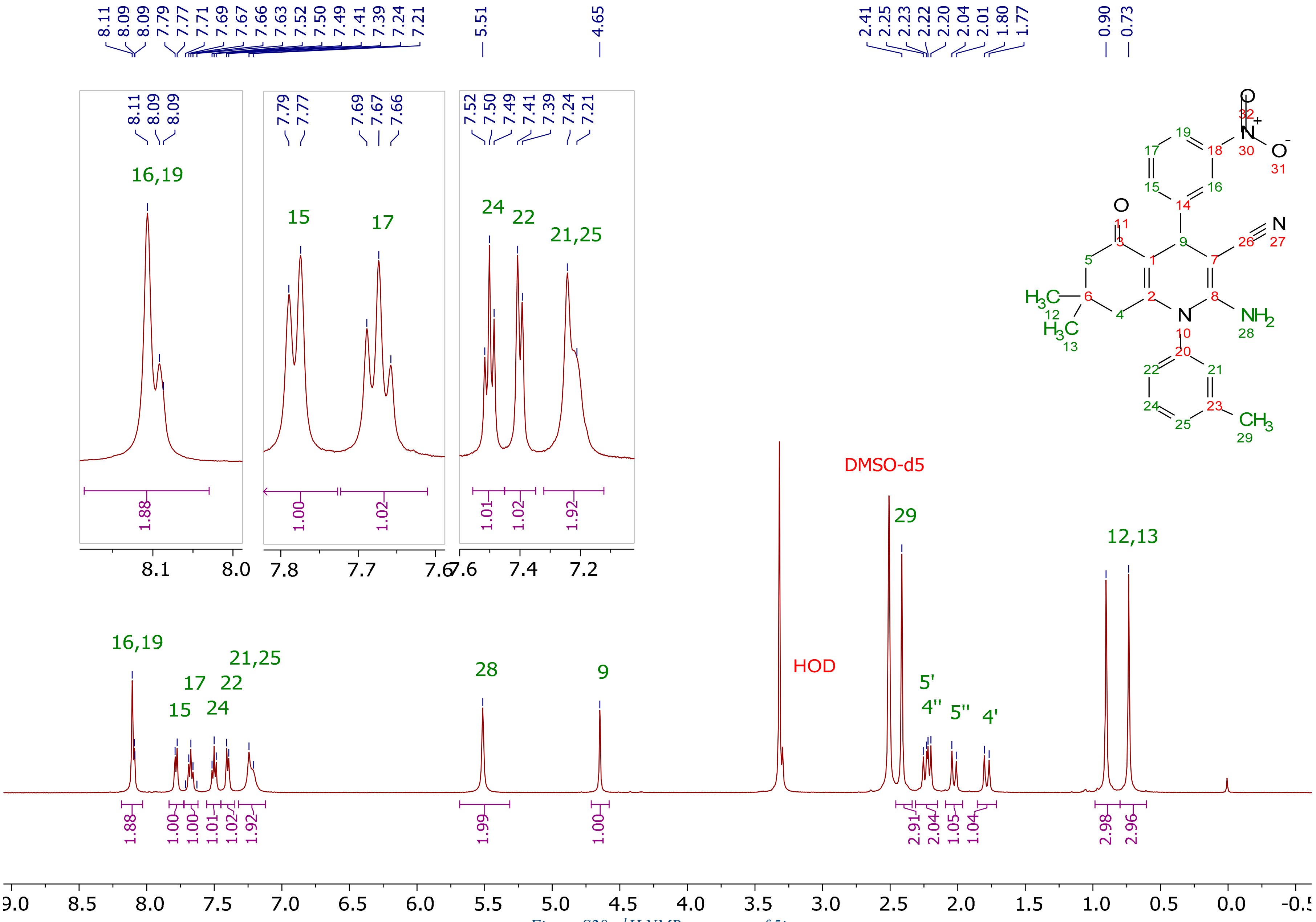

Figure S28 - ^{1}H NMR spectrum of 5j

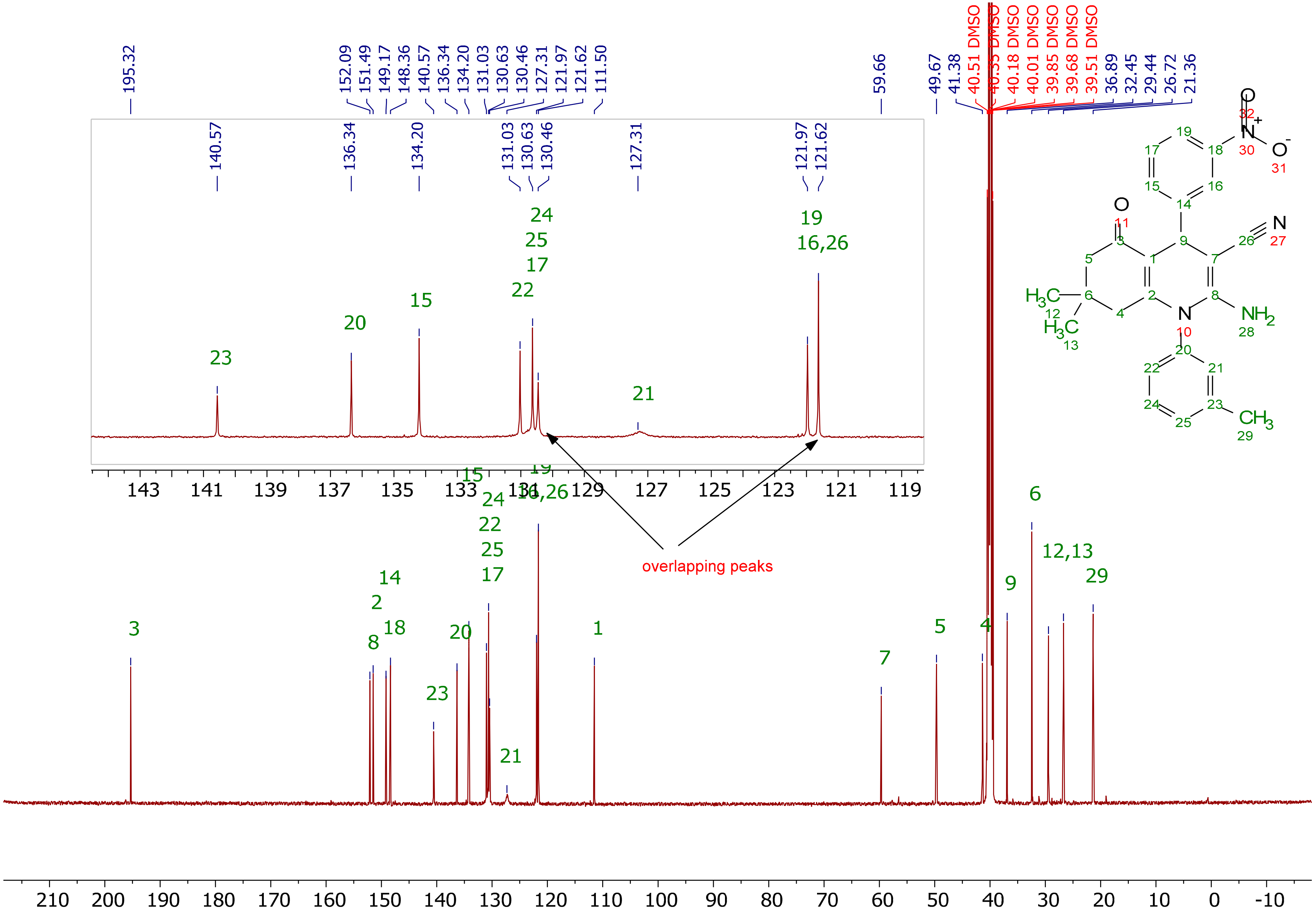

Figure S29 - ^{13}C NMR spectrum of 5j

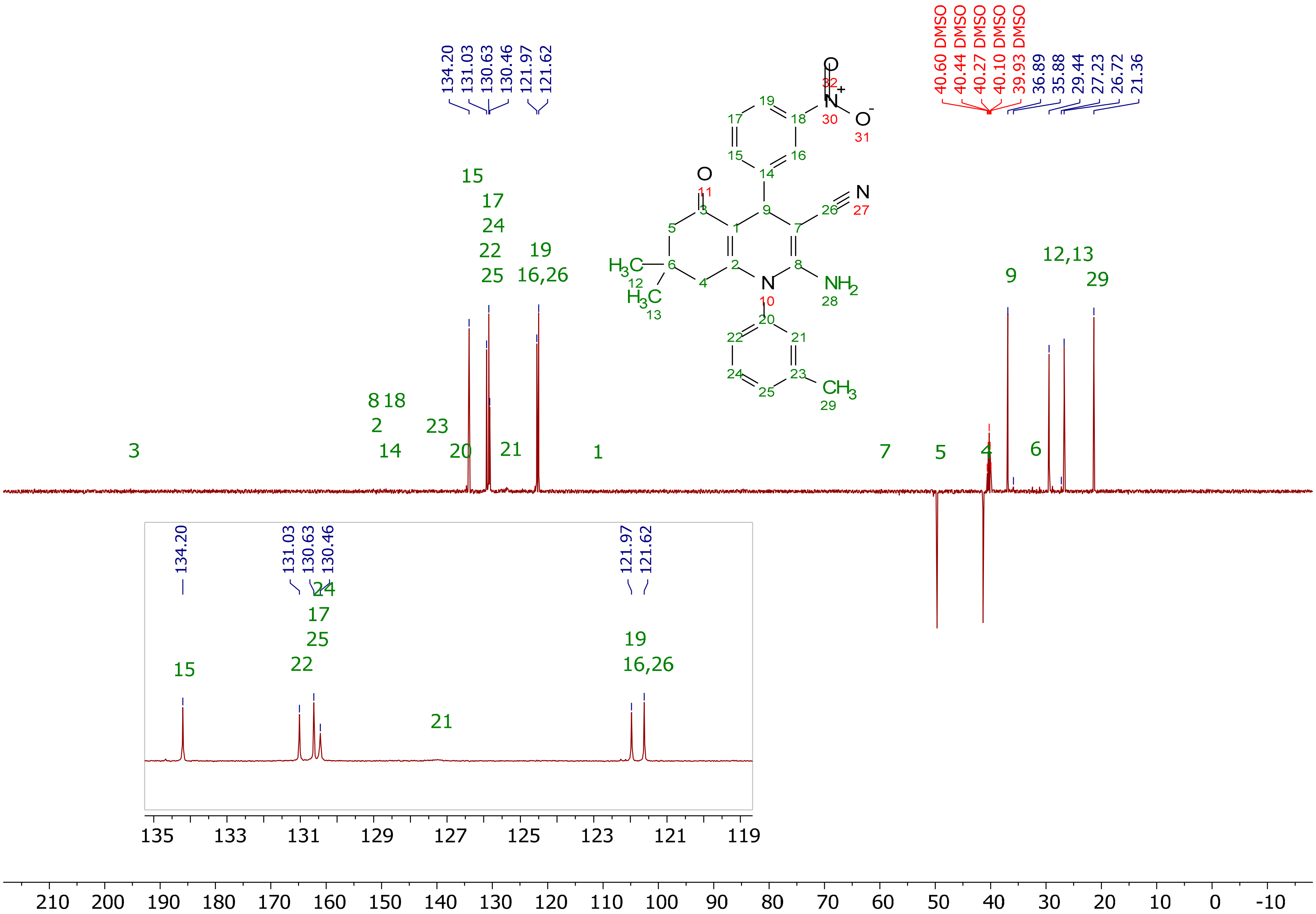

Figure S30 - DEPT spectrum of 5j

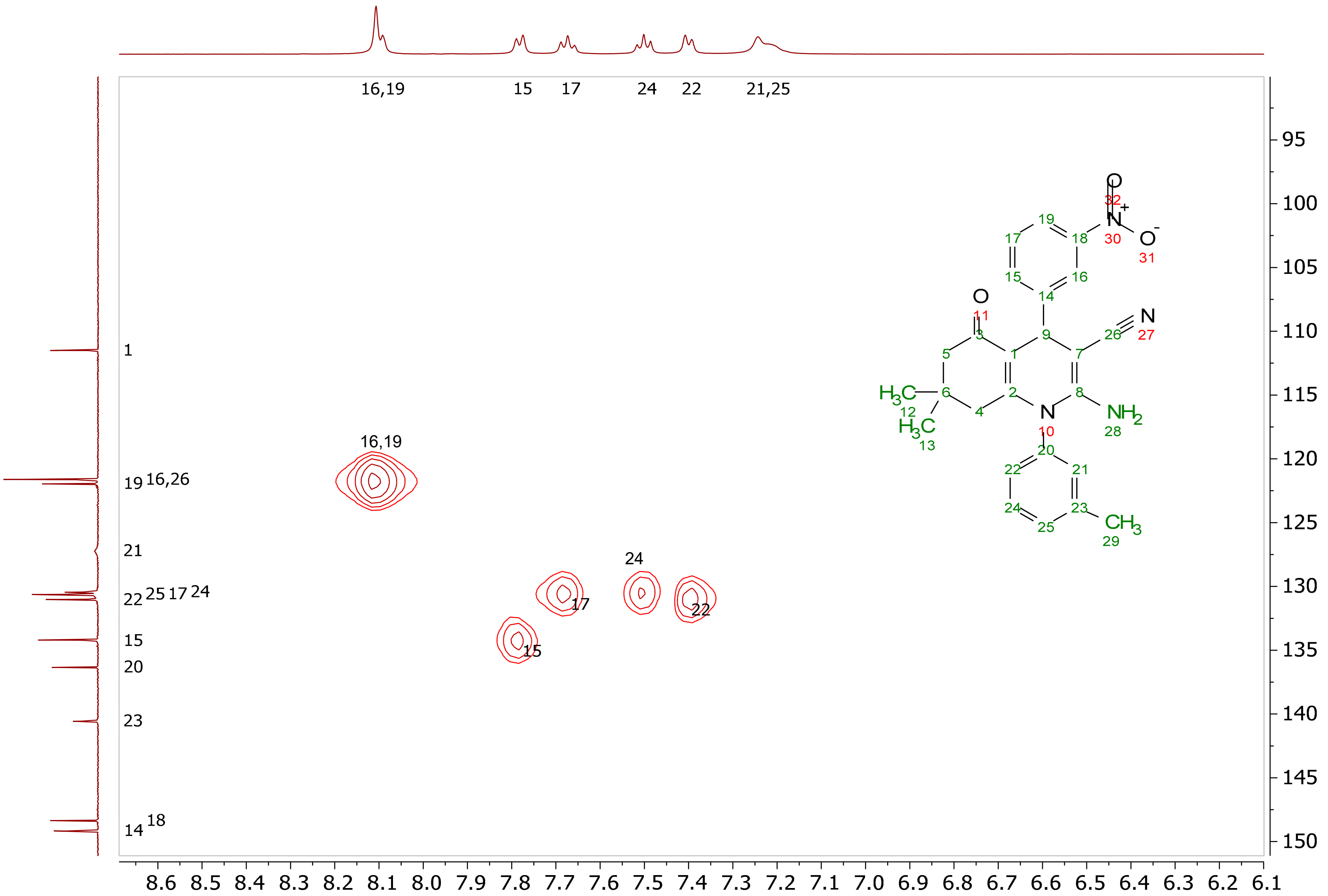

Figure S31 – Downfield region of HSQC NMR spectrum of 5j

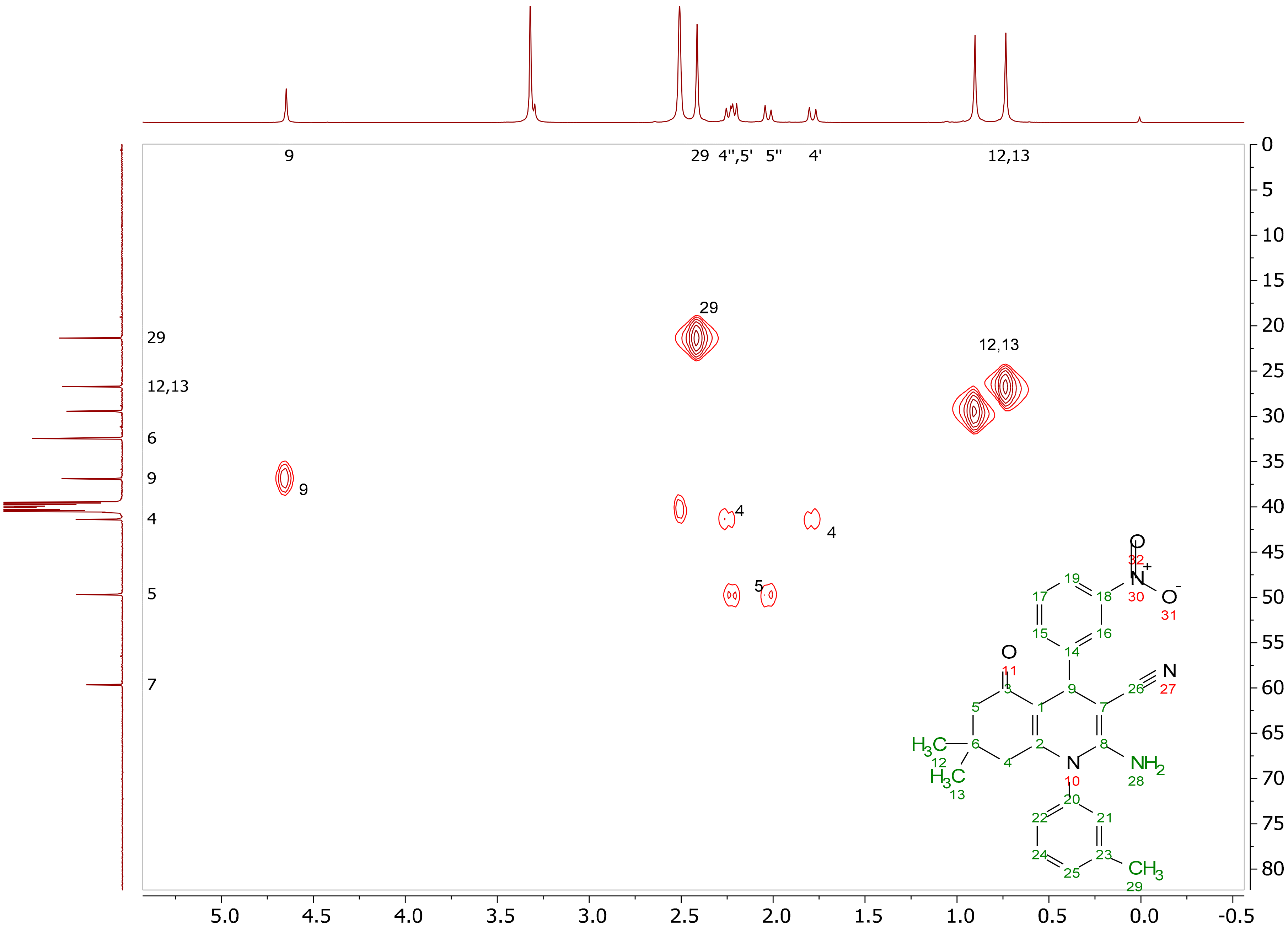

Figure S32 - Upfield region of HSQC spectrum of 5j

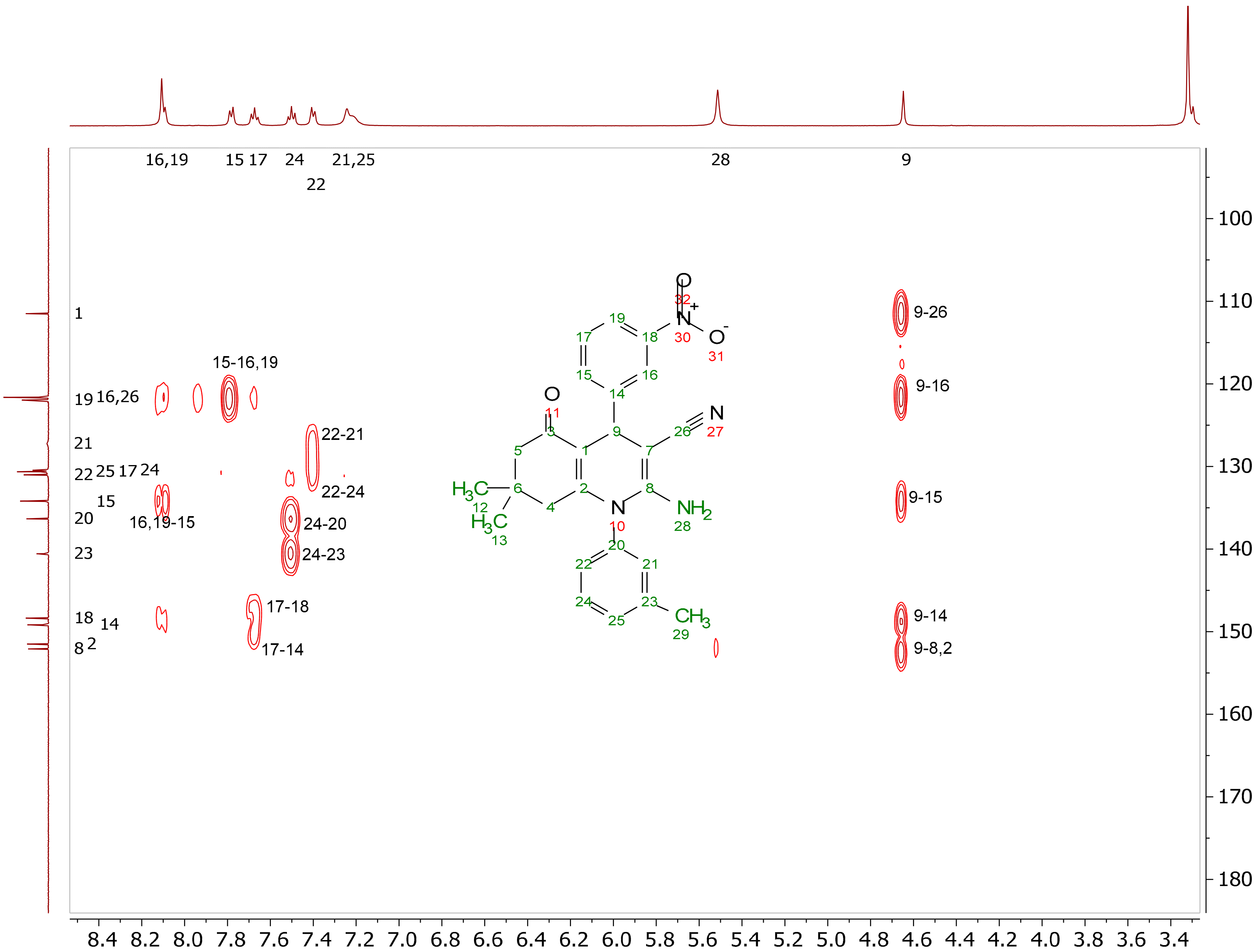

Figure S33 - Downfield region of HMBC spectrum of 5j

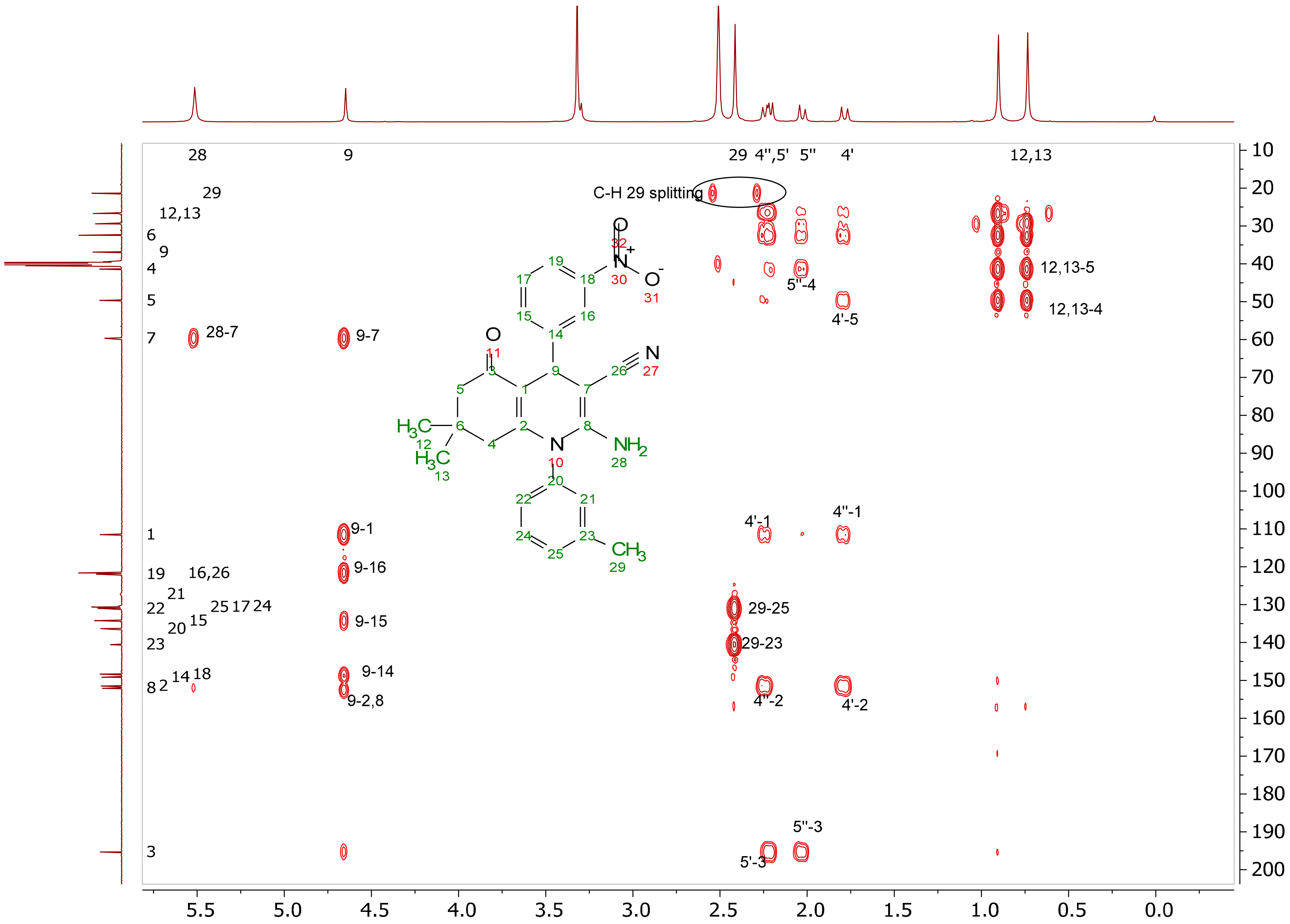

Figure S34 - Upfield region of HMBC spectrum of 5j

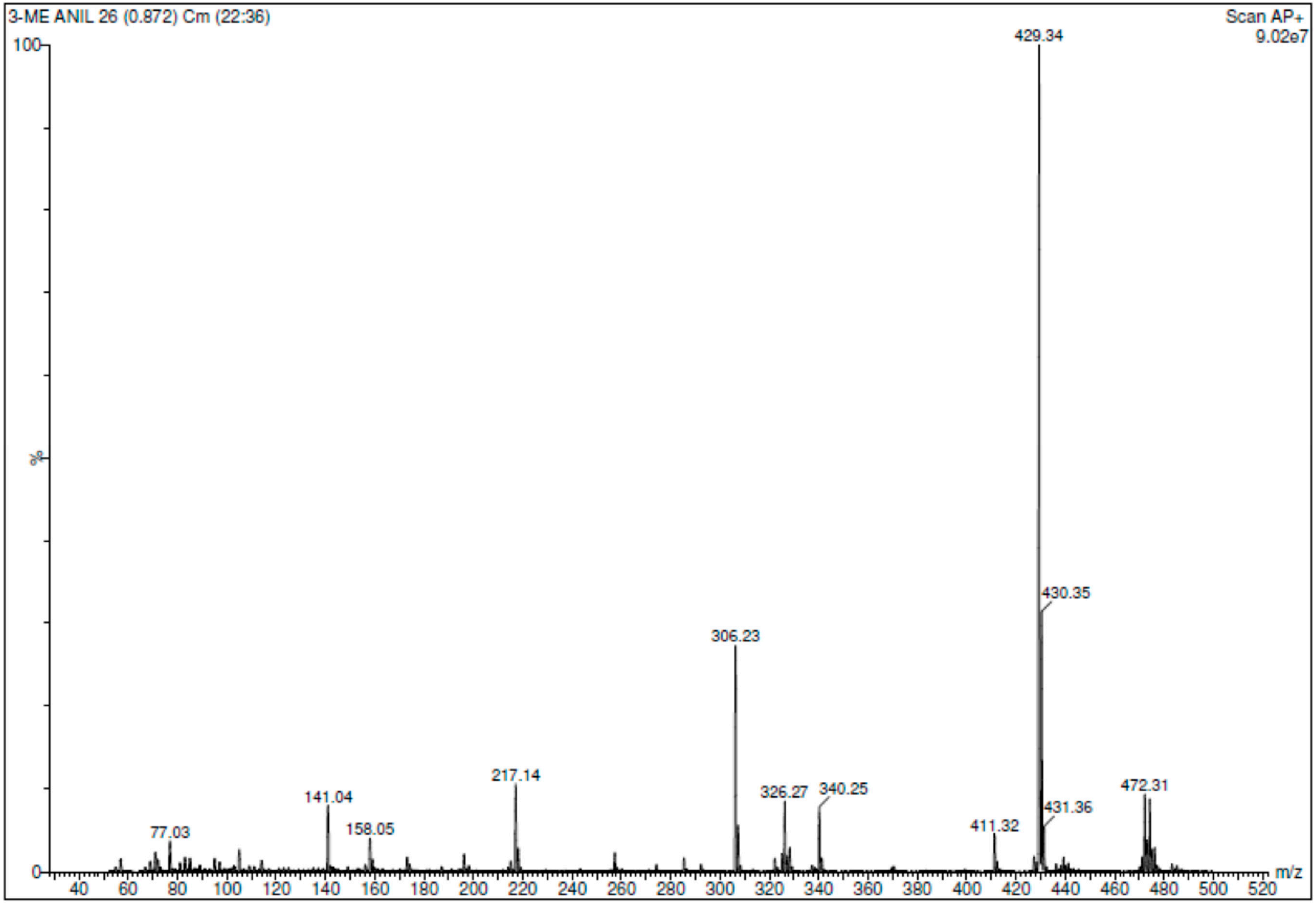

Figure S35 - MS spectrum of 5j

Table S3 - Fragmentation positions for peaks in MS spectrum of 5j

m/z	Fragmentation position and structure
**429.34**	$[M+H]^+$
**340.25**	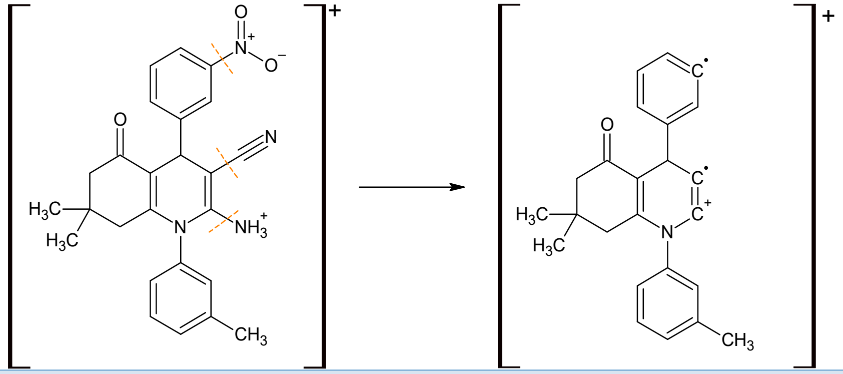
**306.23**	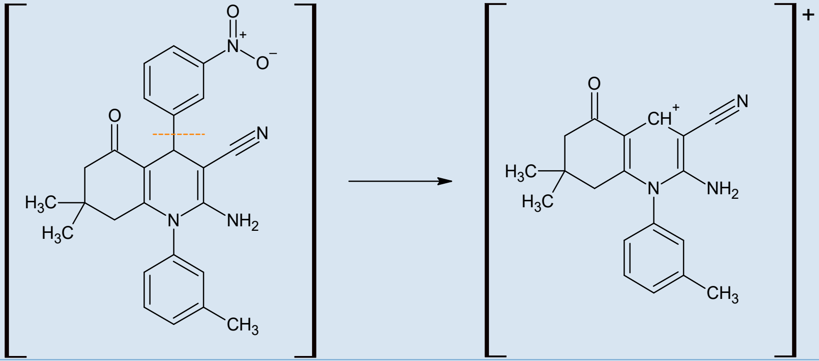

1.11. Product 5k: 2-amino-1-(3-chlorophenyl)-4-(2,4-dichlorophenyl)-7,8-dimethyl-5-oxo-1,4,5,6,7,8-hexahydroquinoline-3-carbonitrile

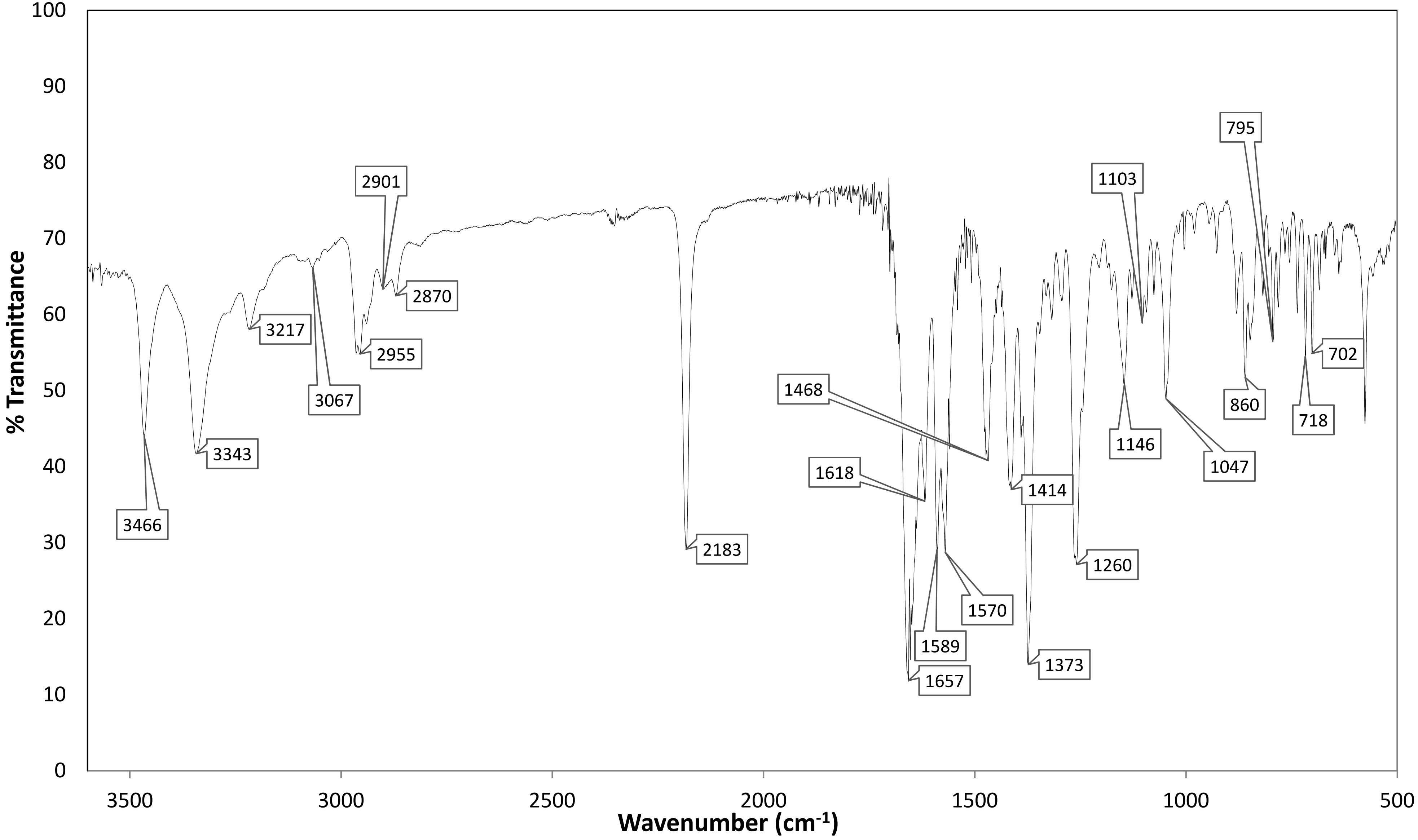

Figure S36 - IR spectrum of 5k

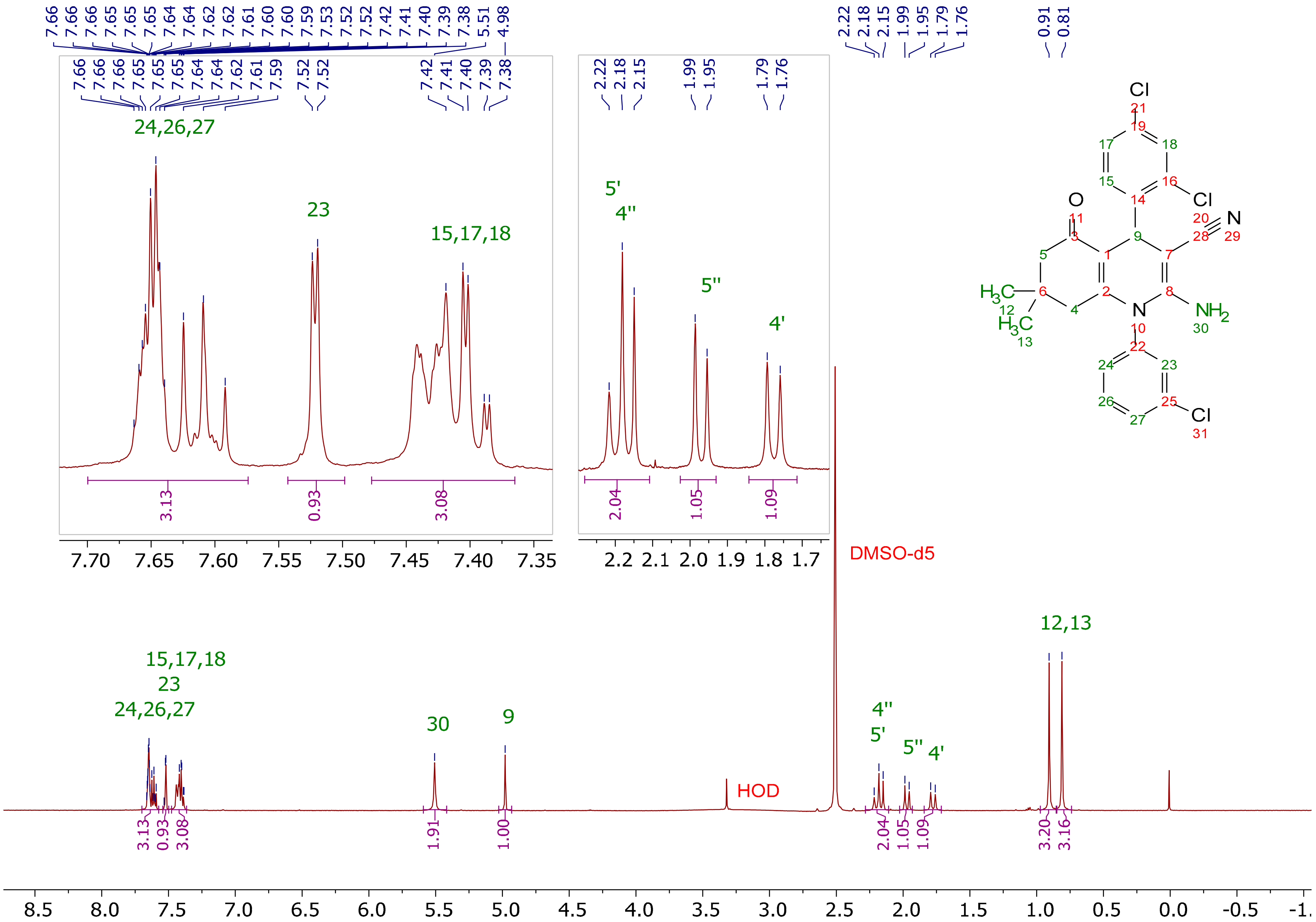

Figure S37 - ^{1}H NMR spectrum of 5k

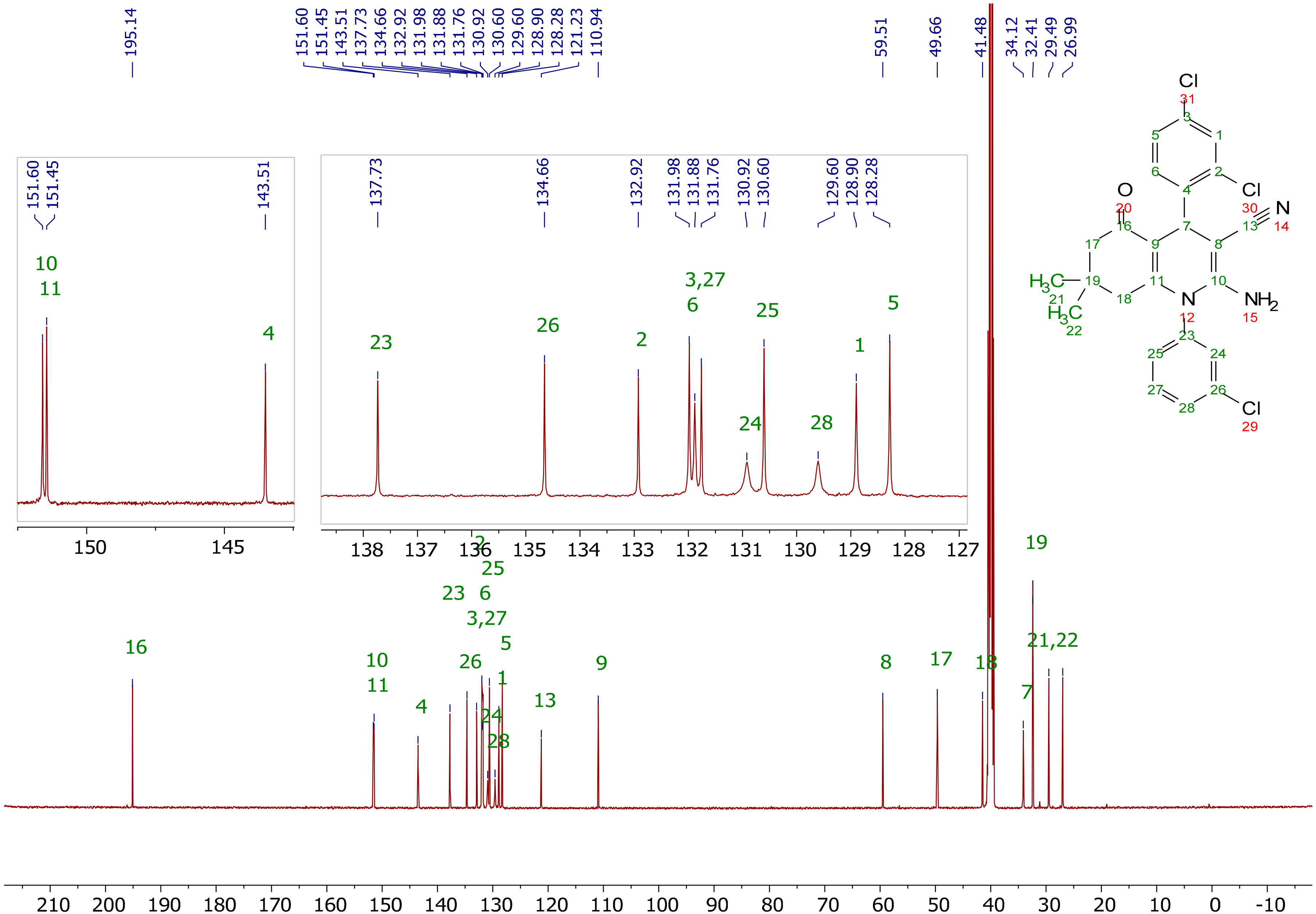

Figure S38 - ^{13}C NMR spectrum of 5k

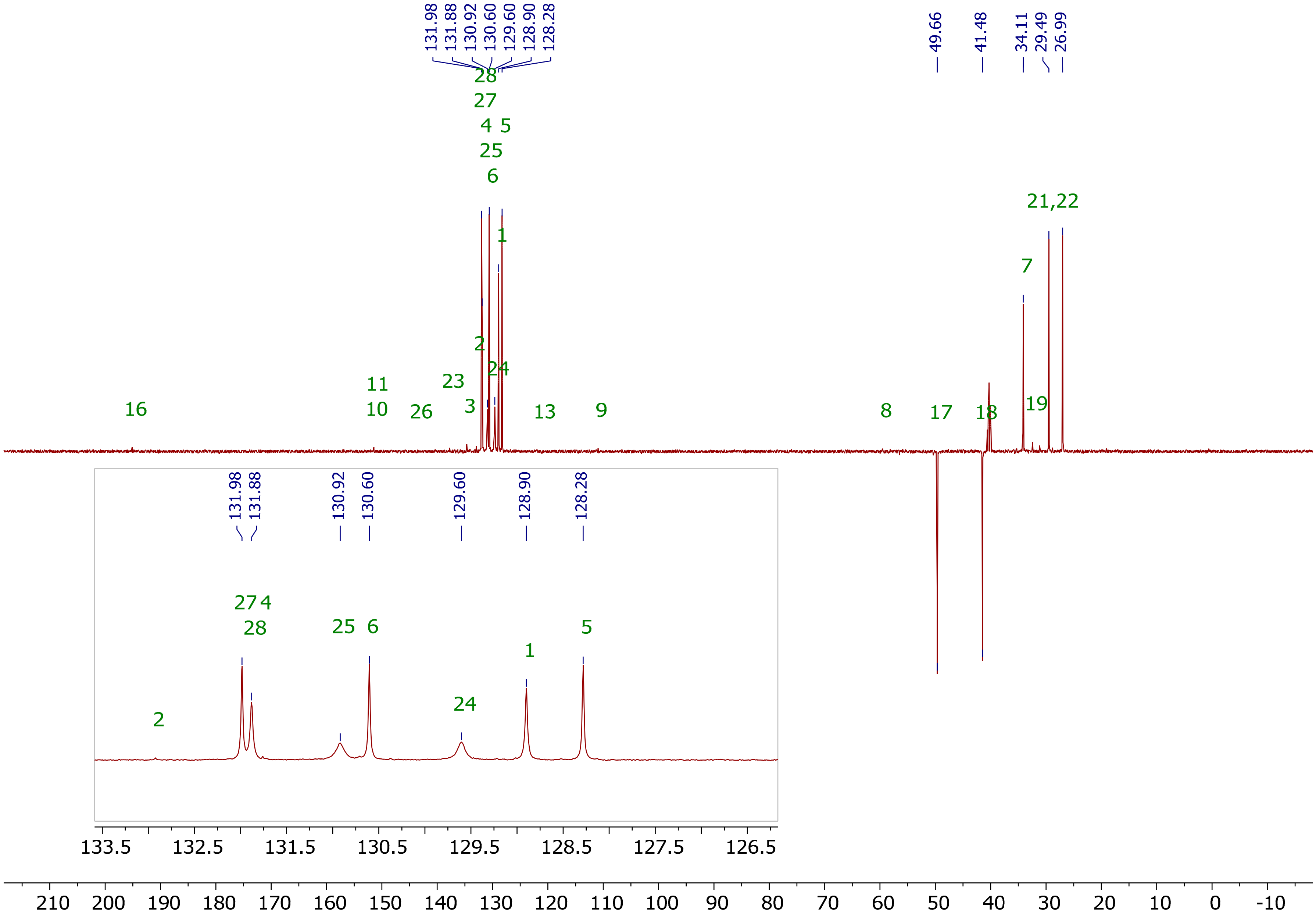

Figure S39 - DEPT spectrum of 5k

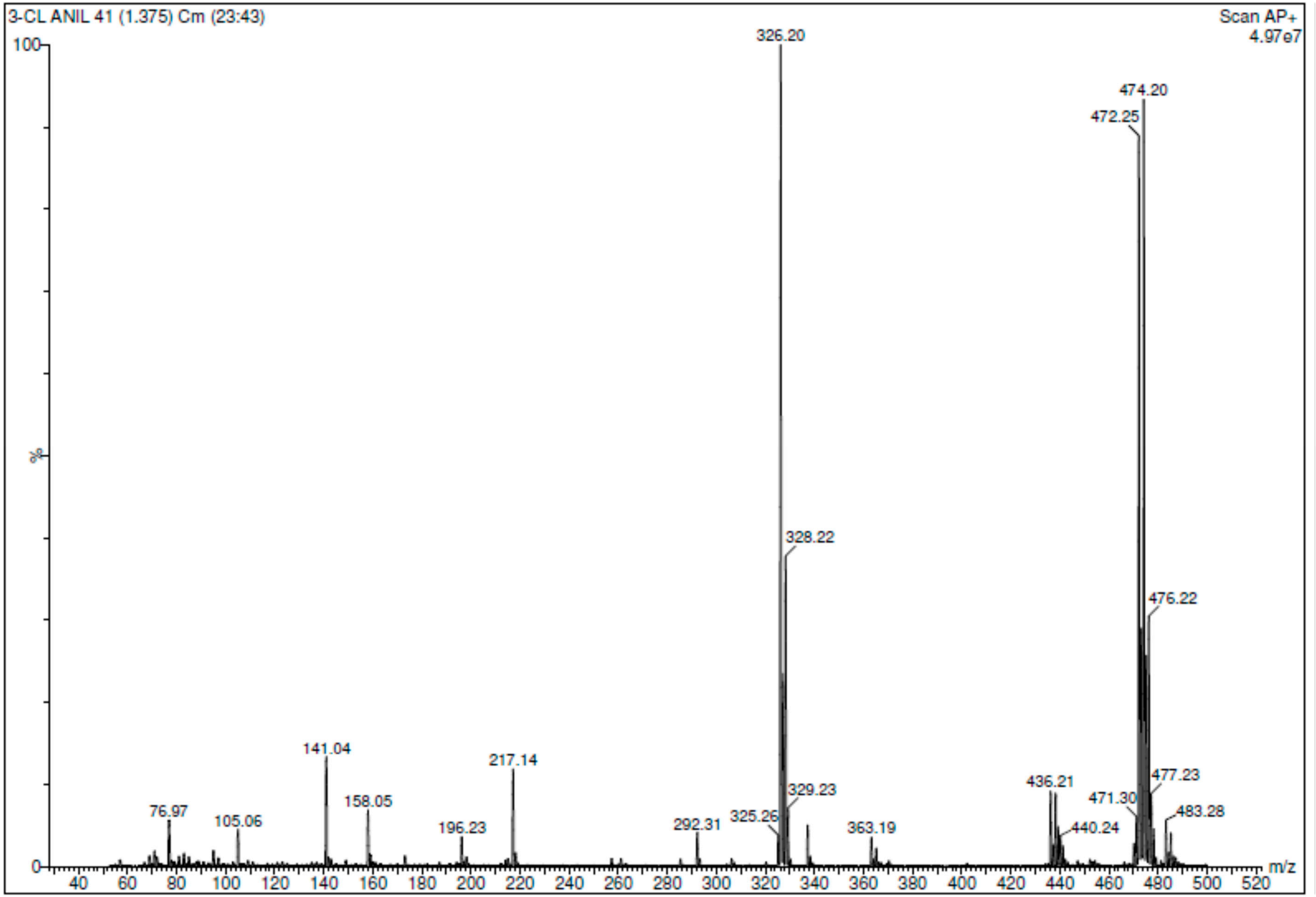

Figure S40 - MS spectrum of 5k

Table S4 - Fragmentation positions for peaks n MS spectrum of 5k

m/z	Fragmentation position and structure
**471.30 – 477.23**	$[M+H]^+$ Multiple isotopes
**436.22-440.24**	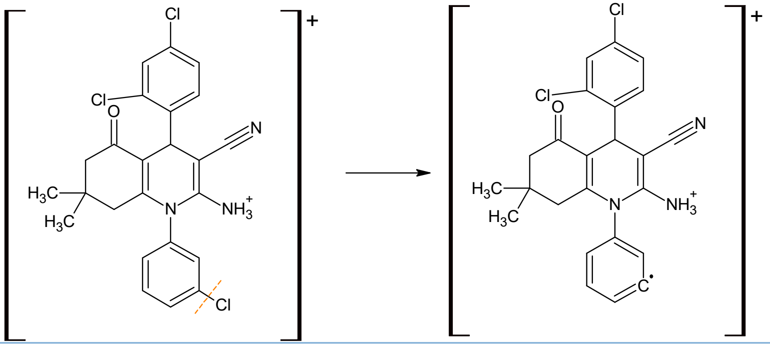
**326.20 – 328.22**	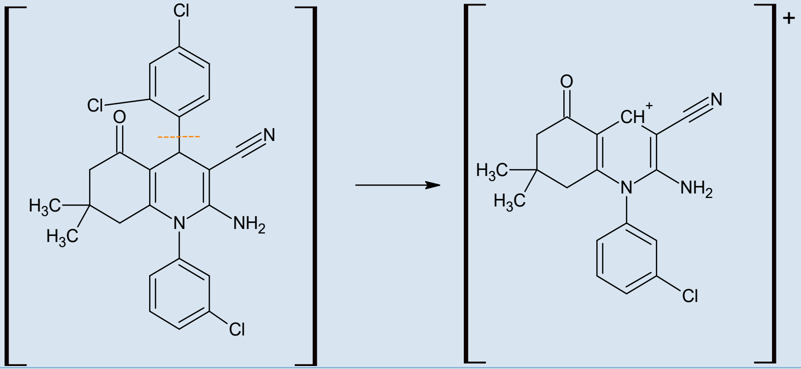

1.12. Product 5l: 2-amino-4-(2,4-dichlorophenyl)-7,8-dimethyl-1-(3-nitrophenyl)-5-oxo-1,4,5,6,7,8-hexahydroquinoline-3-carbonitrile

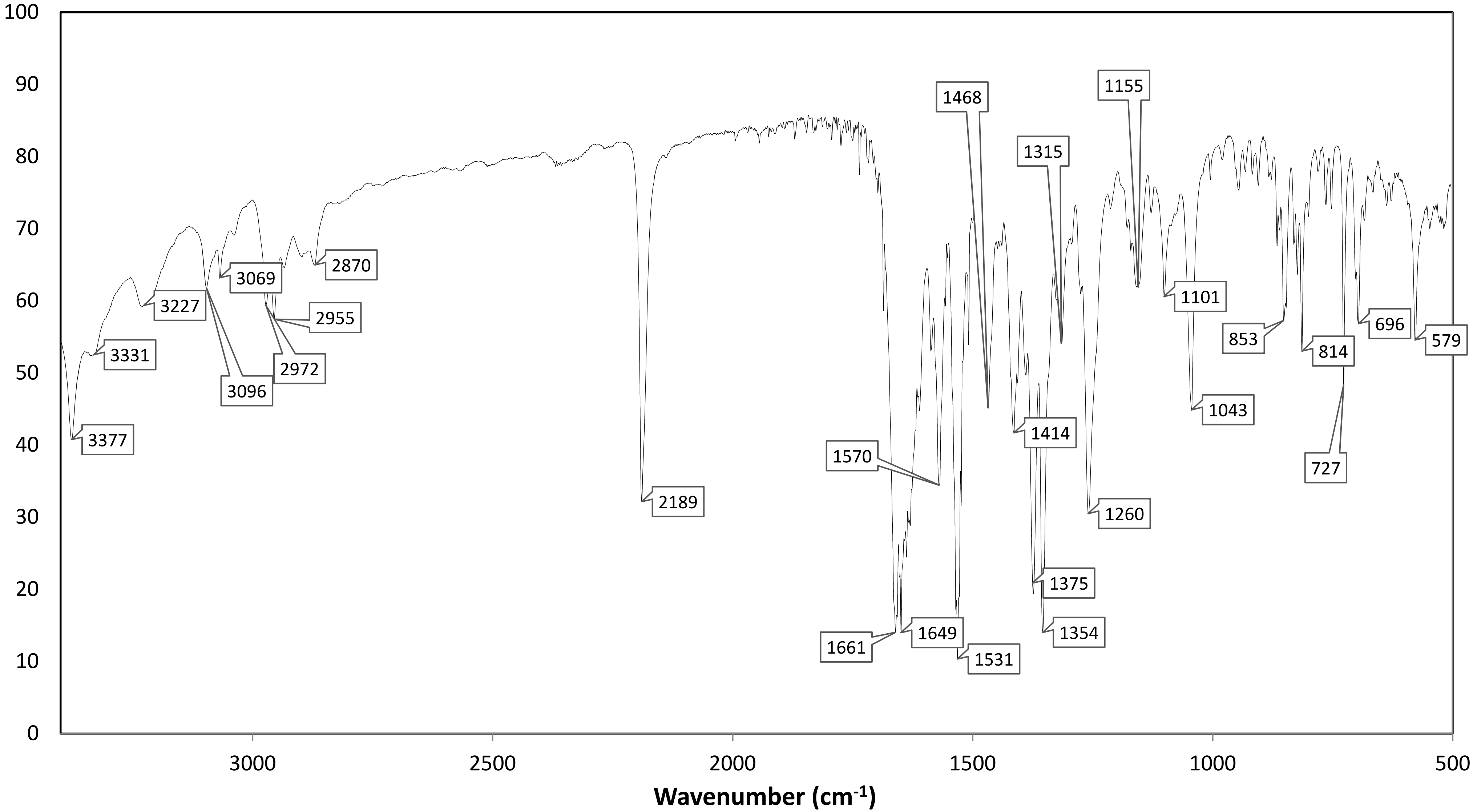

Figure S41 - IR spectrum of 5l

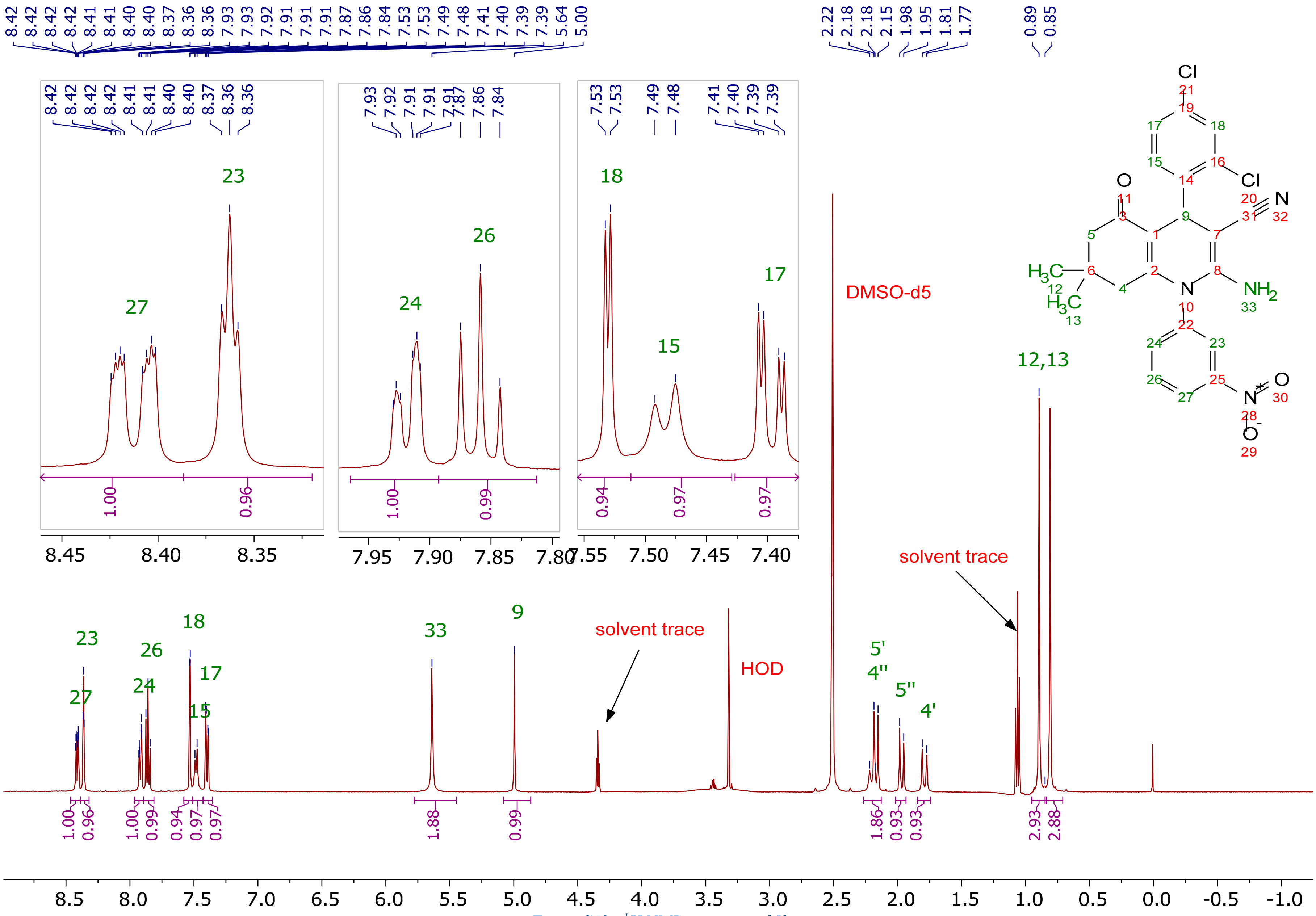

Figure S42 - ^{1}H NMR spectrum of 5l

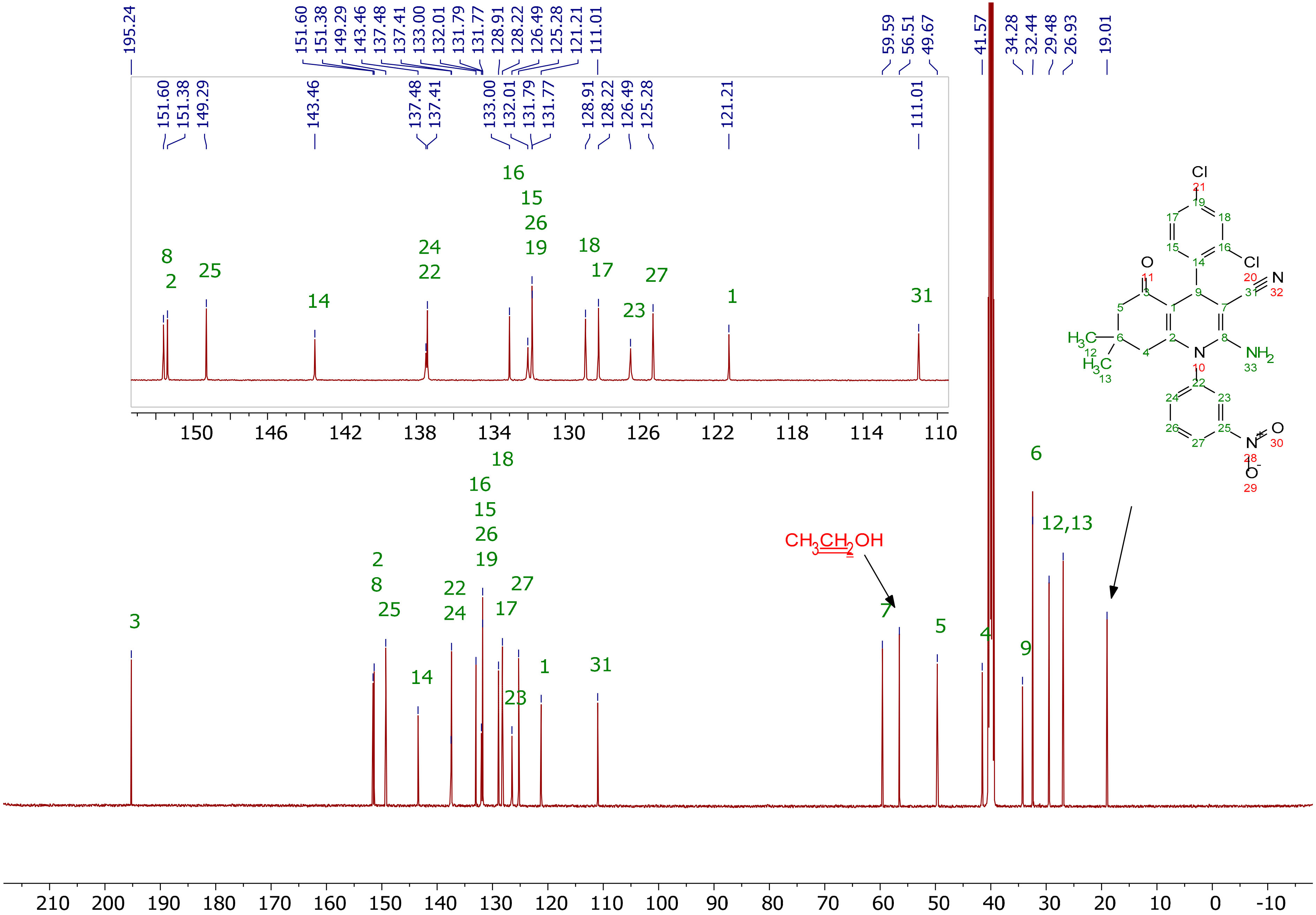

Figure S43 - ^{13}C NMR spectrum of 5l

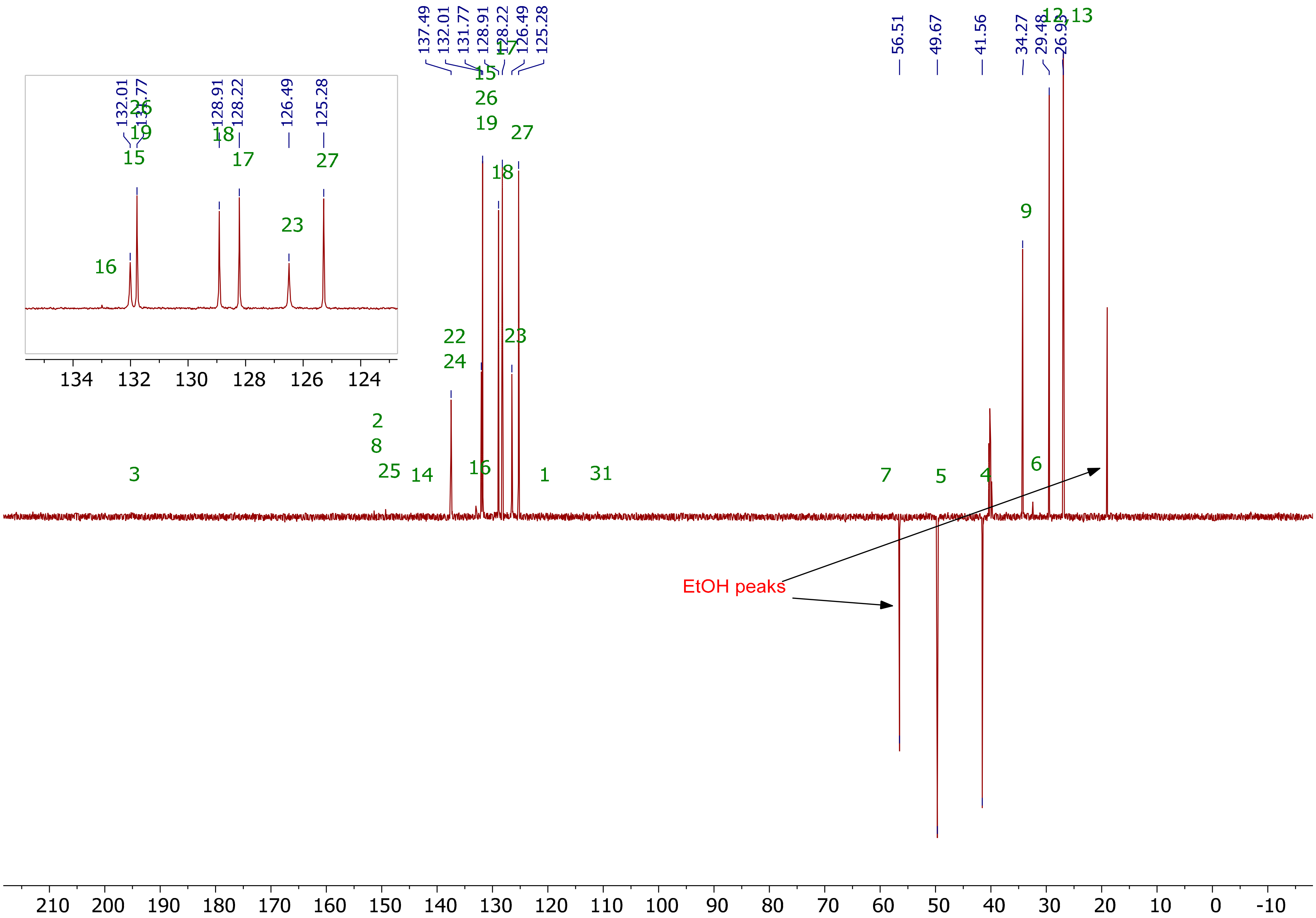

Figure S44 - DEPT spectrum of 5l

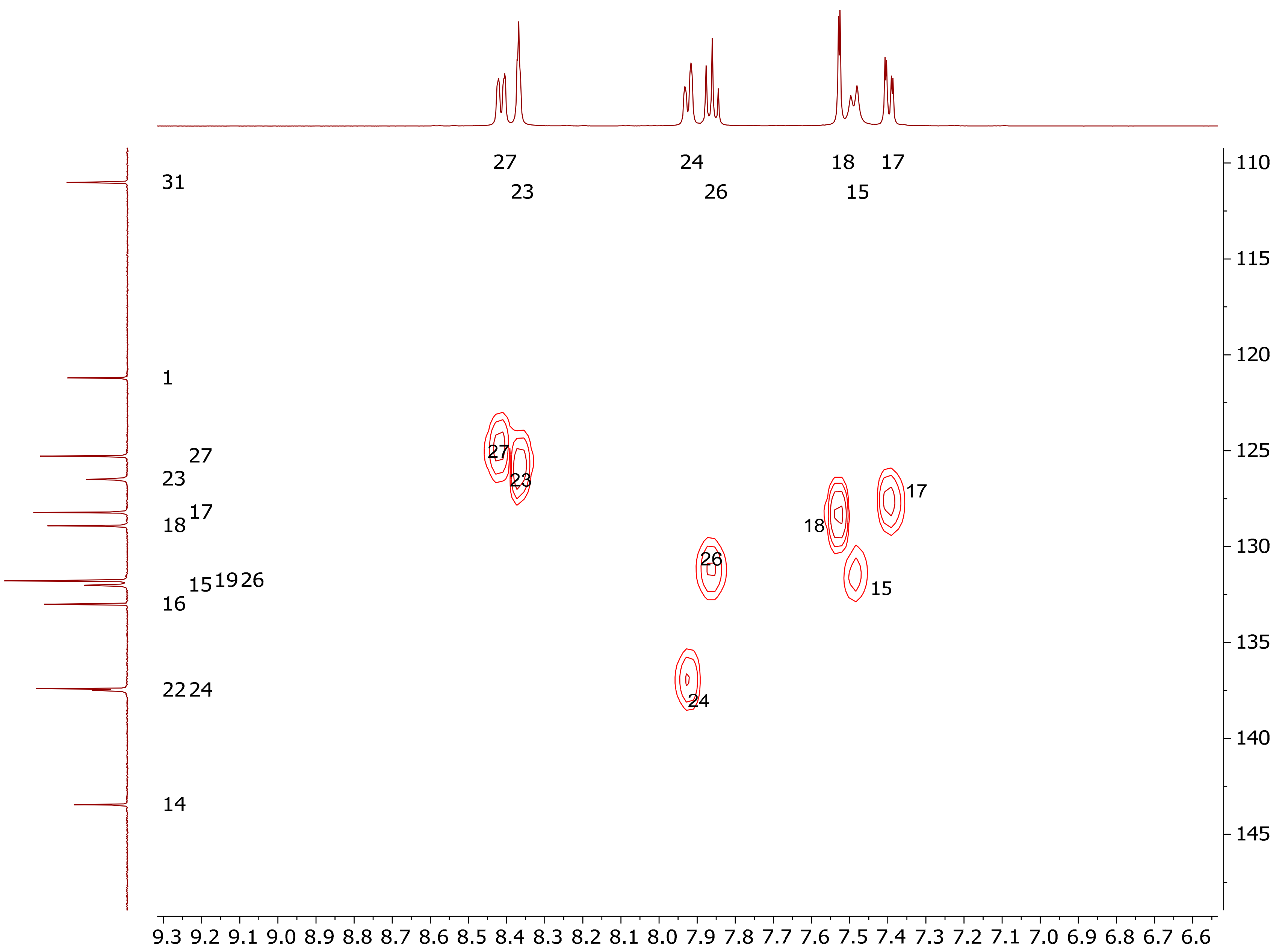

Figure S45 - HSQC spectrum of 5l

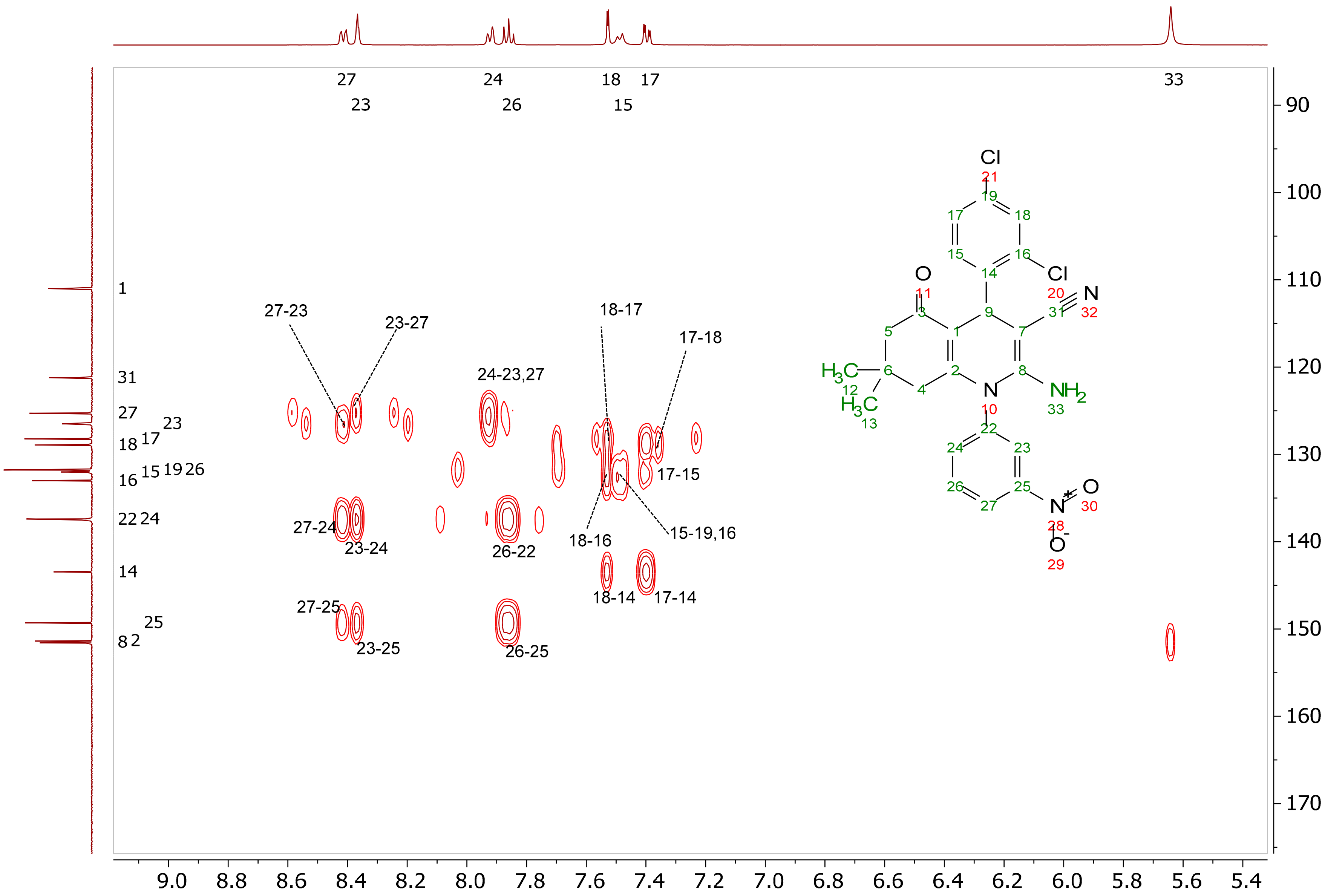

Figure S46 – Downfield region of HMBC spectrum of 5l

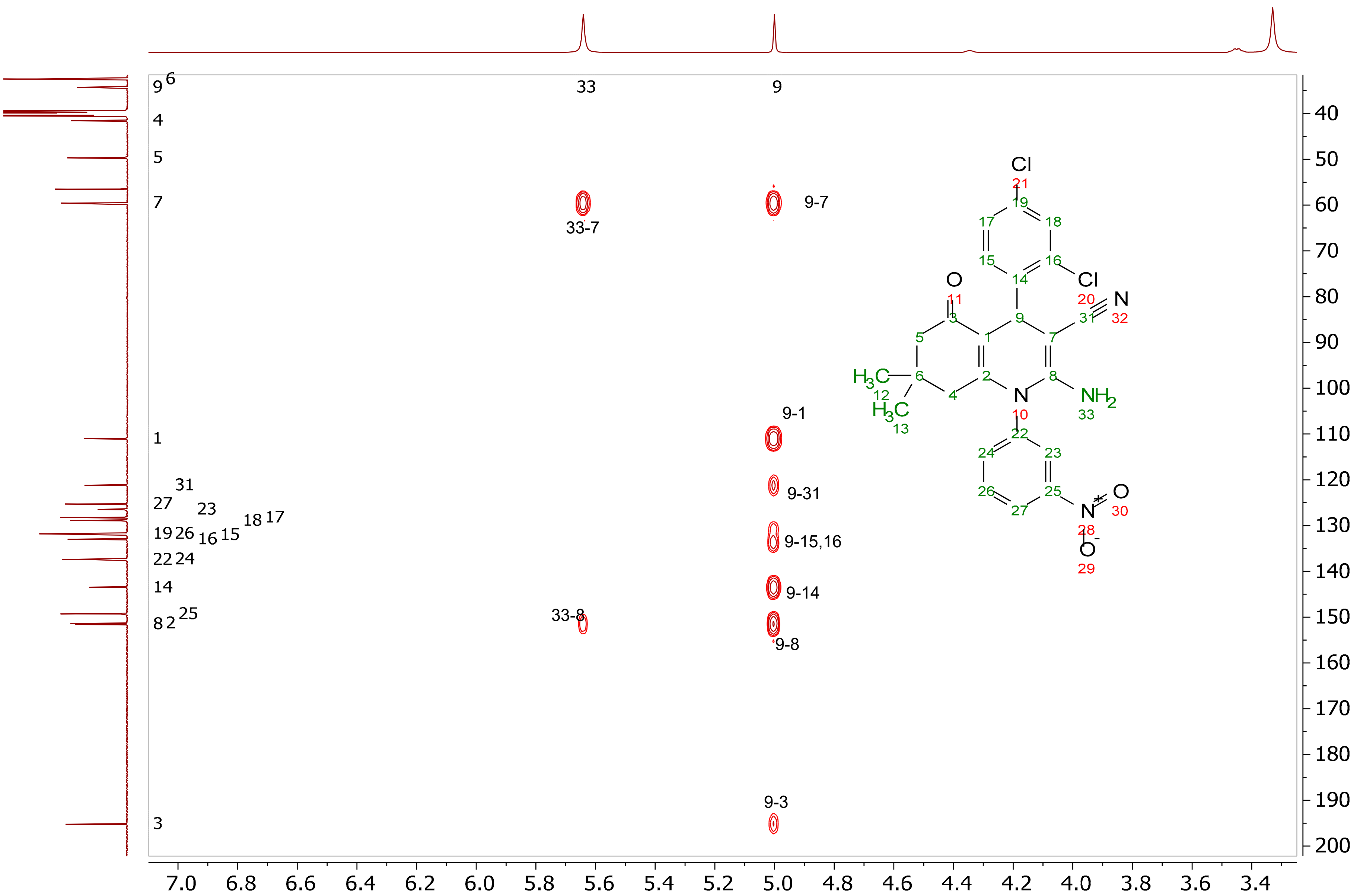

Figure S47 - Upfield region of HMBC spectrum of 5l

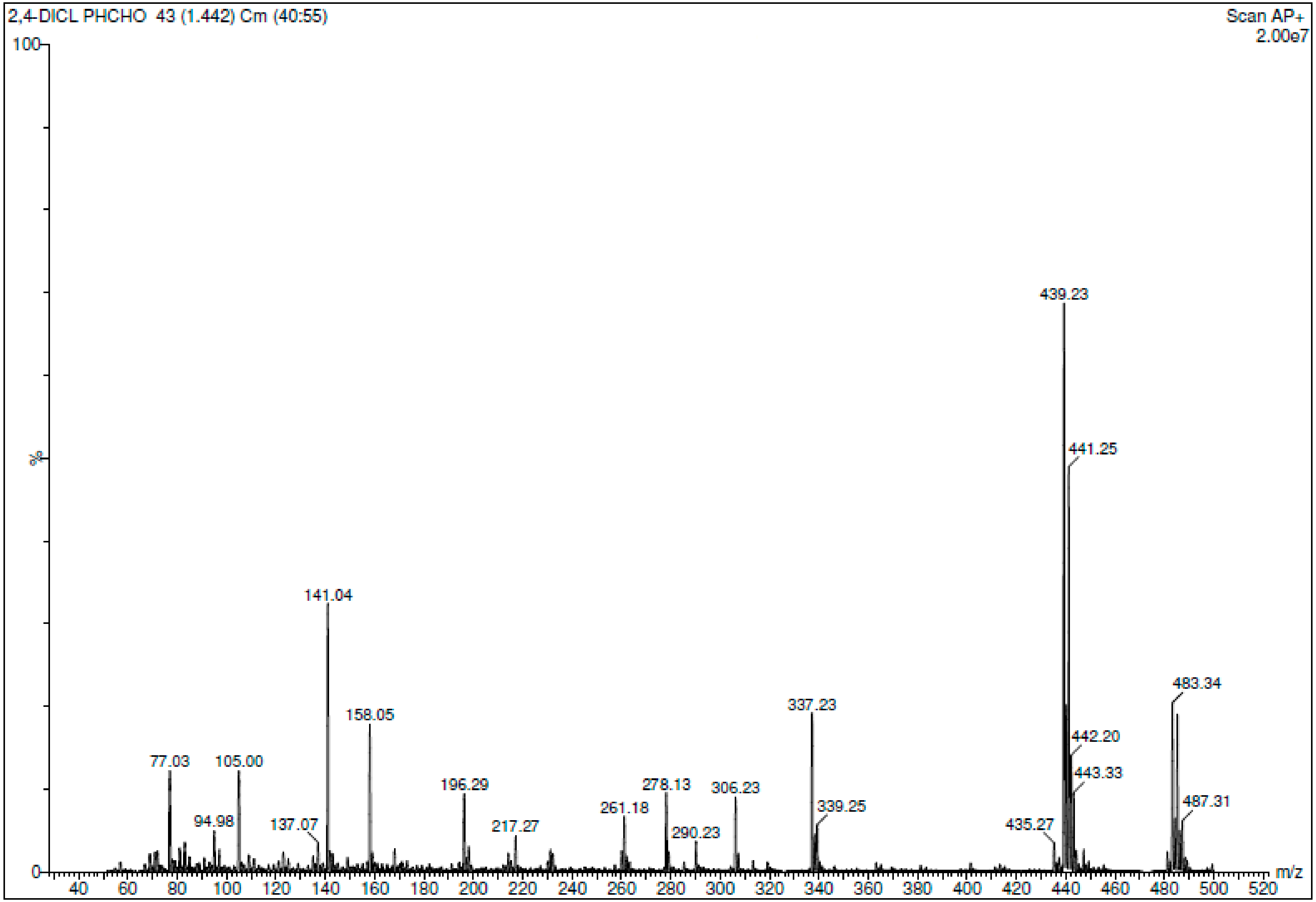

Figure S48 - MS spectrum of 5l

Table S5 – Peaks and fragmentation positions for 5l

m/z	Fragmentation position and structure
**483.34-487.31**	$[M+H]^+$<br>Multiple isotopes
**439.23**	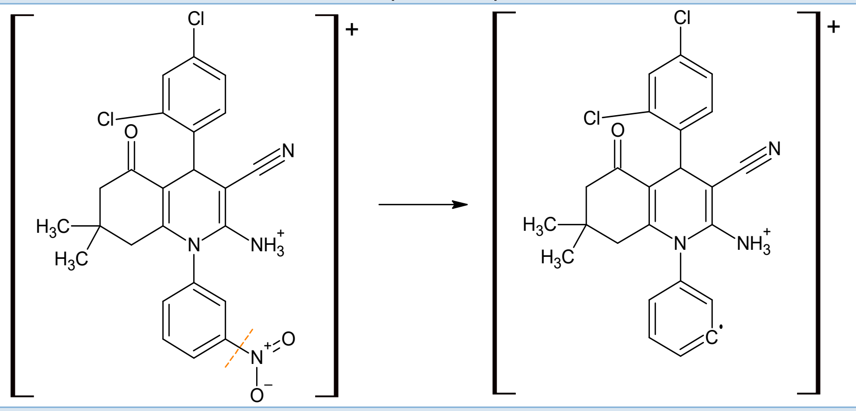
**337.23**	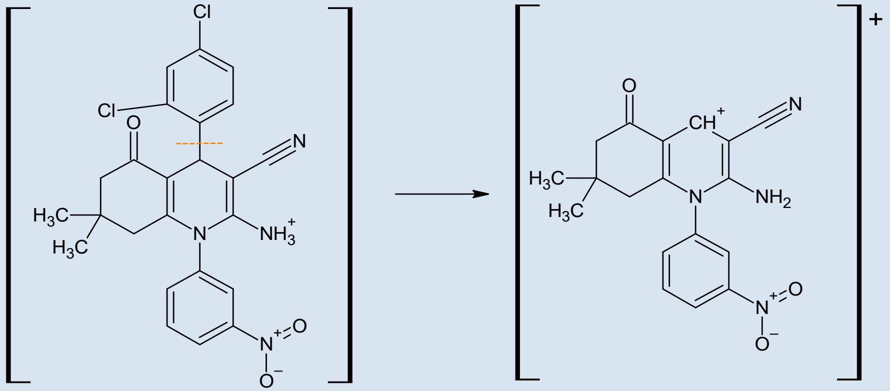
**158.05**	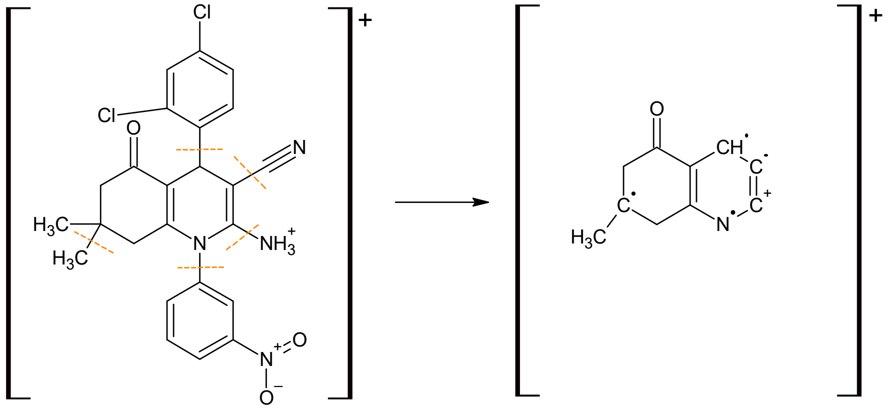

1.13. Product 5m: 2-amino-1-(4-methoxyphenyl)-7,8-dimethyl-4-(3-methylphenyl)-5-oxo-1,4,5,6,7,8-hexahydroquinoline-3-carbonitrile

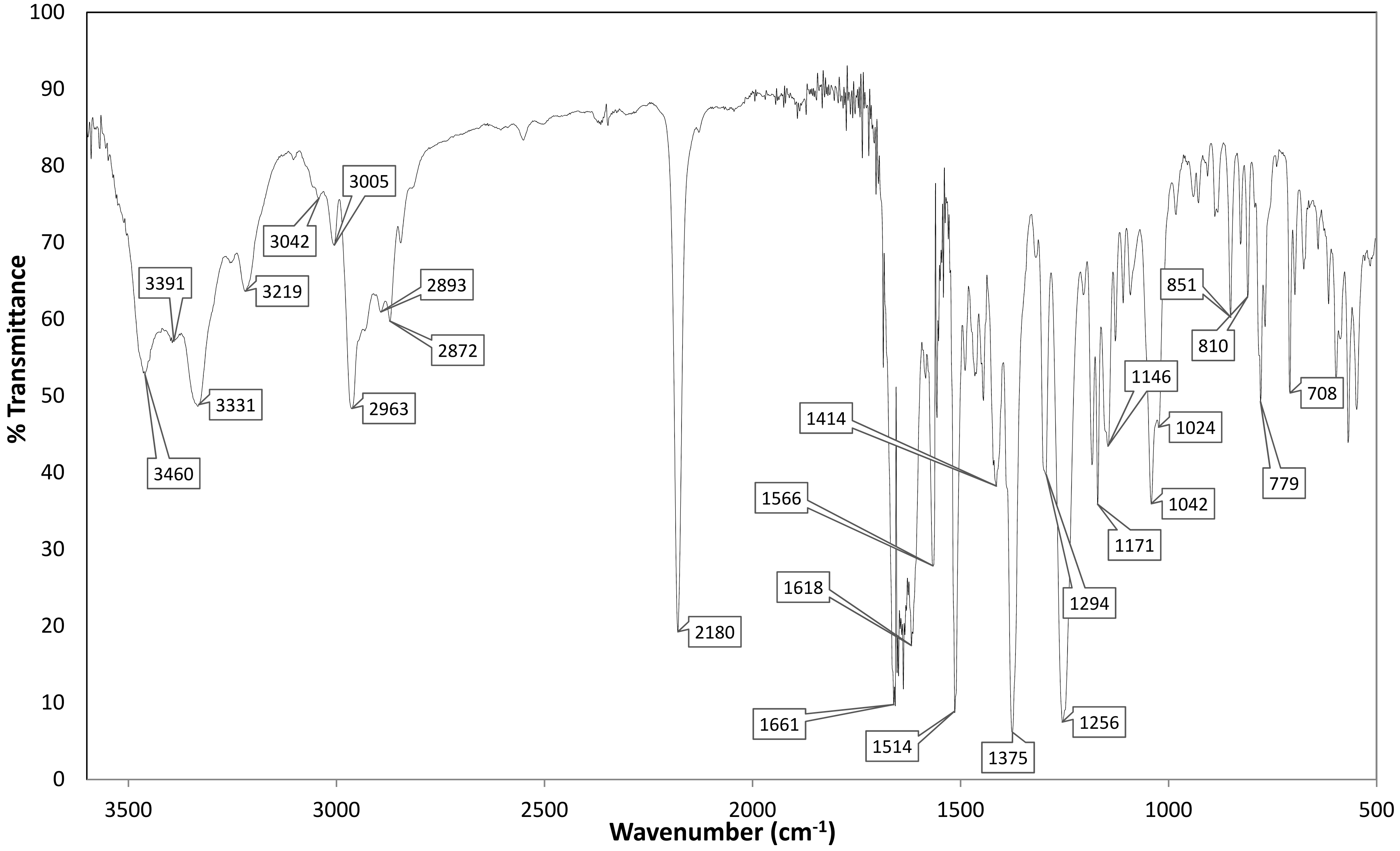

Figure S49 - IR spectrum of 5m

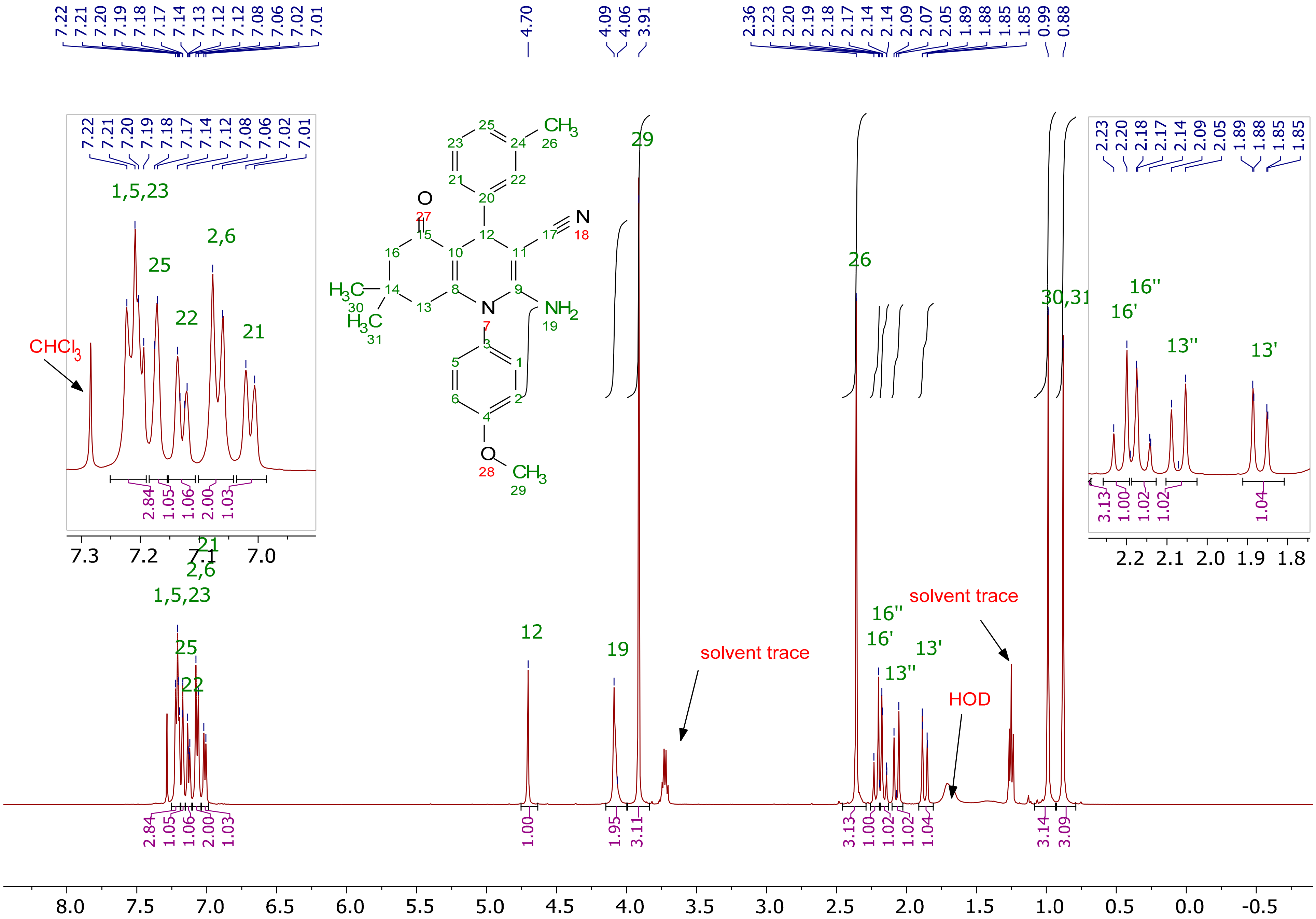

Figure S50 - ^{1}H NMR spectrum of 5m

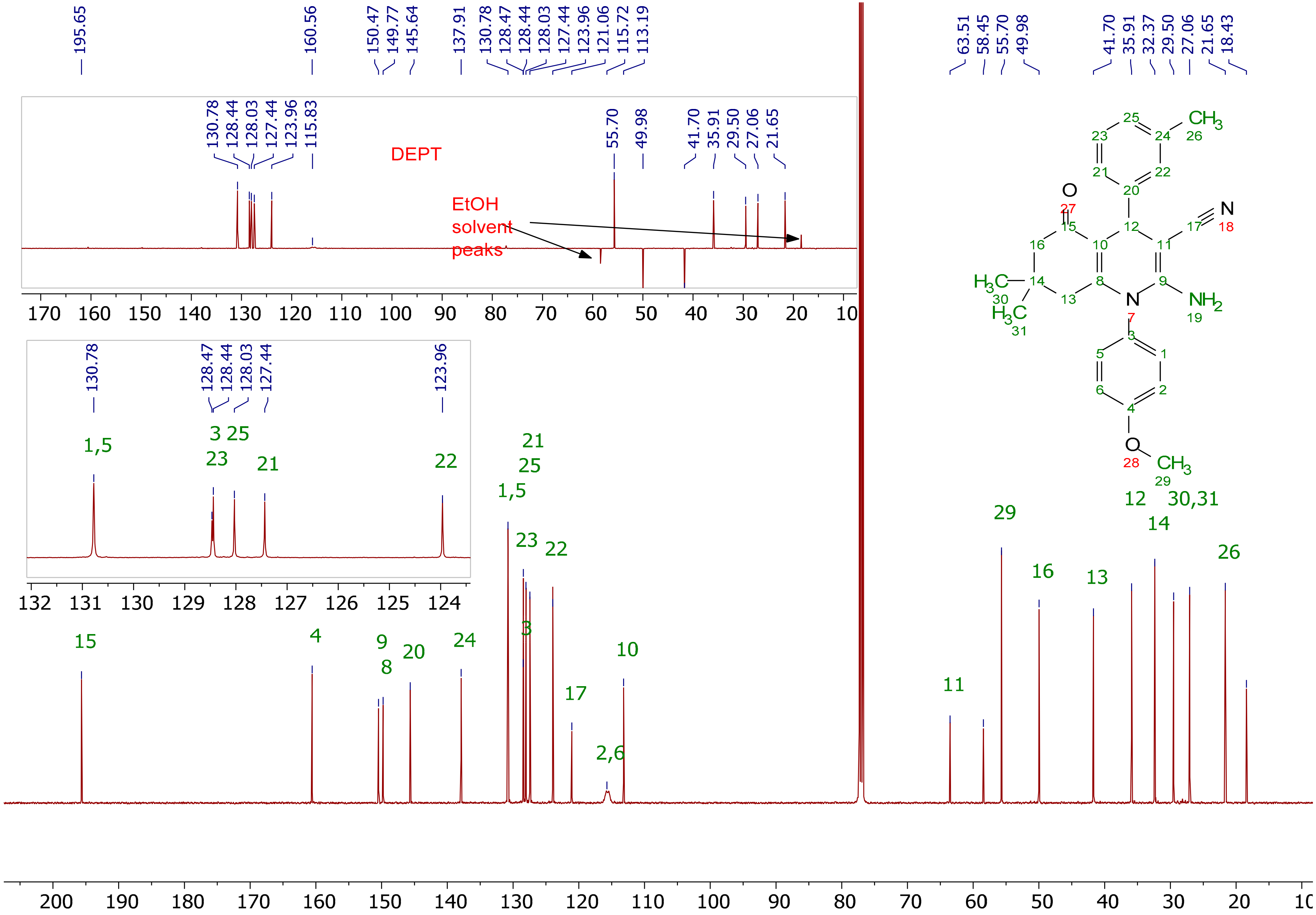

Figure S51 - ^{13}C NMR spectrum of 5m with inset expansion of DEPT spectrum

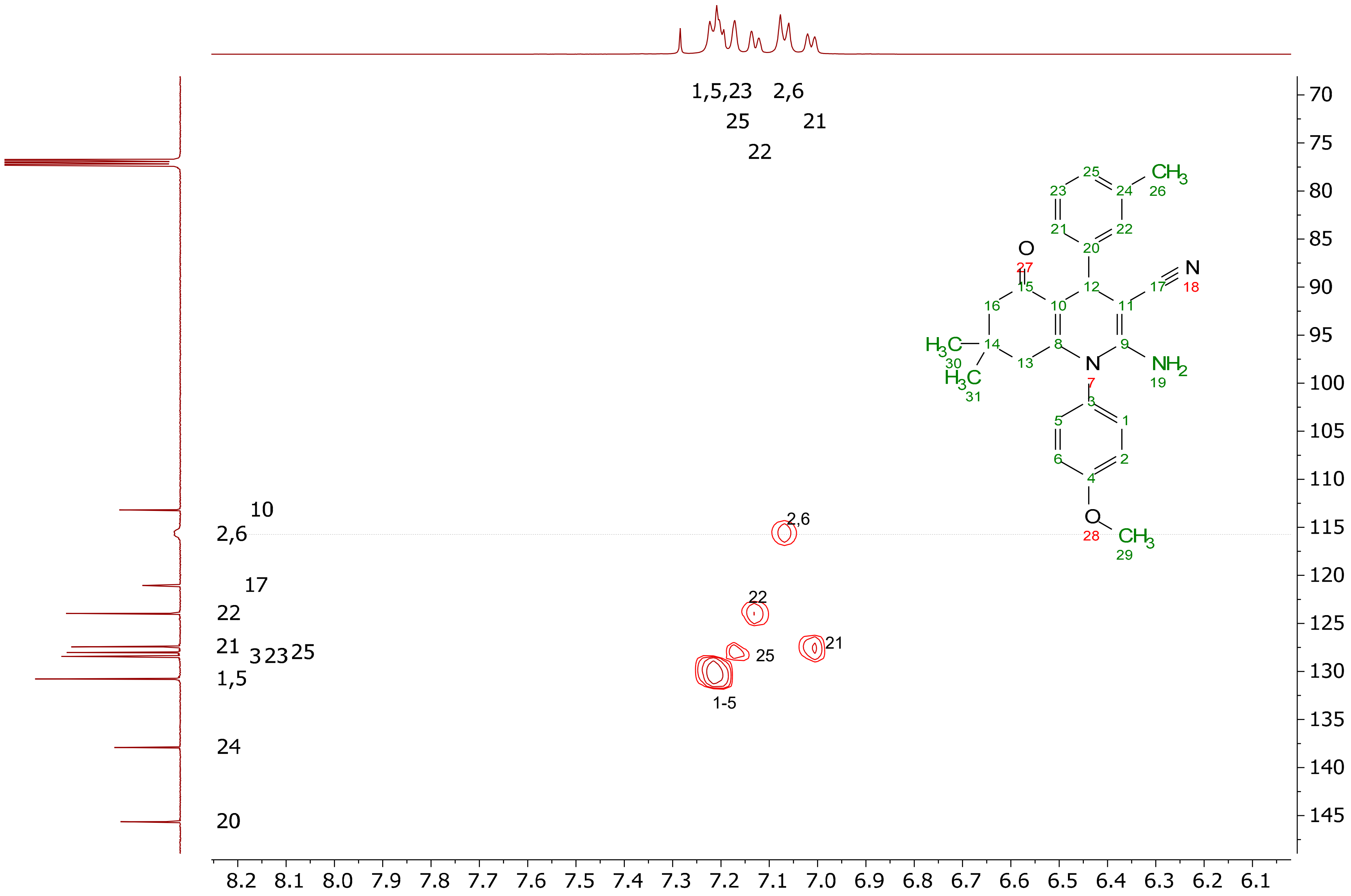

Figure S52 – Downfield region of HSQC NMR spectrum of 5m

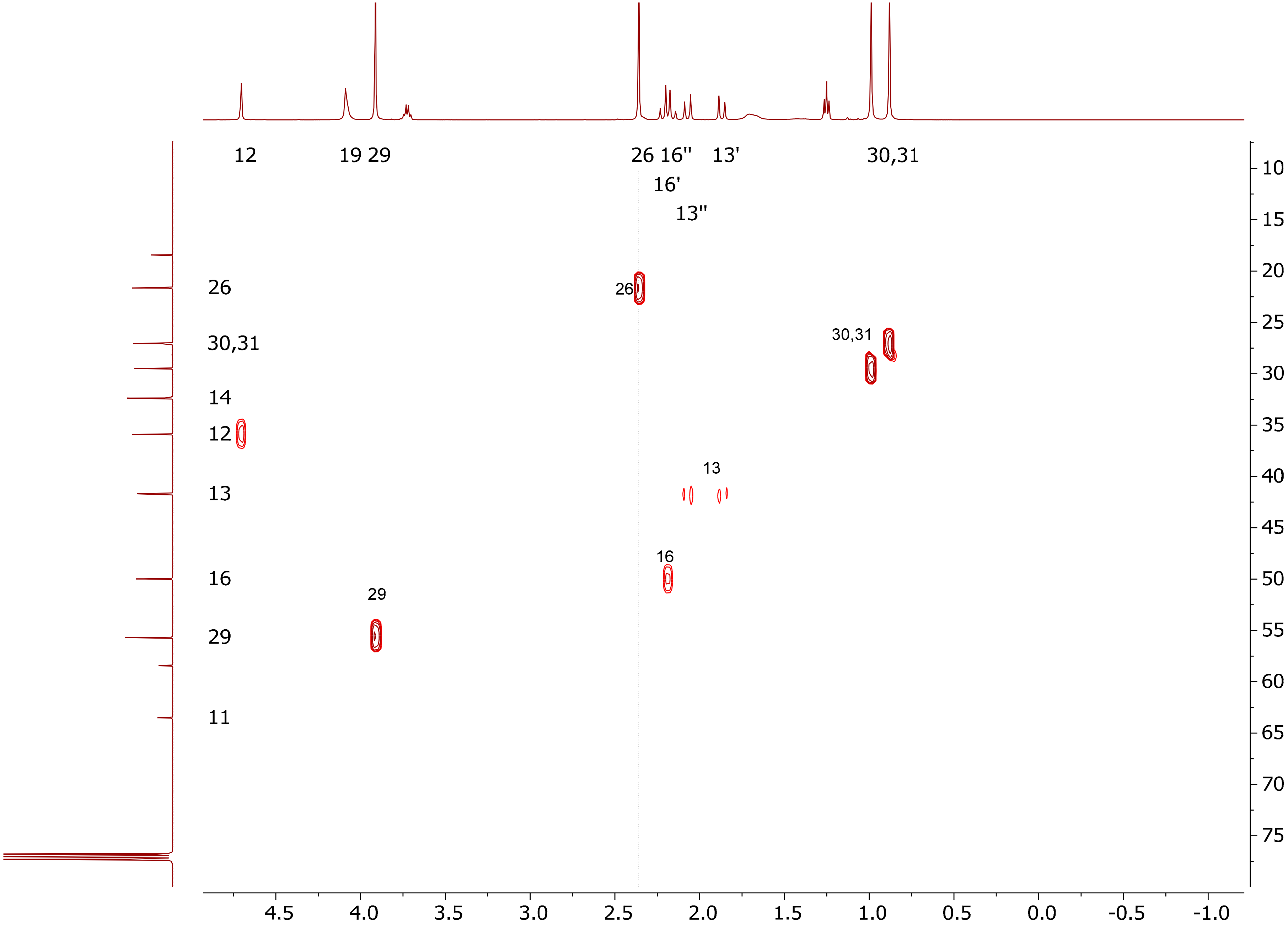

Figure S53 - Upfield region of HSQC spectrum of 5m

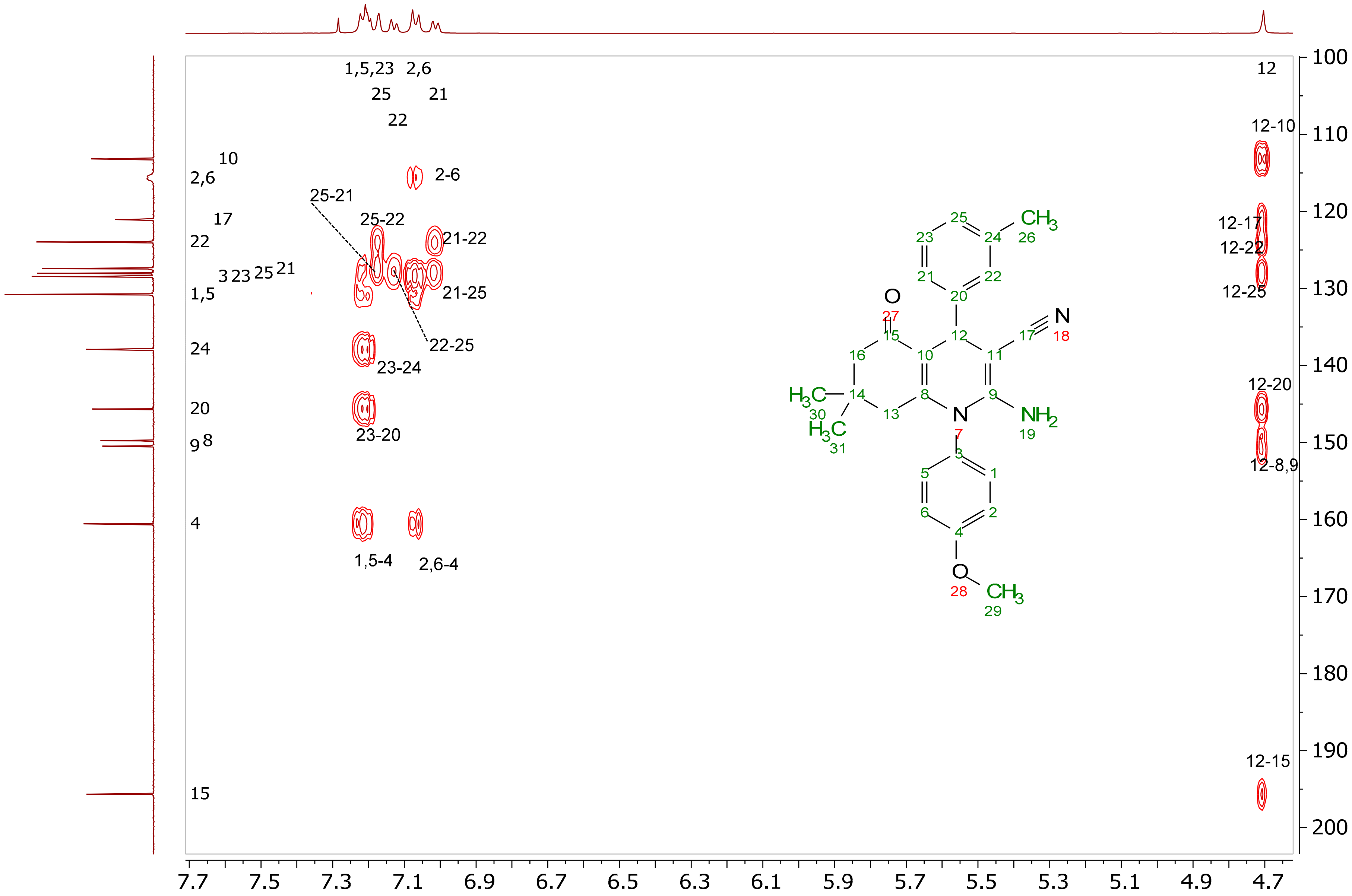

Figure S54 - Downfield region of HMBC NMR spectrum of 5m

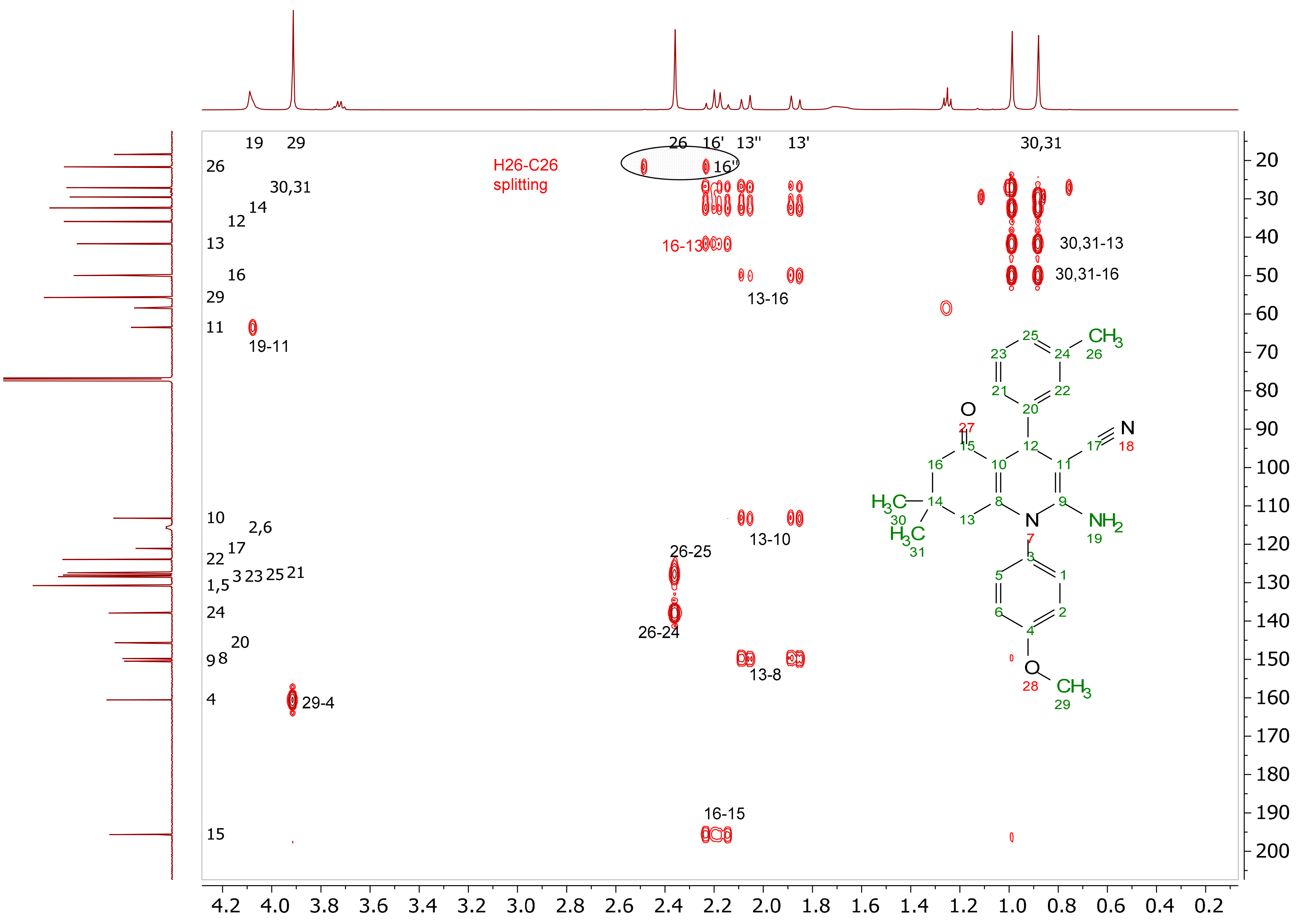

Figure S55 - Upfield region of HMBC NMR spectrum of 5m

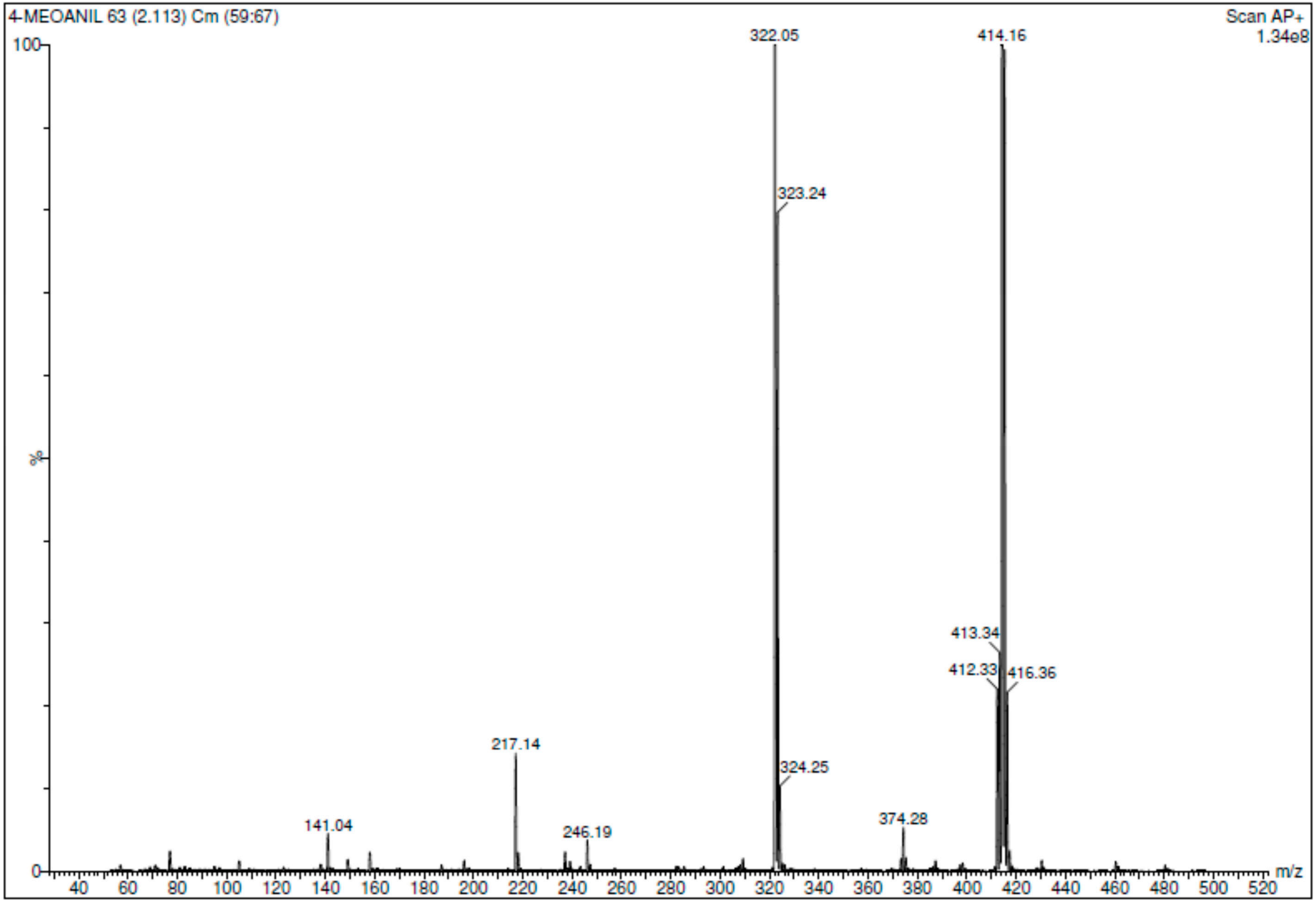

Figure S56 - MS spectrum of 5m

Table S6 - Fragmentation positions for peaks in MS spectrum of 29m

m/z	Fragmentation position and structure
**414.16**	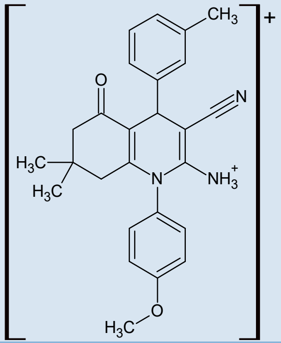
**322.05**	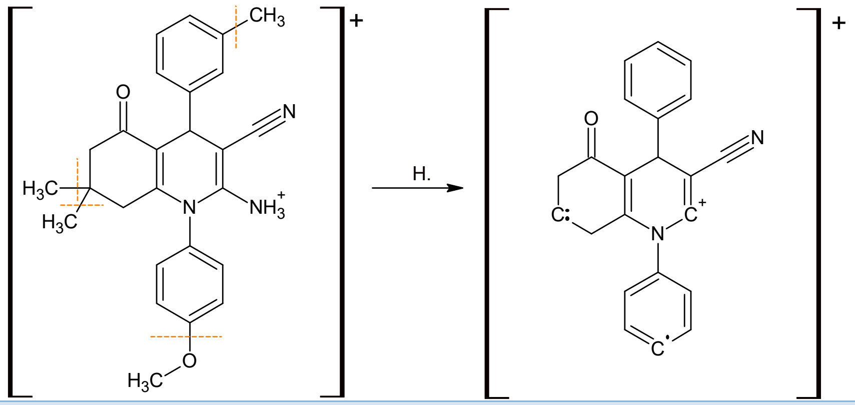
**217.14**	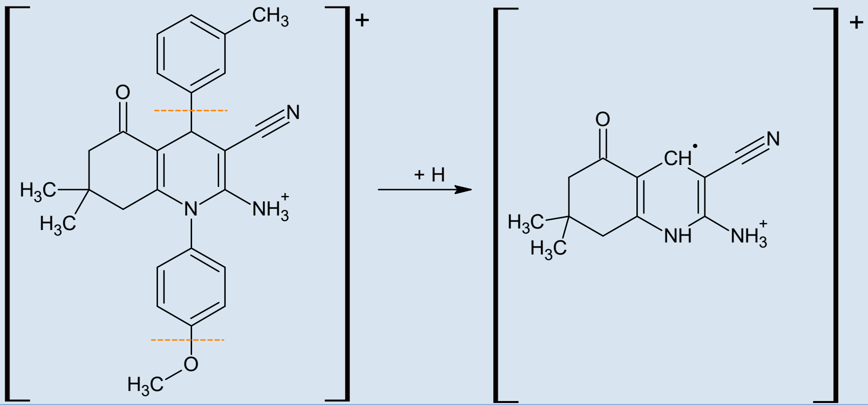

1.14. Product 5n: 2-amino-4-(2,4-dichlorophenyl)-7,8-dimethyl-1-(methylphenyl)-5-oxo-1,4,5,6,7,8-hexahydroquinoline-3-carbonitrile

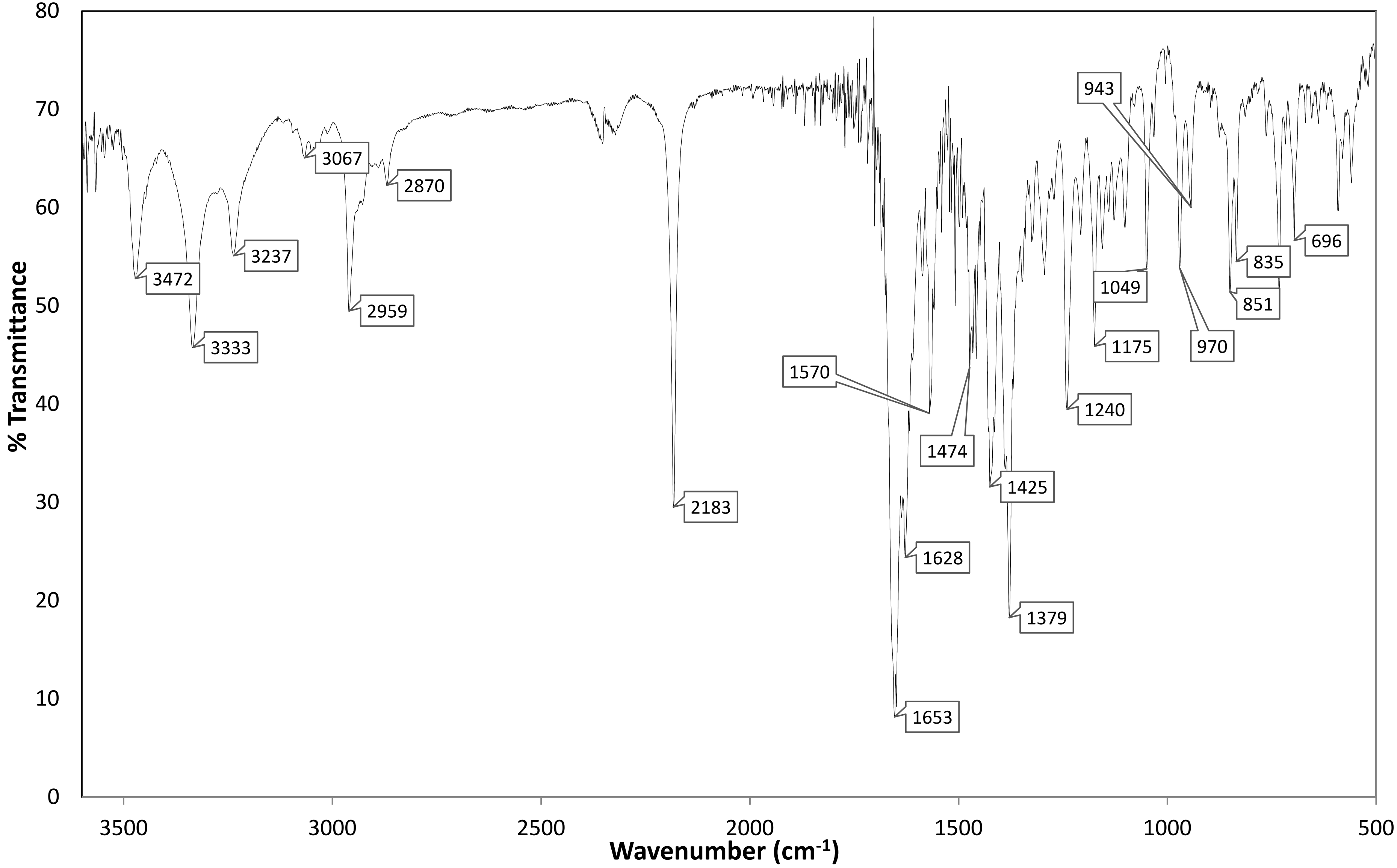

Figure S57 - IR spectrum of 5n

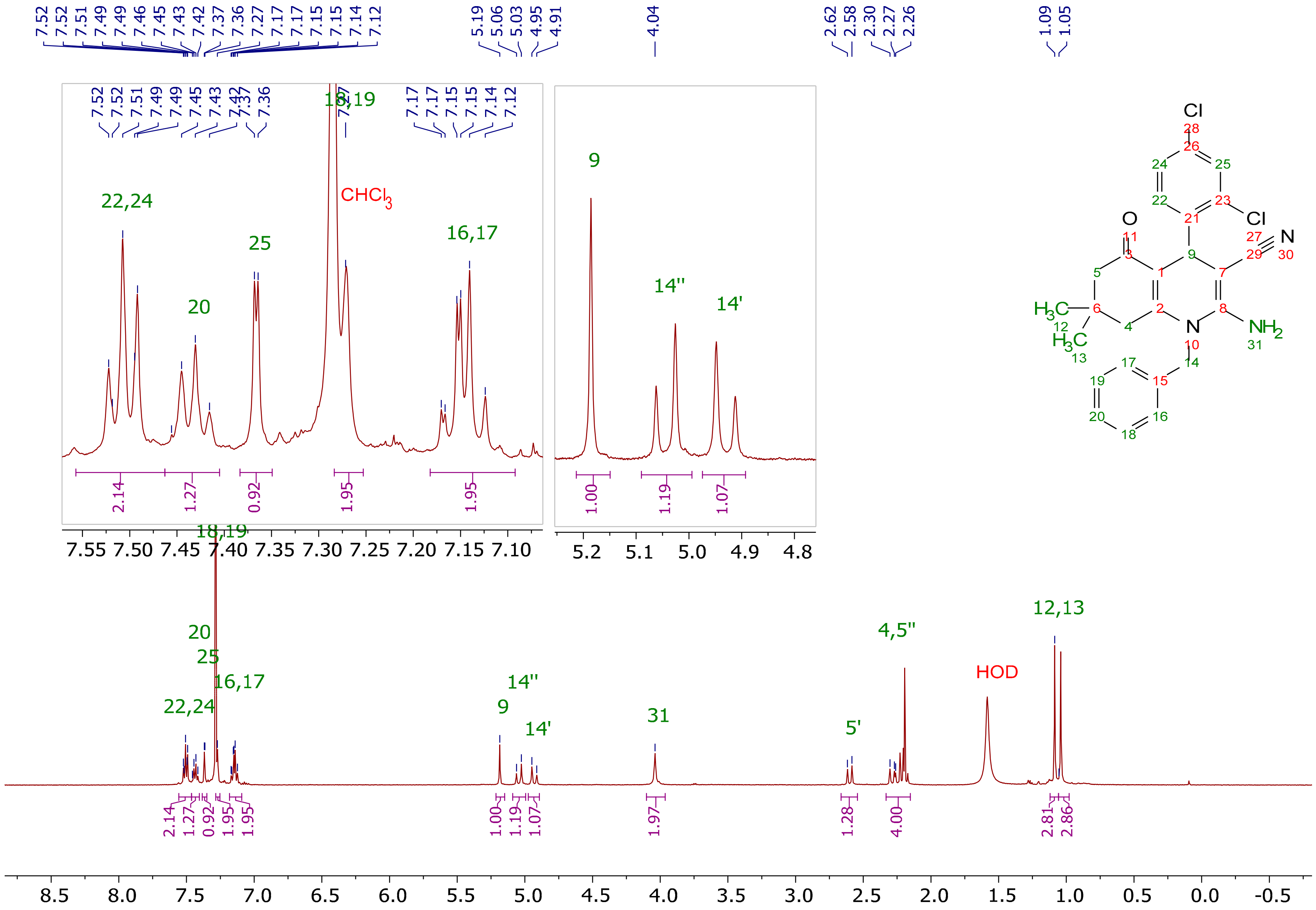

Figure S58 - 1H NMR spectrum of 5n

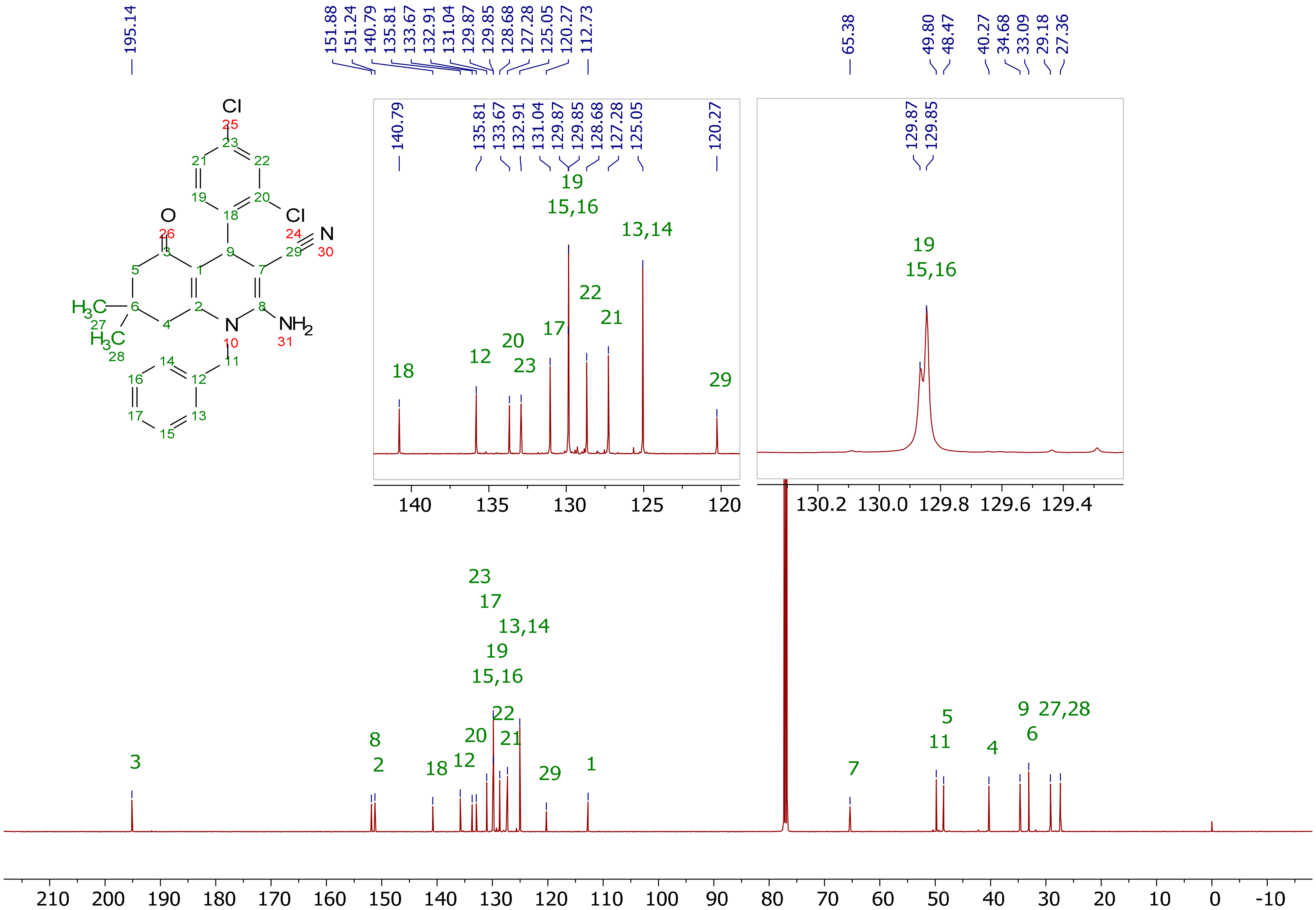

Figure S59 – ^{13}C NMR spectrum of 5n

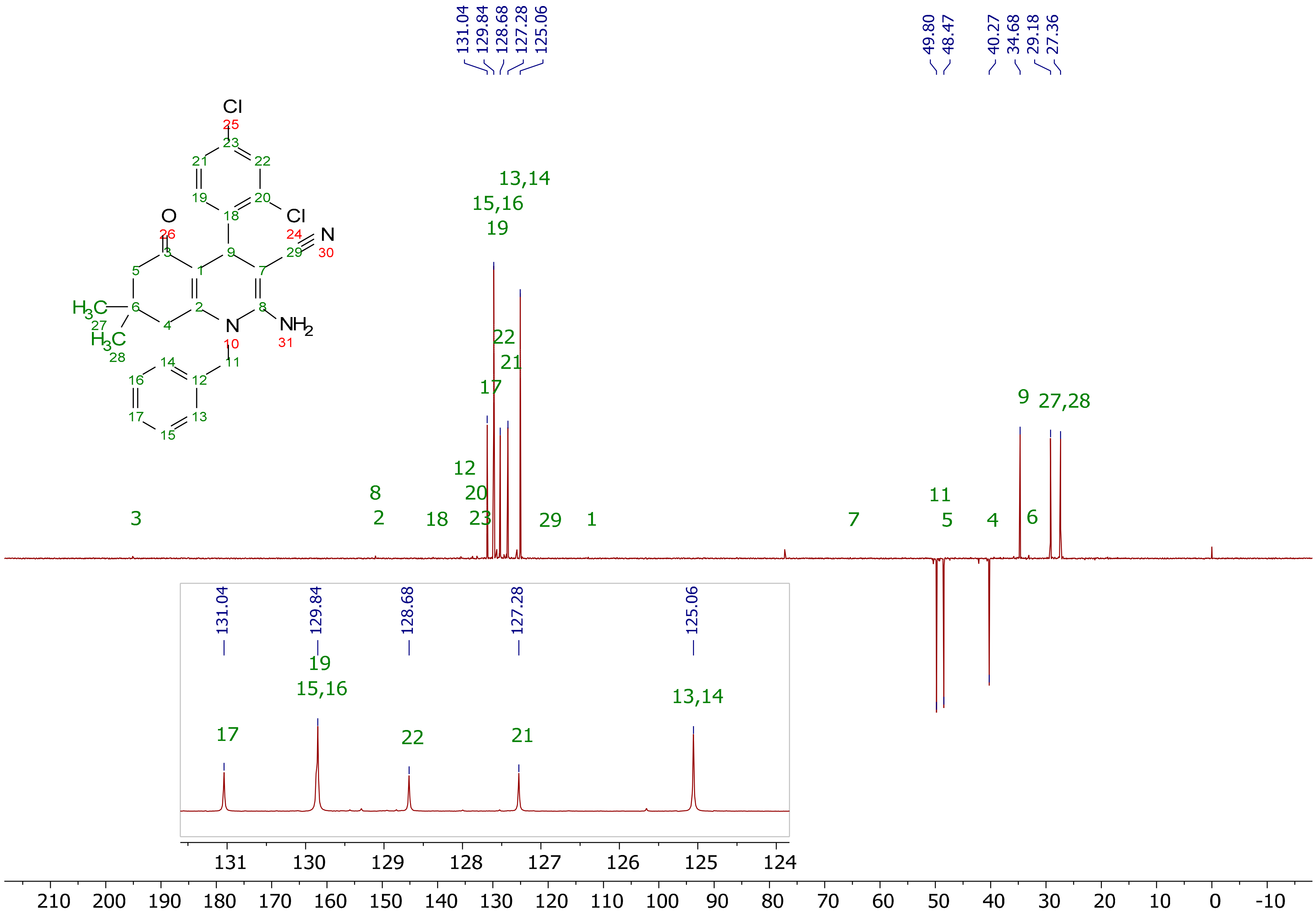

Figure S60 - DEPT spectrum of 5n

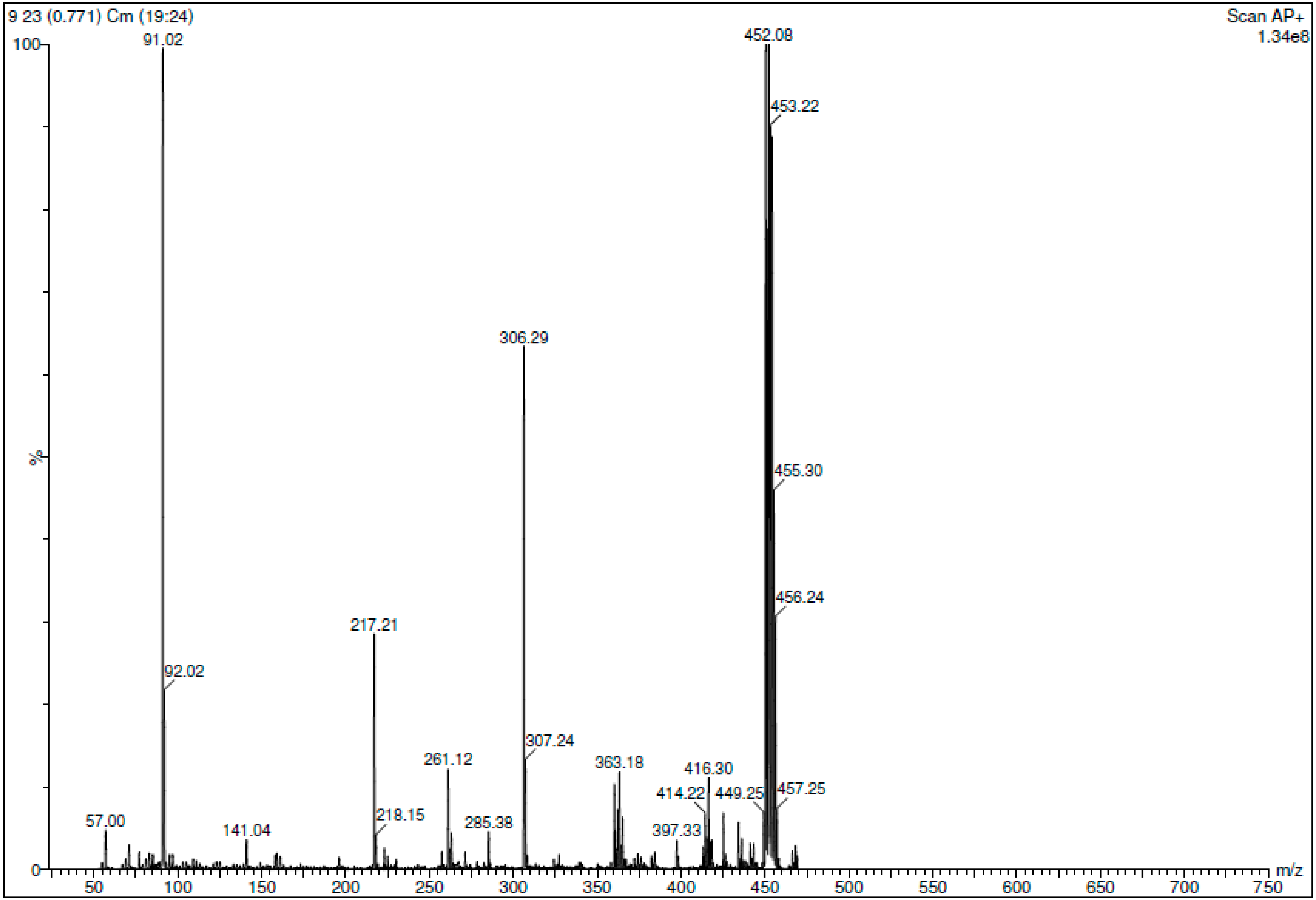

Figure S61 - MS spectrum of 5n

Table S7 - Fragmentation positions for peaks in MS spectrum of 5n

m/z	Fragmentation position
**452.08 – 457.25 (isotopes)**	$[M+H]^+$
**414.22-416.30 (isotopes)**	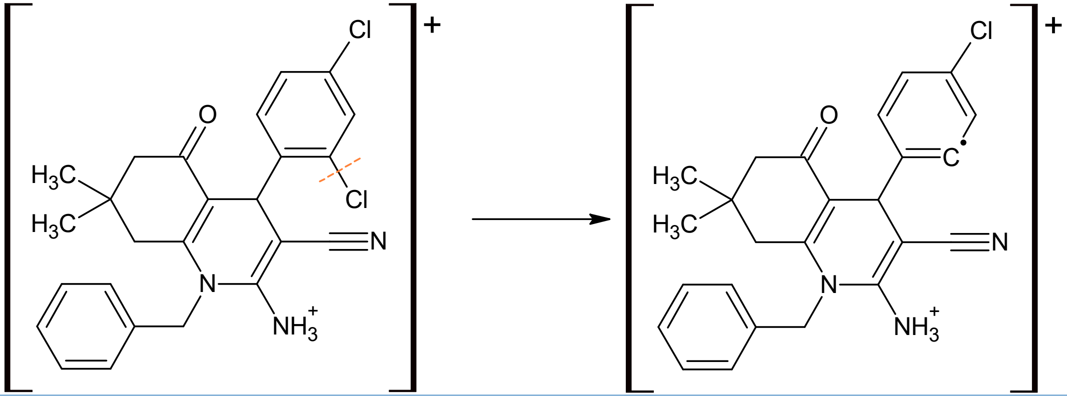
**361.18 - 363.18 (isotopes)**	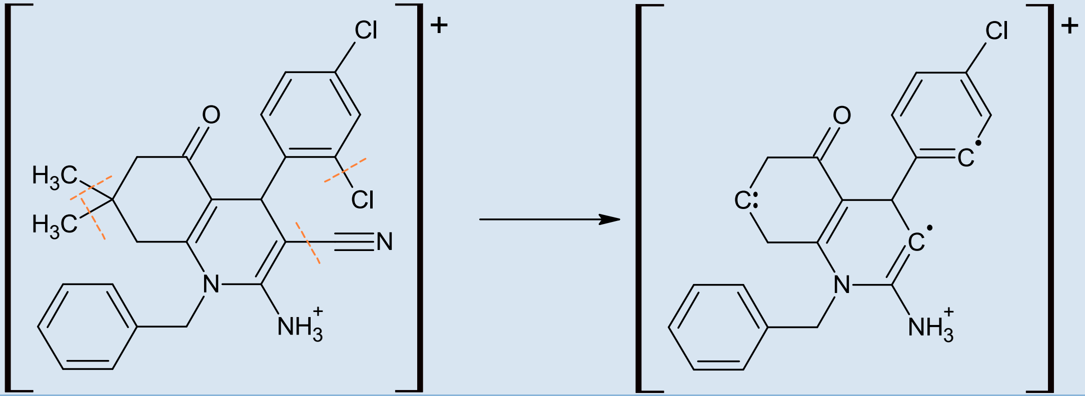
**285.38**	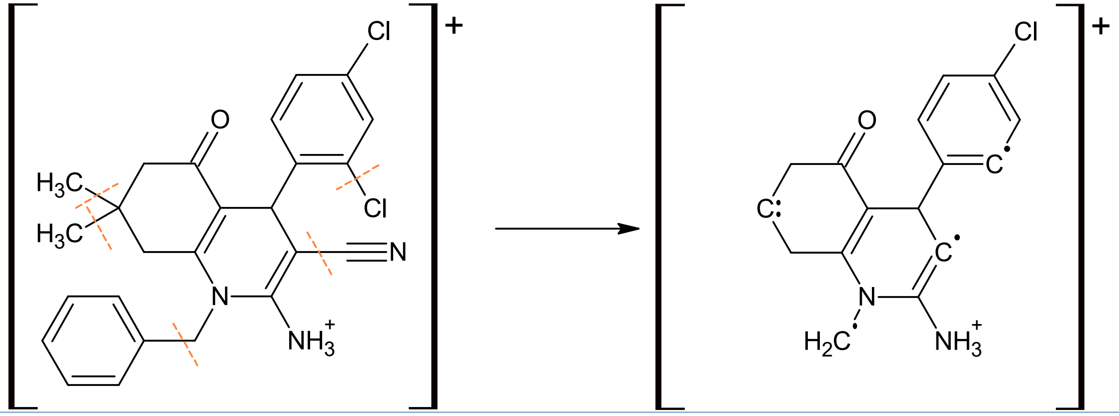

**218.15**	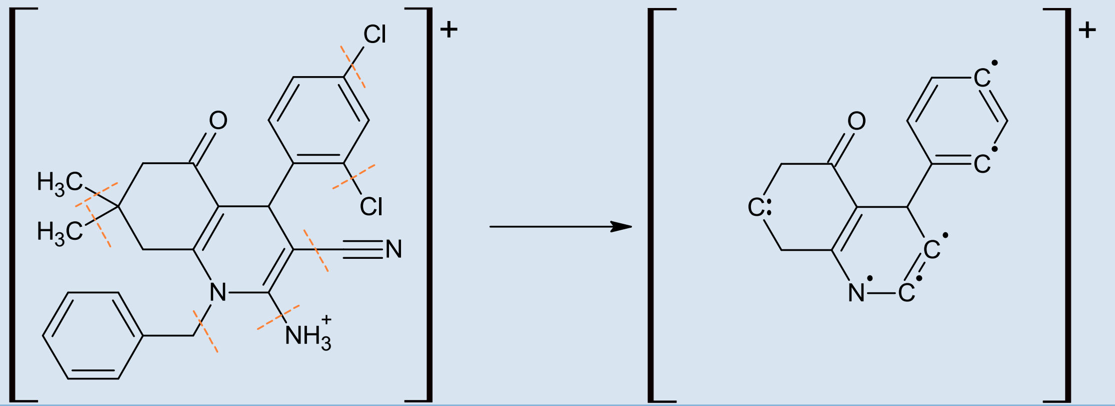
**91.02**	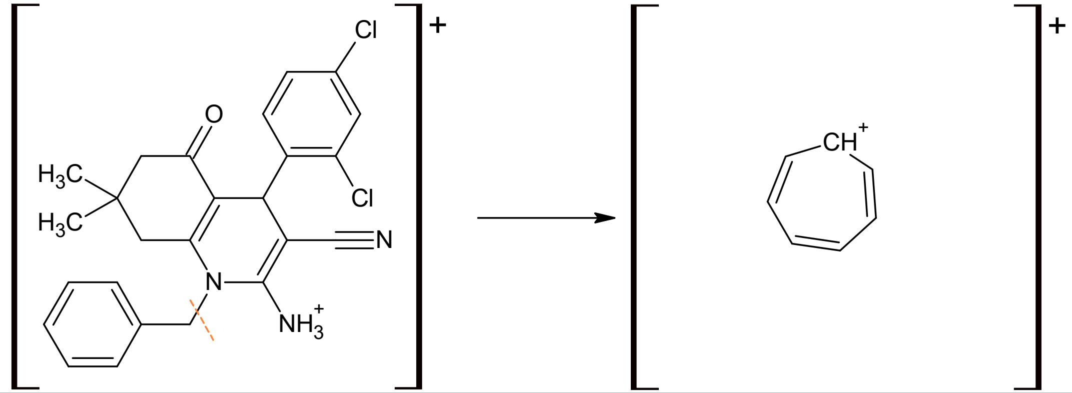

1.15. **Product 5o: 2-amino-1-cyclohexyl-7,8-dimethyl-5-oxo-4-(3-nitrophenyl)-1,4,5,6,7,8-hexahydroquinoline-3-carbonitrile**

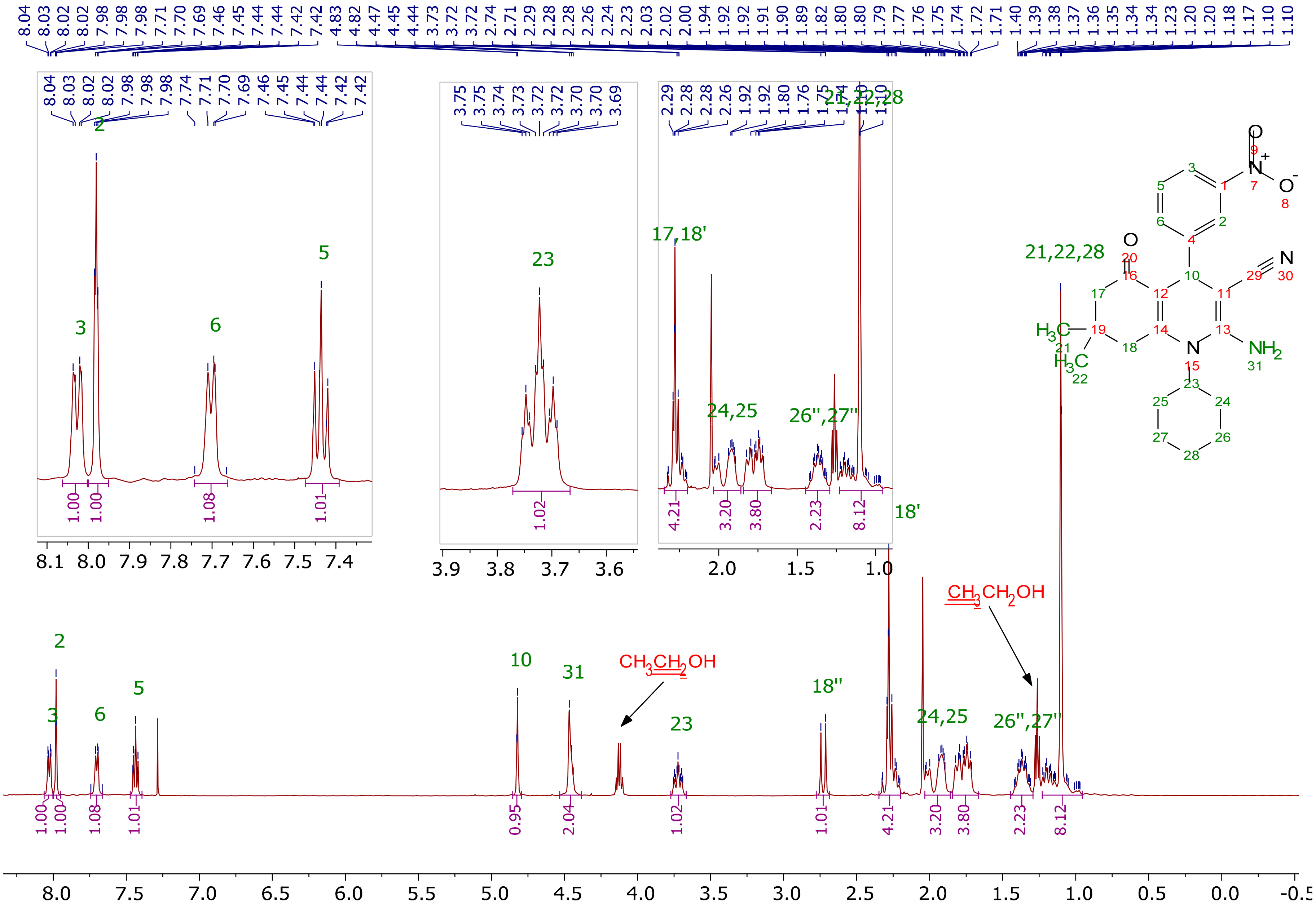

Figure S62 - ^{1}H NMR spectrum of 5o

1.16. Product 5p: 2-amino-4-(4-cyanophenyl)-7,8-dimethyl-1-(4-(phenylazo)phenyl)-5-oxo-1,4,5,6,7,8-hexahydroquinoline-3-carbonitrile

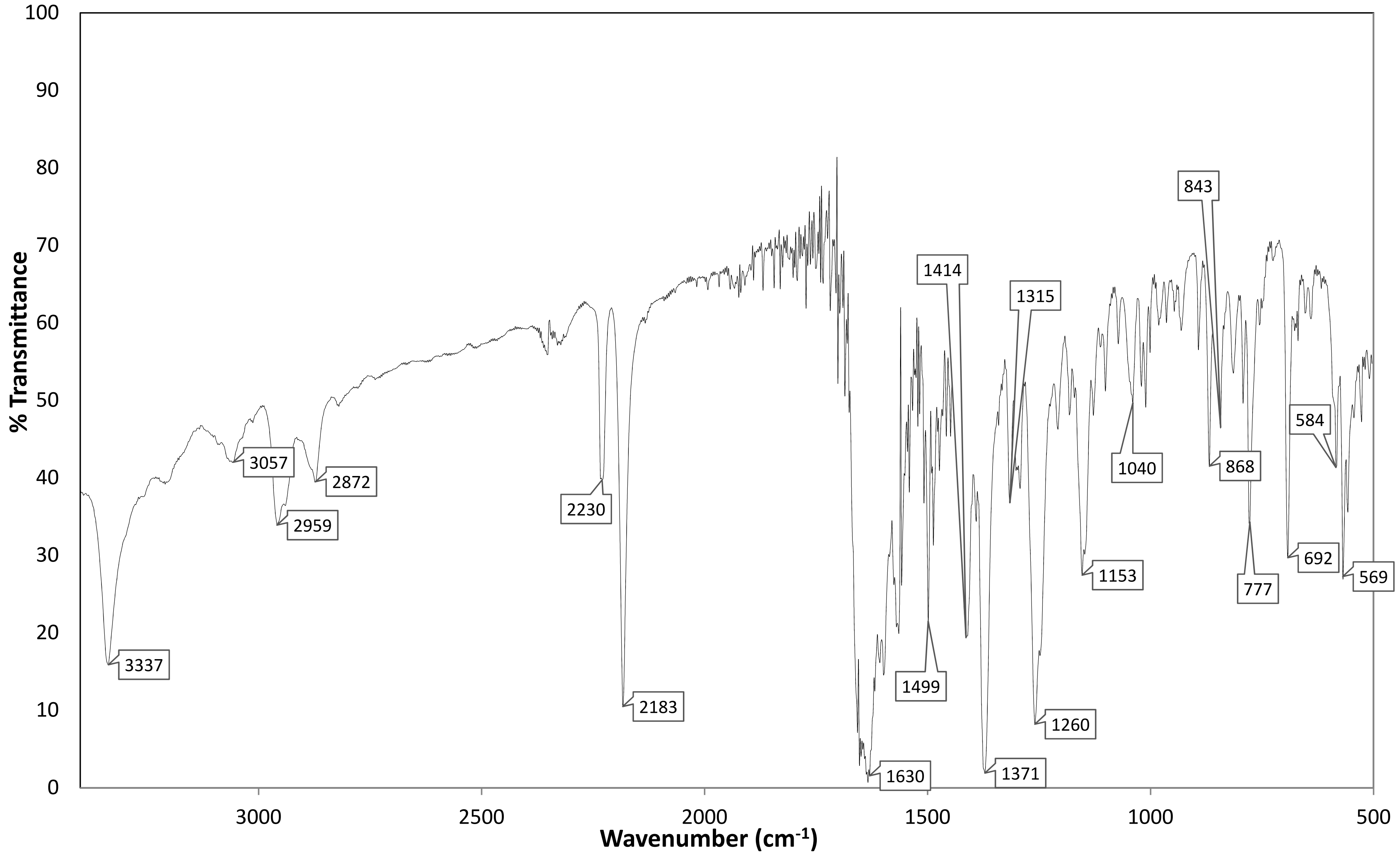

Figure S63 - IR spectrum of 5p

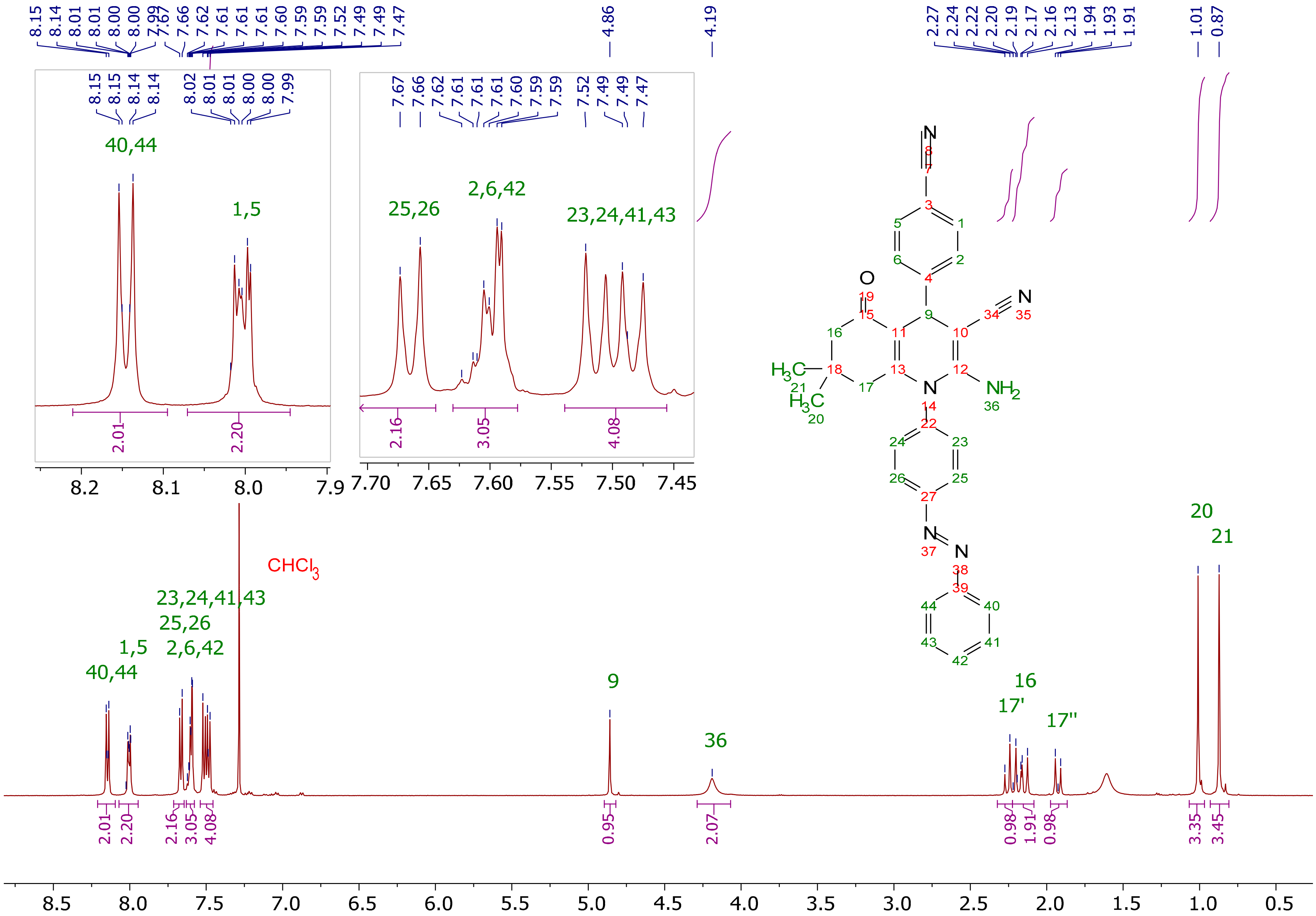

Figure S64 - ^{1}H NMR spectrum of 5p

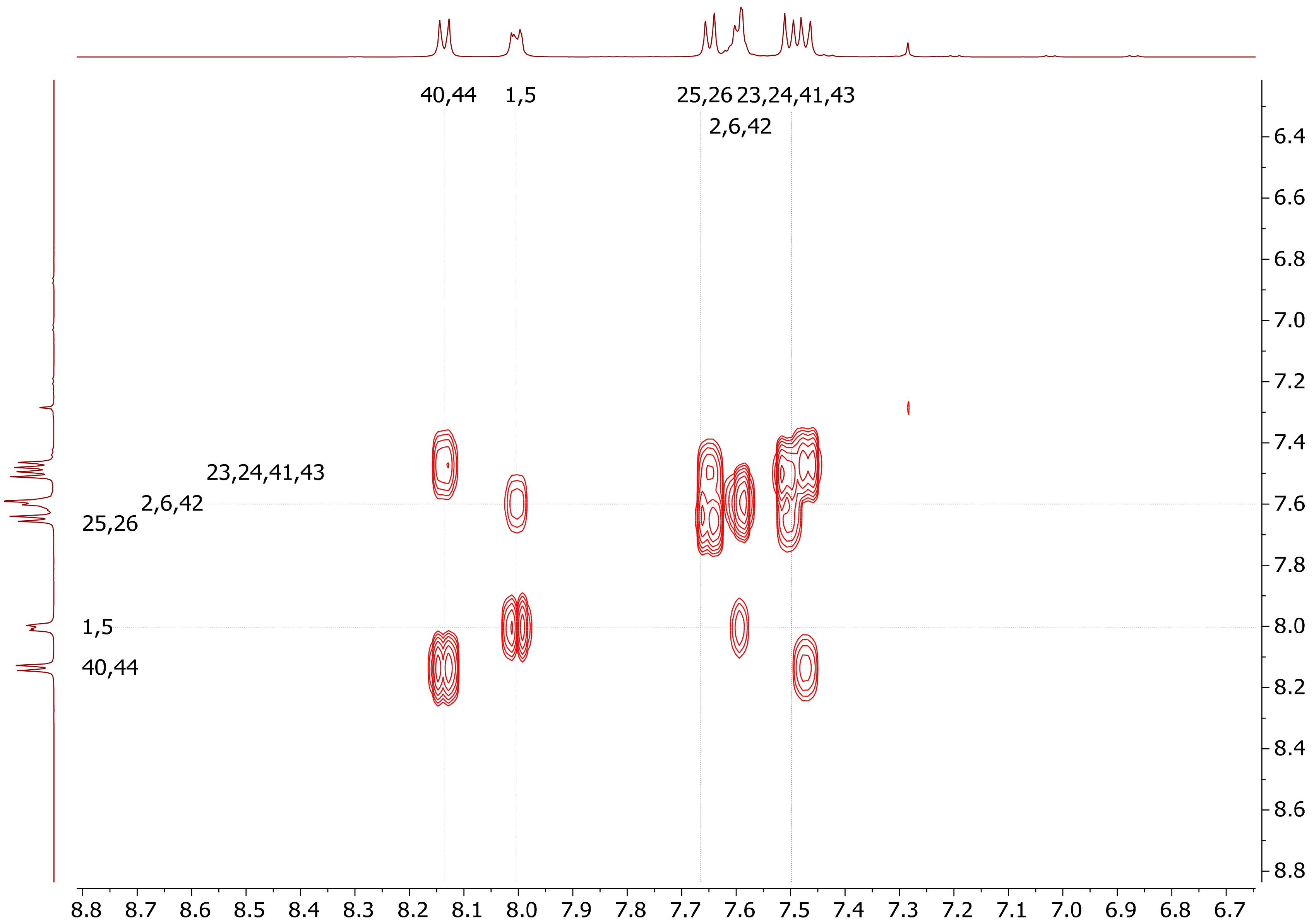

Figure S65 - ^{1}H-^{1}H COSY of 5p

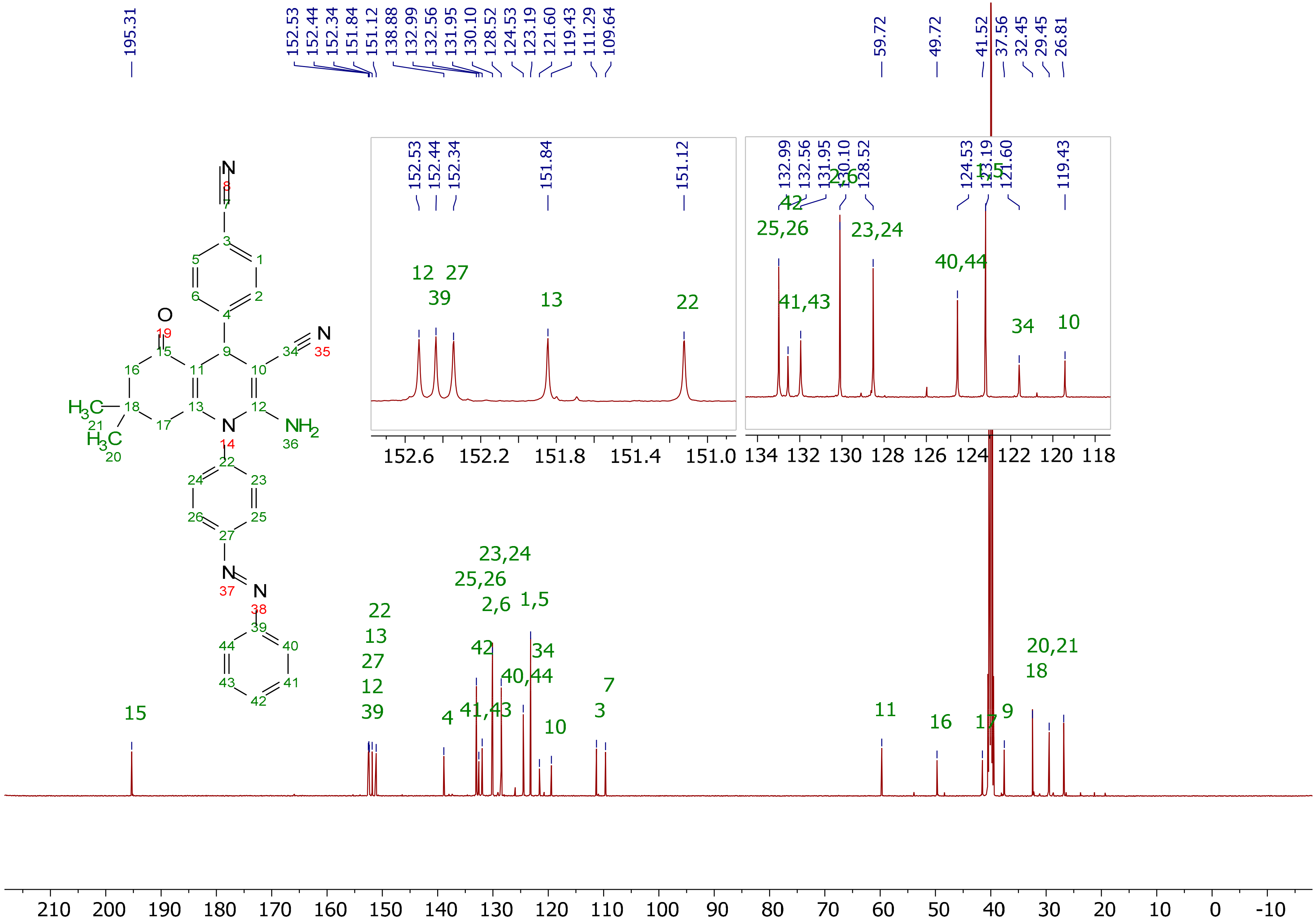

Figure S66 - ^{13}C NMR spectrum of 5p

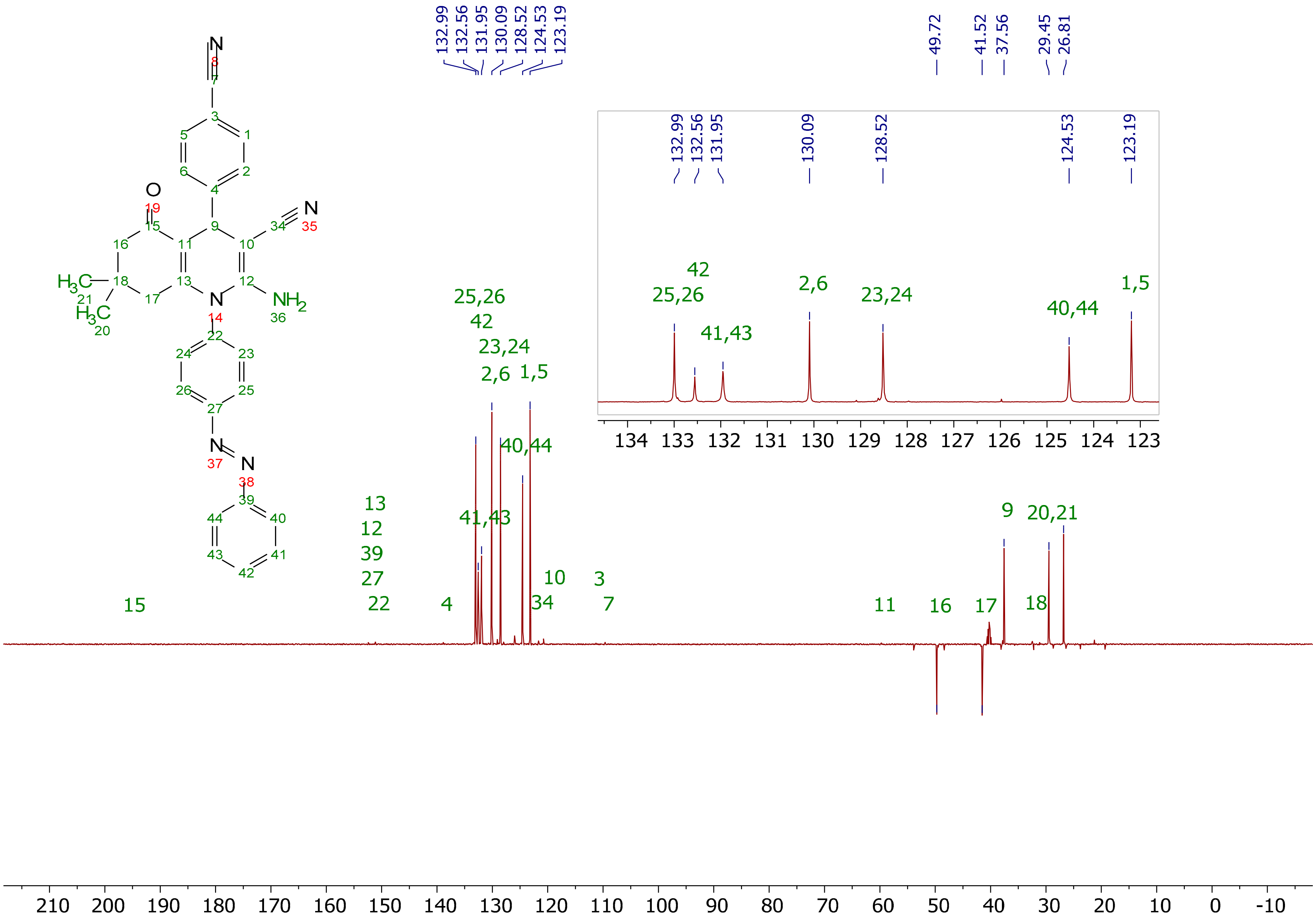

Figure S67 - DEPT spectrum of 5p

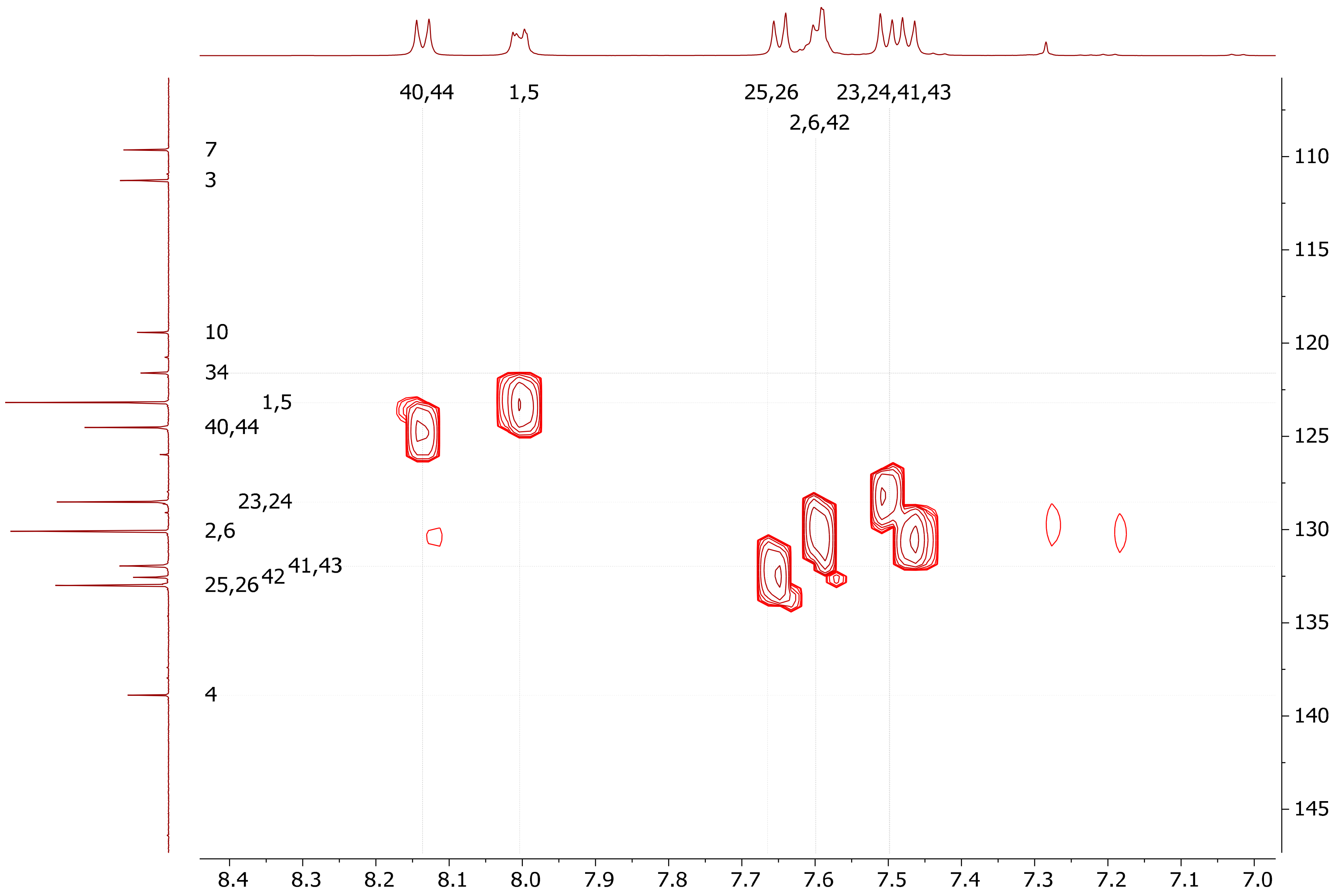

Figure S68 - HSQC spectrum of 5p

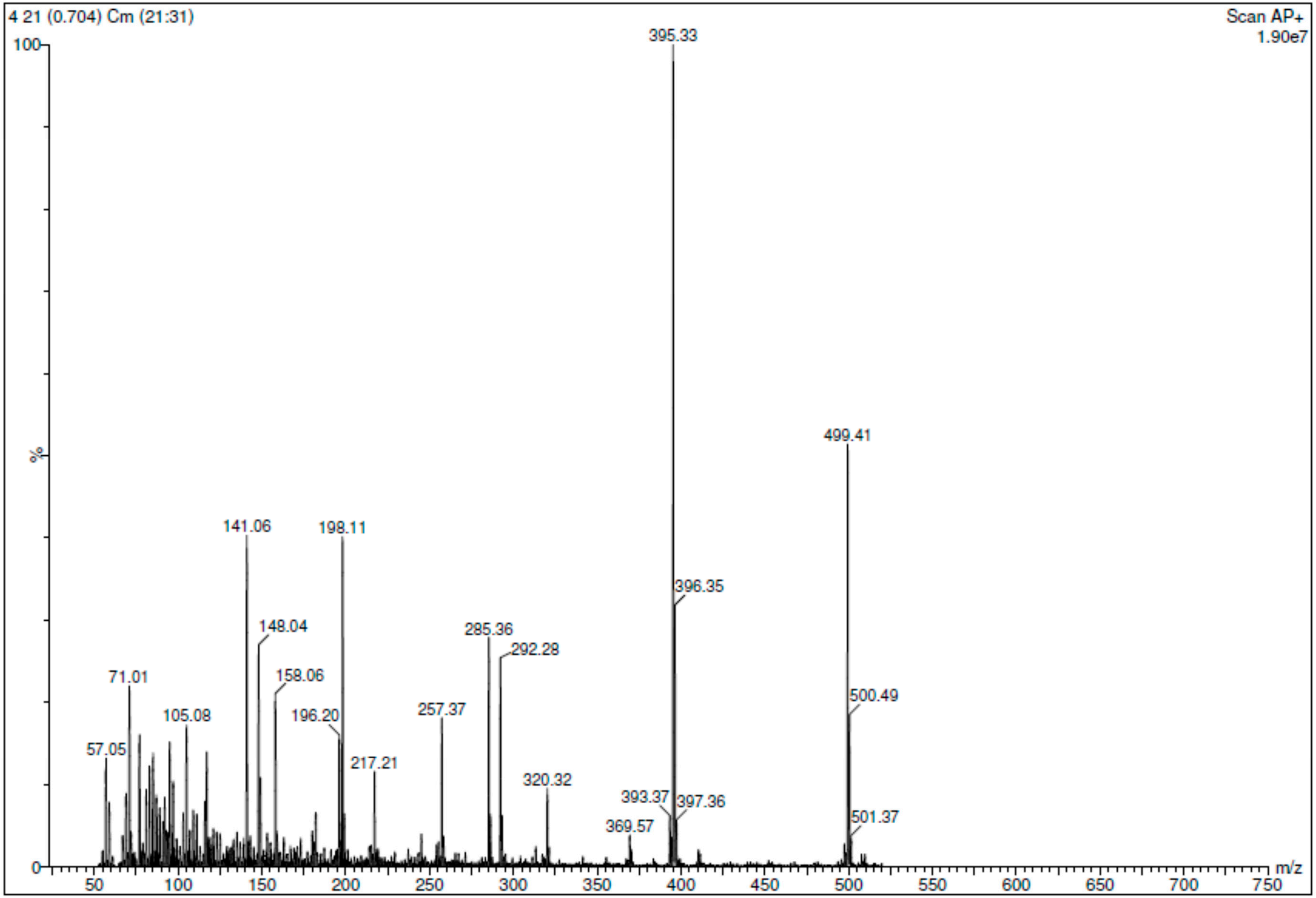

Figure S69 - MS spectrum of 5p

Table S8 - Fragmentation positions for peaks in MS spectrum of 5p

m/z	Fragmentation position
**499.41**	$[M+H]^+$
**395.33**	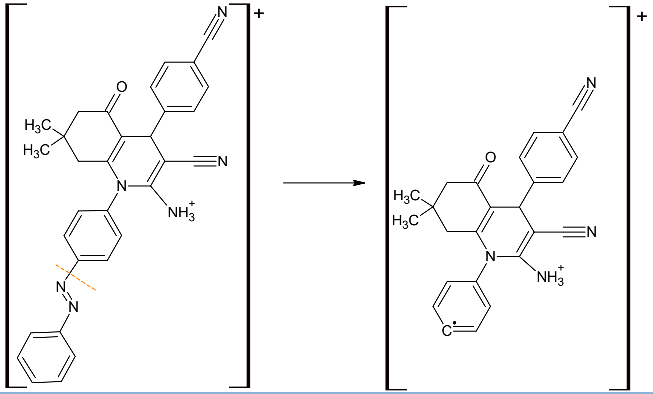
**292.28**	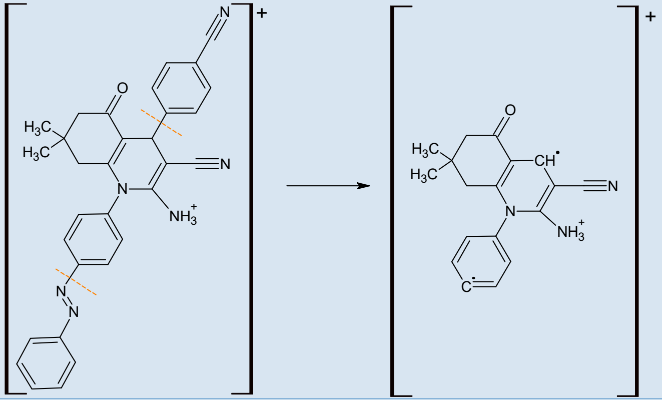
**217.21**	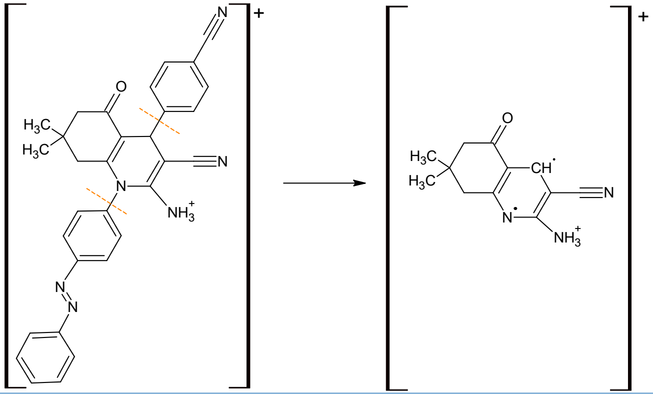

1.17. Product 5q: 2-amino-4-(2,4-dichlorophenyl)-7,8-dimethyl-1-(4-(3-methylphenylazo)-3-methylphenyl)-5-oxo-1,4,5,6,7,8-hexahydroquinoline-3-carbonitrile

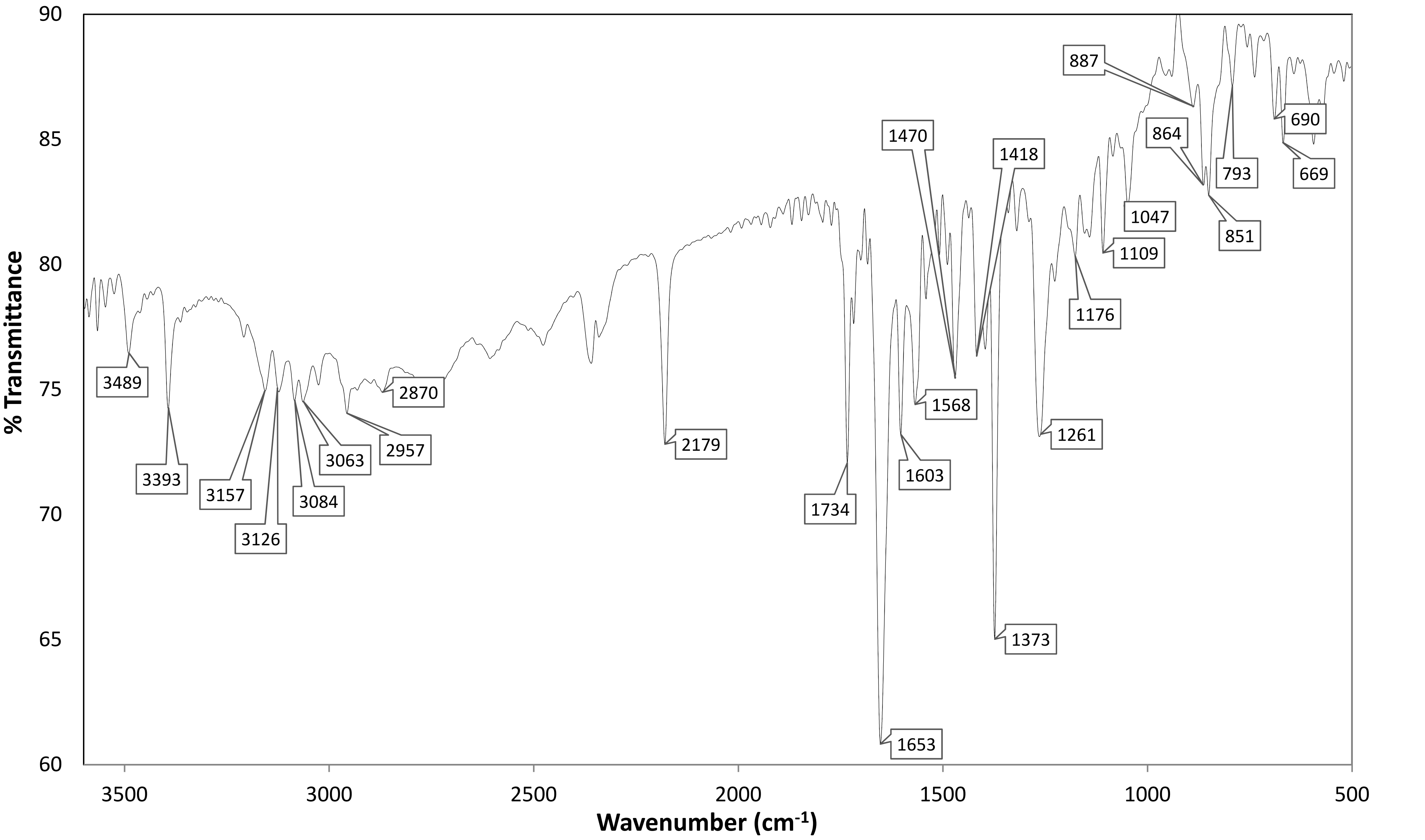

Figure S70 - IR spectrum of 29q

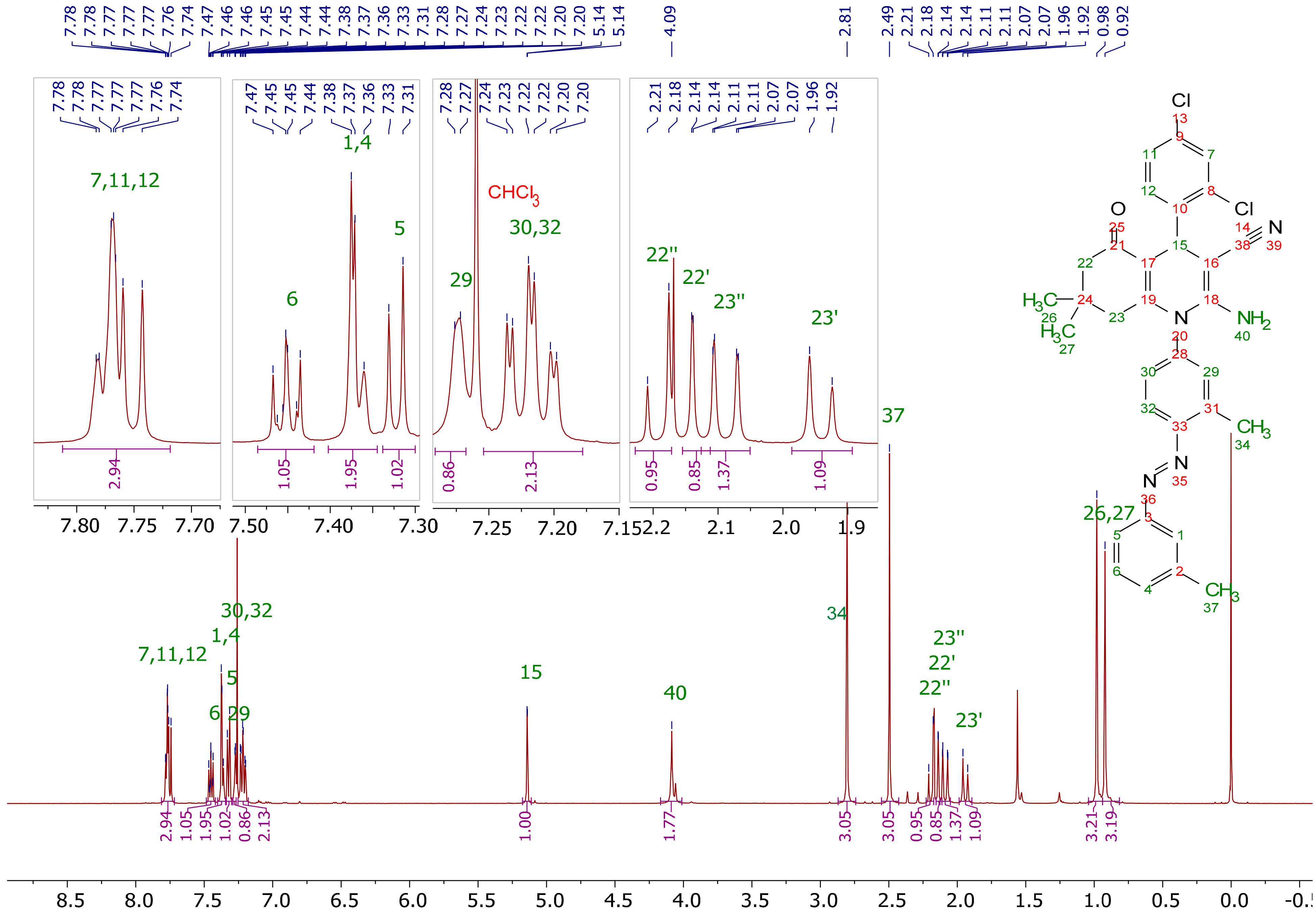

Figure S71 - ^{1}H NMR spectrum of 5q

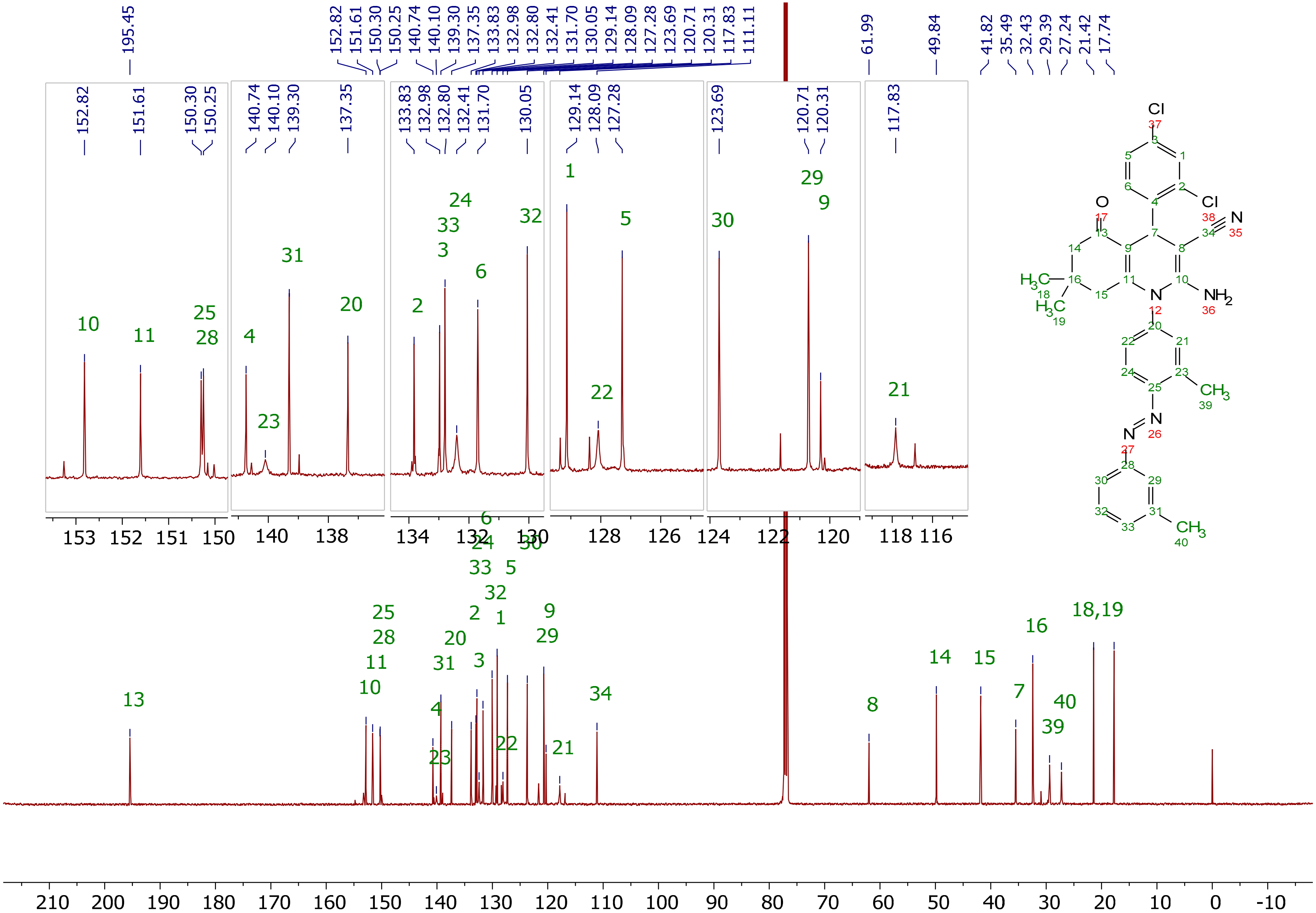

Figure S72 - ^{13}C NMR spectrum of 5q

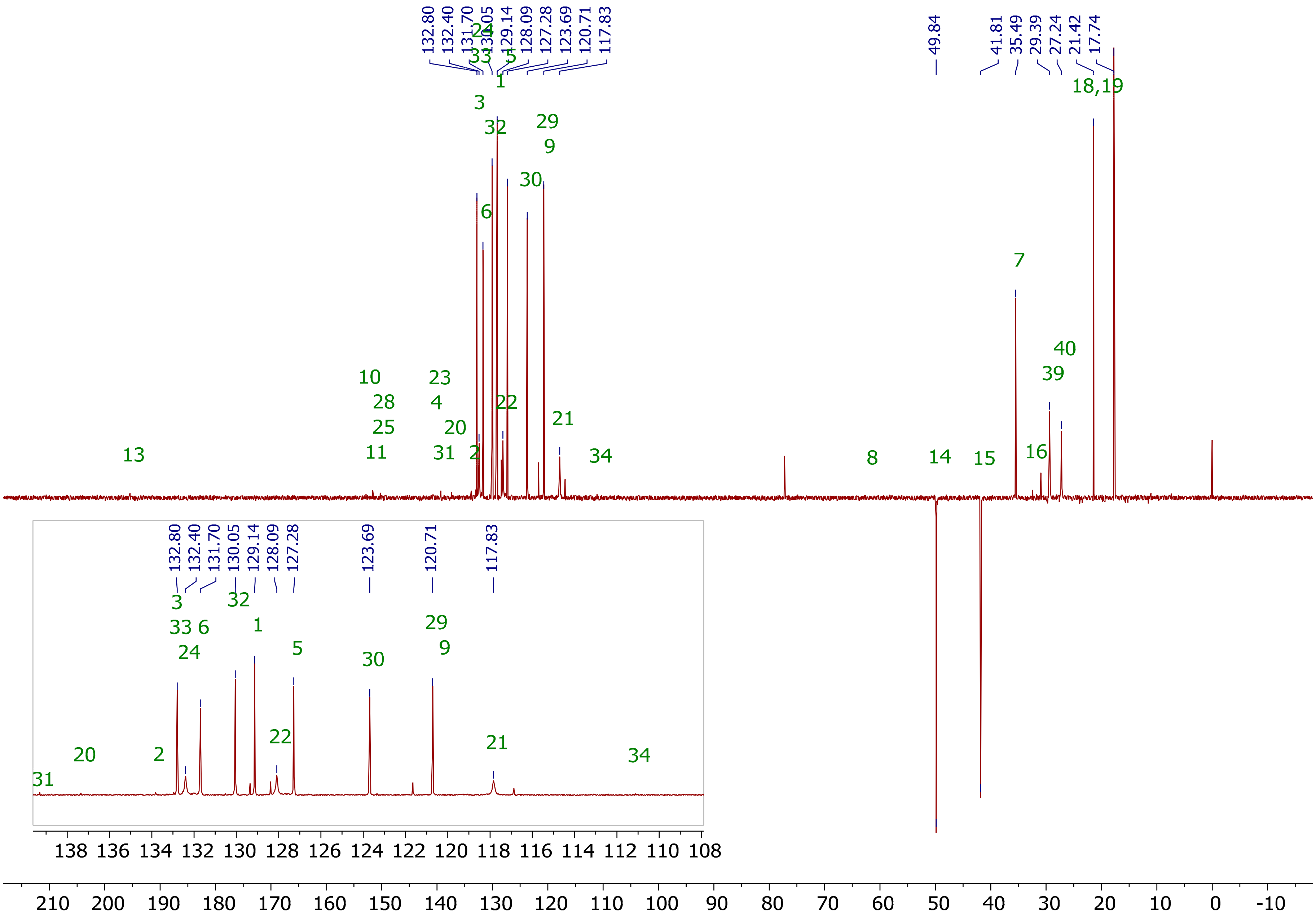

Figure S73 - DEPT spectrum of 5q

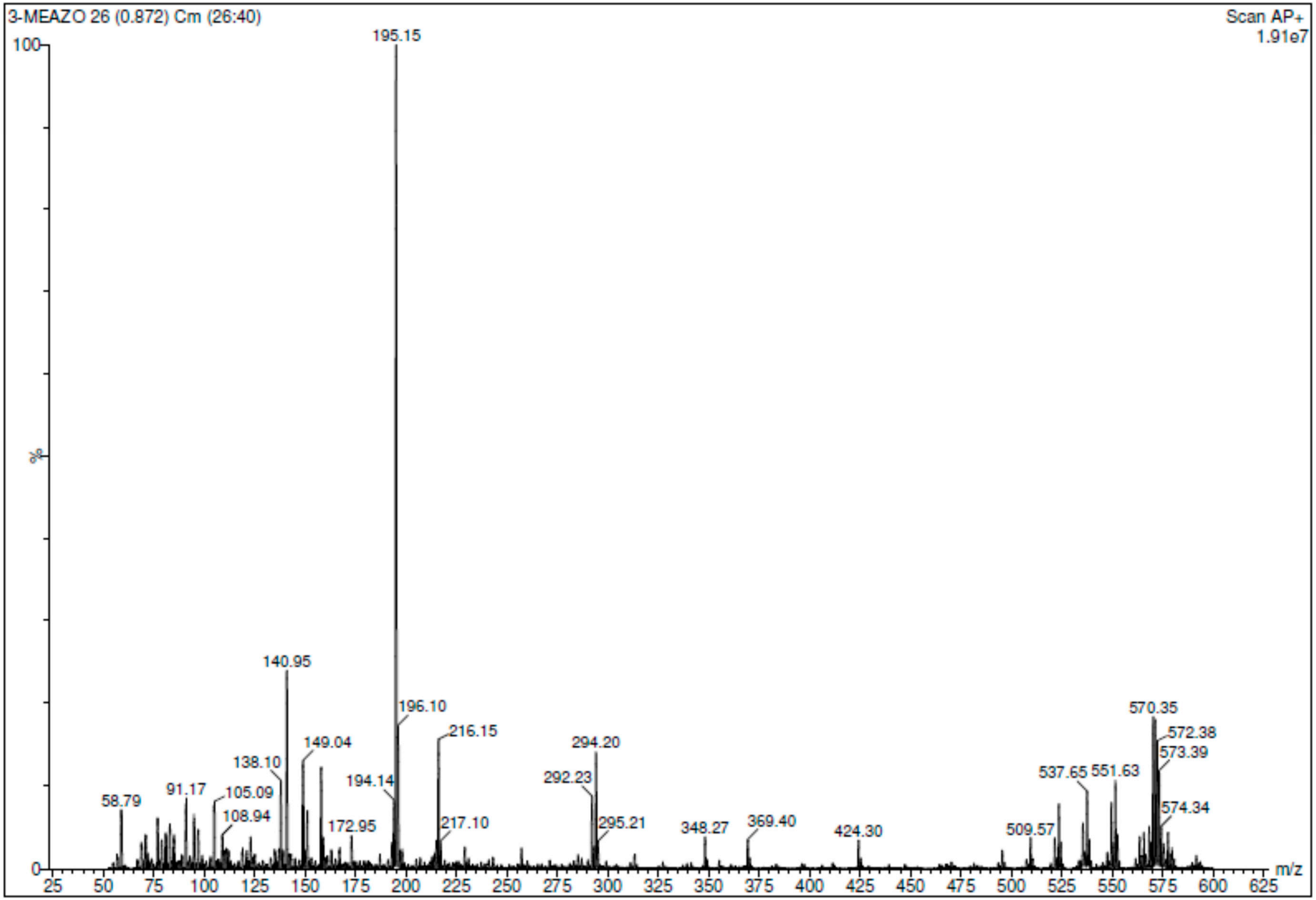

Figure S74 - MS spectrum of 5q

Table S9 - Fragmentation positions for peaks in MS spectrum of 29q

m/z	Fragmentation position	m/z	Fragmentation position
**570.35 – 574.34**	$[M+H]^+$ (isotopes)	**424.30**	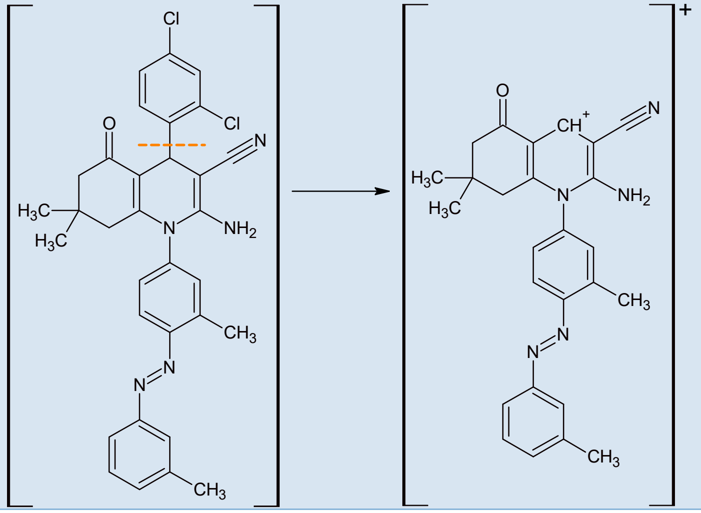
**537.65**	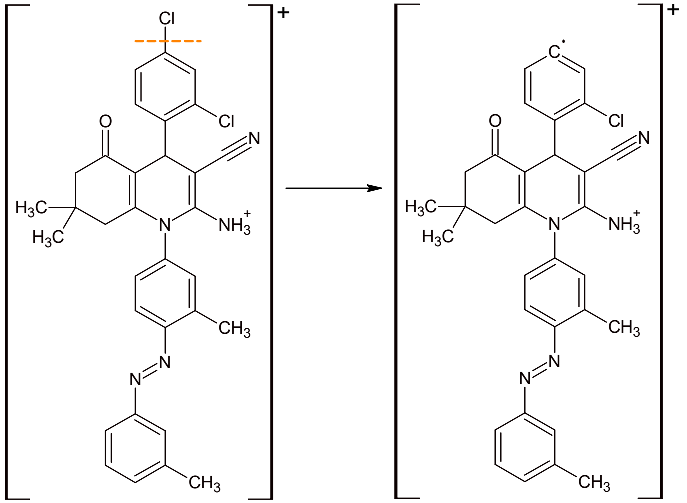	**216.15**	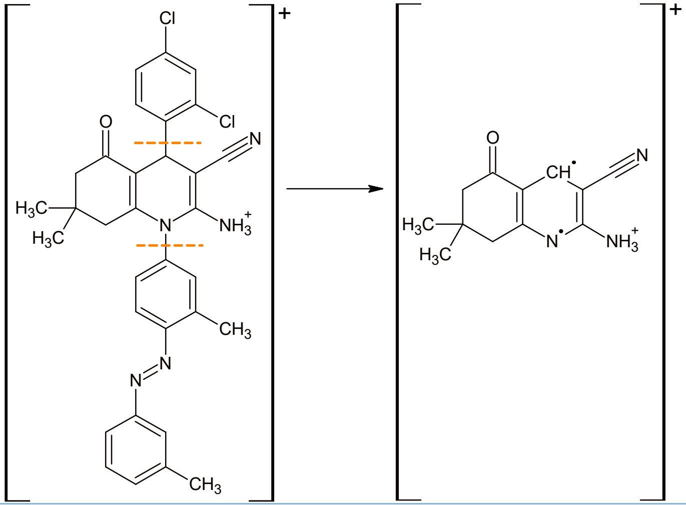

**195.15**	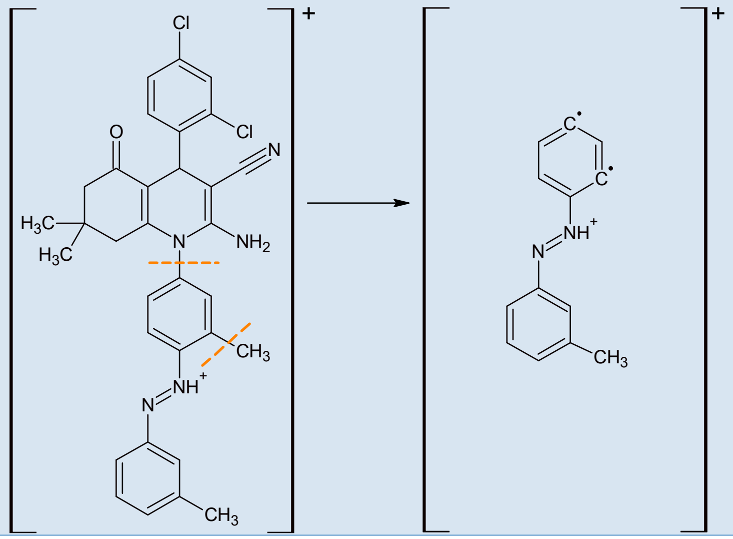 Cl<br>Cl<br>O<br>N<br>H3C<br>H3C<br>N<br>NH2<br>CH3<br>NH+<br>N<br>CH3<br>+<br>C<br>C<br>NH+<br>N<br>CH3<br>+			

1.18. Product 5s: 2-amino-4-(4-methylphenyl)-5-oxo-1-phenyl-1,4,5,6,7,8-hexahydroquinoline-3-carbonitrile

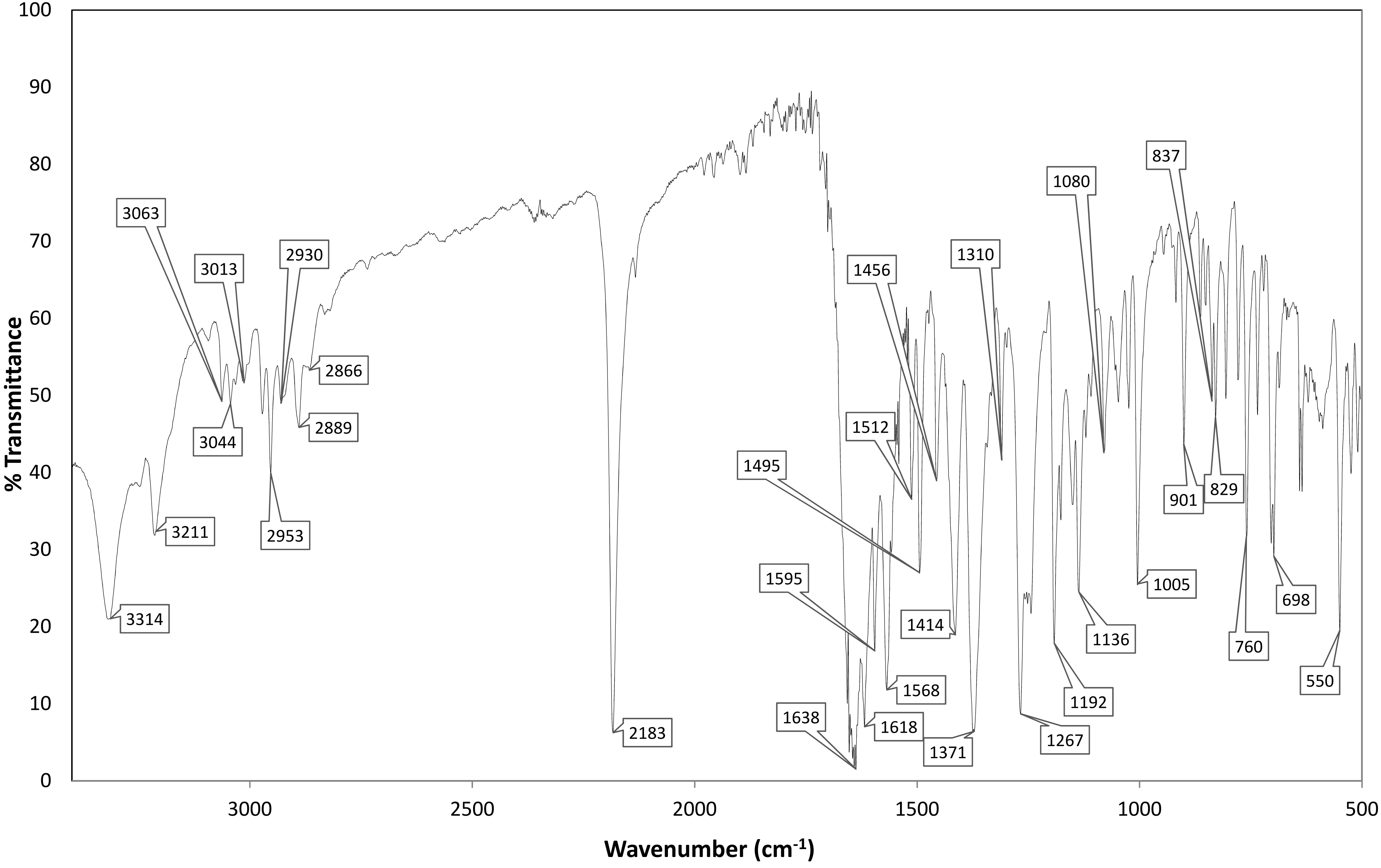

Figure S75 - IR spectrum of 5s

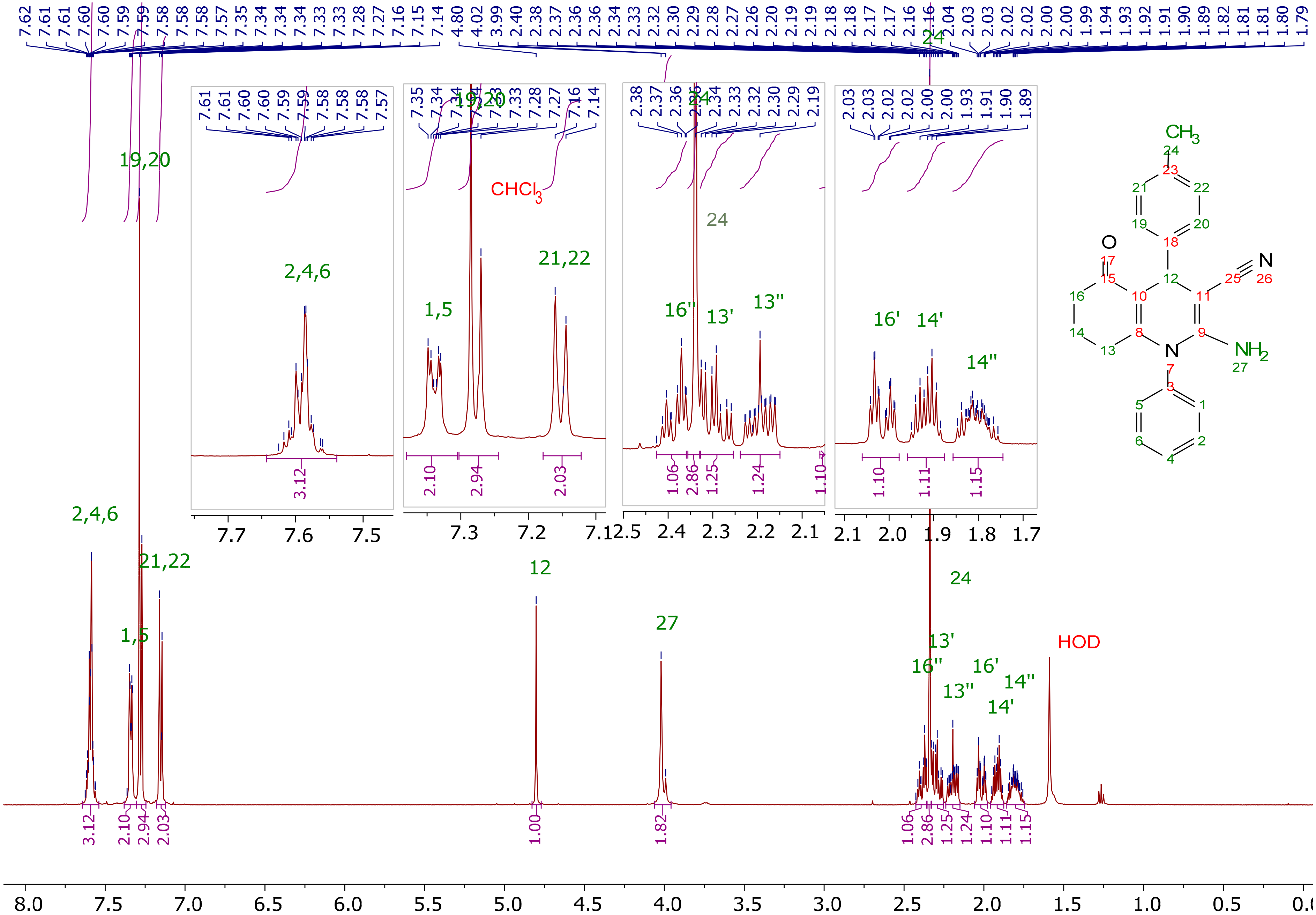

Figure S76 - ^{1}H NMR spectrum of 5s

1.19. Product 5t: 2-amino-4-(4-chlorophenyl)-5-oxo-1-phenyl-1,4,5,6,7,8-hexahydroquinoline-3-carbonitrile

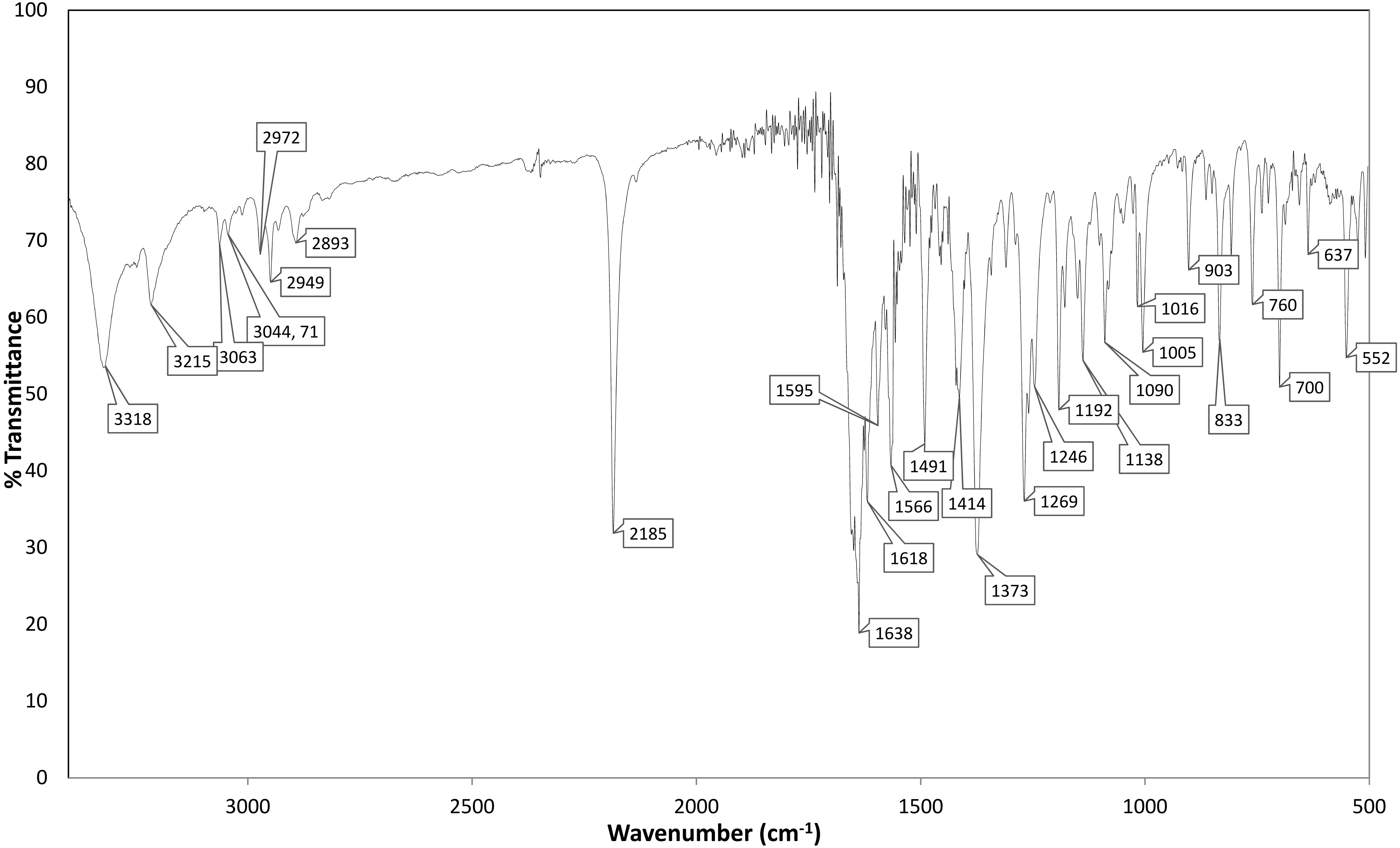

Figure S77 – IR spectrum of 5t

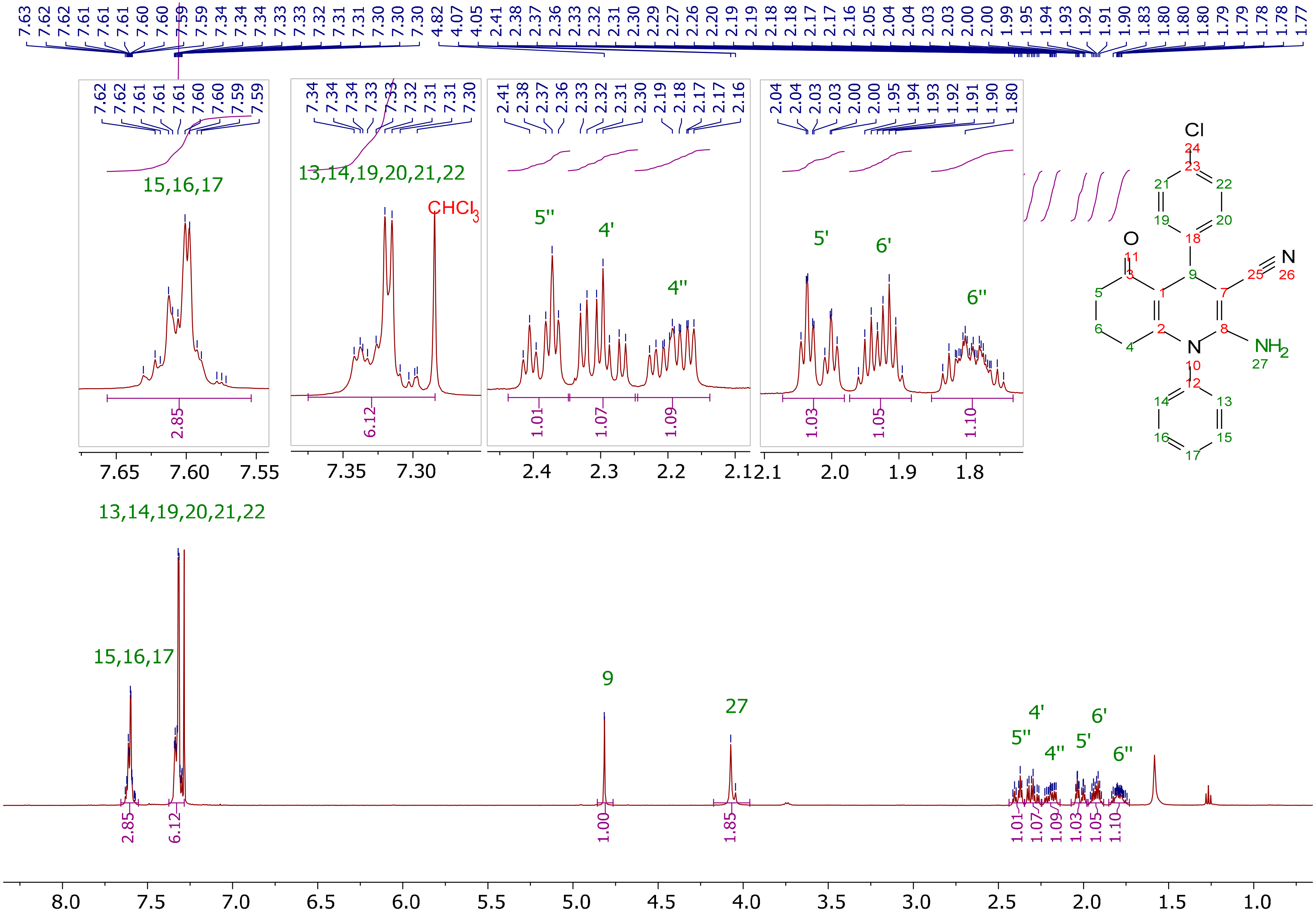

Figure S78 - ^{1}H NMR spectrum of 5t

1.20. Product 7a: 2′-Amino-7′,7′-dimethyl-2,5′-dioxo-1′-phenyl-5′,6′,7′,8′-tetrahydro-1*H*-spiro[indoline-3,4′-quinoline]-3′-carbonitrile

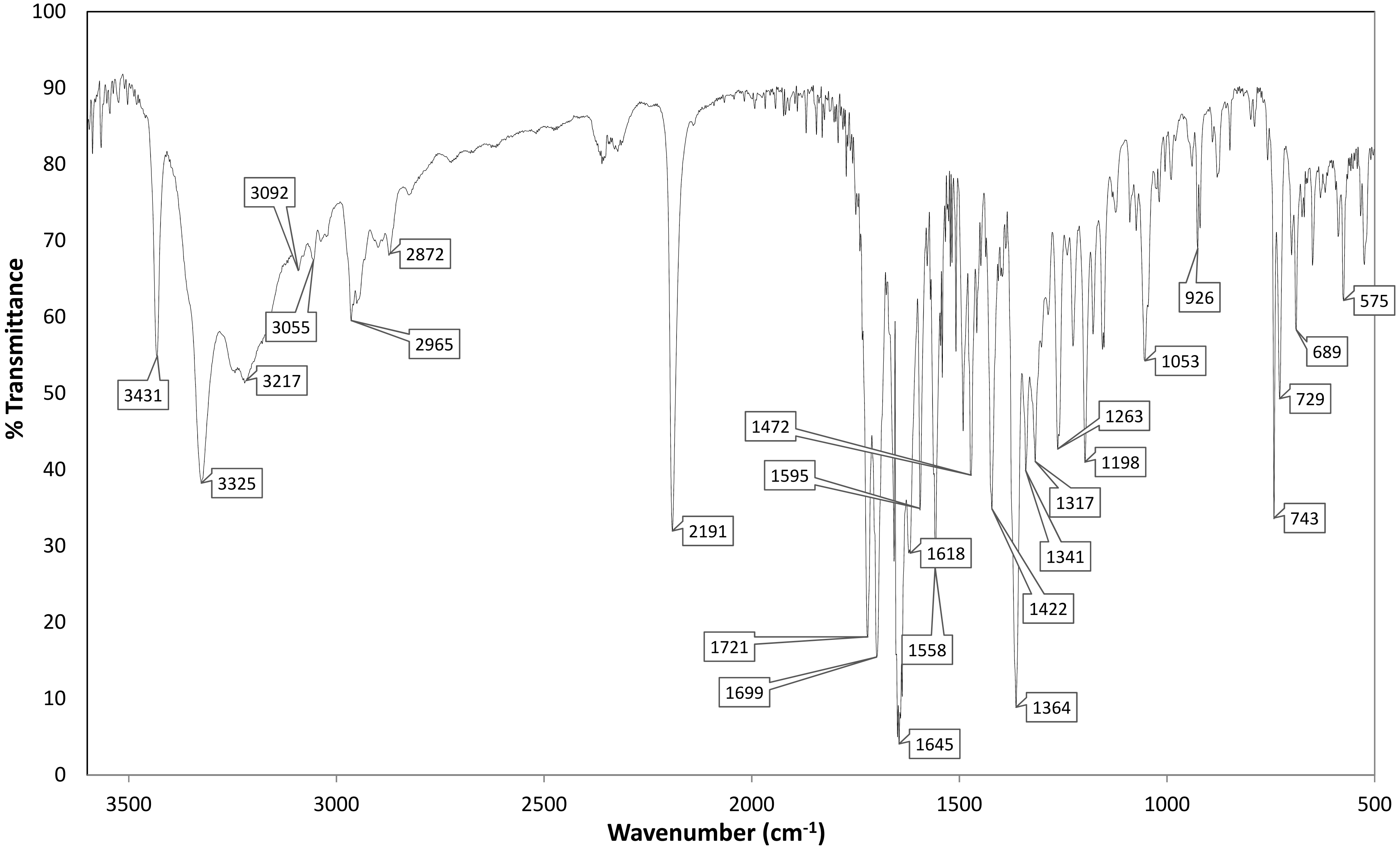

Figure S79 - IR spectrum of 7a

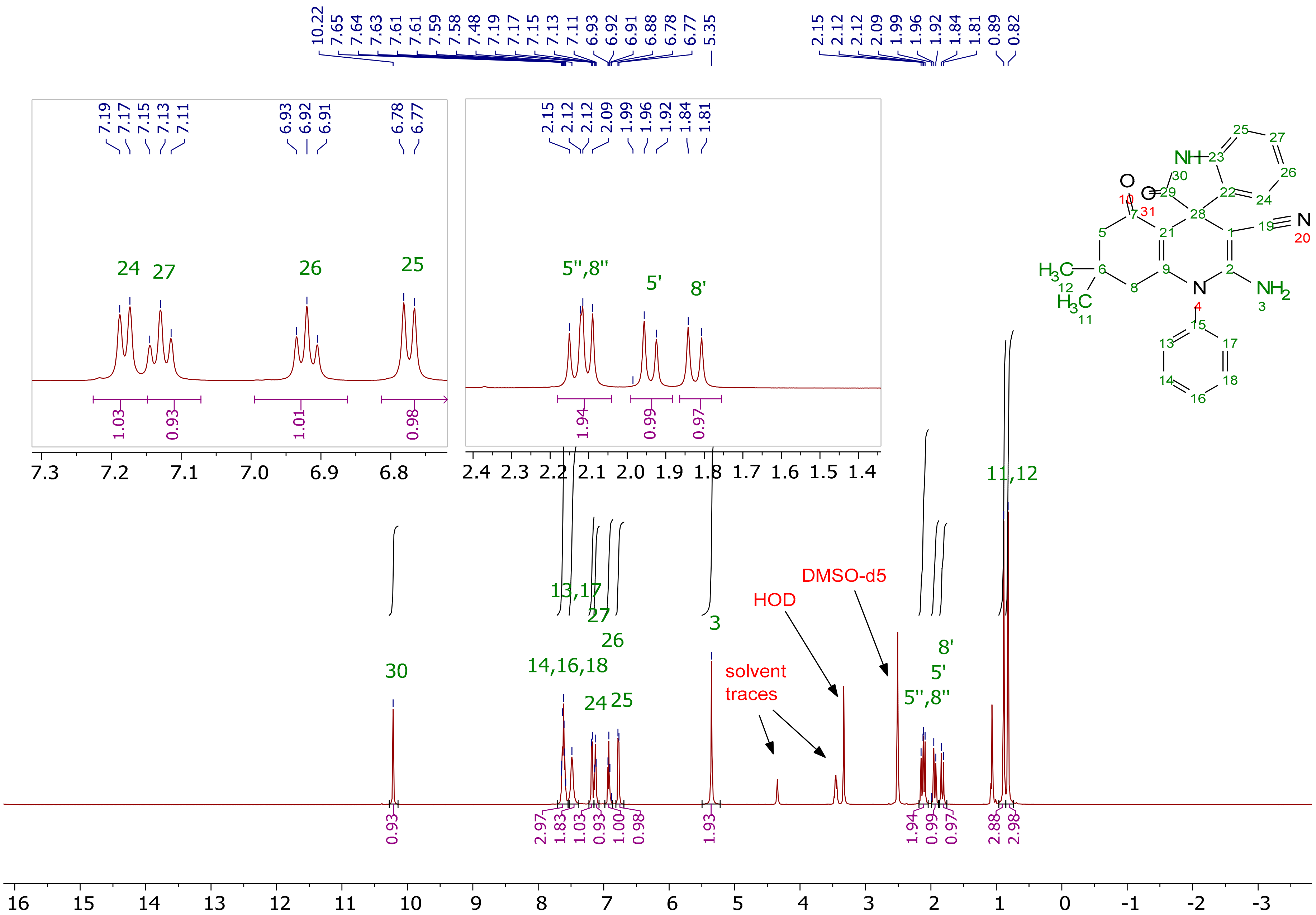

Figure S80 - ^{1}H NMR spectrum of 7a

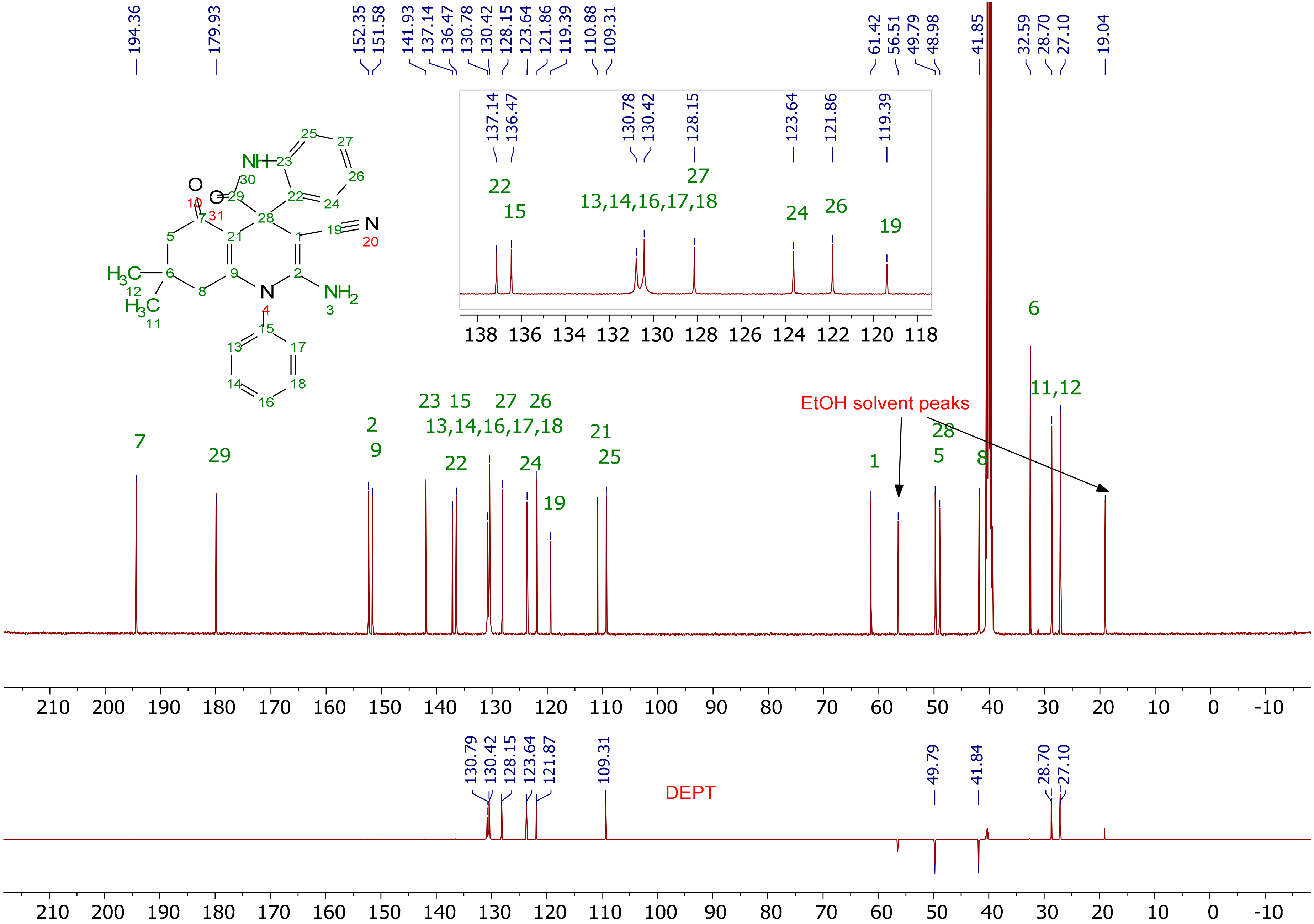

Figure S81 - ^{13}C NMR and DEPT spectra of 7a

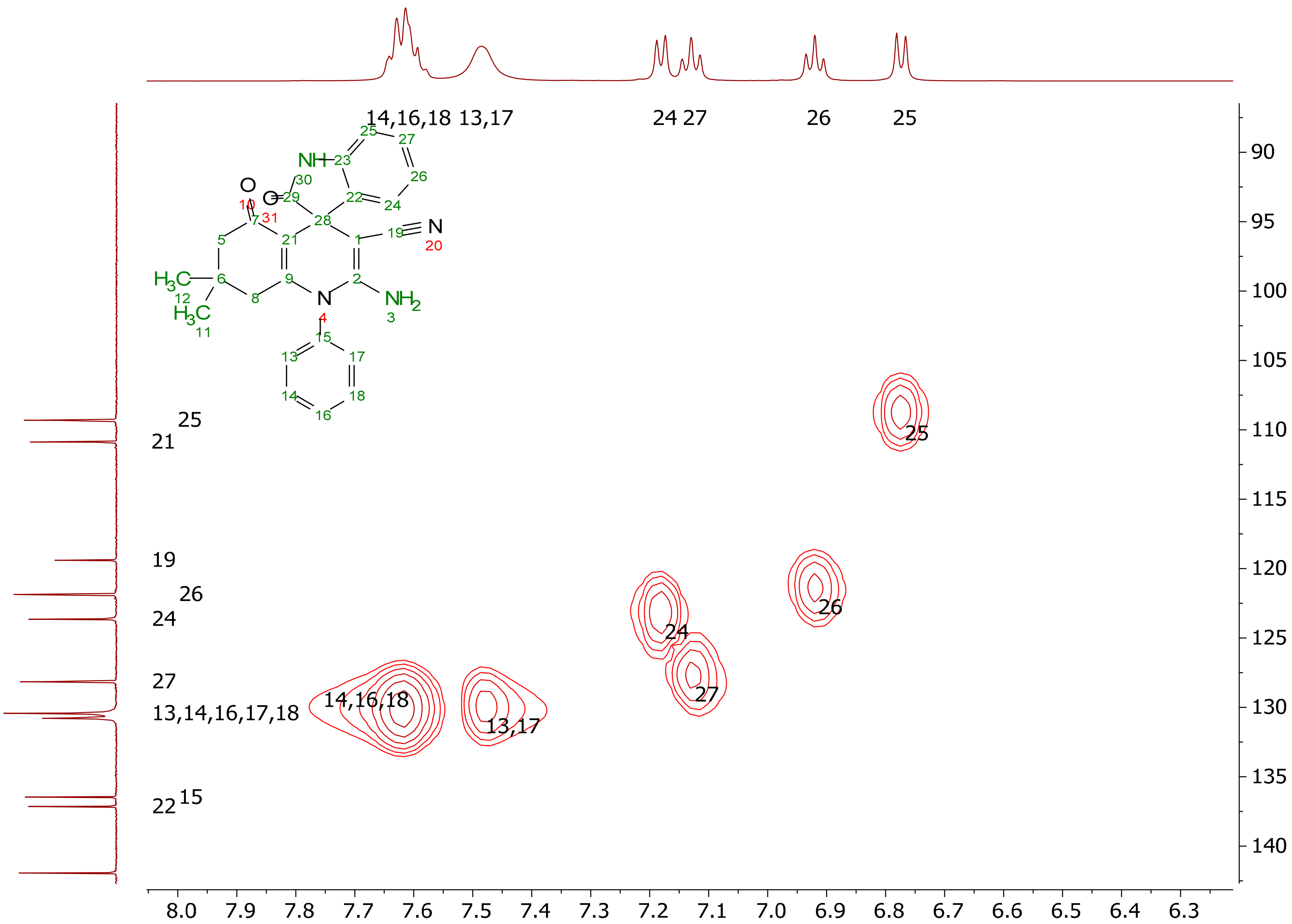

Figure S82 - HSQC spectrum of 7a

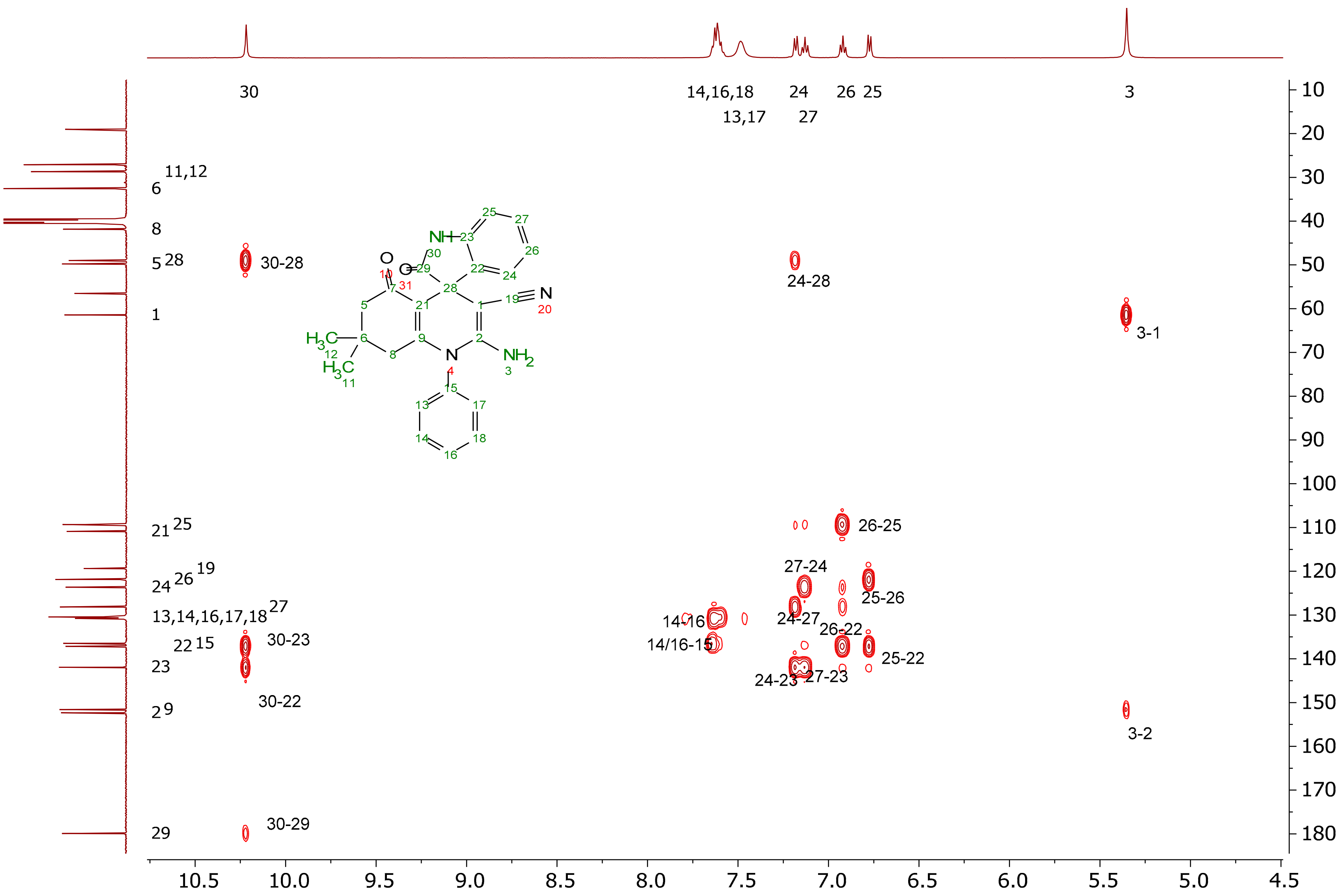

Figure S83 - Downfield region of HMBC spectrum of 7a

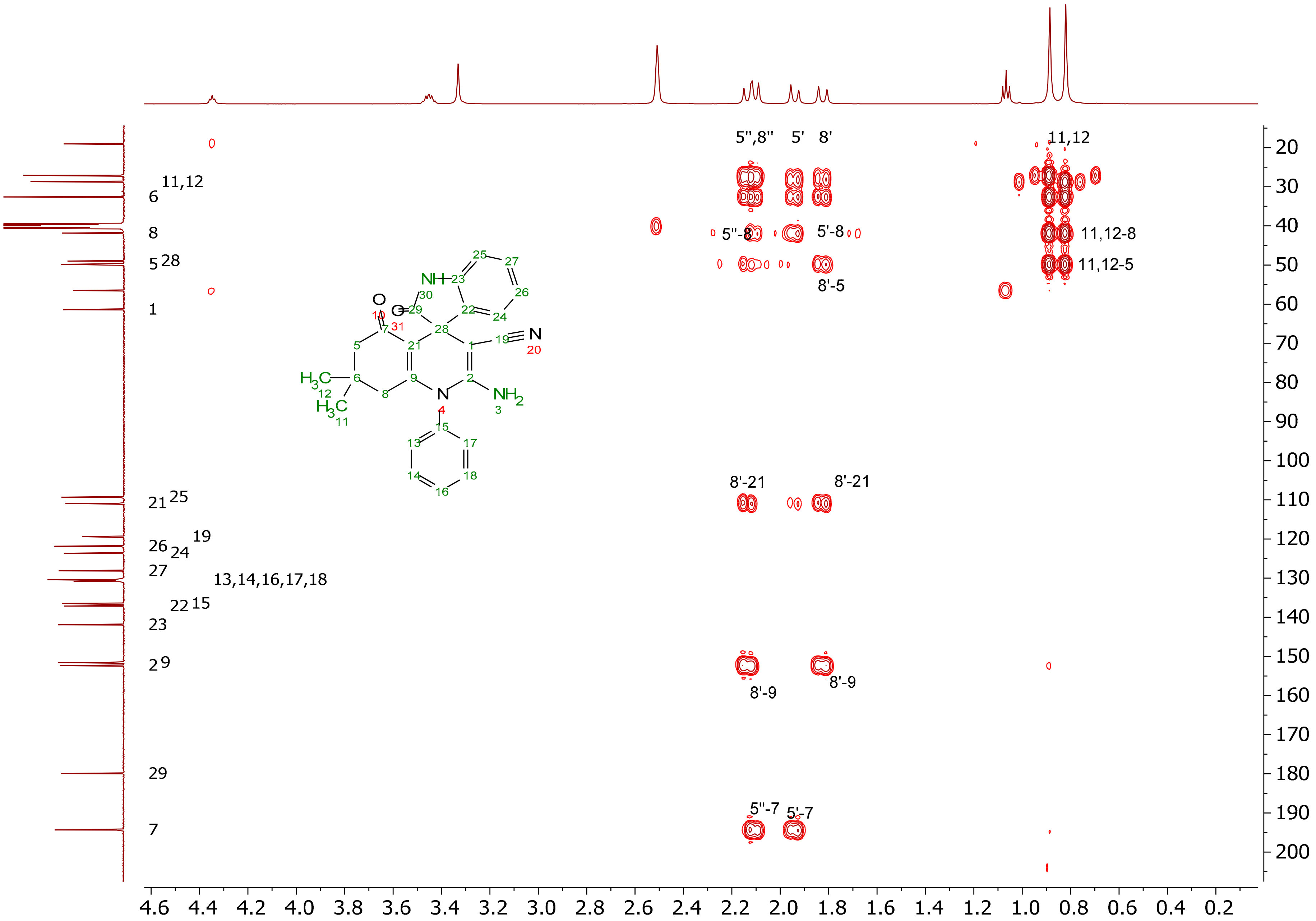

Figure S84 - Upfield region of HMBC spectrum of 7a

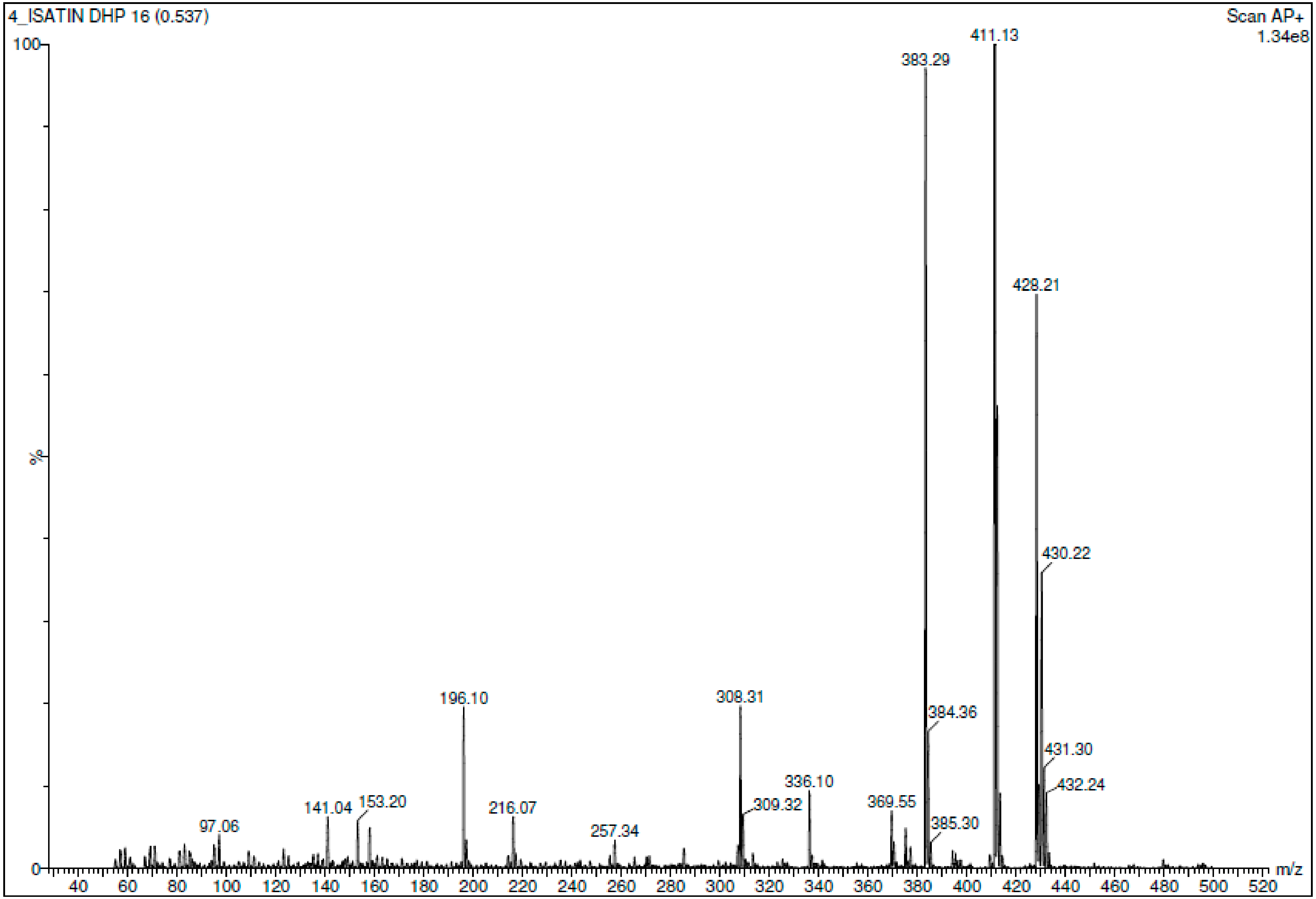

Figure S85 - MS spectrum of 7a

Table S10 - Peaks and fragmentation positions for MS spectrum of 7a

m/z	Fragmentation position and structure
**428.21**	$[M+H_2O]^+$
**411.13**	$[M+H]^+$
**384.36**	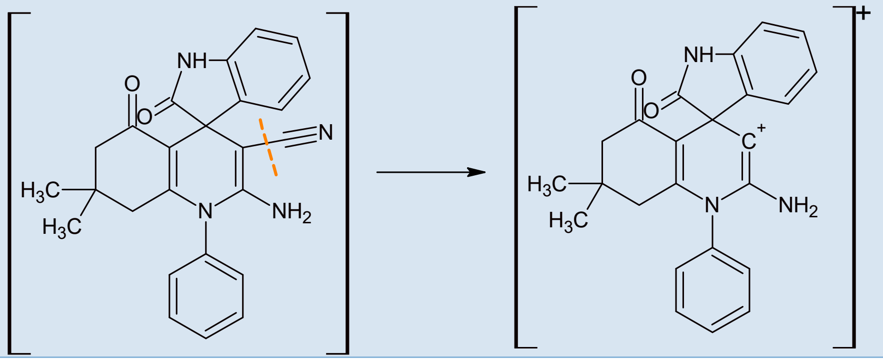
**308.31**	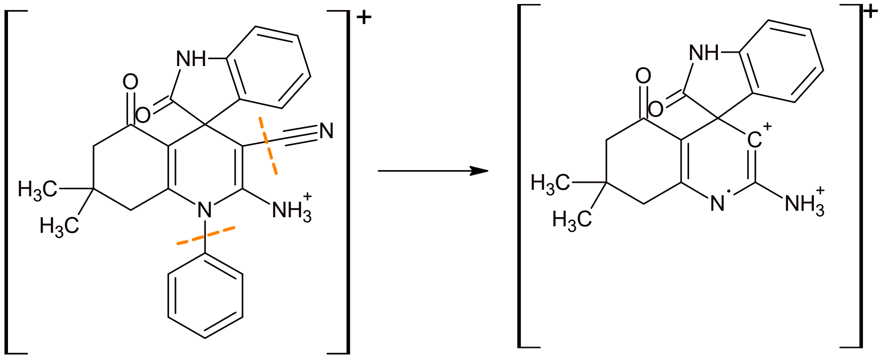

1.21. **Product 7b: 2′-Amino-7′,7′-dimethyl-1′-(3-methylphenyl)-2,5′-dioxo--5′,6′,7′,8′-tetrahydro-1*H*-spiro[indoline-3,4′-quinoline]-3′-carbonitrile**

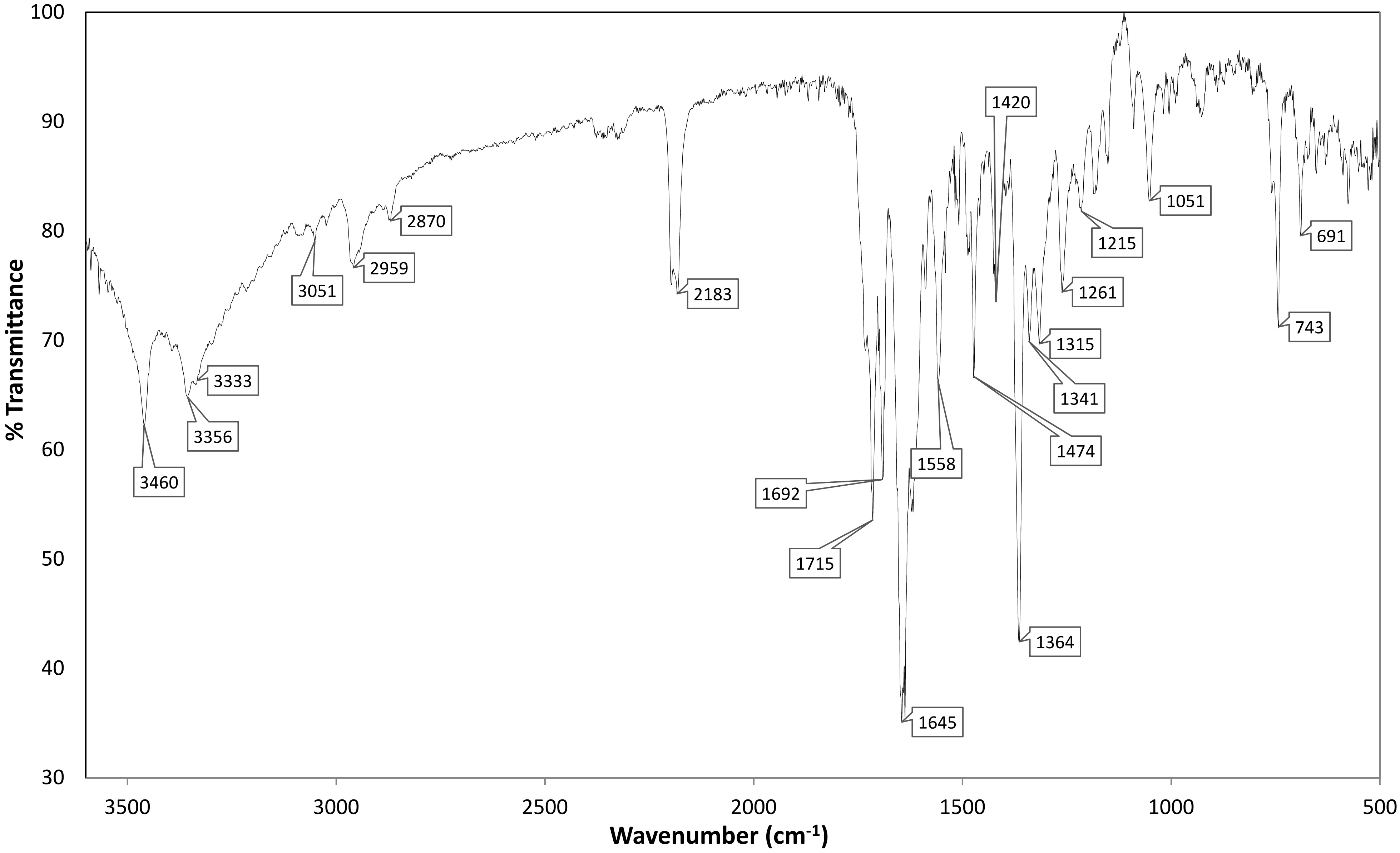

Figure S86 - IR spectrum of 7b

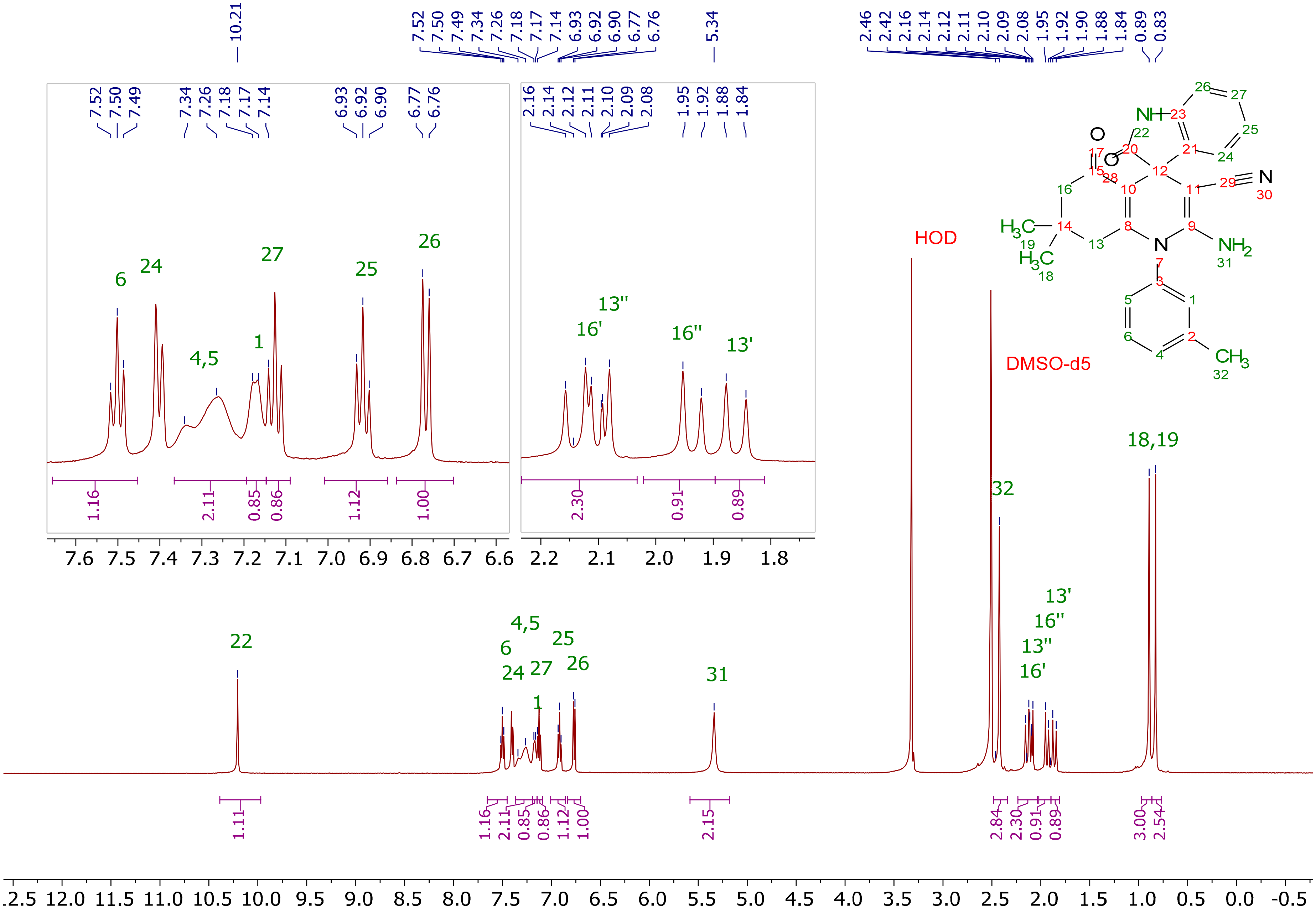

Figure S87 - ^{1}H NMR spectrum of 7b

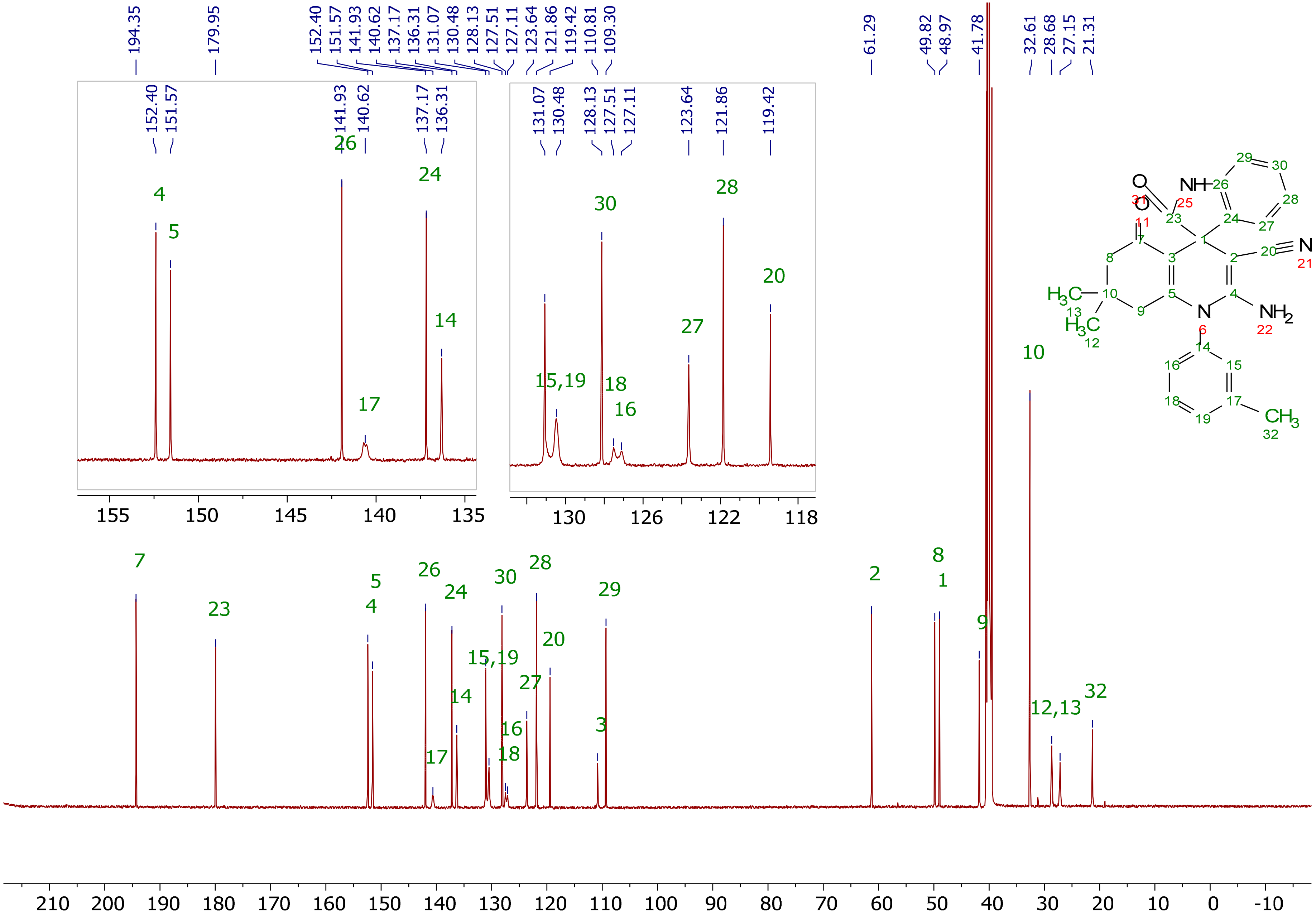

Figure S88 - ^{13}C NMR spectrum of 7b

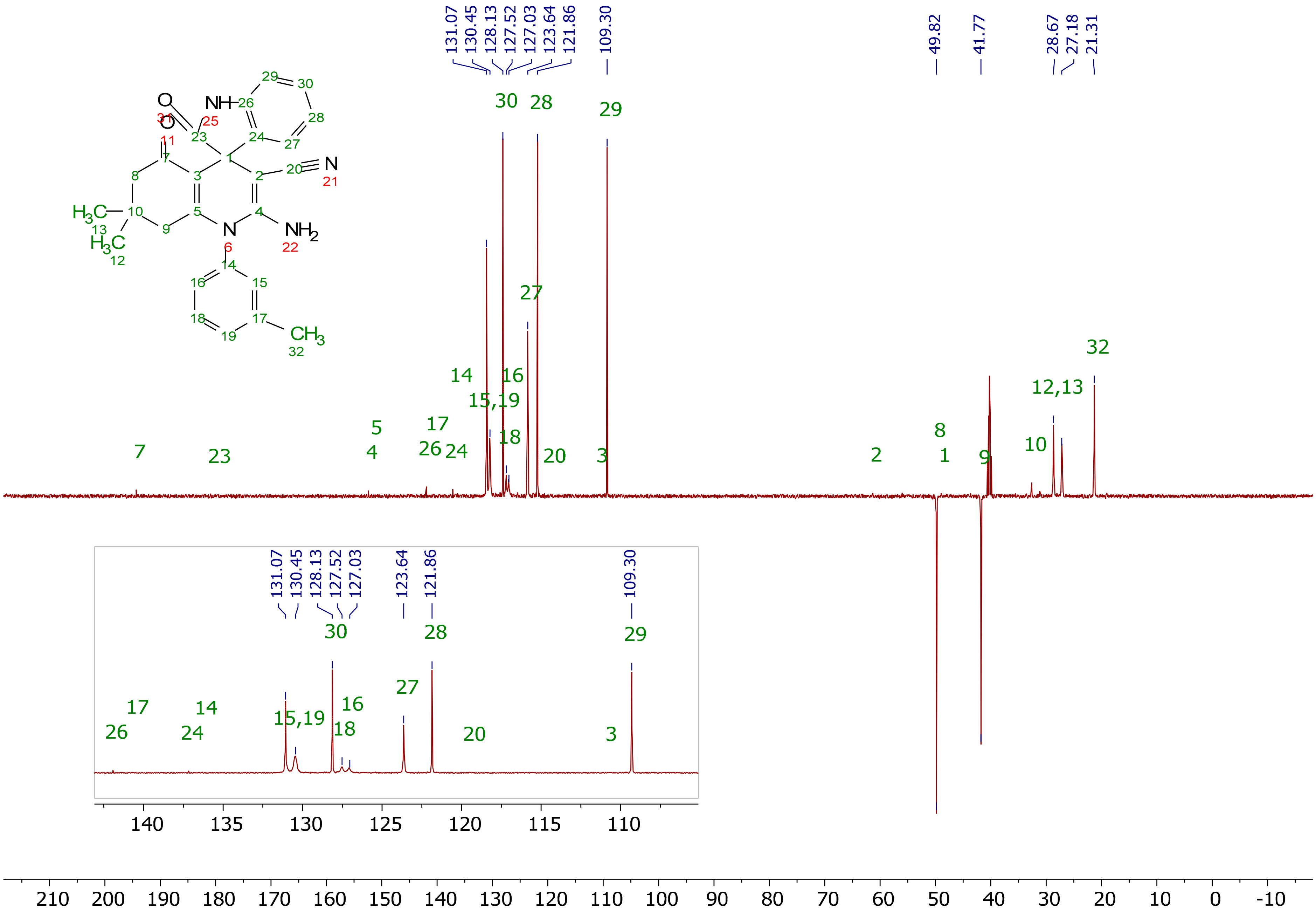

Figure S89 - DEPT spectrum of 7b

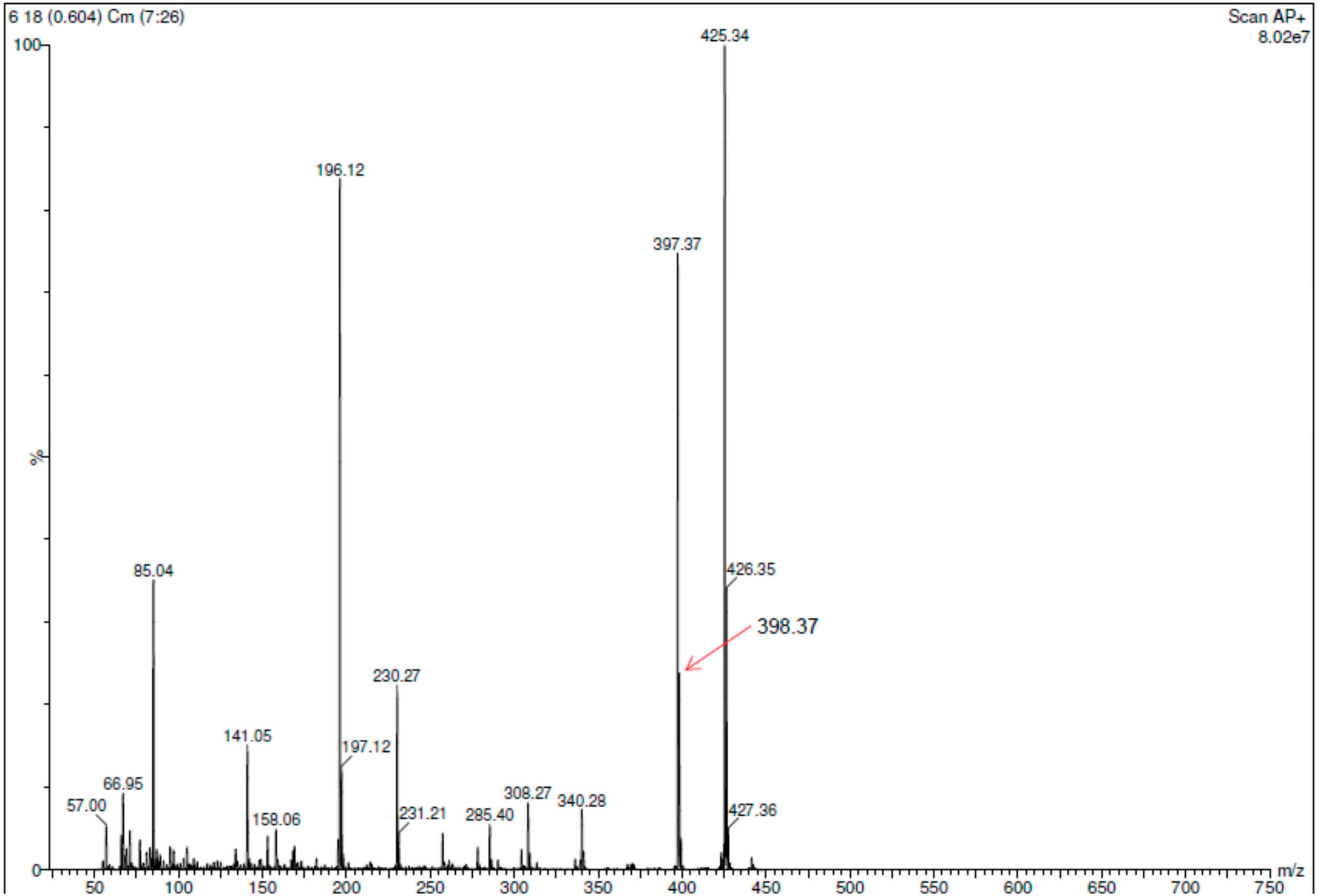

Figure S90 - MS spectrum of 7b

Table S11 - Fragmentation positions for peaks in MS spectrum of 7b

m/z	Fragmentation position
**426.35**	$[M+H]^+$
**398.37**	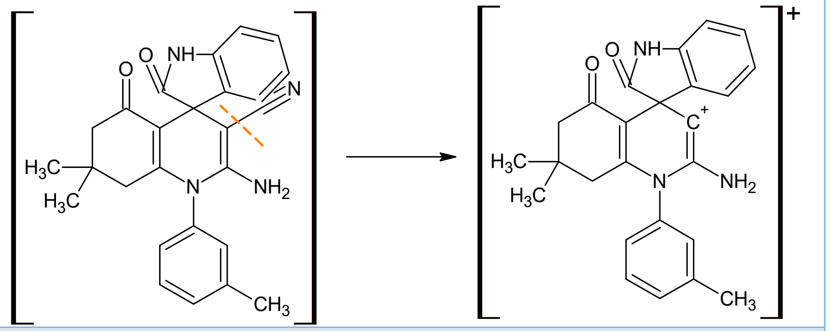
**340.28**	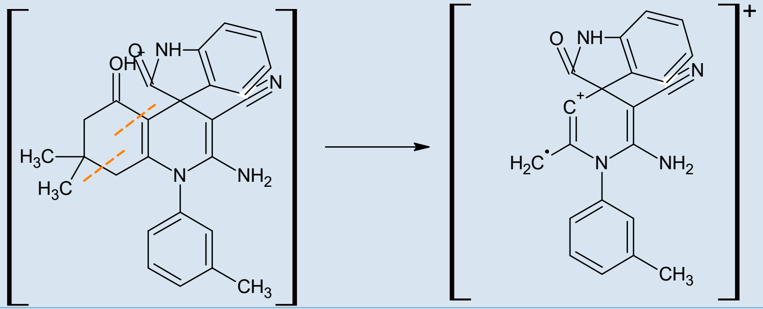
**308.27**	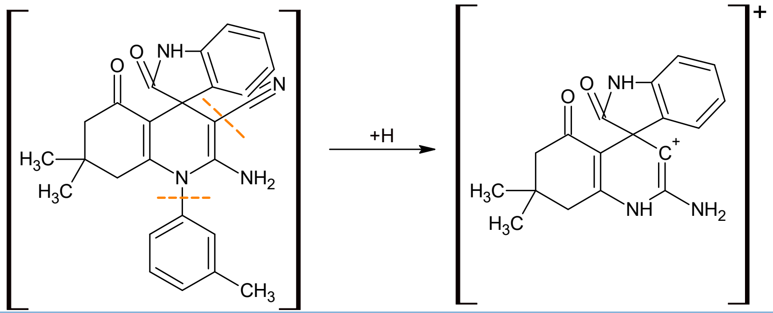

1.22. **Product 7c: 2′-Amino-7′,7′-dimethyl-1′-(3-nitrophenyl)-2,5′-dioxo-5′,6′,7′,8′-tetrahydro-1*H*-spiro[indoline-3,4′-quinoline]-3′-carbonitrile**

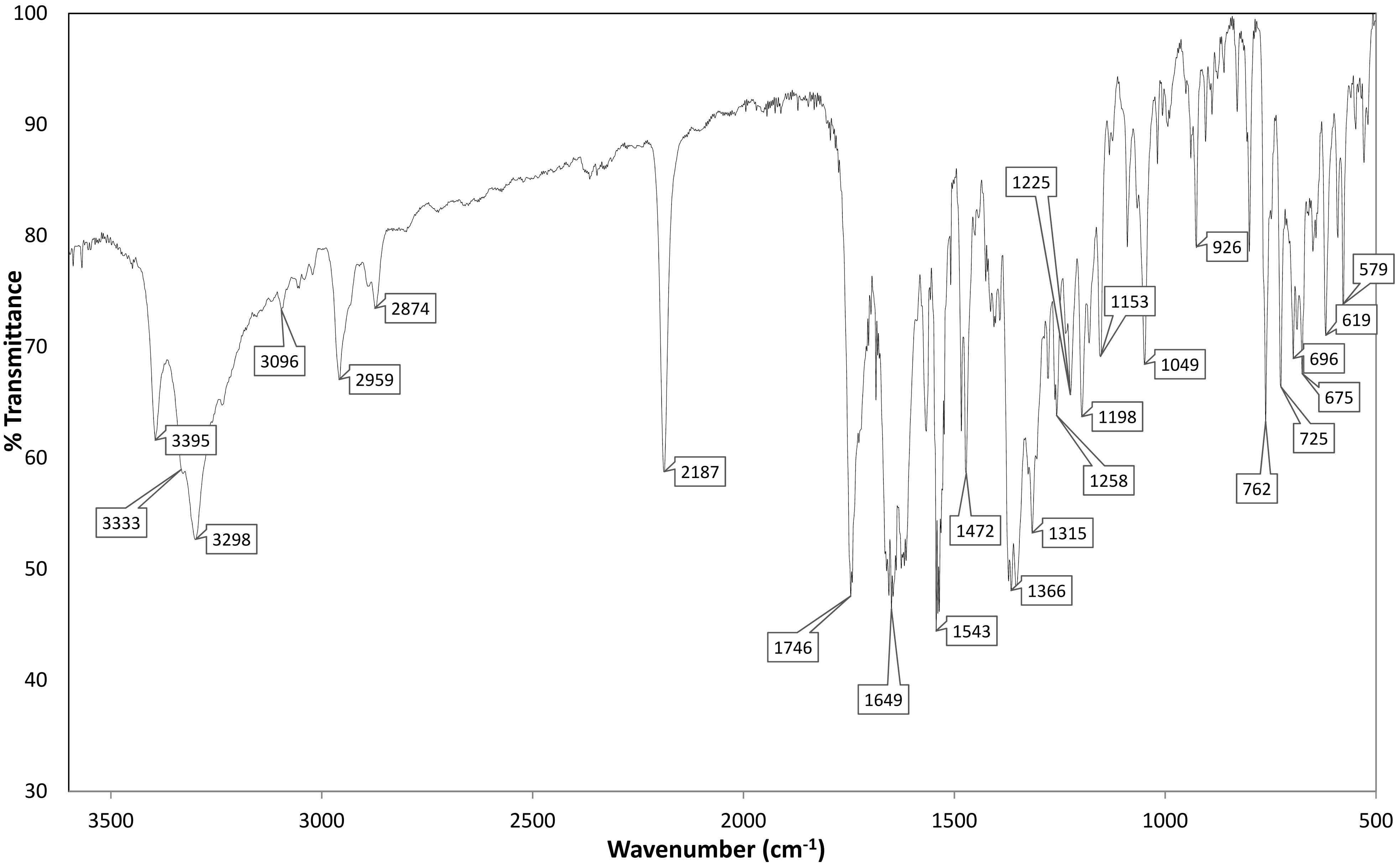

Figure S91 - IR spectrum of 7c

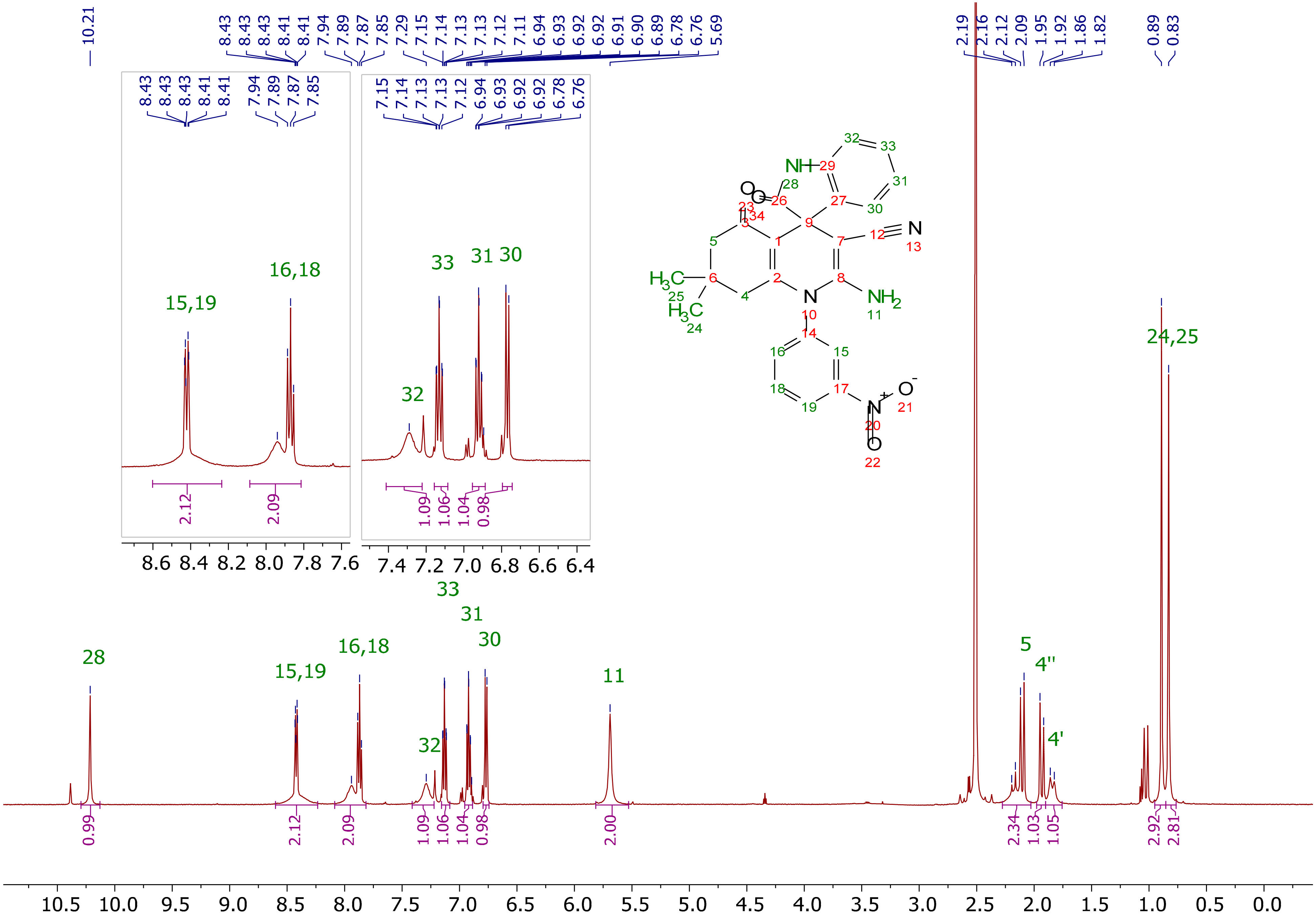

Figure S92 - ^{1}H NMR spectrum of 7c

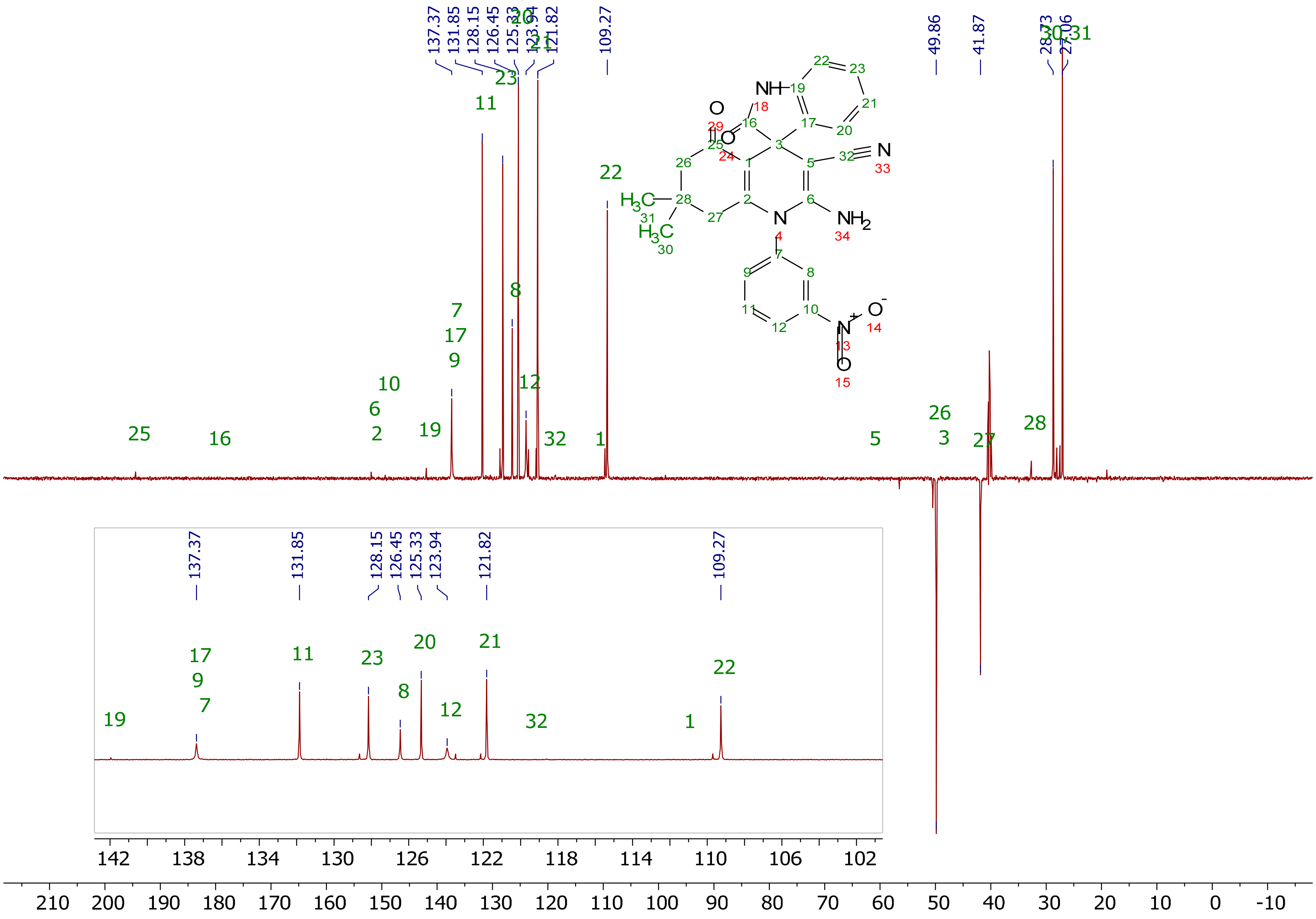

Figure S93 - ^{13}C NMR spectrum of 7c

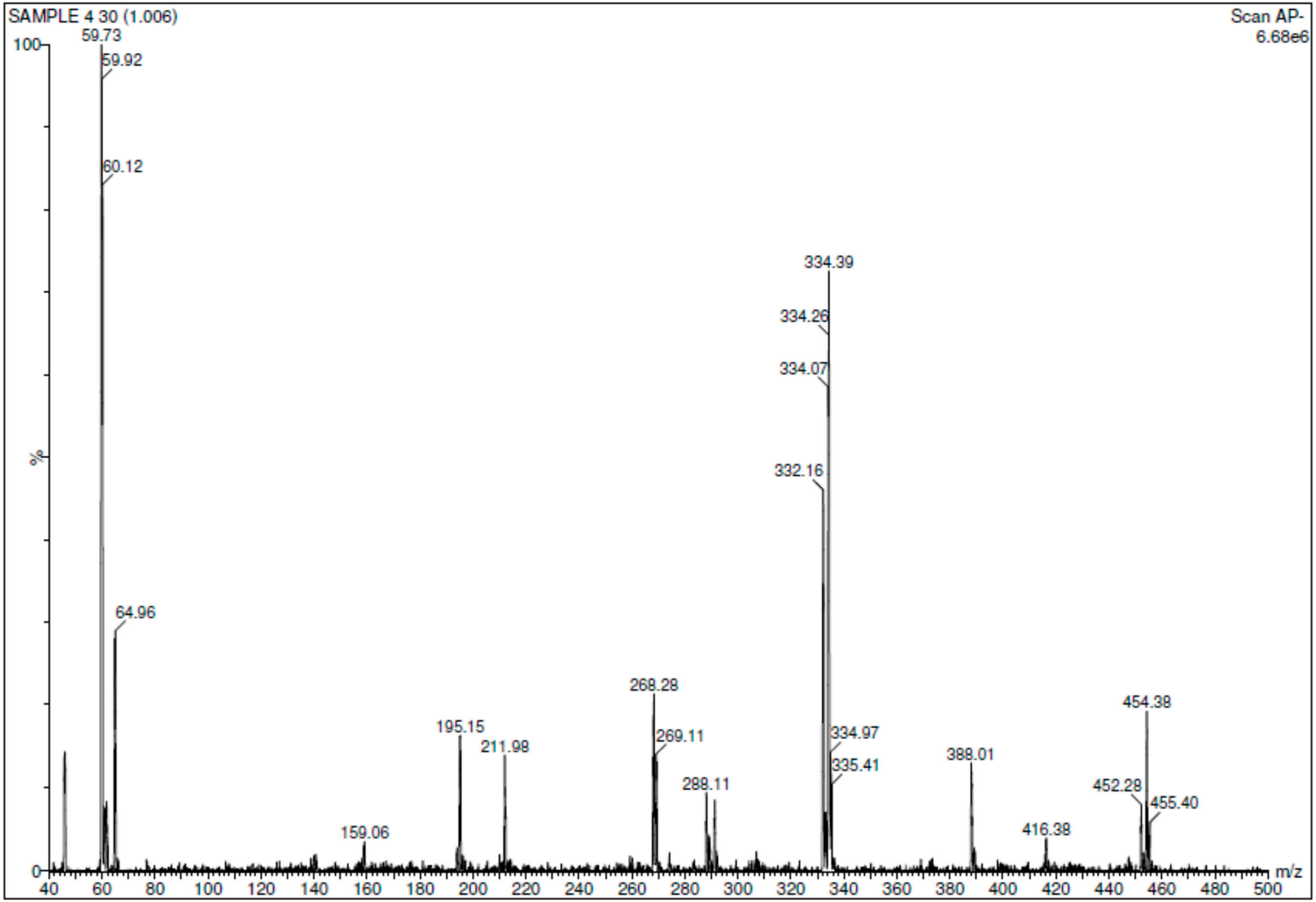

Figure S94 - Mass spectrum of 7c

Table S12 - Fragmentation positions for peaks in mass spectrum of 7c

m/z	Fragmentation position
455.40	$[M]^-$
334.07	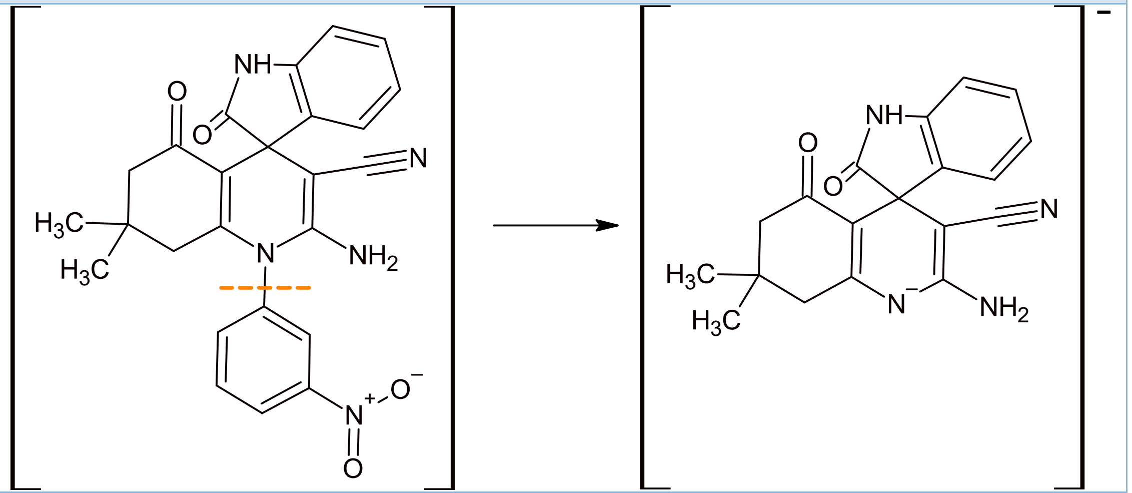

1.23. Product 7d: 2′-Amino-1′-(4-methoxyphenyl)-7′,7′-dimethyl-2,5′-dioxo-5′,6′,7′,8′-tetrahydro-1*H*-spiro[indoline-3,4′-quinoline]-3′-carbonitrile

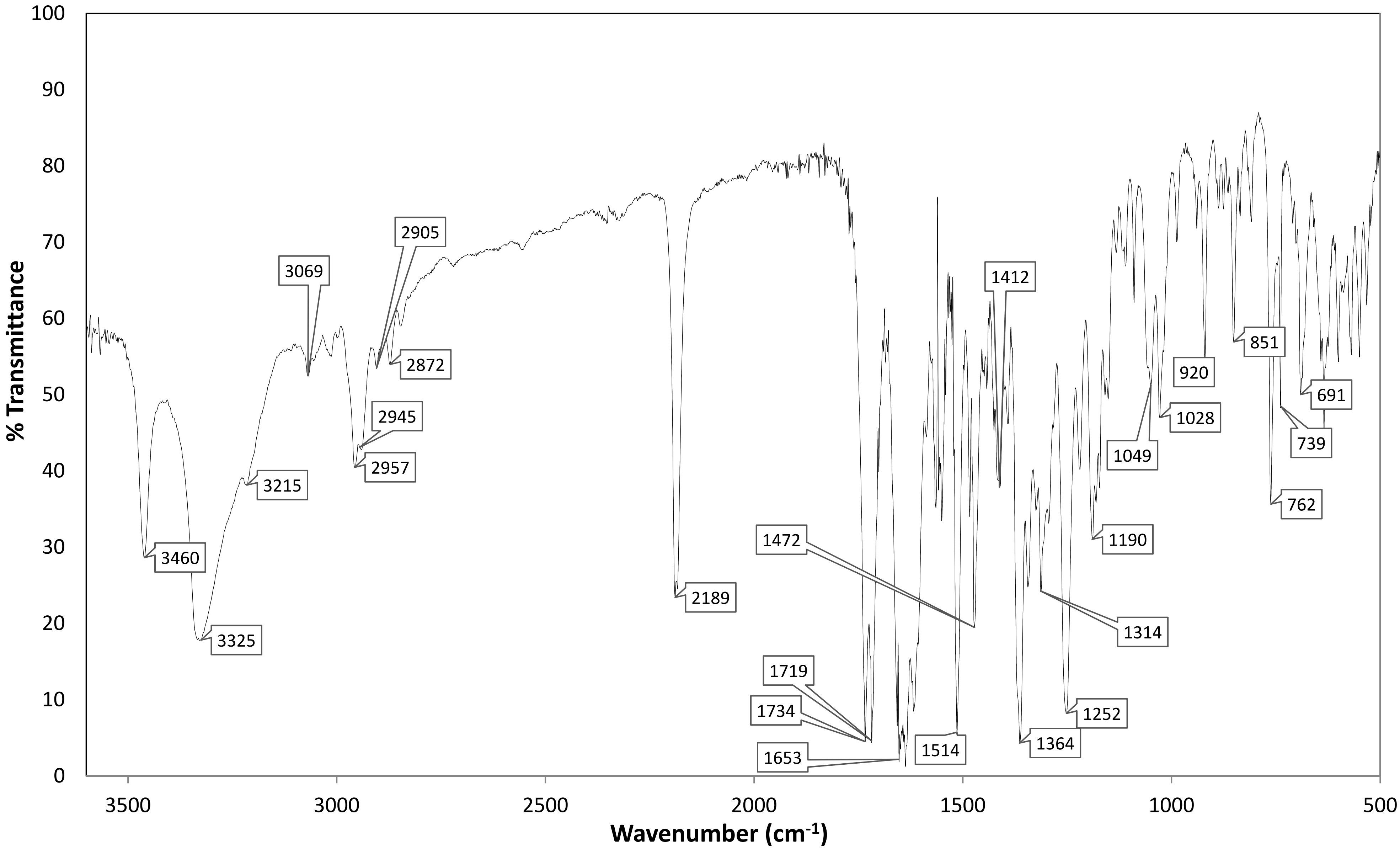

Figure S95 - IR spectrum of 7d

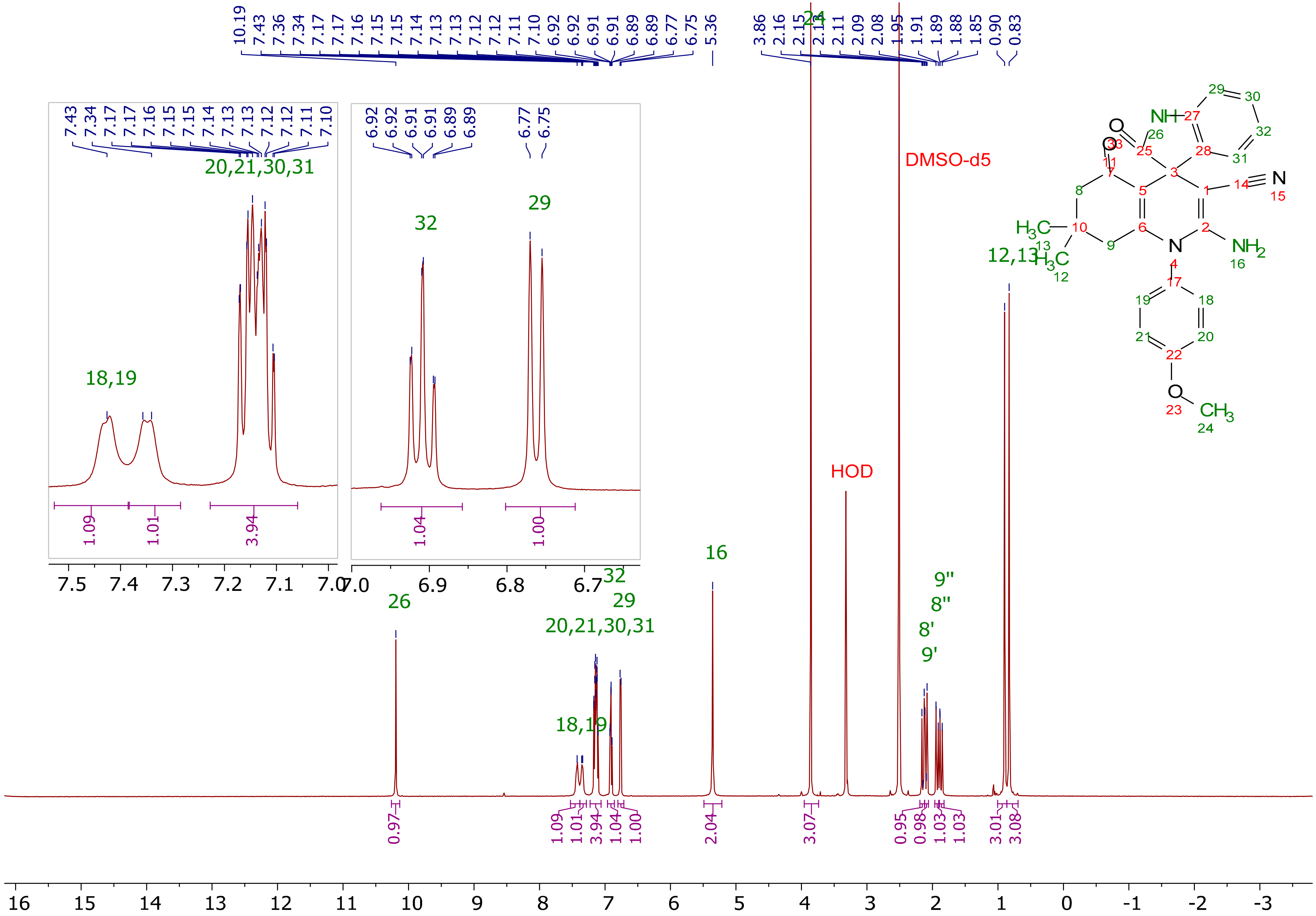

Figure S96 - ^{1}H NMR spectrum of 7d

1.24. Product 7e: 2′-Amino-1′-(4-chlorophenyl)-7′,7′-dimethyl-2,5′-dioxo-5′,6′,7′,8′-tetrahydro-1*H*-spiro[indoline-3,4′-quinoline]-3′-carbonitrile

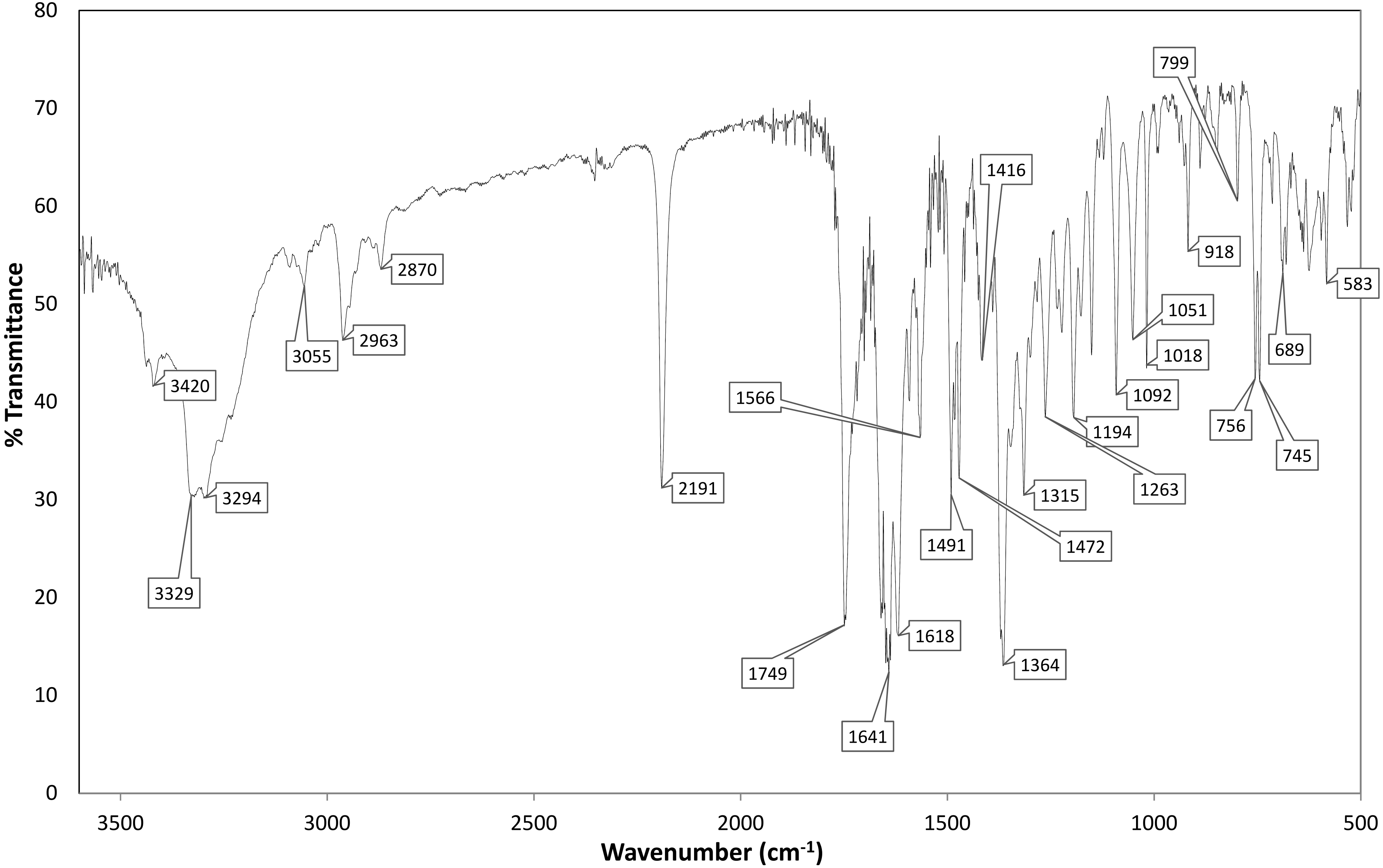

Figure S97 - IR spectrum of 7e

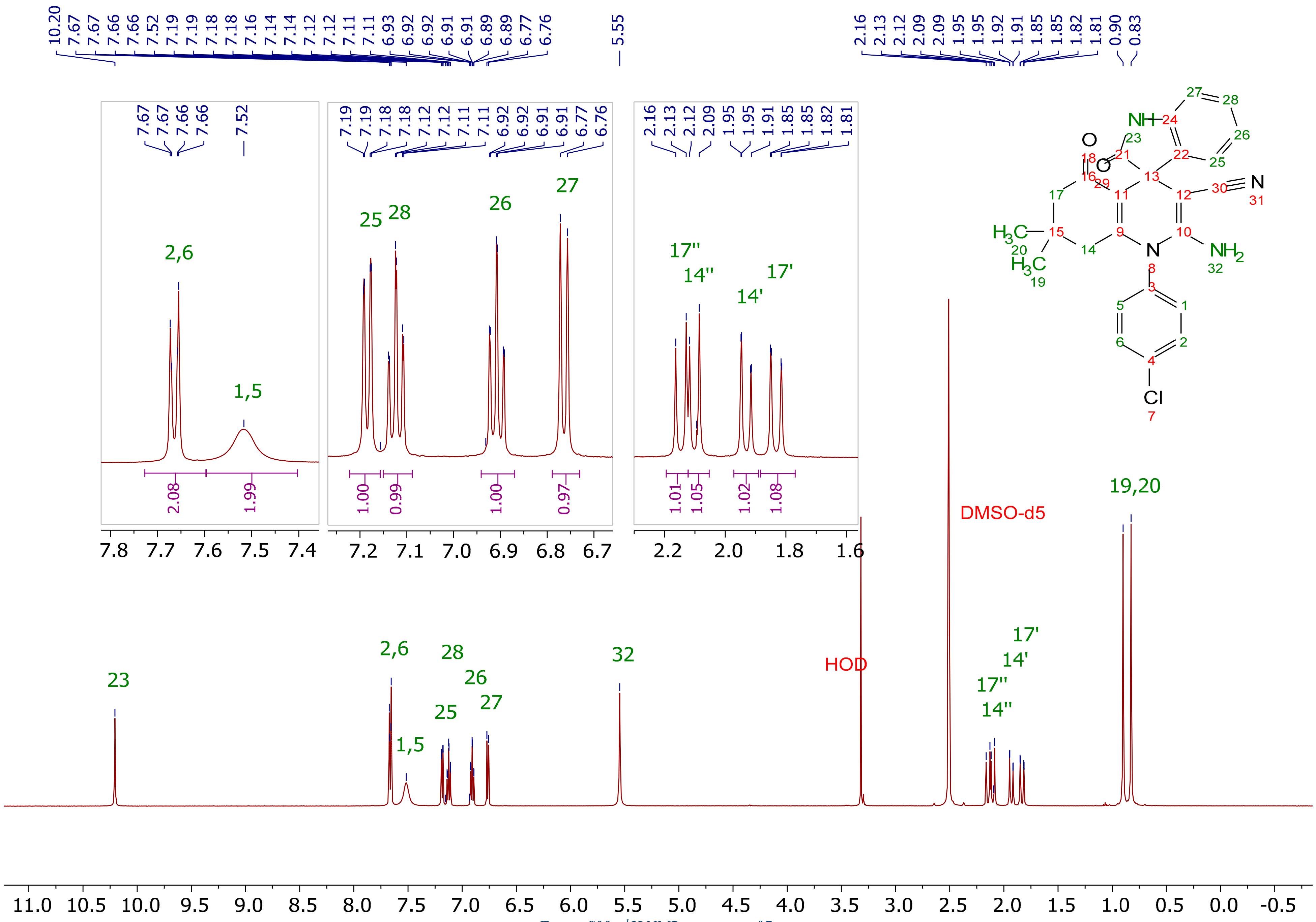

Figure S98 - ^{1}H NMR spectrum of 7e

1.25. Product 7f: 2′-Amino-1′-(4-bromophenyl)-7′,7′-dimethyl-2,5′-dioxo-5′,6′,7′,8′-tetrahydro-1*H*-spiro[indoline-3,4′-quinoline]-3′-carbonitrile

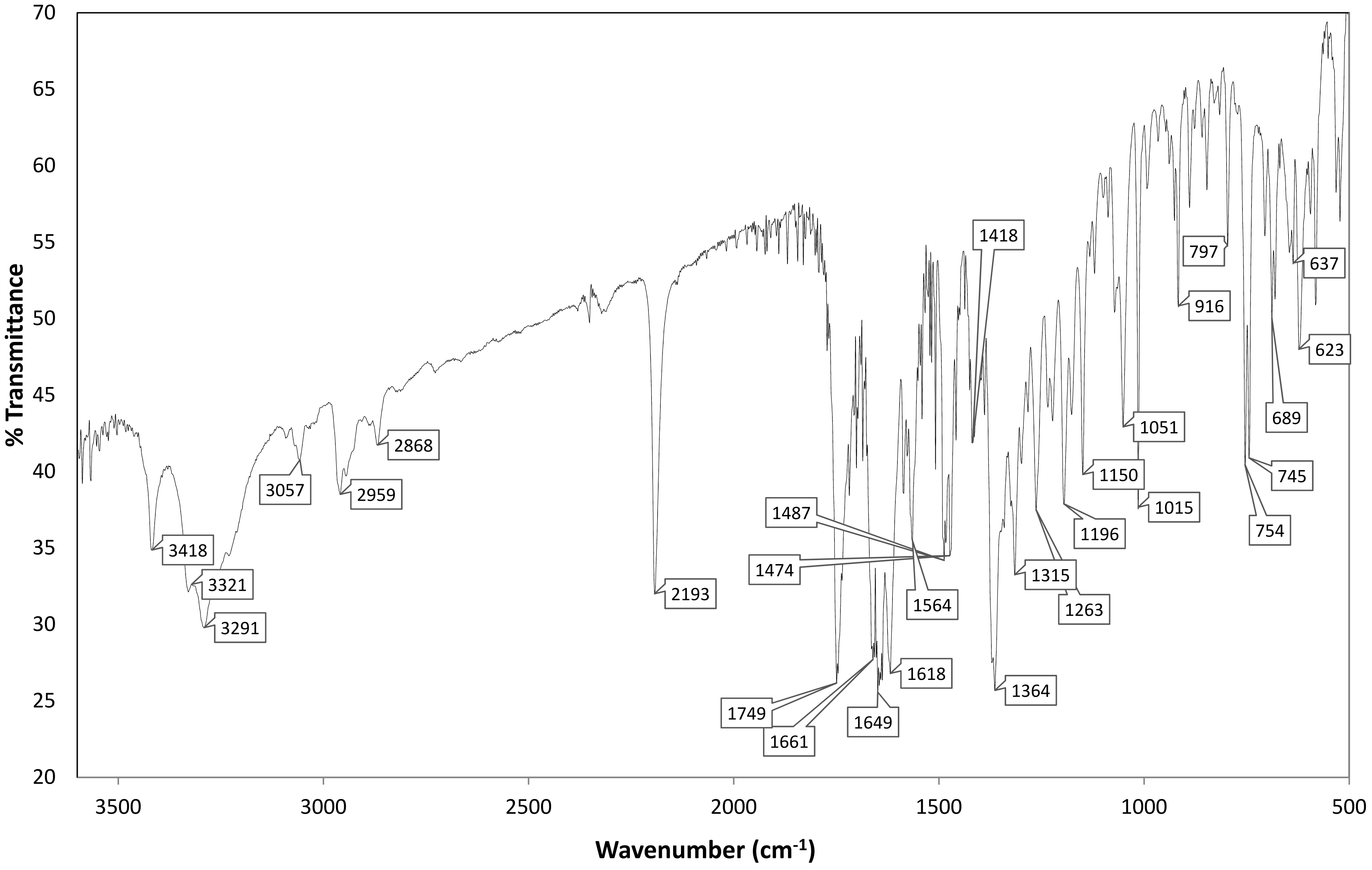

Figure S99 - IR spectrum of 7f

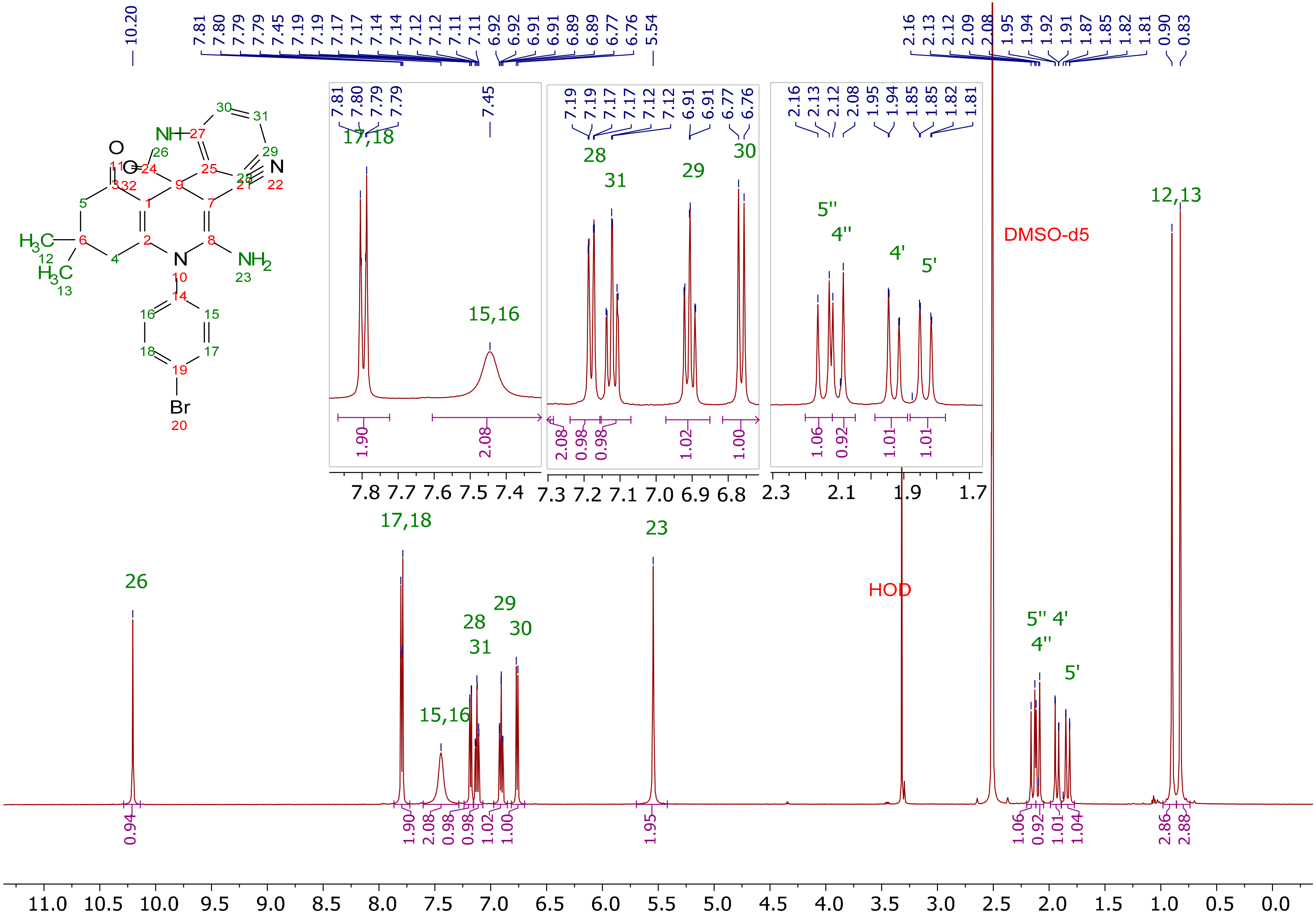

Figure S100 - ^{1}H NMR spectrum of 7f

1.26. Product 7g: 2′-Amino-7′,7′-dimethyl-1′-(4-methylphenyl)-2,5′-dioxo-5′,6′,7′,8′-tetrahydro-1*H*-spiro[indoline-3,4′-quinoline]-3′-carbonitrile

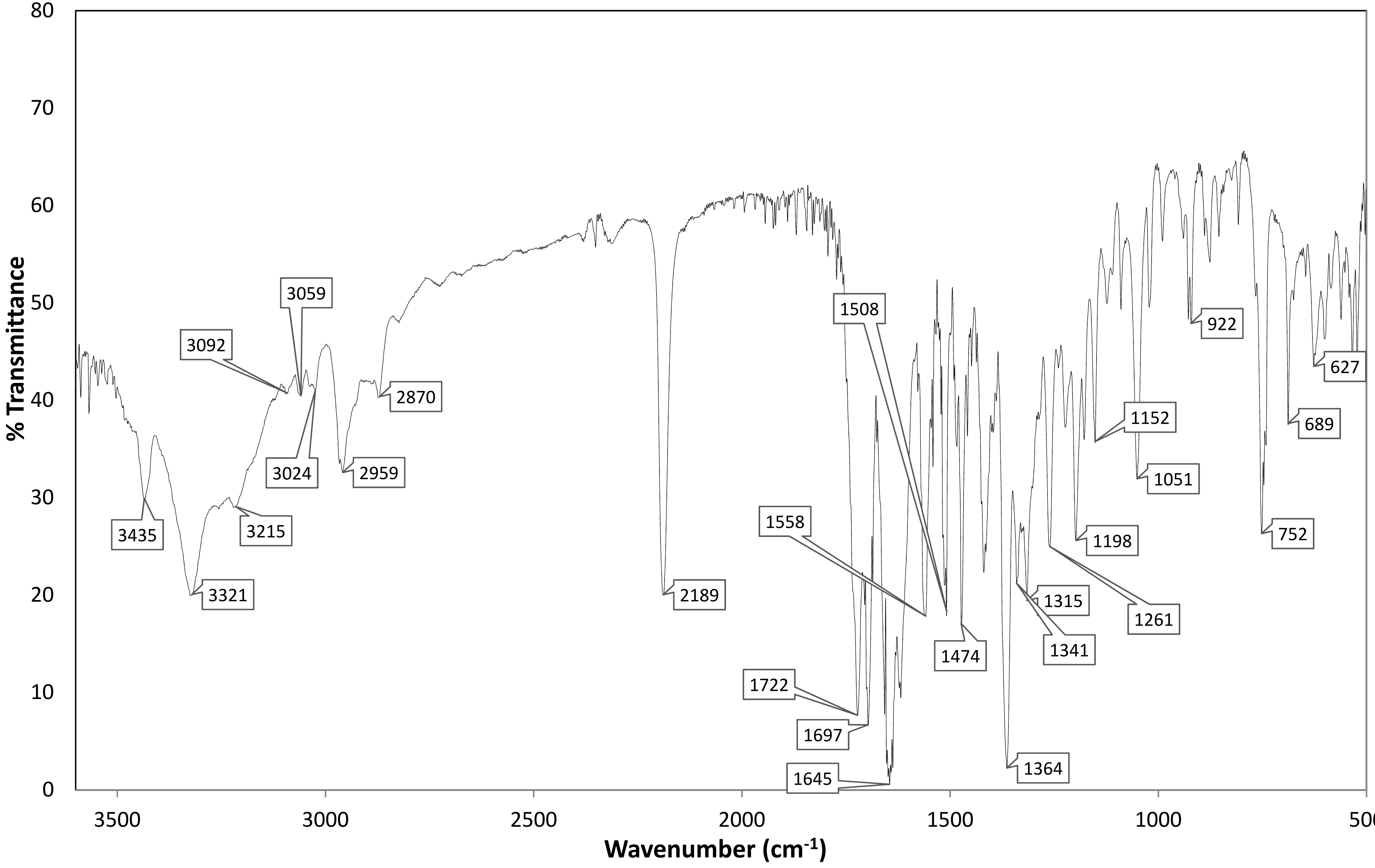

Figure S101 - IR spectrum of 7g

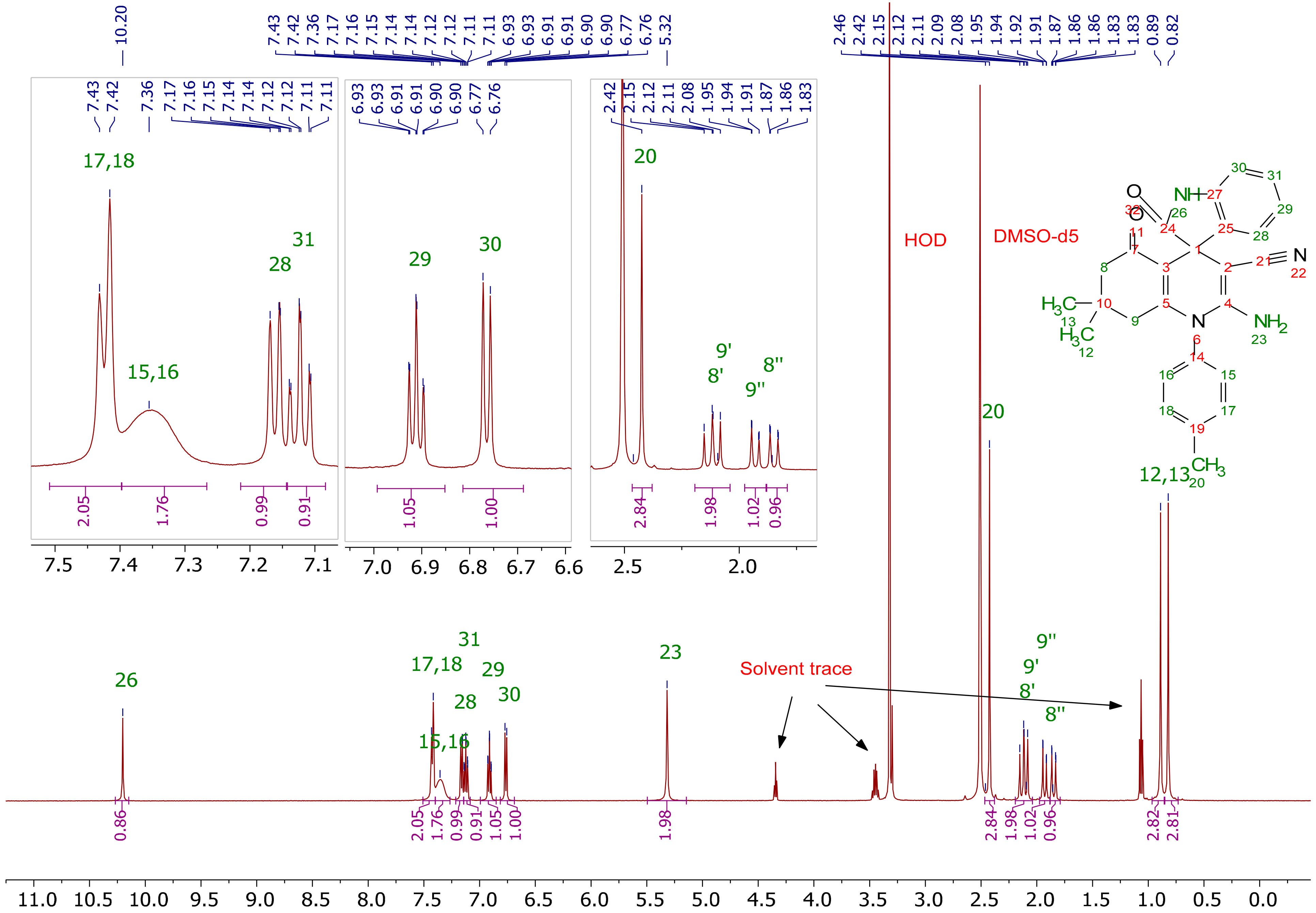

Figure S102 - ^{1}H NMR spectrum of 7g

1.27. Product 7i: 2′-Amino-1′-(3-chlorophenyl)-7′,7′-dimethyl-1′-2,5′-dioxo-5′,6′,7′,8′-tetrahydro-1*H*-spiro[indoline-3,4′-quinoline]-3′-carbonitrile

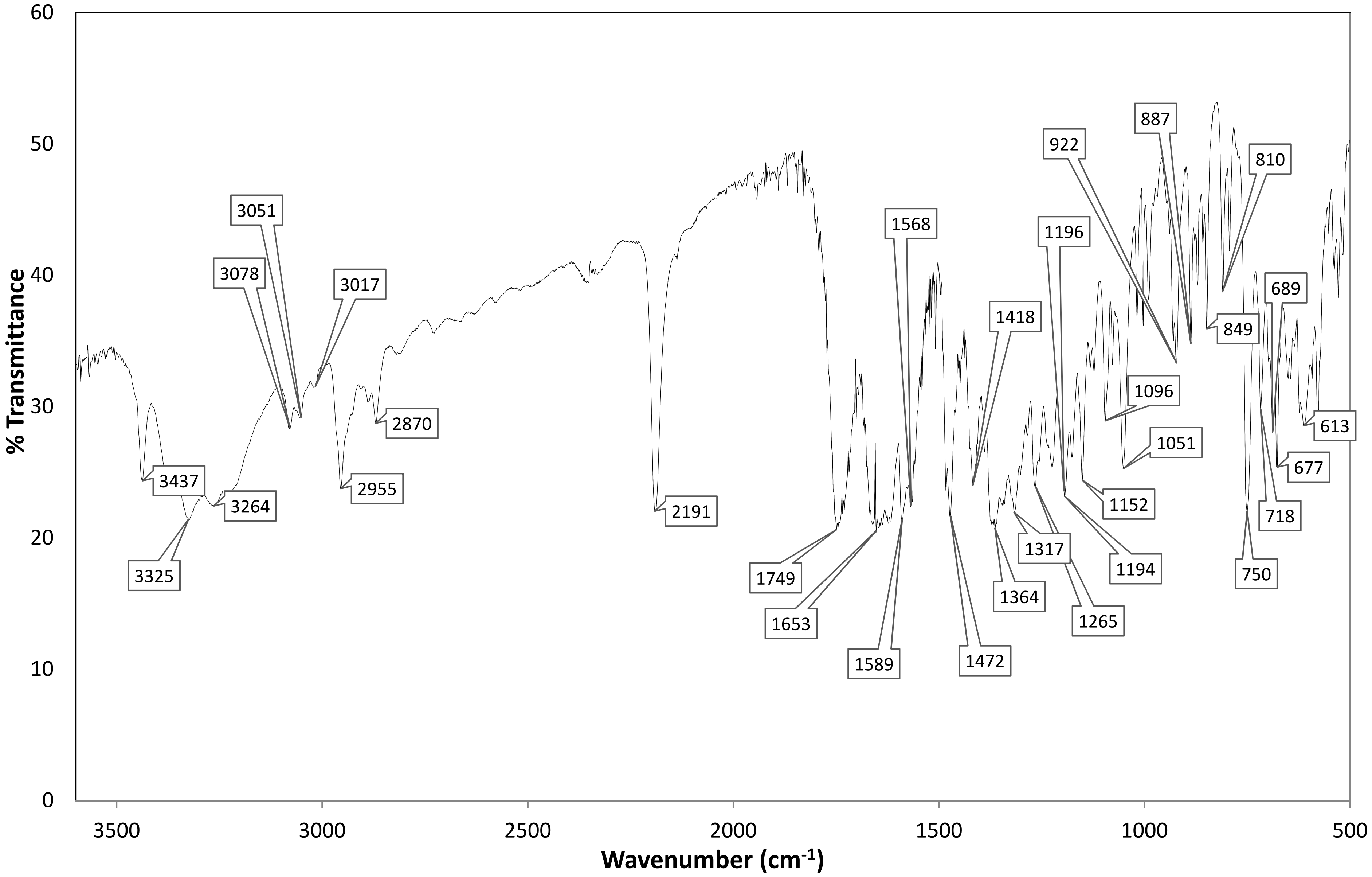

Figure S103 - IR spectrum of 7i

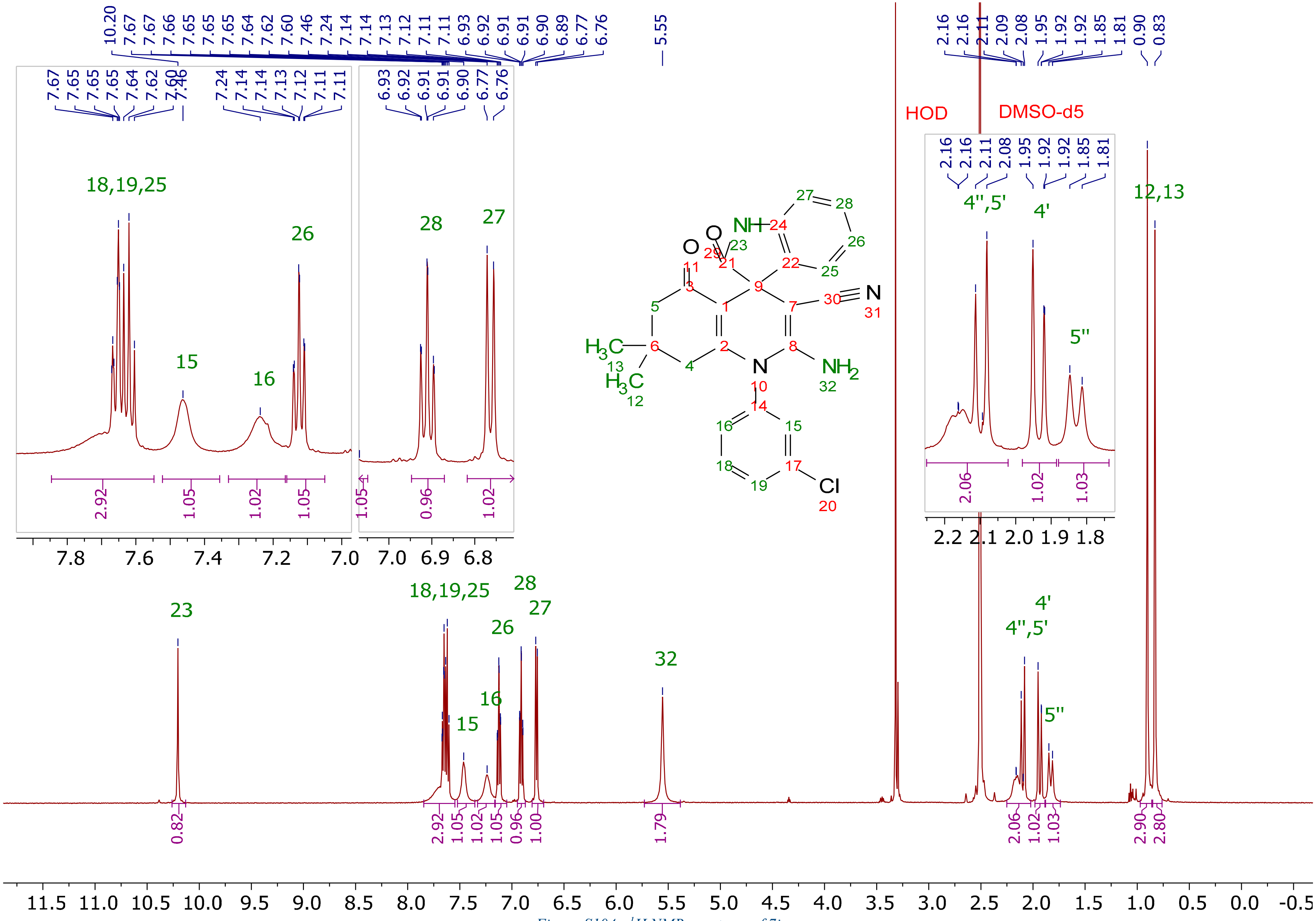

Figure S104 - ^{1}H NMR spectrum of 7i

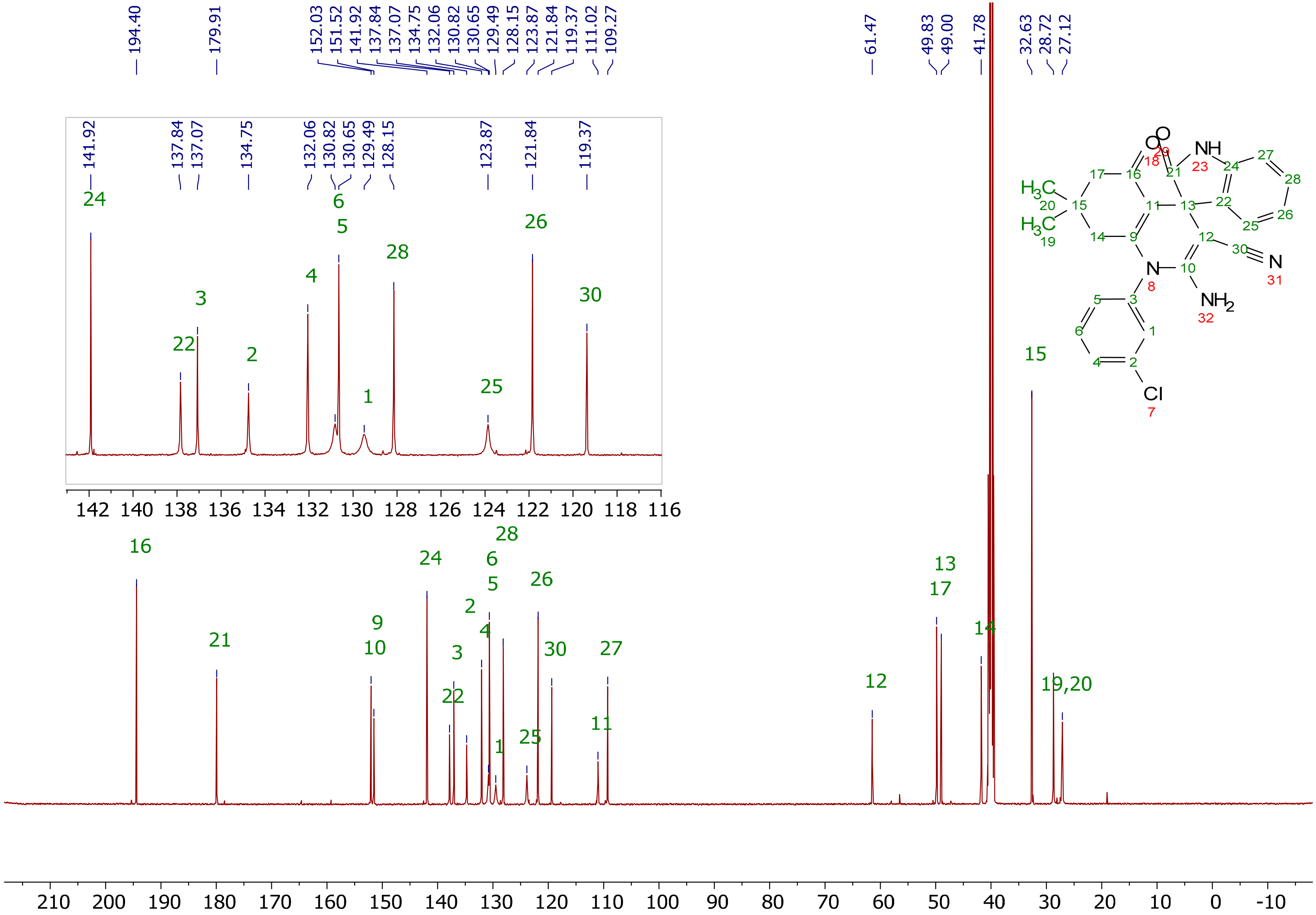

Figure S105 - ^{13}C NMR spectrum of 7i

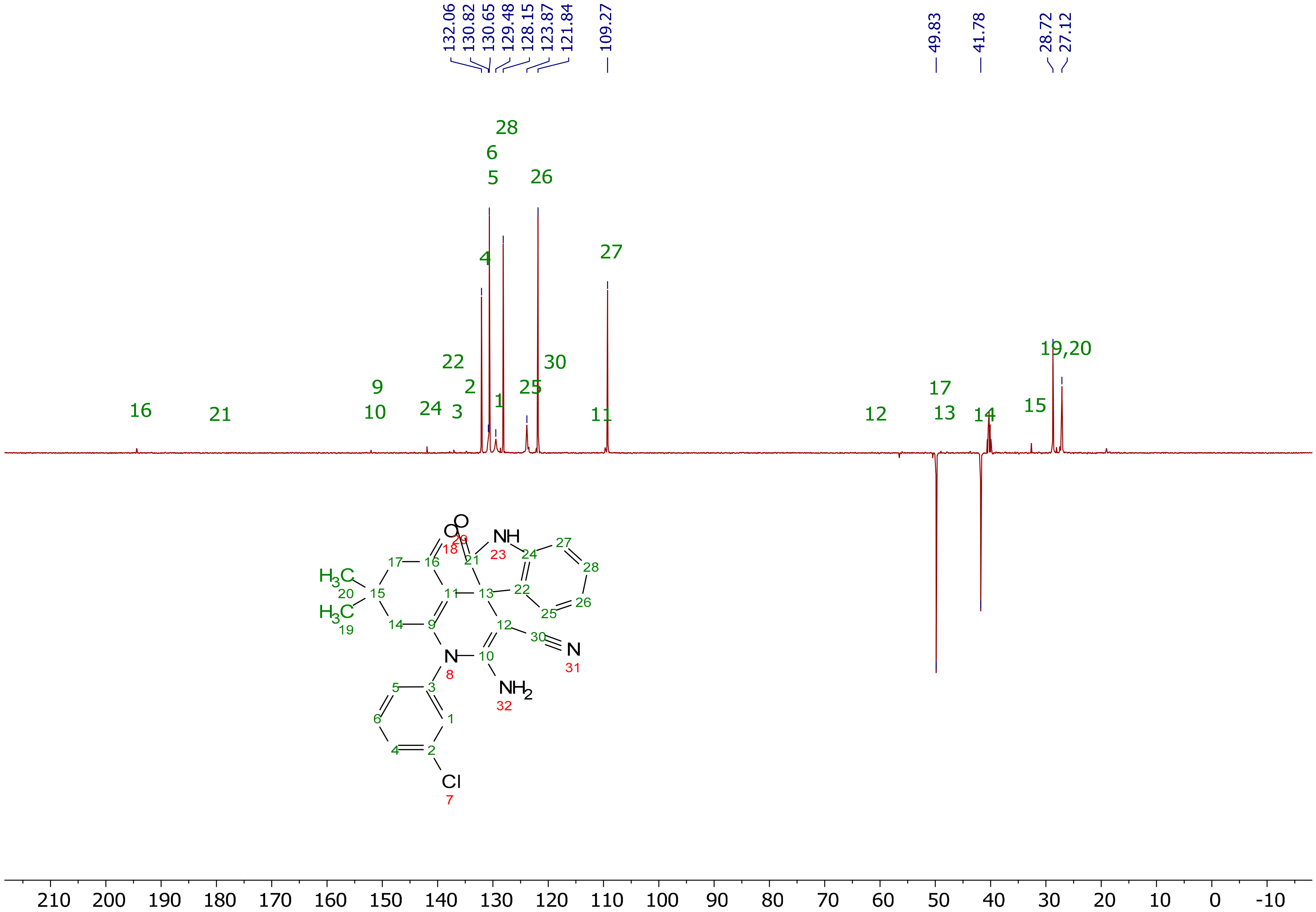

Figure S106 - DEPT spectrum of 7i

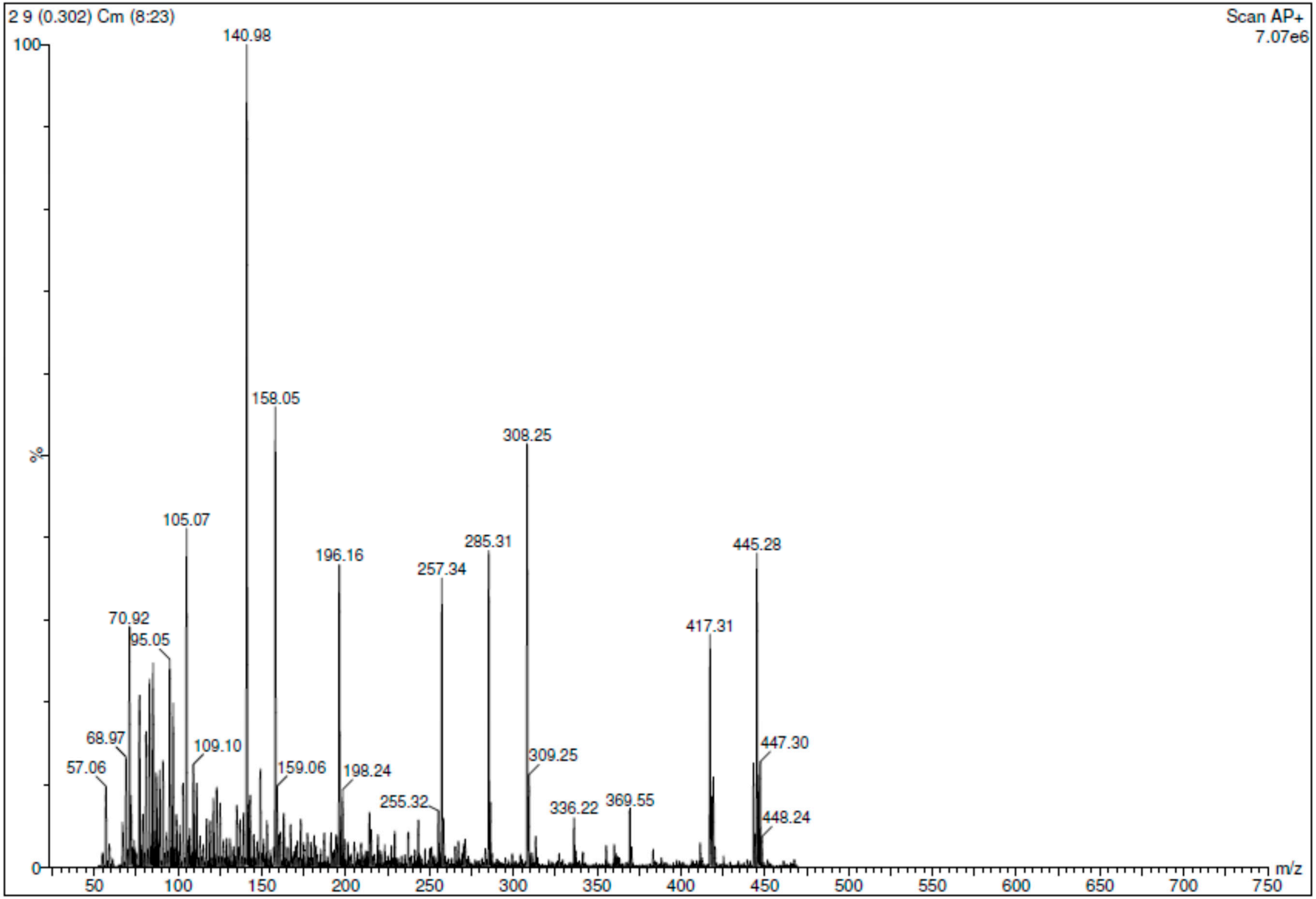

Figure S107 - MS spectrum of 7i

Table S13 - Fragmentation positions for peaks in MS spectrum of 7i

m/z	Fragmentation position
**445.28 – 448.24 (isotopes)**	$[M+H]^+$
**417.31**	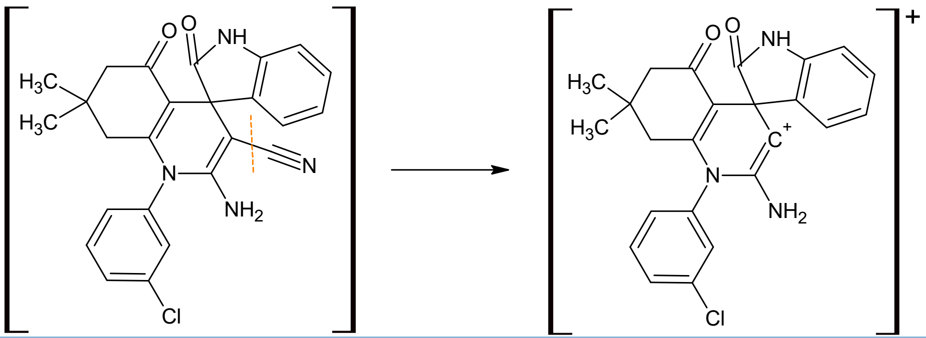
**308.25**	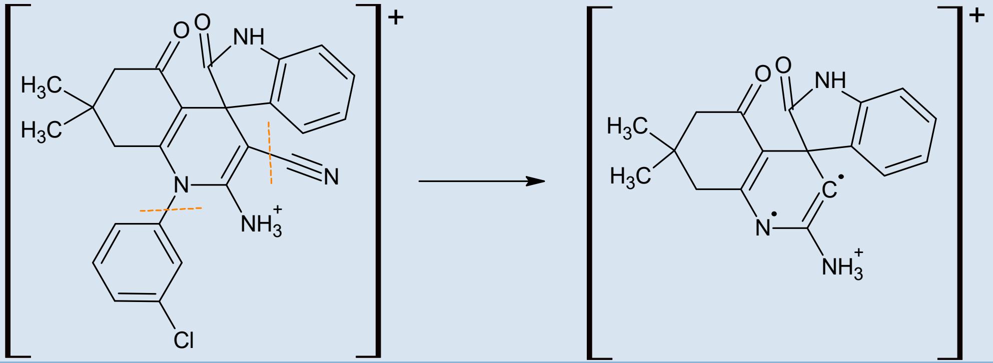
**336.22**	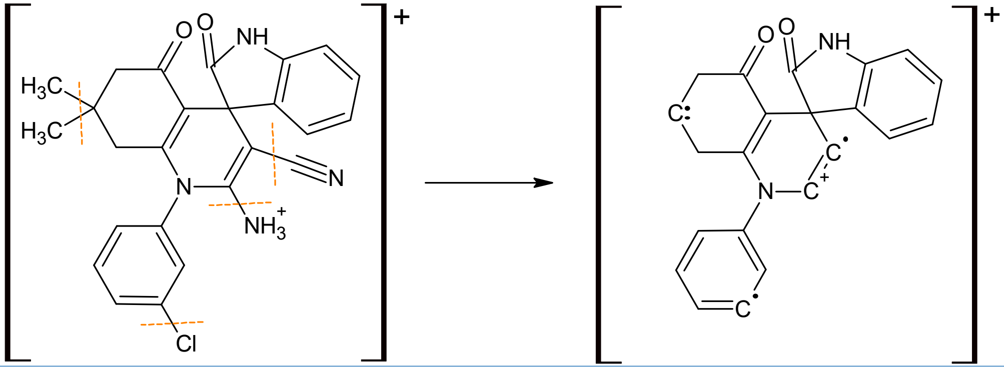

1.28. Product 12: 1-[4-[(4-bromophenyl)(1*H*-indol-3-yl)methyl]phenyl]-7,7-trimethyl-4-(3-nitrophenyl)-4,6,7,8-tetrahydroquinoline-2,5(1*H*,3*H*)-dione

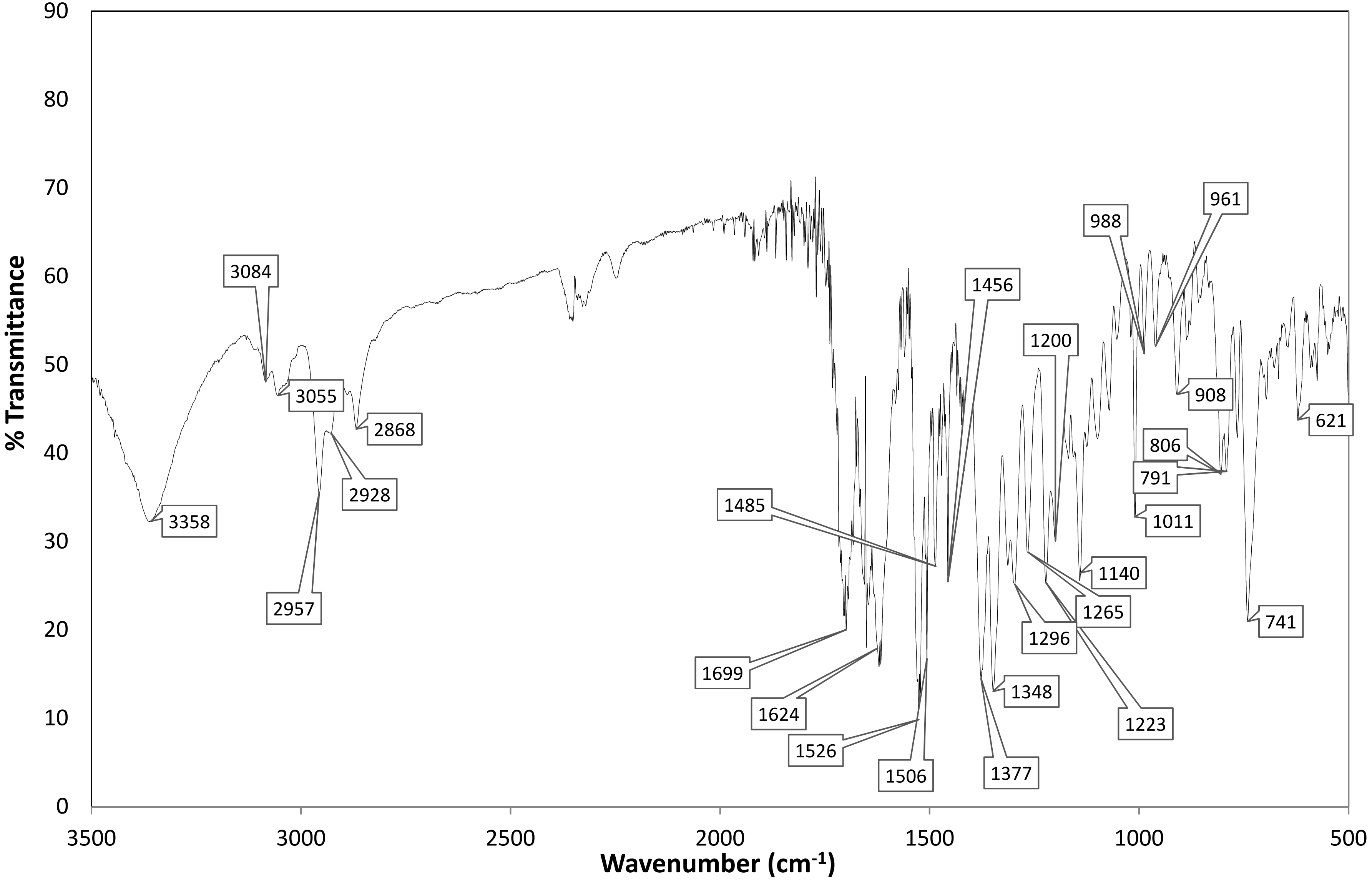

Figure S108 - IR spectrum of 12

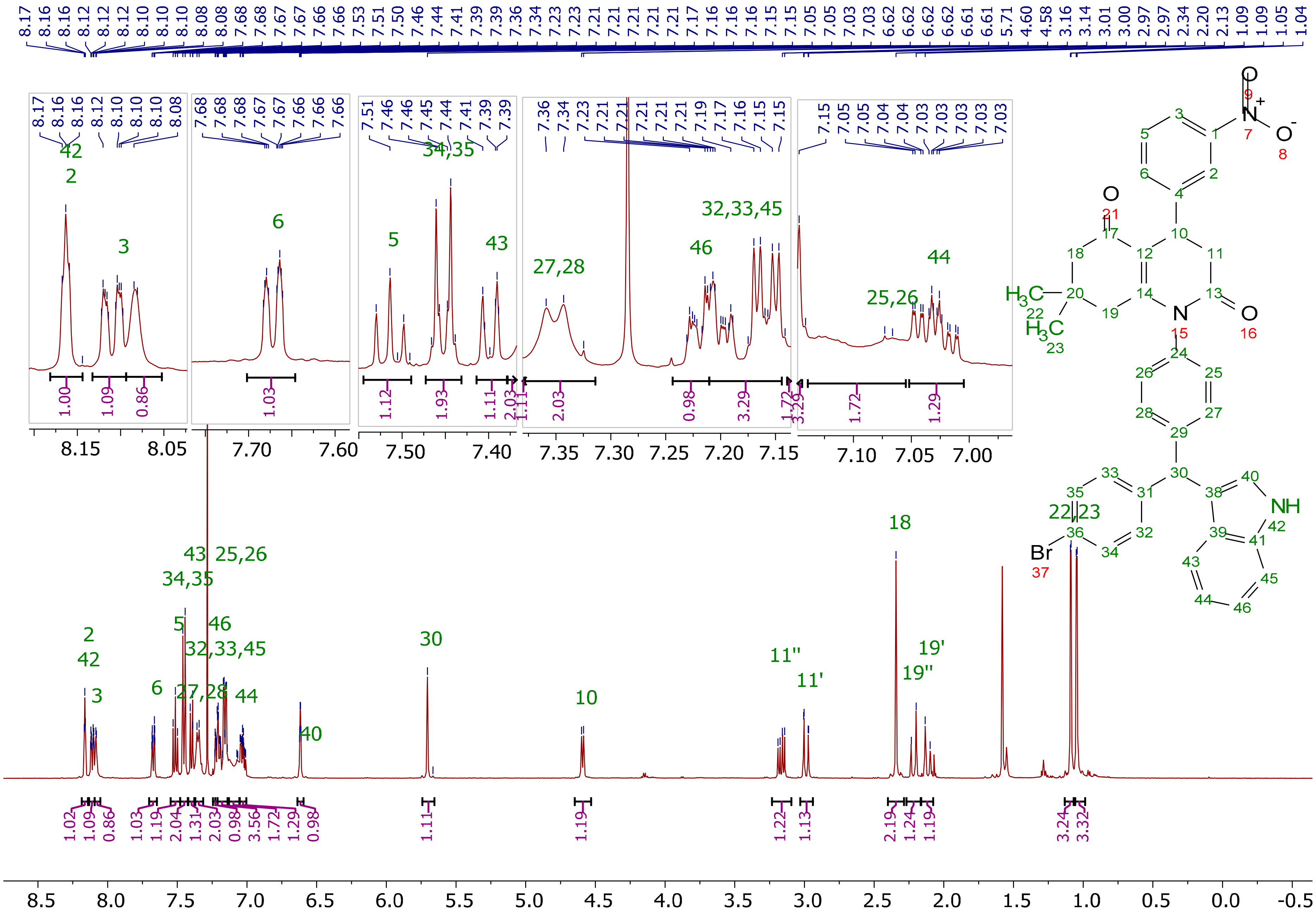

Figure S109 - ¹H NMR spectrum of 12

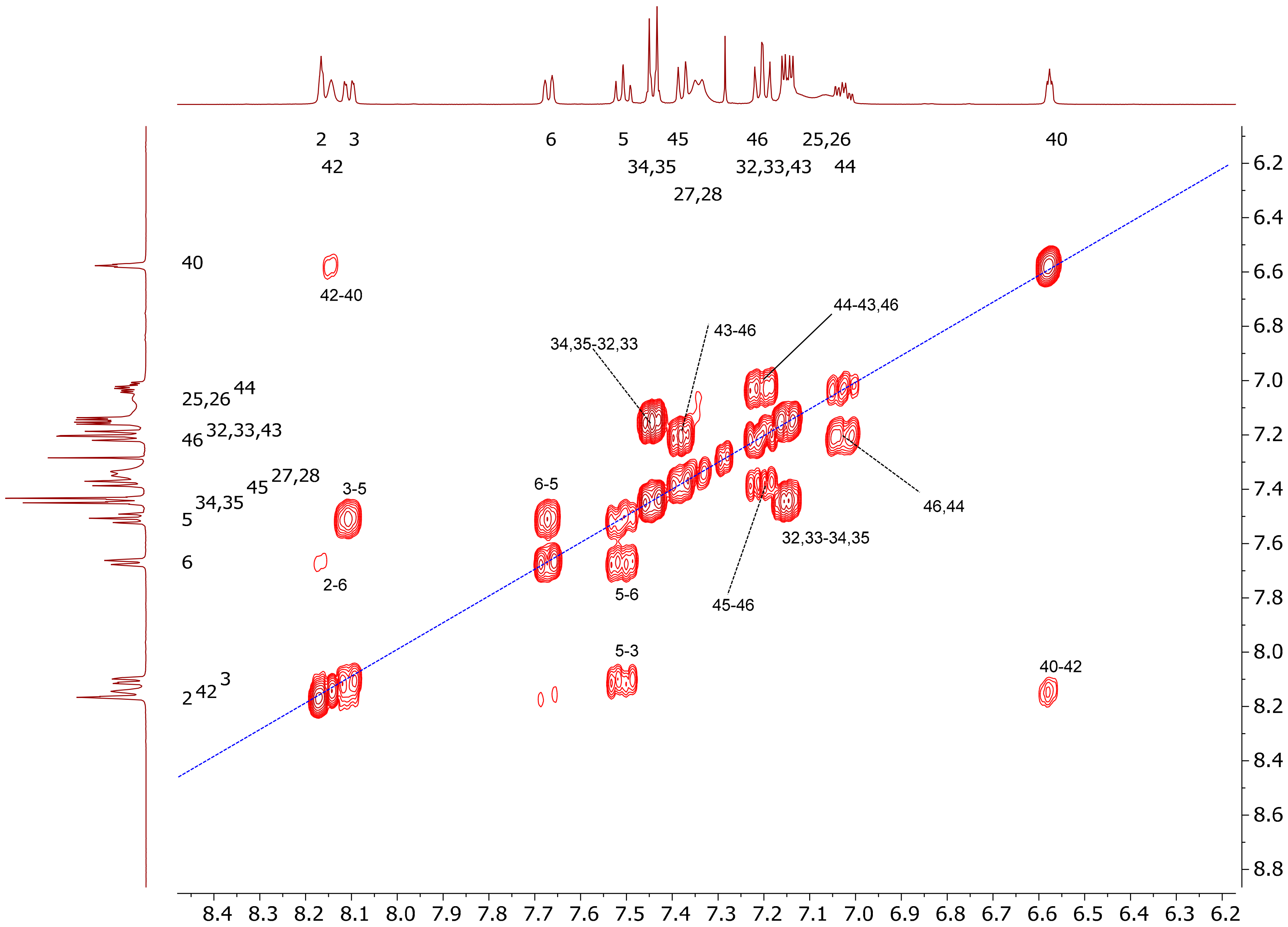

Figure S110 - ^{1}H-^{1}H COSY spectrum of 12

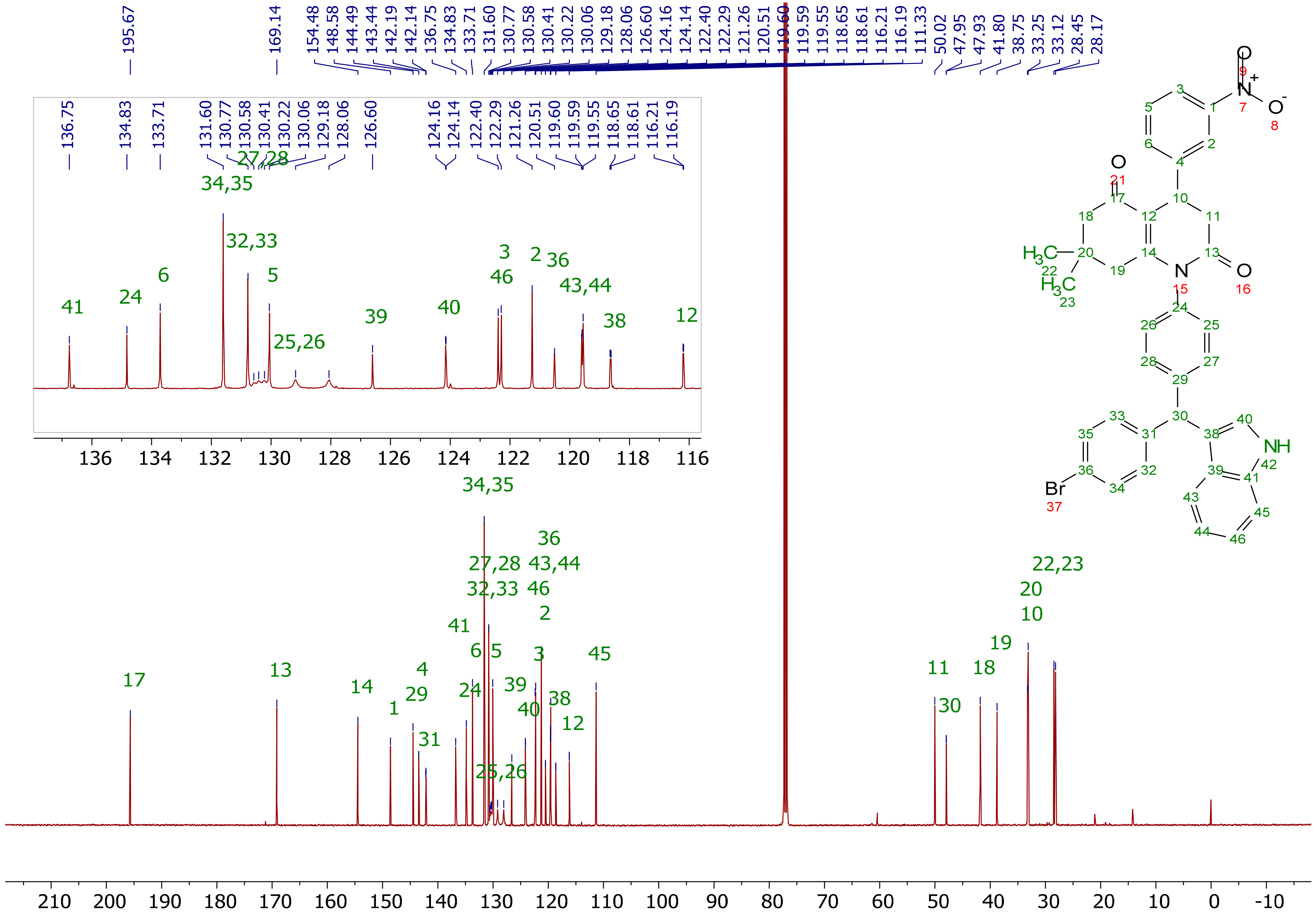

Figure S111 - ^{13}C NMR spectrum of 12

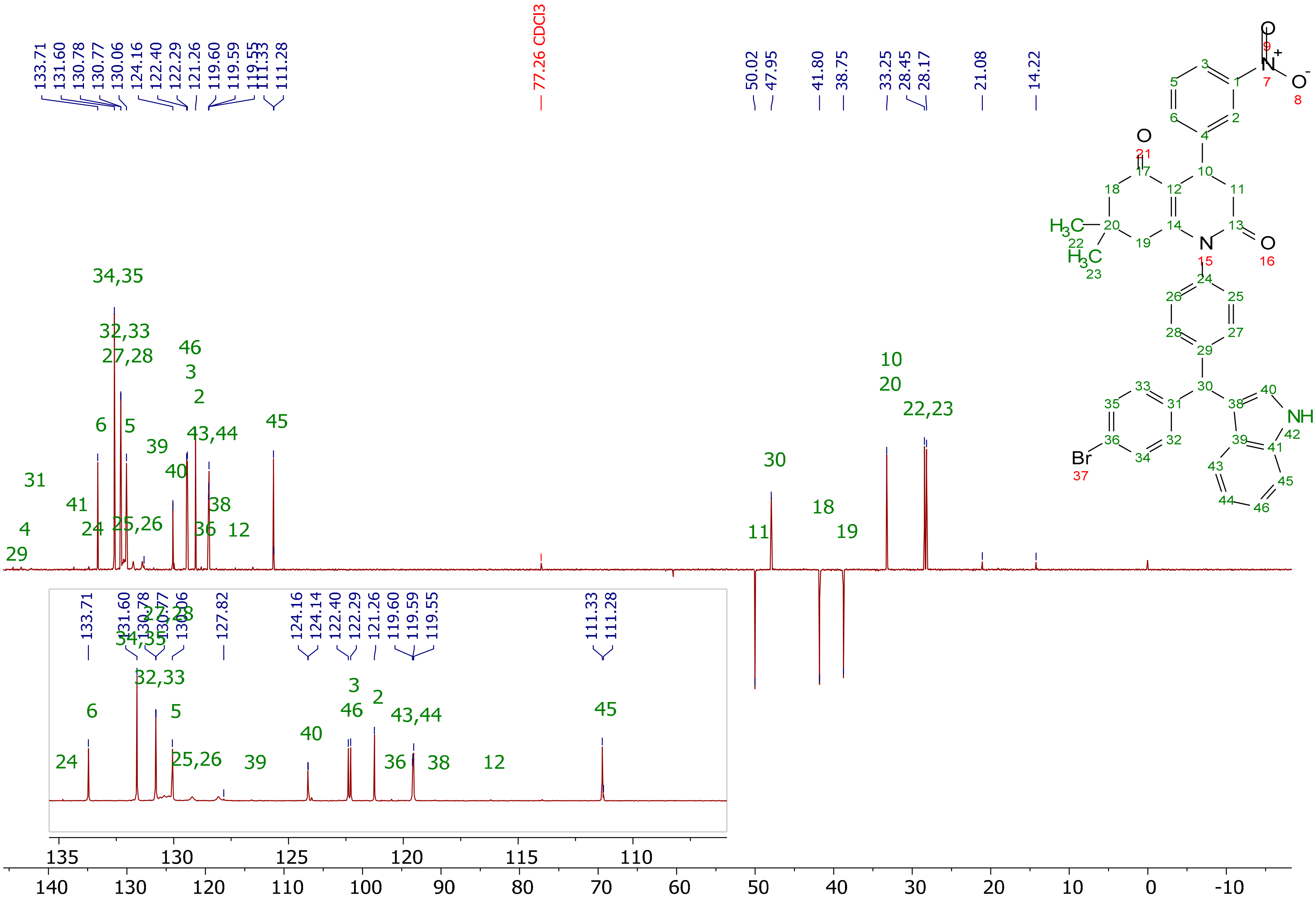

Figure S112 - DEPT spectrum of 12

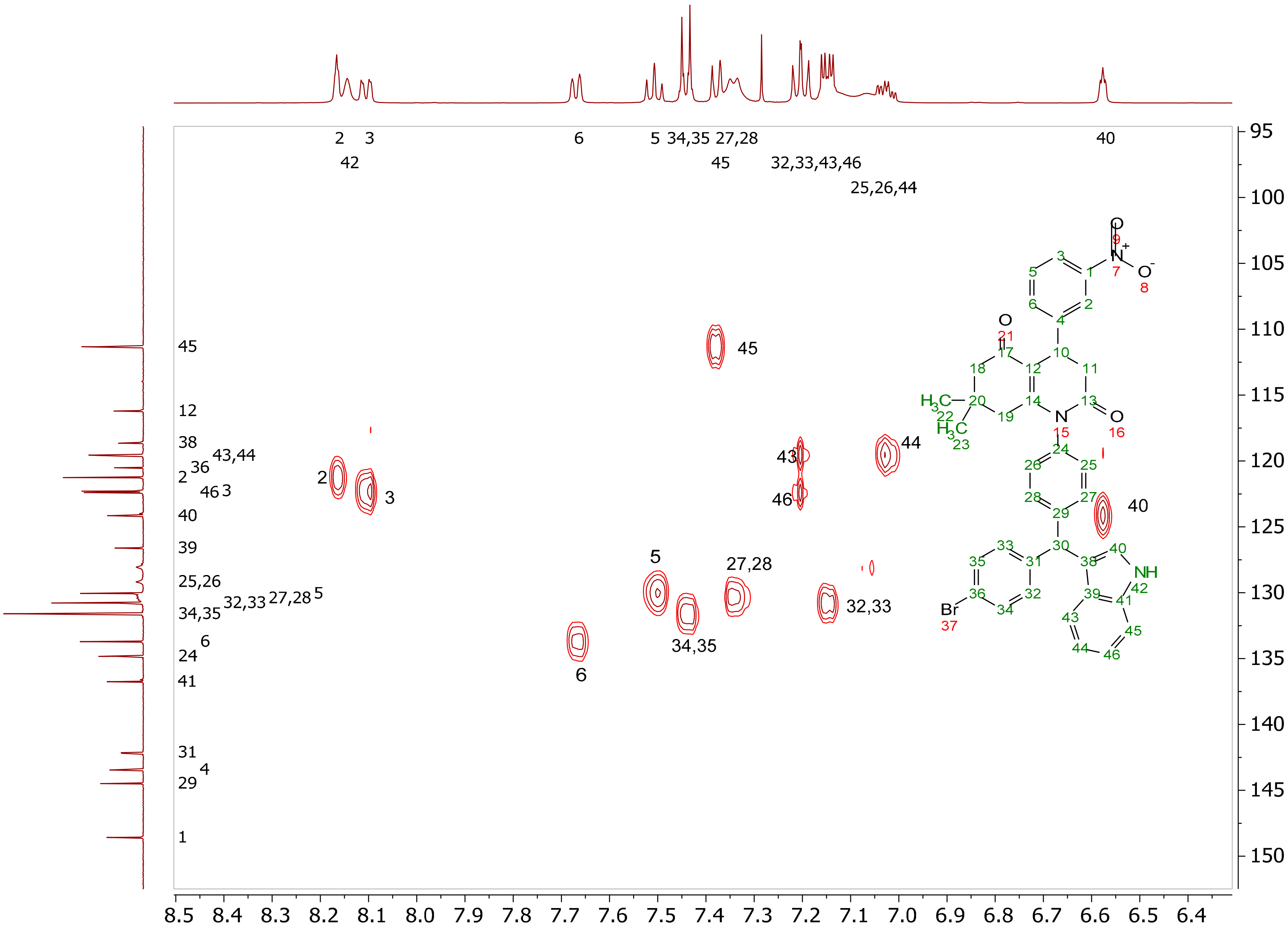

Figure S113 - HSQC spectrum of 12

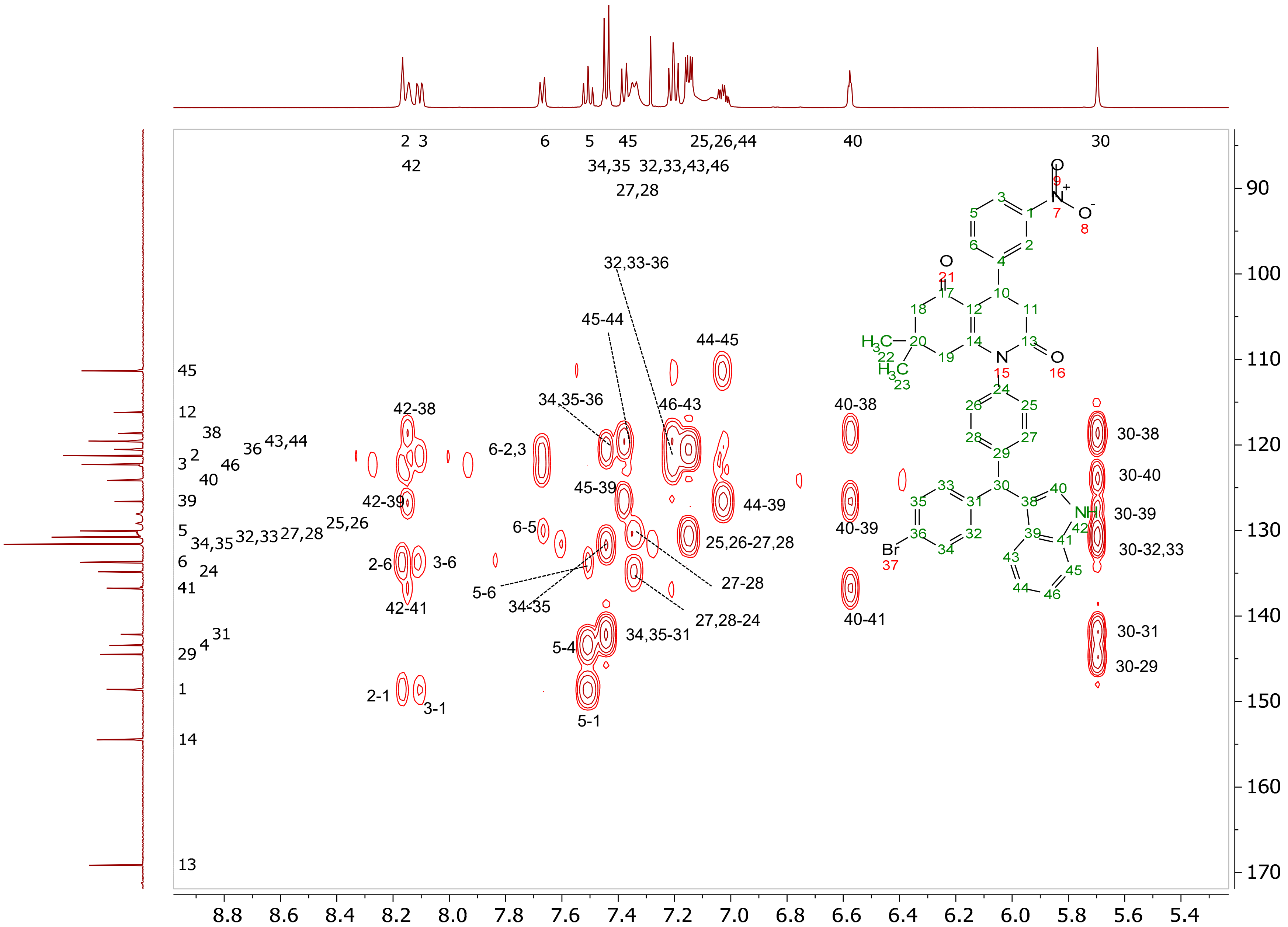

Figure S114 - Downfield region of HMBC spectrum of 12

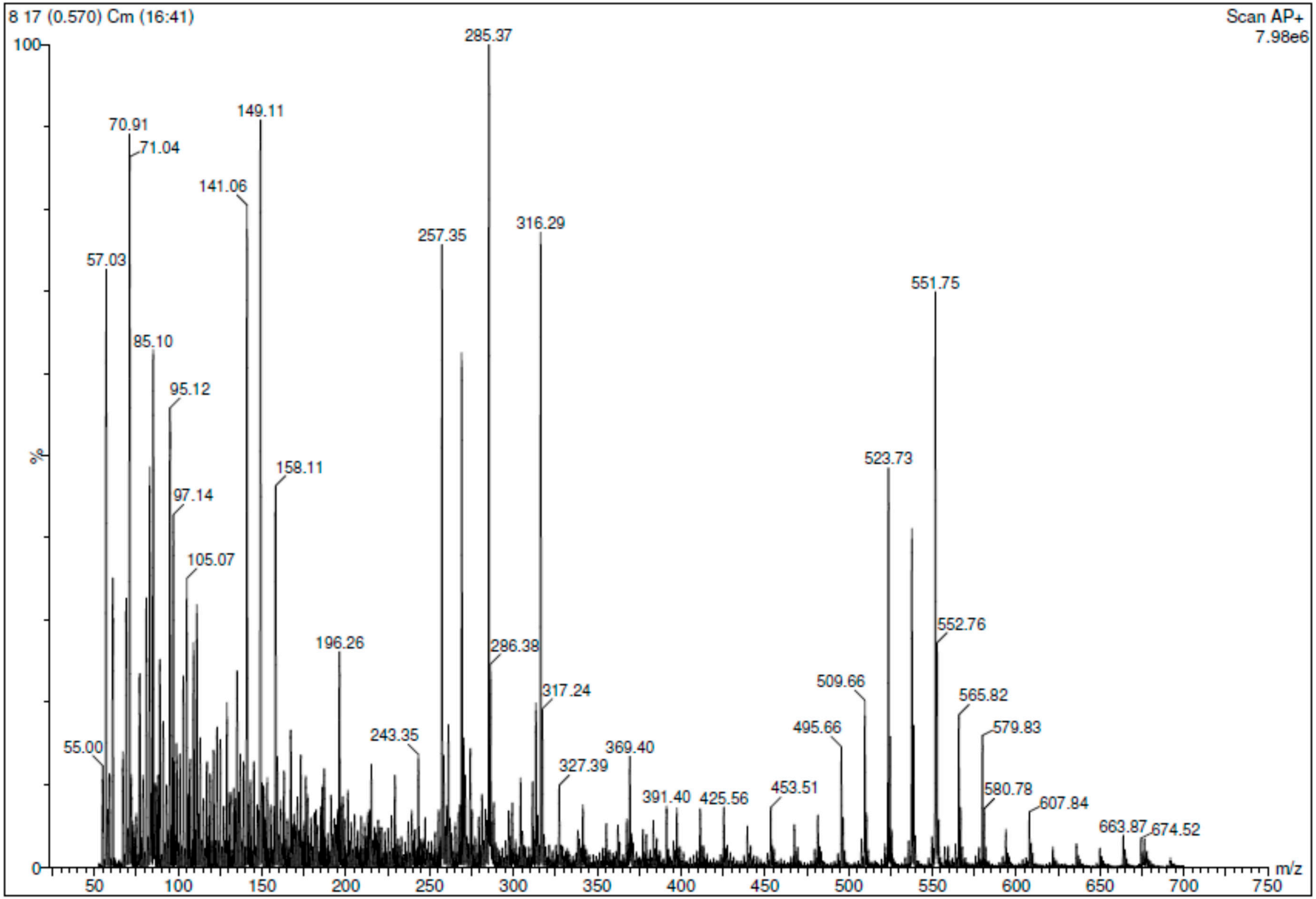

Figure S115 - MS spectrum of 12

Table S14 - Fragmentation positions for peaks in MS spectrum of 12

m/z	Fragmentation position	m/z	
**674.52**	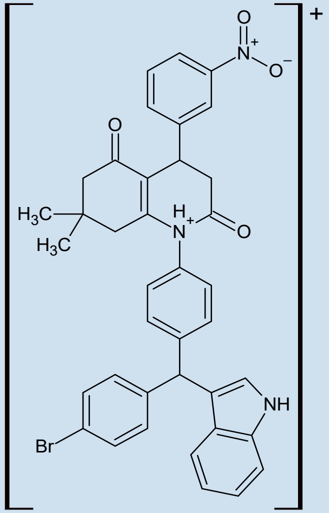	**579.83**	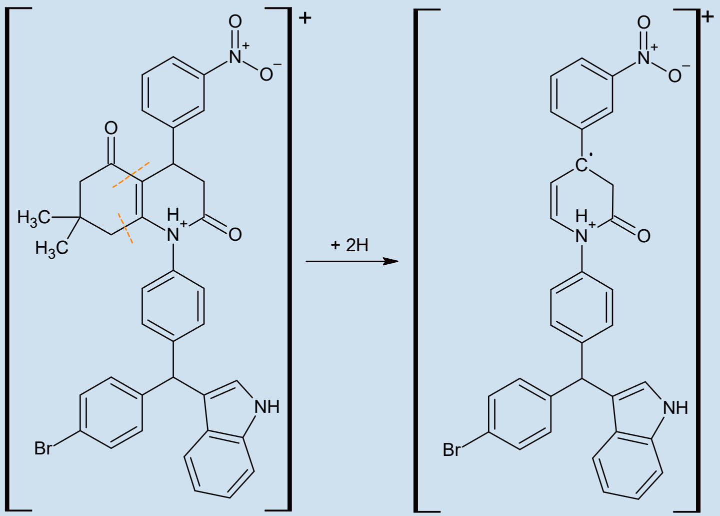
**607.84**	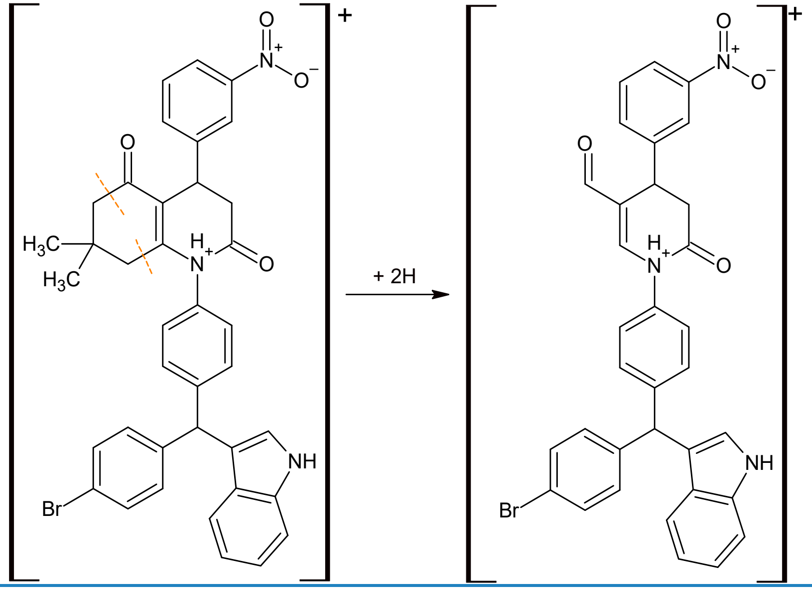	**565.82**	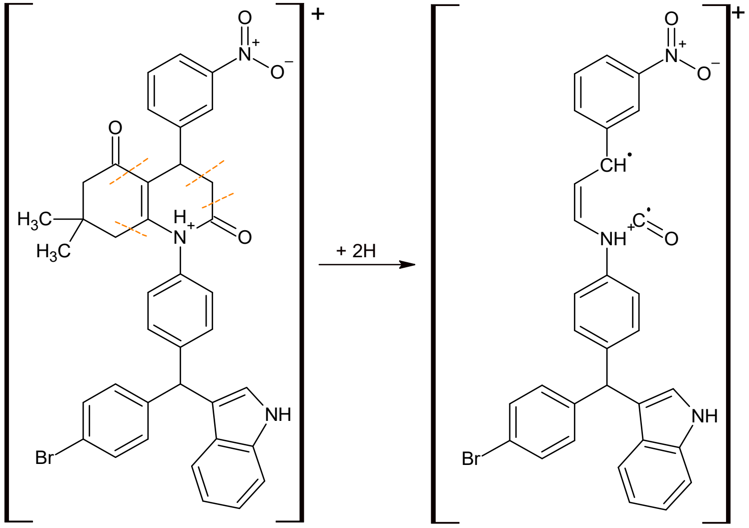

**551.75**	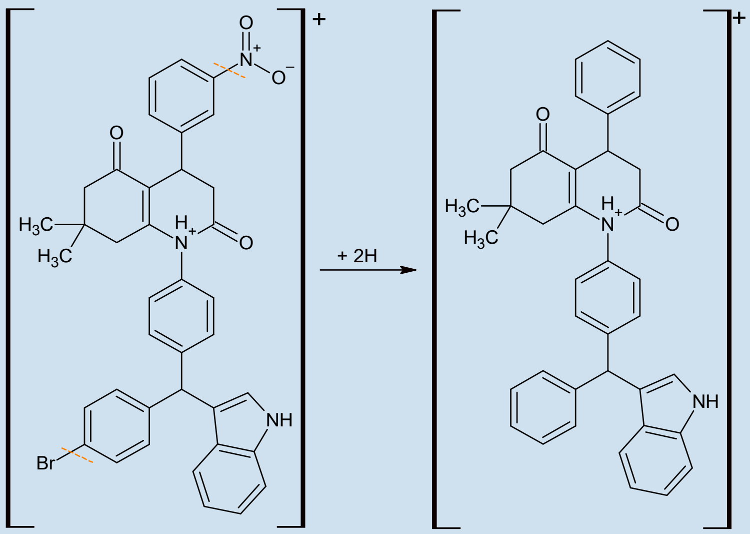	**523.73**	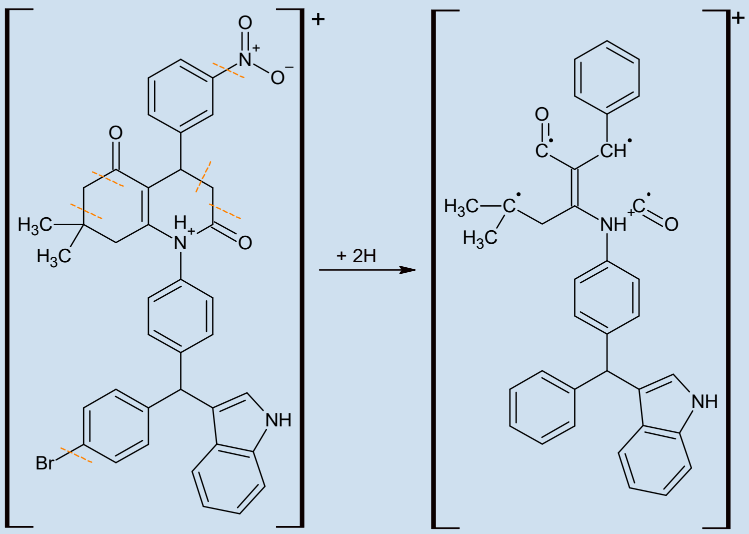
**534.46**	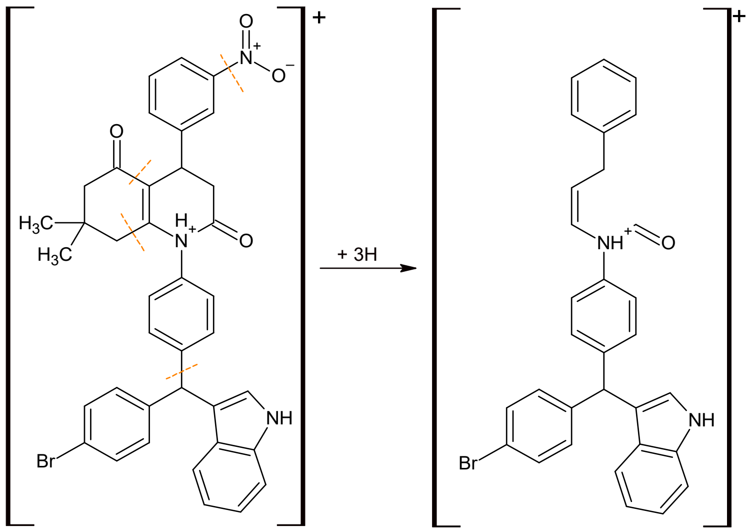	**453.51**	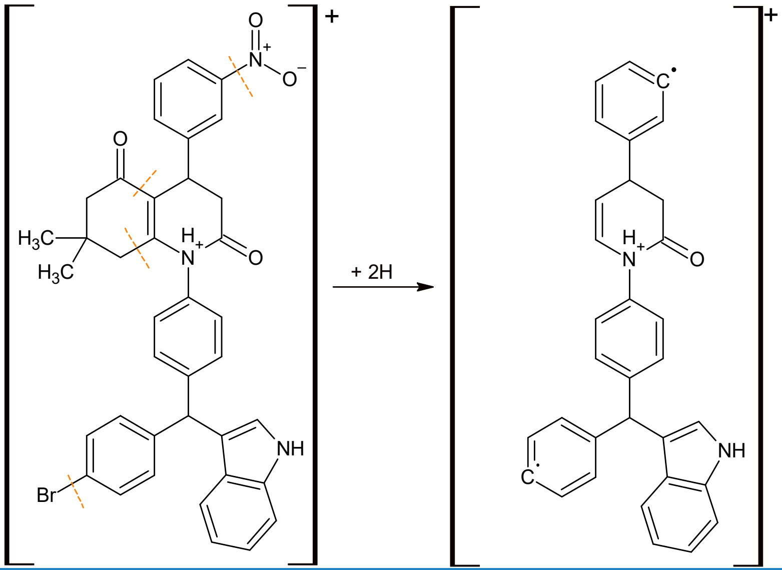

**391.40**	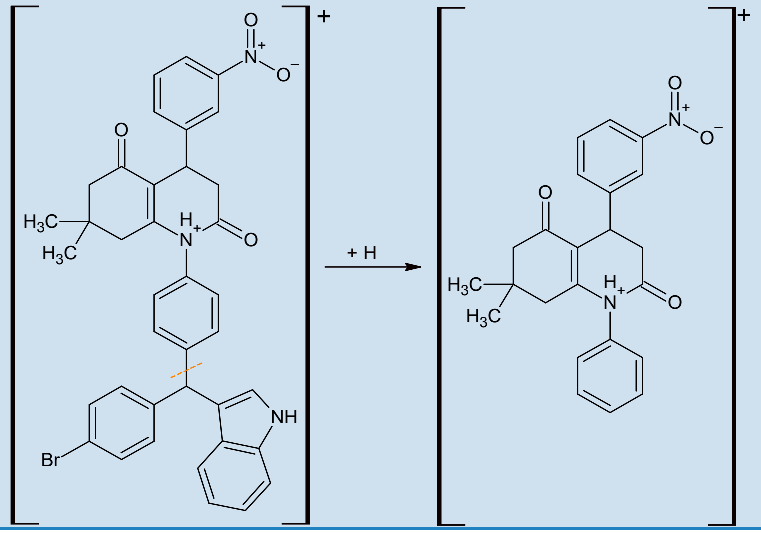			

1.29. Pip-Agar catalyst

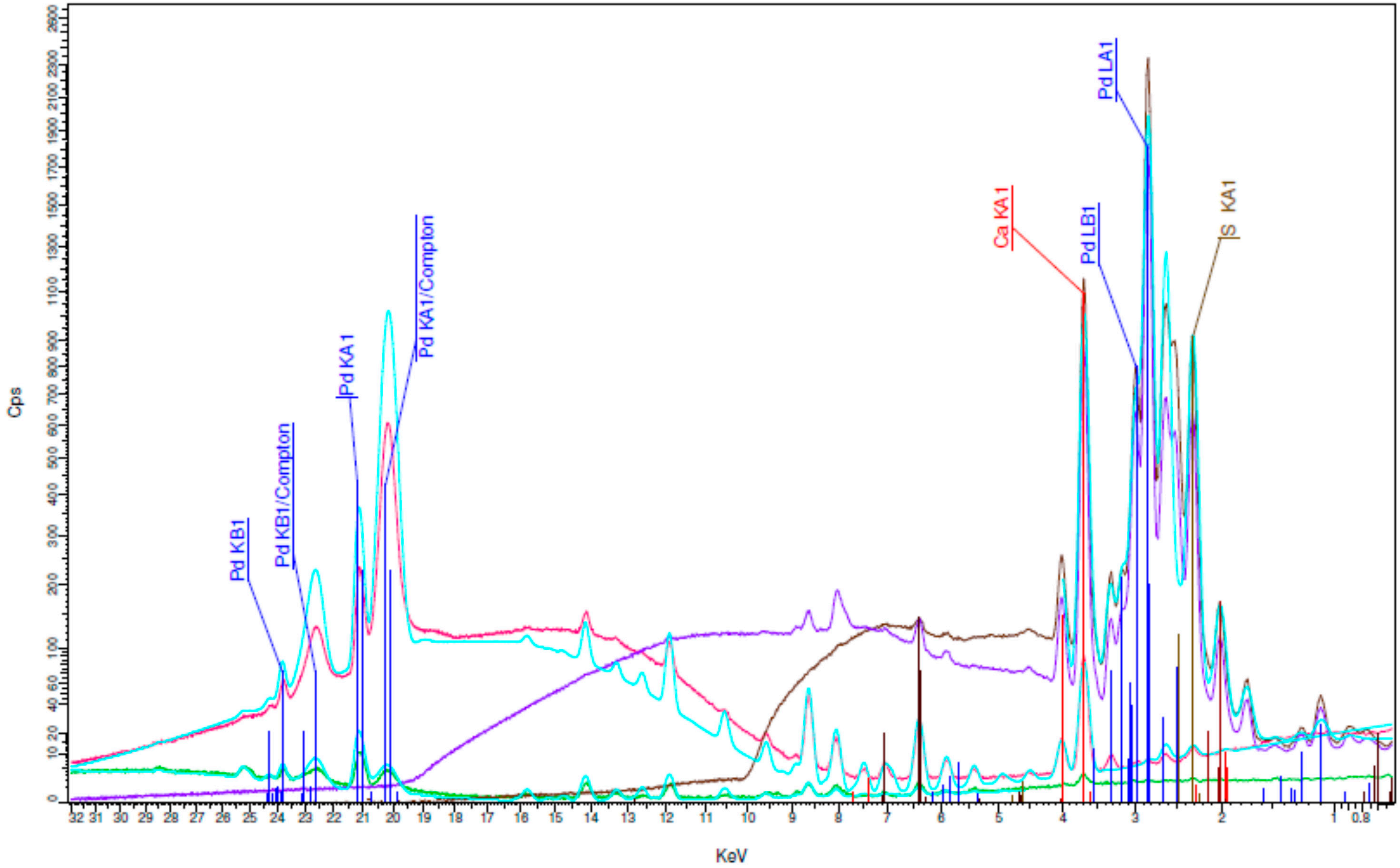

Figure S116 - XRF spectrum of Pip-Agar catalyst

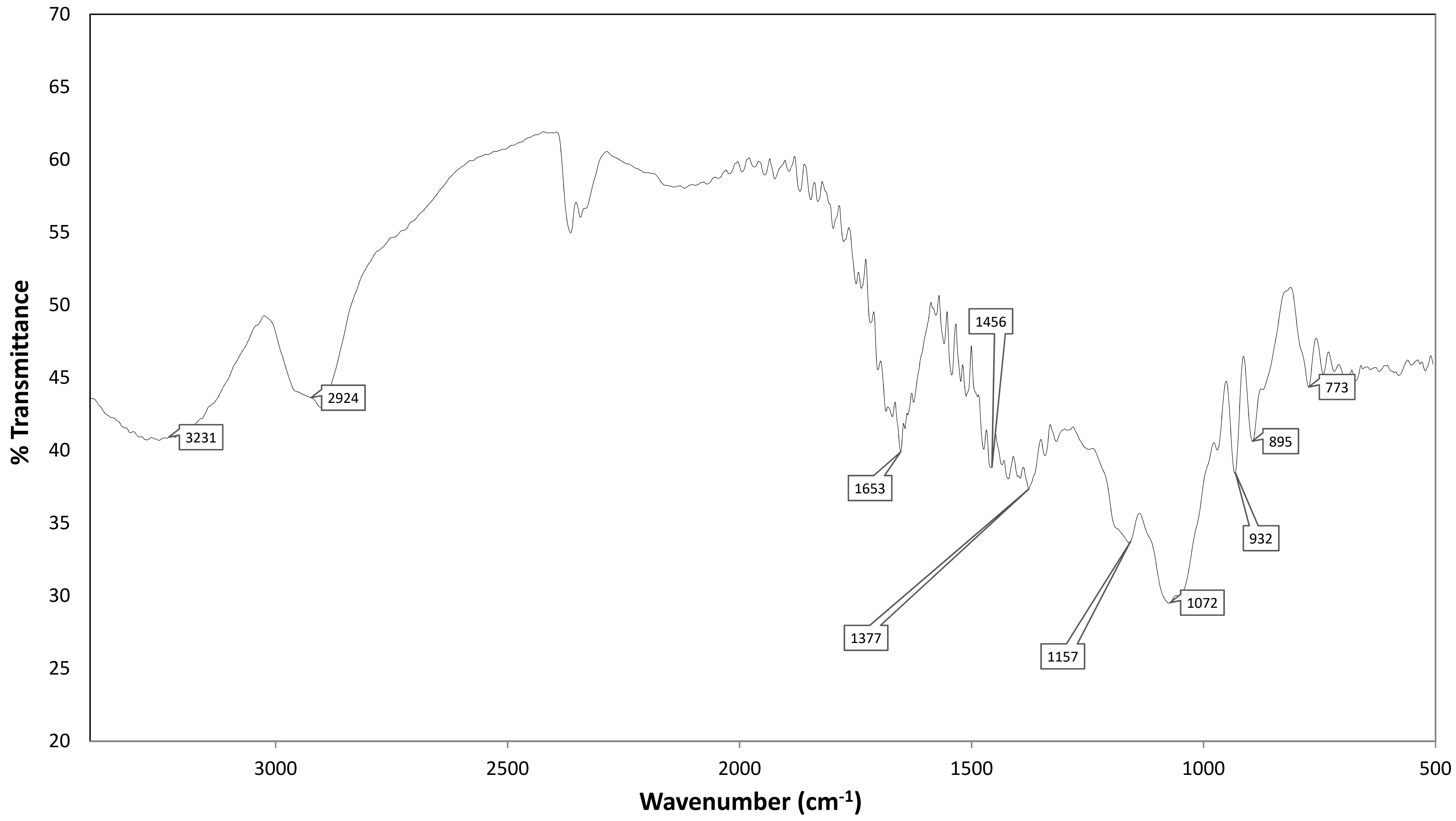

Figure S117 - IR spectrum of agar

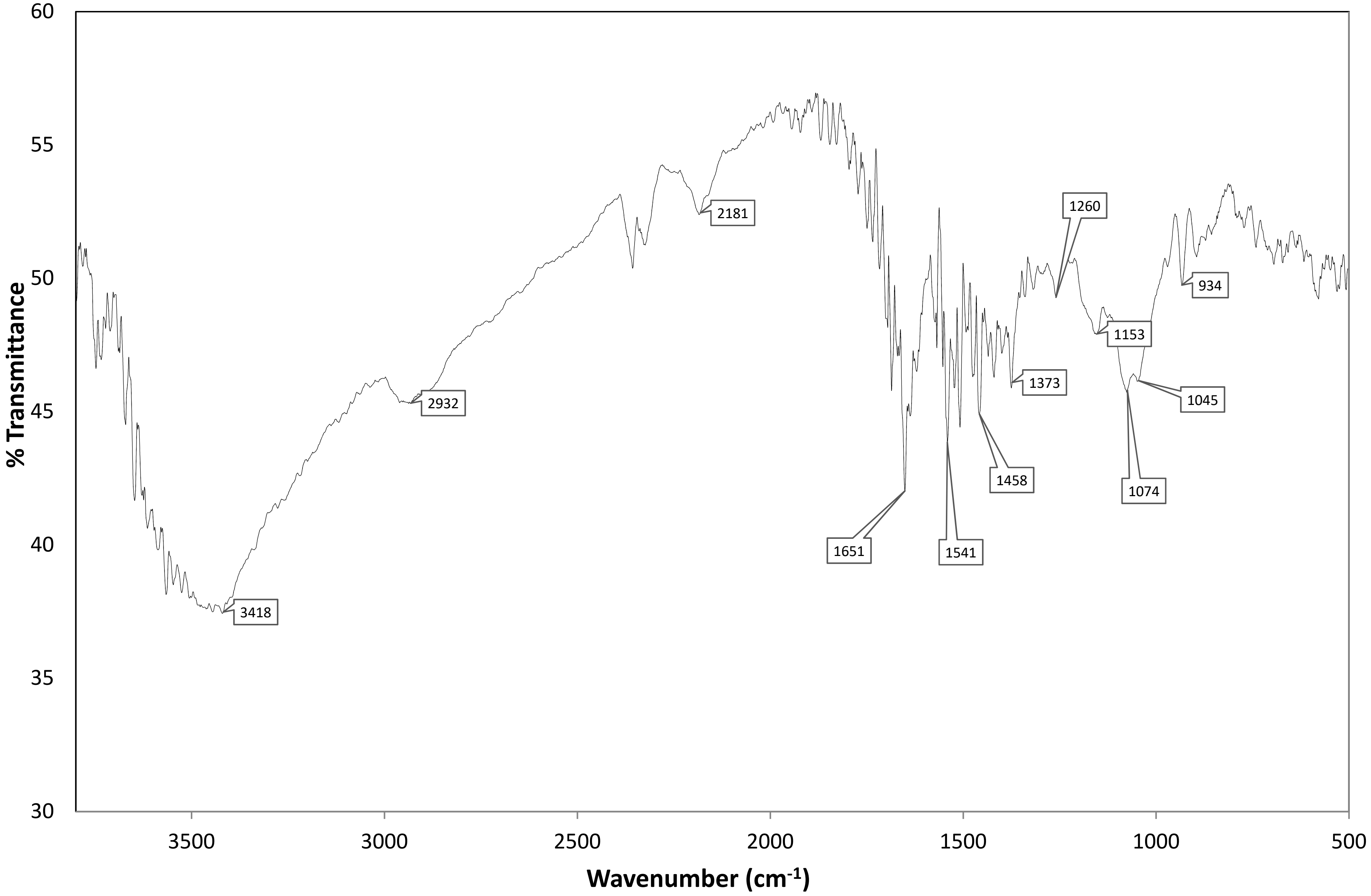

Figure S118 - IR spectrum of fresh Pip-Agar
